# Rh-catalyzed desymmetrization of α-quaternary centers by isomerization-hydroacylation†
†Electronic supplementary information (ESI) available: Materials and methods, reaction procedures, characterization data. CCDC 1056687, 1062118. For ESI and crystallographic data in CIF or other electronic format see DOI: 10.1039/c5sc01553g



**DOI:** 10.1039/c5sc01553g

**Published:** 2015-06-12

**Authors:** Jung-Woo Park, Kevin G. M. Kou, Daniel K. Kim, Vy M. Dong

**Affiliations:** a Department of Chemistry , University of California-Irvine , 4403 Natural Sciences 1, Irvine , California 92697 , USA . Email: dongv@uci.edu

## Abstract

We describe a Rh-catalyzed desymmetrization of all-carbon quaternary centers from α,α-bis(allyl)aldehydes by a cascade featuring isomerization and hydroacylation. This desymmetrization competes with two other novel olefin functionalizations that are triggered by C–H bond activation, including carboacylation and bisacylation. A BIPHEP ligand promotes enantioselective formation of α-vinylcyclopentanones. Mechanistic studies support irreversible and enantioselective olefin-isomerization followed by olefin-hydroacylation.

## Abstract 2

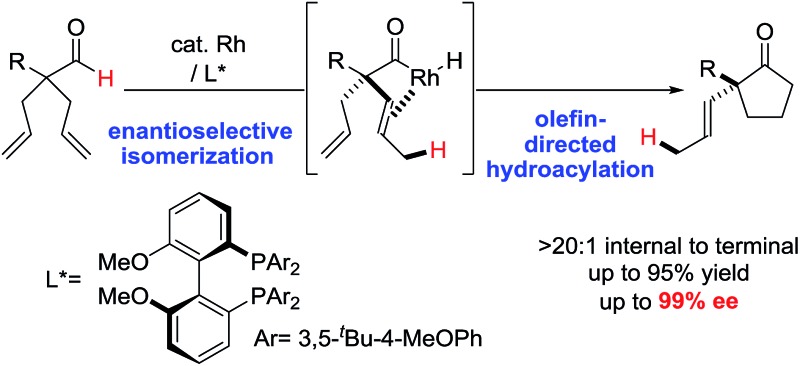
A Rh-catalyzed desymmetrization of α,α-bis(allyl)aldehydes occurs by enantioselective isomerization followed by olefin-directed hydroacylation.

## Introduction

**Fig. 1:**
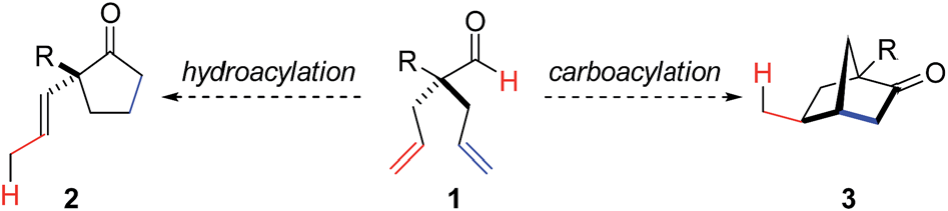
Two pathways to quaternary-carbon motifs from desymmetrization of **1**.

Desymmetrization has emerged as a way to access chiral quaternary-carbon motifs, which are among the most challenging stereocenters to generate with enantiocontrol.^1–4^ Strategies involving C–H bond activation are especially promising yet rare.^5,6^ Given this challenge, we propose that prochiral aldehyde **1** could isomerize to scaffolds bearing quaternary centers *via* two possible pathways triggered by aldehyde C–H bond activation (Fig. 1). Herein, we communicate Rh-catalyzed olefin functionalizations, including hydroacylation and carboacylation from a common aldehyde. This initial report focuses on hydroacylation of bis(allyl)aldehydes to generate α-vinylcyclopentanones **2** bearing quaternary stereocenters.^7^ Mechanistic studies reveal a cascade process featuring an enantioselective olefin-isomerization followed by olefin-hydroacylation.

**Fig. 2:**
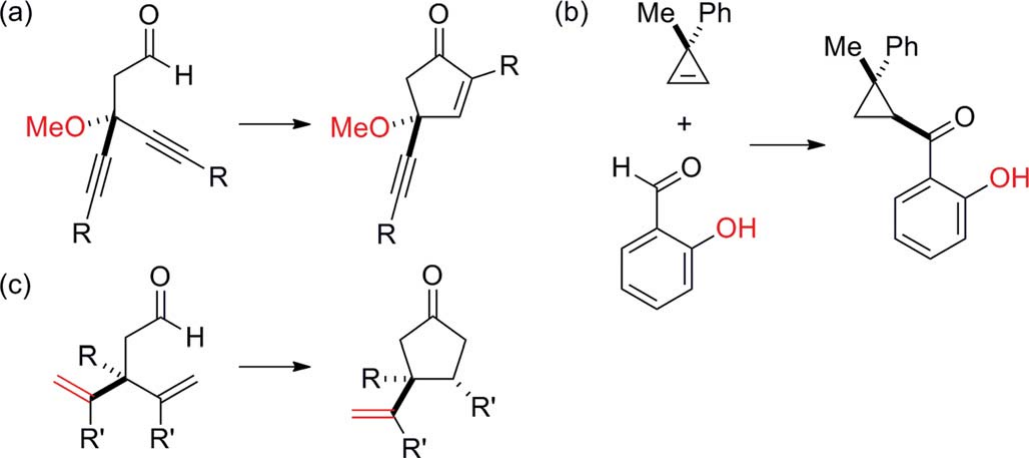
Previous desymmetrizations by hydroacylation result in ketones bearing β-quaternary stereocenters.

The use of oxygen, nitrogen, and sulfur-based functional groups has allowed breakthroughs in enantioselective Rh-catalyzed hydroacylation.^8^ These heteroatoms act as directing groups by binding to rhodium and favoring C–H bond activation while accelerating hydroacylation over competitive pathways, such as decarbonylation or catalyst decomposition.^9^ Fu demonstrated intramolecular hydroacylation of alkynals bearing β-methoxy groups (Fig. 2a).^10^ Our laboratory reported intermolecular hydroacylation of cyclopropenes using chelating aldehydes, specifically salicylaldehyde derivatives (Fig. 2b).^5^ Given their ability to bind Rh, we reasoned that olefins could be used as directing groups for hydroacylation.^11^ We were encouraged that Tanaka and Suemune reported desymmetrization of β-bis(alkenyl) aldehydes (Fig. 2c).^12^ Although not proposed, we reason that the pendant olefin in their substrate could be acting as a directing group. These previous desymmetrizations by hydroacylation generate ketones bearing β-quaternary stereocenters. Given this limitation, we chose to develop a complementary desymmetrization of α-trisubstituted aldehydes, which represents a sterically hindered and thus, challenging substrate class.^13b–c,14^ If successful, our strategy would allow access to cyclopentanones bearing α-quaternary centers, whereby the pendant olefin serves as both a directing group and versatile handle for further elaboration.

## Results and discussion

To test our proposal, we studied the desymmetrization of model **1a**, which can be prepared in one-step from commercially available phenylacetaldehyde.^15^ Aldehyde **1a** was subjected to cationic Rh(i)-catalysts and various bidentate phosphine ligands that are known to promote formyl C–H bond activation.^16^ We imagined that oxidative addition followed by alkene insertion would generate metallacycle **5**, which could diverge into various scaffolds (Table 1). The choice of phosphine ligand had a dramatic impact on product outcome and enabled chemoselective formation of two major products, cyclopentanone **2a** and bicyclo[2.2.1]heptanone **3a**.

**Table 1:** Divergent pathways for desymmetrization of **1a** based on ligand-choice^*a*^

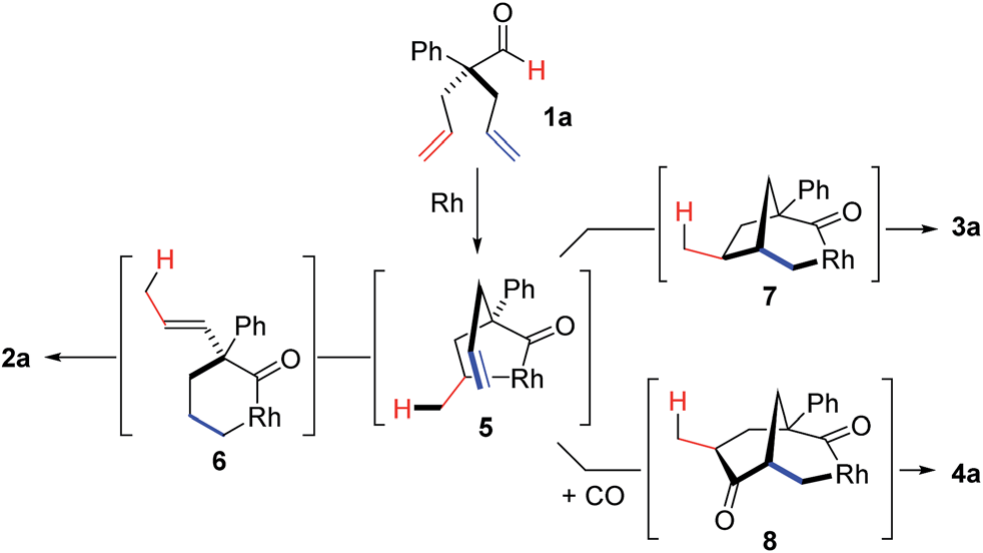			
Entry	Ligand	Major product, yield^*c*^	Minor product(s), yield^*d*^
1	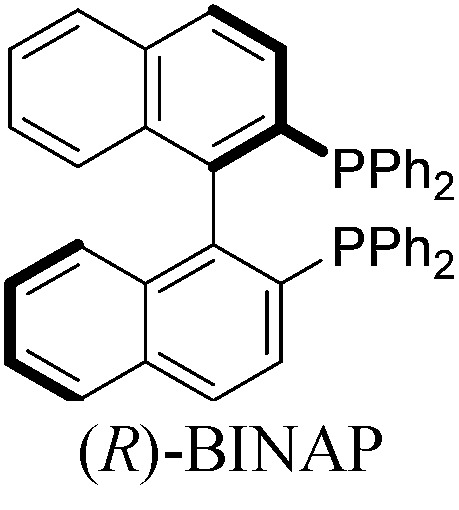	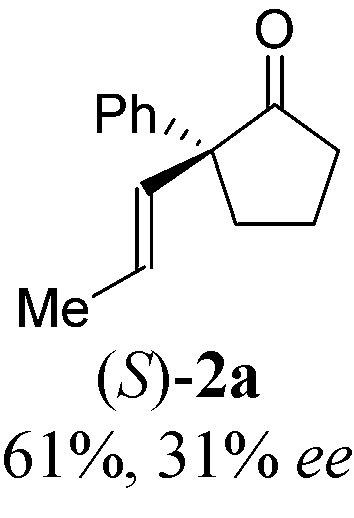	**3a** 19%, 33% *ee*
2	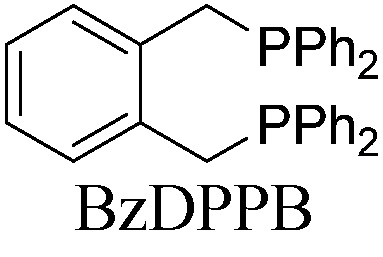	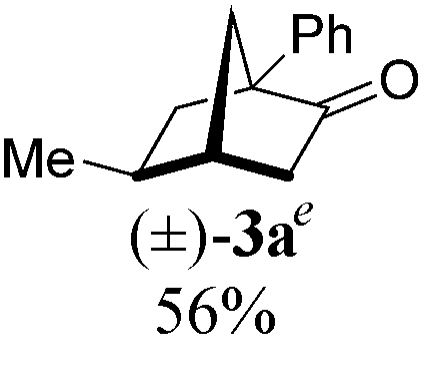	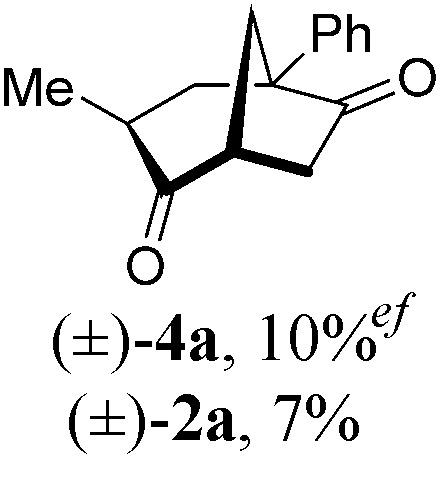
3^*b*^	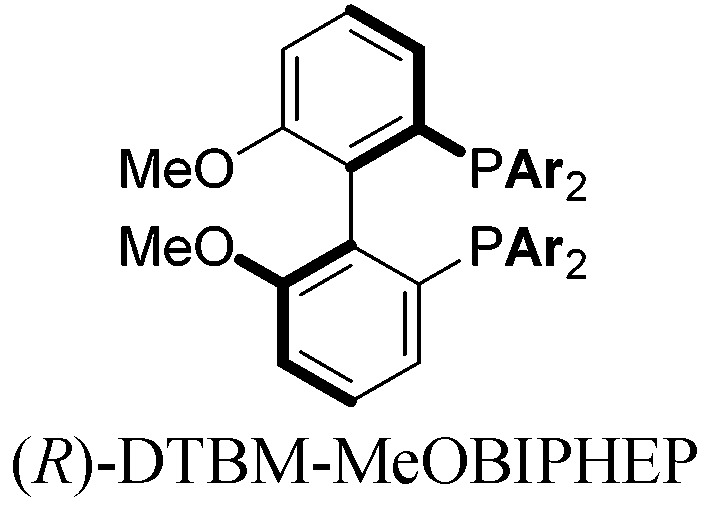	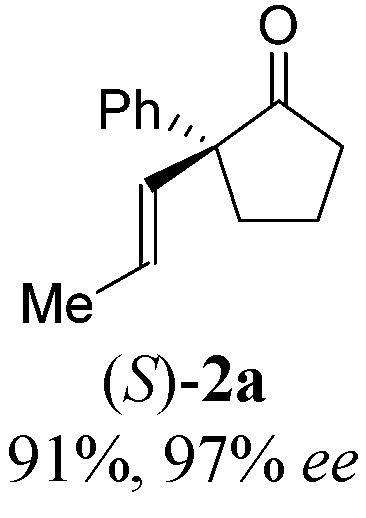	**3a** and **4a** not observed

^*a*^Reaction conditions: [(coe)_2_RhCl]_2_ (5 mol%), ligand (10 mol%), AgBF_4_ (10 mol%), DCE (0.2 M), 40 °C, 36 h.

^*b*^Reaction conditions: [(coe)_2_RhCl]_2_ (2.5 mol%), ligand (5 mol%), AgBF_4_ (5 mol%), 40 °C, 4 h.

^*c*^Isolated yield.

^*d*^Determined by GC-FID.

^*e*^>20 : 1 *dr*, determined by ^1^H NMR.

^*f*^One equivalent of **1a** was used as a CO donor. DTBM: 3,5-di(*tert*-butyl)-4-methoxyphenyl.

With a BINAP-ligated rhodium catalyst, we observed formation of both **2a** and **3a** in 61% and 19% yields, respectively (entry 1). We discovered that the hydroacylation product, cyclopentanone **2a**, bears an internal olefin, which presumably results from isomerization of the terminal olefin. Carbometallation of the pendant olefin from **5** results in intermediate **7**, which undergoes reductive elimination to form bicycloheptanone **3a** as a minor product. Use of BzDPPB ligand, however, favors the carboacylation pathway to generate **3a** as the major product in 56% yield with high diastereoselectivity (>20 : 1, entry 2). This unique olefin functionalization takes advantage of C–H activation rather than strained C–C activation to achieve carboacylation.^17^ While our study was in progress, Aïssa reported a related carbocyclization using pyridyl directing groups.^18^ We also observed bicyclo[3.2.1]octadione **4a** as a minor product in 10% yield (entry 2). The molecular structure of this homologated ketone **4a** was confirmed by X-ray crystallography (see ESI†). We believe that the second carbonyl arises from a disproportionation process where a second equivalent of aldehyde **1a** undergoes decarbonylation to generate CO.

**Fig. 3:**
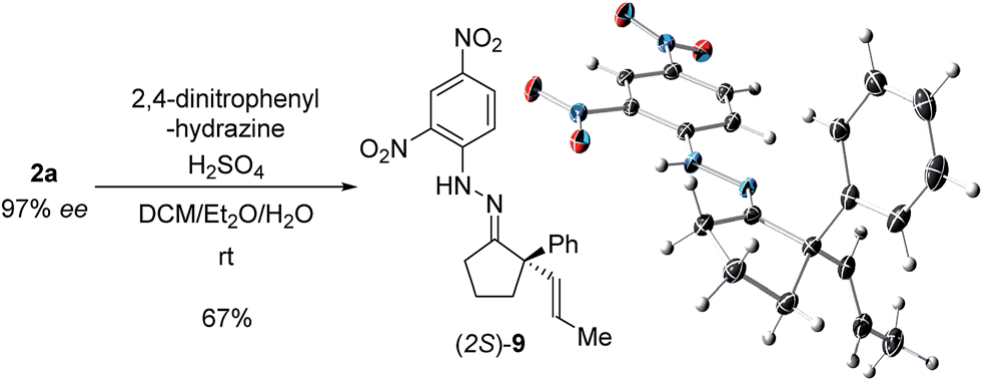
Determination of the absolute configuration of **2a** and X-ray crystal structure of (2*S*)-**9**.

With these promising leads in hand, we plan to further study each pathway and develop enantioselective variants. Towards this goal, we realized that electron-donating aromatic groups on phosphines enhance selectivity for **2a**. Among the ligands evaluated, (*R*)-DTBM-MeOBIPHEP provided the best reactivity and enantioselectivity for **2a** (entry 3). The absolute configuration of cyclopentanone **2a** was determined by elaboration with 2,4-dinitrophenylhydrazine to hydrazone **9**, in which the molecular structure was established by X-ray crystallographic analysis (Fig. 3).

With this protocol, we prepared eleven cyclopentanones bearing various α-quaternary stereocenters (Table 2). Aldehydes with aromatic substituents (**1a–1g**) undergo desymmetrization in 83–91% yields and high enantioselectivities (95–99% *ee*). Ether, aryl halide, and acetal functional groups are well-tolerated. Heteroaromatic aldehyde (**1h**) as well as aldehydes bearing aliphatic substituents (**1i–1k**) rearrange to the corresponding cyclopentanones in excellent enantioselectivities albeit using increased catalyst loading at lower temperature.^19,20^

**Table 2:** Desymmetrization of α-quaternary aldehyde **1** by isomerization-hydroacylations^*a*^

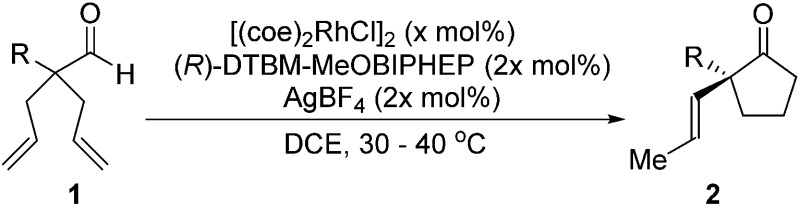
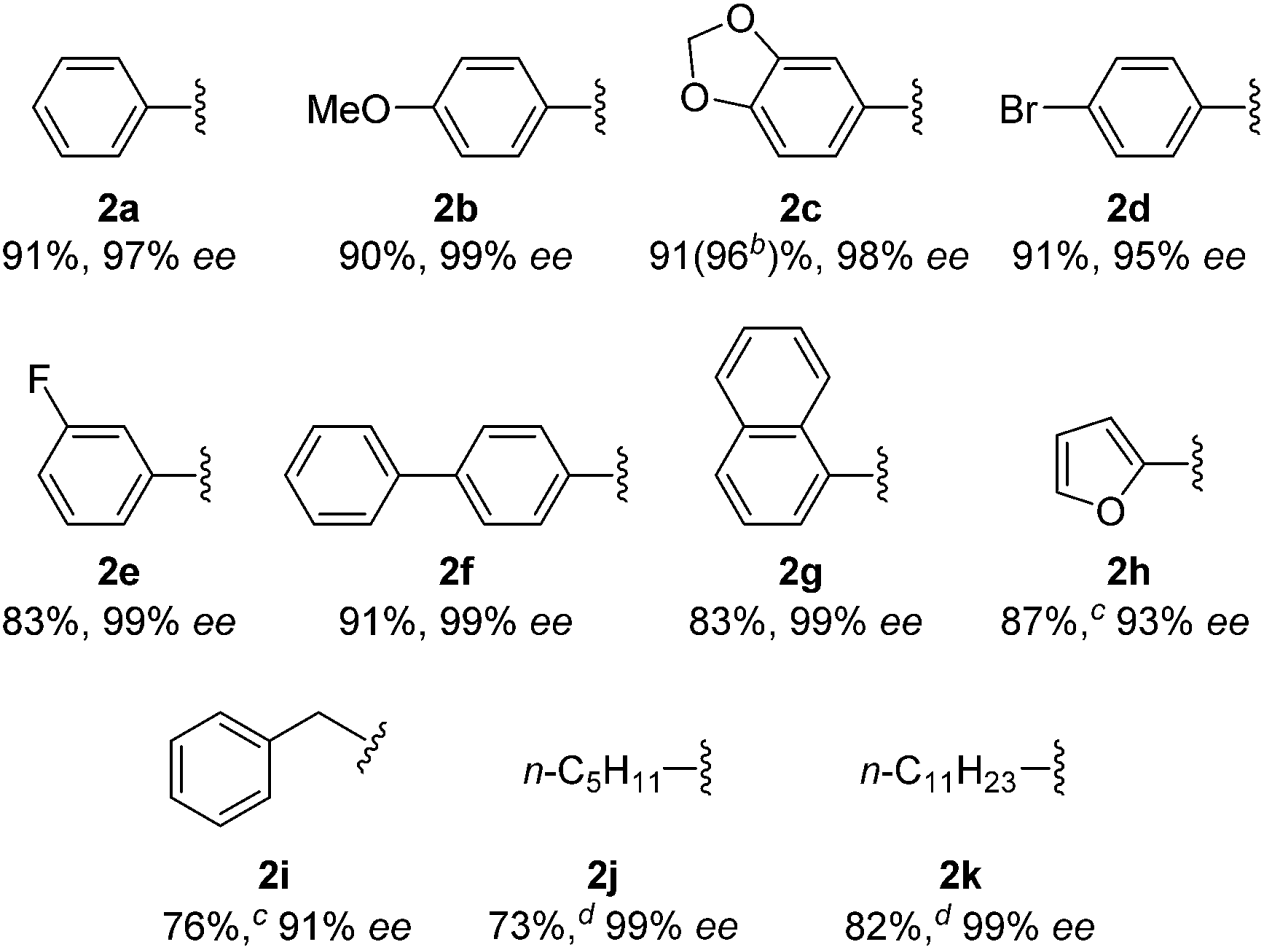

^*a*^Reaction conditions: 0.1 mmol **1**, *x* = 2.5, DCE (0.2 M), 40 °C, 4 h.

^*b*^Reaction conditions: 1 mmol **1c** used.

^*c*^Reaction conditions: *x* = 5, DCE (0.33 M), 30 °C, 2 h.

^*d*^Reaction conditions: *x* = 6, DCE (0.33 M), 30 °C, 2 h.

To understand the mechanism, we performed a deuterium-labelling study with *d*-**1a**. Desymmetrization of *d*-**1a**, under standard reaction conditions, led to exclusive formation of *d*-**2a** where the deuterium label was incorporated into the methyl group of the α-propenyl substituent (eqn (1)). This result indicates that isomerization of one allyl group takes place first through an endocyclic β-hydride elimination of a 5-membered rhodacycle *d*-**5a**.^21^ Our observations corroborate Aïssa's recent report on the isomerisation of 4-pentenals.^22^ Although β-hydride eliminations of this type are uncommon, it has been predicted that binding of a pendant alkene to the metal center significantly lowers the barrier to this process.^23^

When the reaction of *d*-**1a** was quenched at an early stage (40% conversion to *d*-**2a**), we recovered three deuterated aldehydes, *d*-**1a**, *d*-**1a′** and *d*-**1a′′** (eqn (2)). The observation of *d*-**1a′** suggests that olefin-insertion is reversible. Yet, the deuterium is incorporated into only the methyl group of the α-propenyl unit in product *d*-**2a**. This lack of deuterium scrambling on the cyclopentanone ring suggests that Rh-D insertion occurs with high enantioselectivity (with the olefin shown in red). Thus, the insertion step is both reversible and highly enantioselective.^24^

Further experiments support the notion that the α-vinyl group (formed from initial isomerization) directs hydroacylation. For example, α-trisubstituted aldehyde **10** (with only one allyl group) does not undergo hydroacylation. Instead, this aldehyde undergoes isomerization to generate α-vinyl aldehyde **11** (eqn (3)).^22^ In addition, subjecting α-allylcyclopentanone **12a** to the optimized reaction conditions results in trace formation of α-vinylcyclopentanone **2a** (eqn (4)). Thus, the cyclopentanones obtained in Table 2 must arise from an isomerization that occurs prior to hydroacylation. In contrast, we discovered that aldehyde **1l**, containing an acetal group, yields α-allylcyclopentanone **12l** as the major product (eqn (5)). In this case, we reason that the acetal acts as an oxygen-directing group which promotes hydroacylation over olefin isomerization.
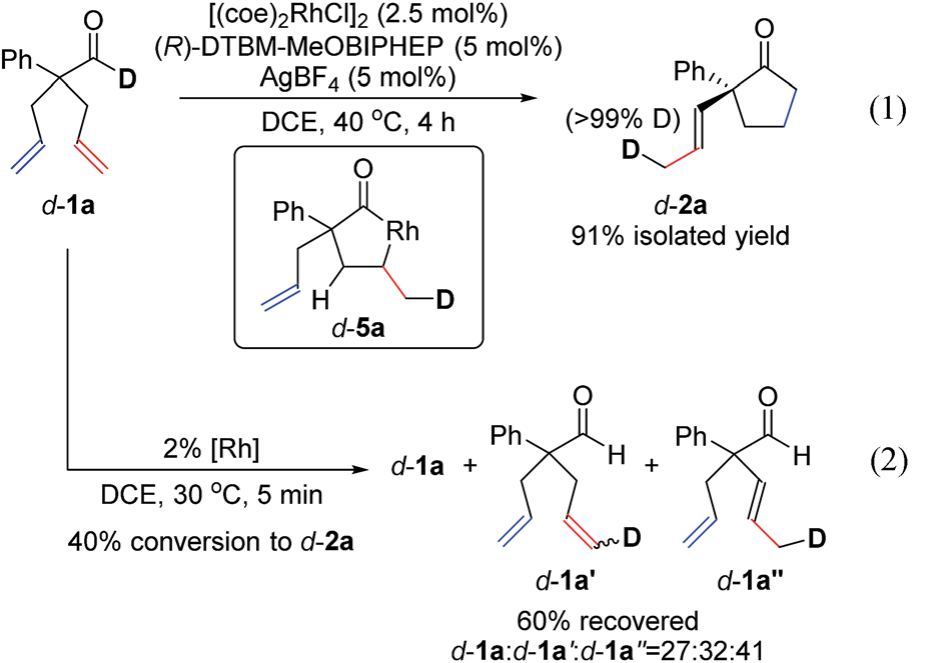
 
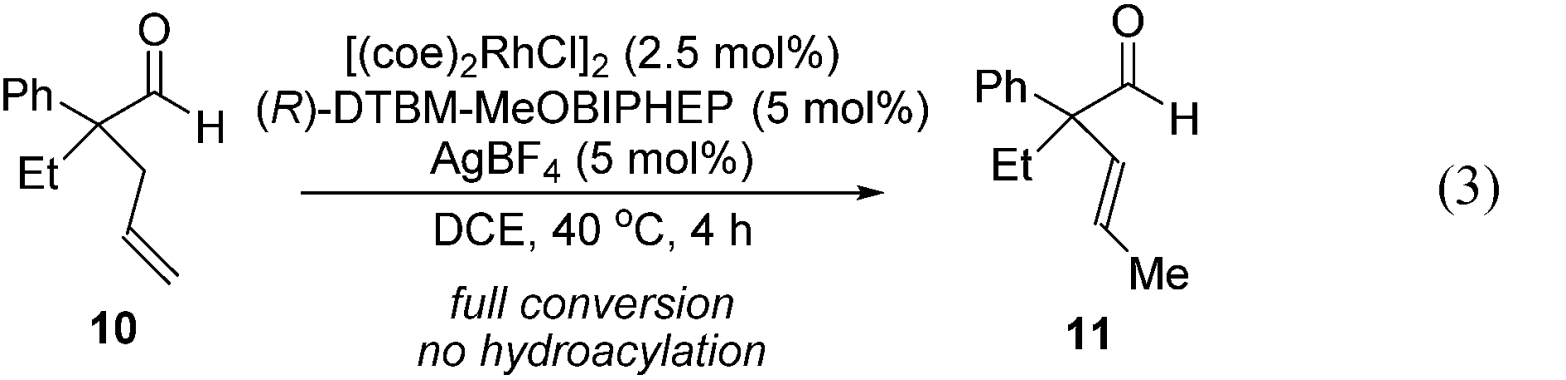
 
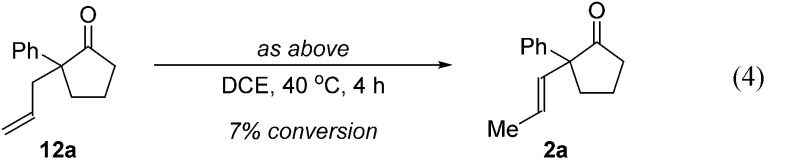
 
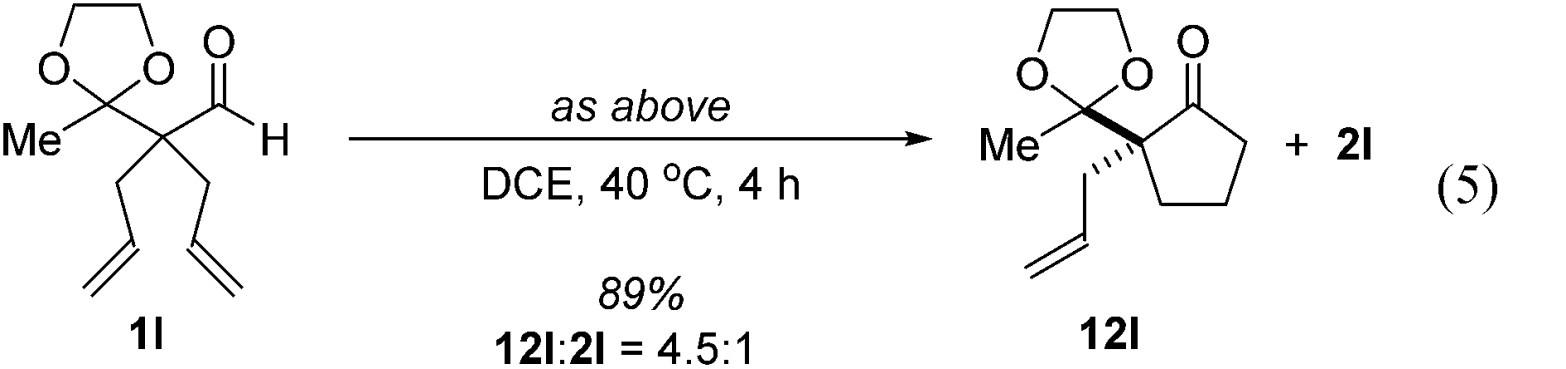


**Scheme 1:**
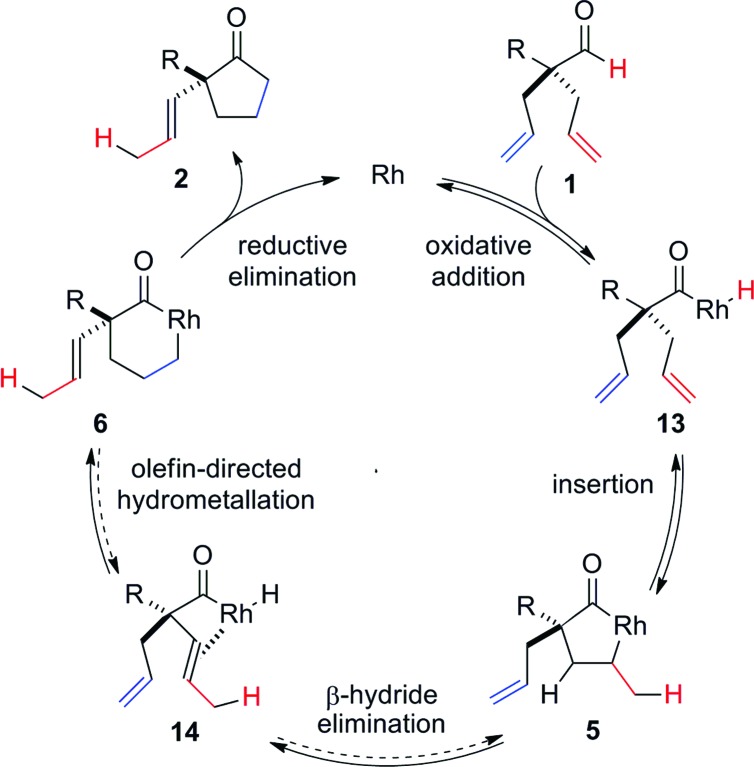
Proposed mechanism for Rh-catalyzed cascade.

On the basis of literature reports and our own observations, we propose a mechanism starting with cationic Rh(i)-complex activating the aldehyde C–H bond of **1** to form acyl-Rh(iii)-hydride **13** (Scheme 1). Insertion of the olefin into Rh(iii)-hydride **13** leads to formation of the more thermodynamically stable 5-membered metallacycle **5**.^25^ A rare endocyclic β-hydride elimination takes place to produce isomerized acyl-Rh(iii)-hydride **14**. The allyl olefin inserts into the Rh(iii)-hydride to form a 6-membered rhodacycle **6**. Finally, reductive elimination affords the cyclopentanone product **2** and regenerates the Rh(i)-catalyst.

## Conclusions

We have demonstrated a Rh-catalyzed enantioselective synthesis of α-quaternary cyclopentanones. Studies on the scope and mechanism support an olefin-assisted isomerization^23^ and olefin-directed hydroacylation cascade. While endocyclic β-hydride elimination has been proposed in the literature on the basis of theoretical studies,^21^ our results provide experimental evidence for this elementary step. The use of a BIPHEP ligand enables high selectivity for one out of three possible rearrangements, all initiated by the activation of an aldehyde C–H bond. Insights from these studies will guide efforts to understand and expand the power of the related carboacylation and bisacylation as routes to scaffolds containing chiral all-carbon stereocenters.
