# Supplementary material for: Rh-catalyzed desymmetrization of α-quaternary centers by isomerization-hydroacylation
Source: Chem Sci. 2015 Jun 12;6(8):4479–83. doi: 10.1039/c5sc01553g (PMC4618402; doi:10.1039/c5sc01553g)
Supplement: Supplementary file 1 [file SC-006-C5SC01553G-s001.pdf]

Electronic Supplementary Material (ESI) for Chemical Science.
This journal is © The Royal Society of Chemistry 2015

Electronic Supplementary Information

Rh-Catalyzed Desymmetrization of α-Quaternary Centers by Isomerization-Hydroacylation

Jung-Woo Park, Kevin G. M. Kou, Daniel K. Kim, Vy M. Dong*

<dongv@uci.edu>

†*Department of Chemistry, University of California, Irvine, California, 92697-2025, USA.*

Table of Contents: **Page**

1.	General Considerations	S2
2.	Rh-Catalyzed Desymmetrization of α,α-Bisallylaldehydes **1**	S3
3.	Preparation of Substrates	S8
4.	X-Ray Crystallographic Data for (±)-**4a** and (*S*)-**9**	S16
5.	NMR spectra	S20
6.	Chiral SFC Analysis	S62

1 General Considerations

All experiments were performed in oven-dried or flame-dried glassware under an atmosphere of N_2. Tetrahydrofuran, dichloromethane, toluene, and diethyl ether were purified using an Innovative Technologies Pure Solv system, degassed by three freeze-pump-thaw cycles, and stored over 3A MS within an N_2 filled glove box. The molarity of organolithium reagents was determined by titration with *iso*-propanol/1,10-phenanthroline. Reactions were monitored either *via* gas chromatography using an Agilent Technologies 7890A GC system equipped with an Agilent Technologies 5975C inert XL EI/CI MSD or by analytical thin-layer chromatography on EMD Silica Gel 60 F_{254} plates. Visualization of the developed plates was performed under UV light (254 nm) or using either $KMnO_4$ or *p*-anisaldehyde stain. Column chromatography was performed with Silicycle Silia-P Flash Silica Gel using glass columns. Automated column chromatography was performed using either a Biotage SP1 or Teledyne Isco CombiFlash Rf 200 purification system. ^{1}H, ^{2}D and ^{13}C spectra were recorded on a Bruker DRX-400 (400 MHz ^{1}H, 100 MHz ^{13}C, 376.5 MHz ^{19}F), GN-500 (500 MHz ^{1}H, 125.7 MHz ^{13}C) or CRYO-500 (500 MHz ^{1}H, 125.7 MHz ^{13}C) spectrometer. ^{1}H NMR spectra were internally referenced to the residual solvent signal or TMS. ^{13}C NMR spectra were internally referenced to the residual solvent signal. Data for ^{1}H NMR are reported as follows: chemical shift (δ ppm, δ 7.27 for $CDCl_3$), multiplicity (s = singlet, d = doublet, t = triplet, q = quartet, m = multiplet, br = broad), coupling constant (Hz), integration. Data for ^{13}C NMR are reported in terms of chemical shift (δ ppm, δ 77.16 for $CDCl_3$). Infrared spectra were obtained on a Thermo Scientific Nicolet iS5 FT-IR spectrometer equipped with an iD5 ATR accessory. Enantiomeric excesses for stereoselective reactions were determined by chiral SFC analysis using an Agilent Technologies HPLC (1200 series) system and Aurora A5 Fusion. High resolution mass spectrometry (HRMS) was performed by the University of California, Irvine Mass Spectrometry Center. X-ray crystallography was performed by the University of California, Irvine, X-ray Crystallography Facility.

2 Rh-Catalyzed Desymmetrization of α,α-Bisallylaldehydes 1

Study on Ligand Effects for Desymmetrization of 1a (Table 1)

In a nitrogen-filled glove box, a 1-dram vial was charged with the indicated amount of $[(coe)_2RhCl]_2$, bisphosphine ligand, internal standard (durene), and 1,2-dichloroethane. The solution was stirred at ambient temperature (30 °C) for 30 minutes to until homogeneous. Next, $AgBF_4$ was added and the resulting mixture was stirred for additional 5 minutes prior to addition of the α,α-bisallylaldehyde **1a**. The vial was then sealed with a Teflon-lined screw cap, and the reaction mixture was stirred for the indicated reaction time. Reaction progress and chemoselectivity were determined from analysis of the GC-FID chromatogram or ^{1}H NMR spectrum of the reaction mixture. The carbonyl products were isolated by preparative TLC.

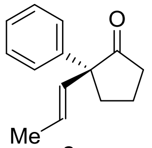

2a

(*S*)-(–)-2-Phenyl-2-(prop-1-en-1-yl)cyclopentan-1-one (2a)

The product **2a** was obtained by purification using preparative TLC (eluting with 20:1 hexanes/ethyl acetate) and isolated as a colorless oil (18.2 mg, 91%). ^{1}H NMR (400 MHz, $CDCl_3$) δ 7.37–7.28 (m, 4H), 7.26–7.20 (m, 1H), 5.64–5.56 (m, 1H), 5.50 (dq, *J* = 15.6, 6.1 Hz, 1H), 2.50–2.27 (m, 4H), 2.02–1.84 (m, 2H), 1.78–1.68 (m, 3H); ^{13}C NMR (101 MHz, $CDCl_3$) δ 218.4, 141.7, 132.8, 128.5, 127.4, 127.0, 126.9, 60.3, 38.1, 36.8, 19.0, 18.3; IR (ATR): 3024, 2961, 1735, 1598, 968, 755, 697 cm^{-1}; HRMS (ESI-TOF) *m / z* calcd for $C_{14}H_{16}ONa$ $[M + Na]^+$: 223.1099, found: 223.1091. SFC analysis: 97% *ee*, 150 mm CHIRALCEL OD-H, 2% *i*PrOH, 2.5 mL/min, 220 nm, 44 °C, nozzle pressure = 200 bar CO_2, t_{R1} (major) = 1.68 min, t_{R2} (minor) = 1.34 min. $[\alpha]_D^{24}$ –5.1 (*c* 0.880, $CHCl_3$).

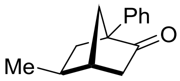

3a

***rac*-5-Methyl-1-phenylbicyclo[2.2.1]heptan-2-one (3a)**

Using BzDPPB as the ligand, the product **3a** was obtained by purification using preparative TLC (eluting with 20:1 hexanes/ethyl acetate) and isolated as a colorless oil (11.2 mg, 56%). The ^{1}H and ^{13}C NMR spectra matched the literature reported values.[1] ^{1}H NMR (500 MHz, $CDCl_3$) δ 7.38–7.32 (m, 2H), 7.27 (s, 2H), 2.39–2.30 (m, 2H), 2.28–2.20 (m, 1H), 2.20–2.10 (m, 3H), 2.04 (dd, *J* = 12.5, 6.9 Hz, 1H), 1.50 (dd, *J* = 12.9, 4.7 Hz, 1H), 1.14 (d, *J* = 7.0 Hz, 3H); ^{13}C NMR (126 MHz, $CDCl_3$) δ 216.0, 138.2, 128.3, 127.7, 127.1, 62.5, 46.4, 40.3, 40.1, 38.9, 36.2, 22.3.

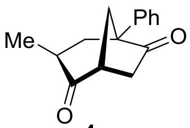

4a

***rac*-3-Methyl-5-phenylbicyclo[3.2.1]octane-2,6-dione (4a)**

The product **4a** was obtained by purification using preparative TLC (eluting with 20:1 hexanes/ethyl acetate) and isolated as a colorless oil (x.x mg, 10%). ^{1}H NMR (500 MHz, $CDCl_3$) δ 7.45 (dd, *J* = 8.3, 1.1 Hz, 2H), 7.39 (dd, *J* = 10.4, 4.9 Hz, 2H), 7.31 (t, *J* = 7.3 Hz, 1H), 3.24–3.18 (m, 1H), 2.82–2.75 (m, 1H), 2.75–2.68 (m, 1H), 2.64 (dt, *J* = 12.7, 6.5 Hz, 1H), 2.58 (dd, *J* = 18.7, 3.2 Hz, 1H), 2.51–2.45 (m, 1H), 2.42 (dd, *J* = 12.5, 2.5 Hz, 1H), 1.75 (t, *J* = 12.5 Hz, 1H), 1.13 (d, *J* = 6.4 Hz, 3H); ^{1}H NMR (500 MHz, $CDCl_3$) δ 215.6, 211.4, 139.5, 128.6, 127.5, 126.8, 54.8, 46.0, 45.3, 43.8, 42.5, 39.0, 14.7; IR (ATR): 3063, 3026, 2962, 2924, 2852, 1739, 1711, 1602, 1110, 1050, 928, 766, 704 cm^{-1}. The chemical structure was unambiguously determined by single crystal X-ray diffraction.

[1] C. Aïssa, K. Y. T. Ho, D. J. Tetlow, M. Pin-Nó, *Angew. Chem. Int. Ed.* 2014, **53**, 4209.

Synthesis of hydrozone 9 (Figure 2)

Cyclopentanone **2a** (30 mg, 0.15 mmol, 97% *ee*) and 2,4-dinitrohydrazone (52 mg, 0.26 mmol, 1.7 equiv.) was dissolved in the solution mixture of DCM (1.5 ml), ethanol (1.5 ml) and water (0.5 ml). Then, H_2SO_4 (100 μl) was added dropwise, and the reaction mixture was stirred at room temperature for 12 hours. After the reaction, the mixture was diluted with ethyl acetate, and washed with aq. $NaHCO_3$ and brine. The collecting organic layer was dried over $MgSO_4$, concentrated *in vacuo*. The pure hydrazone **9** was obtained by preparatory TLC (40 mg, 67 %). ^{1}H NMR (400 MHz, $CDCl_3$) δ 10.94 (s, 1H), 9.14 (d, J = 2.6 Hz, 1H), 8.35 – 8.24 (m, 1H), 7.95 – 7.84 (m, 1H), 7.40 – 7.31 (m, 4H), 7.31 – 7.23 (m, 2H), 5.89 – 5.77 (m, 1H), 5.47 (dq, J = 15.5, 6.5 Hz, 1H), 2.71 – 2.54 (m, 2H), 2.42 (dt, J = 12.5, 6.2 Hz, 1H), 2.20 (ddd, J = 7.9, 7.1, 4.9 Hz, 1H), 2.04 – 1.94 (m, 1H), 1.87 (tdd, J = 8.2, 7.3, 4.0 Hz, 1H), 1.77 (dd, J = 6.5, 1.6 Hz, 3H); ^{13}C NMR (101 MHz, $CDCl_3$) δ 169.4, 145.4, 143.2, 138.0, 135.3, 130.2, 129.3, 128.4, 127.6, 126.9, 125.7, 123.6, 116.8, 57.8, 38.6, 28.4, 21.0, 18.3. The chemical structure was unambiguously determined by single crystal X-ray diffraction.

General Procedure for Cascade Isomerization-Hydroacylation Reactions

In a nitrogen-filled glove box, a 1-dram vial was charged with the indicated amount of $[(coe)_2RhCl]_2$, (*R*)-DTBM-MeOBIPHEP, internal standard (durene), and 1,2-dichloroethane. The solution was stirred at ambient temperature (30 ºC) for 30 minutes until homogeneous. Next, $AgBF_4$ was added and the resulting mixture was stirred for an additional 5 minutes prior to addition of the α,α-bis(allyl)aldehyde. The vial was then sealed with a Teflon-lined screw cap, and the reaction mixture was stirred for the indicated reaction time. Reaction progress and chemoselectivity were determined from analysis of the GC-FID chromatogram or ^{1}H NMR spectrum of the reaction mixture. The pure cyclopentanone was isolated either by column chromatography or preparative TLC.

For reactions of **2a**–**2g** and **2l**, 2.5 mol% $[(coe)_2RhCl]_2$, 5 mol% (*R*)-DTBM-MeOBIPHEP, 5 mol% $AgBF_4$, and 0.2 M DCE (1,2-chloromethane) were used, and the reactions were performed at 40 °C for 4 hours.
For reactions of **2h** and **2i**, 5 mol% $[(coe)_2RhCl]_2$, 10 mol% (*R*)-DTBM-MeOBIPHEP, 10 mol% $AgBF_4$, and 0.33 M DCE were used, and the reactions were performed at 30 ºC for 2 hours.
For reactions of **2j** and **2k**, 6 mol% $[(coe)_2RhCl]_2$, 12 mol% (*R*)-DTBM-MeOBIPHEP, 12 mol% $AgBF_4$, and 0.33 M DCE were used, and the reactions were performed at 30 ºC for 2 hours.

The stereochemistry of all α-vinylcyclopentanone **2** were assigned to be (*S*) by analogy of the result of **2a**.

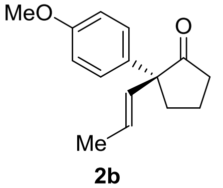

2b

(*S*)-(–)-2-(4-Methoxyphenyl)-2-(prop-1-en-1-yl)cyclopentan-1-one (2b)

The product **2b** was obtained by purification using preparative TLC (eluting with 20:1 hexanes/ethyl acetate) and isolated as a colorless oil (20.6 mg, 90%). ^{1}H NMR (400 MHz, $CDCl_3$) δ 7.25–7.20 (m, 2H), 6.89–6.83 (m, 2H), 5.58 (dq, J = 15.6, 1.4 Hz, 1H), 5.46 (dq, J = 15.6, 6.2 Hz, 1H), 3.82–3.76 (m, 3H), 2.49–2.22 (m, 4H), 2.03–1.81 (m, 2H), 1.72 (dd, J = 6.2, 1.4 Hz, 3H); ^{13}C NMR (101 MHz, $CDCl_3$) δ

218.6, 158.5, 133.4, 133.2, 128.5, 126.8, 113.9, 59.6, 55.4, 37.9, 36.7, 18.9, 18.3; IR (ATR): 3025, 2959, 1733, 1608, 1580, 1509, 1248, 1182, 1034, 828 cm^{-1}. HRMS (ESI-TOF) *m/z* calcd for $C_{15}H_{18}O_2Na$ $[M + Na]^+$: 253.1205, found: 253.1209. SFC analysis: 99% *ee*, 100 mm CHIRALCEL OD-H, 2% *i*PrOH, 2.5 mL/min, 220 nm, 44 °C, nozzle pressure = 200 bar CO_2, t_{R1} (major) = 2.77 min, t_{R2} (minor) = 3.20 min. $[\alpha]_D^{24}$ –5.6 (*c* 0.445, $CHCl_3$)

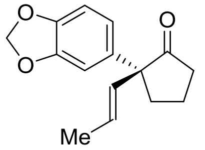

2c

(*S*)-(–)-2-(Benzo[d][1,3]dioxol-5-yl)-2-(prop-1-en-1-yl)cyclopentan-1-one (2c)

The product **2c** was obtained by purification using preparative TLC (eluting with 20:1 hexanes/ethyl acetate) and isolated as a colorless oil (22.3 mg, 91%). For the reaction that was performed on 1 mmol scale, purification was achieved using column chromatography (240.0 mg, 95%). ^{1}H NMR (400 MHz, $CDCl_3$) δ 6.83 (dd, J = 1.5, 0.8 Hz, 1H), 6.79–6.69 (m, 2H), 6.01–5.87 (m, 2H), 5.64–5.37 (m, 2H), 2.48–2.17 (m, 4H), 2.03–1.80 (m, 2H), 1.72 (dt, J = 5.0, 2.5 Hz, 3H); ^{13}C NMR (101 MHz, $CDCl_3$) δ 218.2, 147.9, 146.5, 135.4, 132.9, 127.0, 120.5, 108.3, 108.1, 101.2, 60.0, 37.9, 37.0, 18.9, 18.3; IR (ATR): 3024, 2960, 2915, 2883, 1733, 1610, 1503, 1485, 1434, 1239, 1037, 932, 812 cm^{-1}. HRMS (ESI-TOF) *m / z* calcd for $C_{15}H_{16}O_3Na$ $[M + Na]^+$: 267.0997, found: 267.1001. SFC analysis: 98% *ee*, 100 mm CHIRALCEL OD-H, 2% *i*PrOH, 2.5 mL/min, 220 nm, 44 °C, nozzle pressure = 200 bar CO_2, t_{R1} (major) = 3.01 min, t_{R2} (minor) = 3.54 min. $[\alpha]_D^{24}$ –13.4 (*c* 0.655, $CHCl_3$)

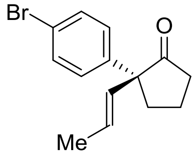

2d

(*S*)-(–)-2-(4-Bromophenyl)-2-(prop-1-en-1-yl)cyclopentan-1-one (2d)

The product **2d** was obtained by purification using preparative TLC (eluting with 20:1 hexanes/ethyl acetate) and isolated as a colorless oil (25.3 mg, 91%). ^{1}H NMR (400 MHz, $CDCl_3$) δ 7.51–7.40 (m, 2H), 7.24–7.14 (m, 2H), 5.60–5.42 (m, 2H), 2.49–2.23 (m, 4H), 2.05–1.83 (m, 2H), 1.73 (d, J = 4.7 Hz, 3H); ^{13}C NMR (101 MHz, $CDCl_3$) δ 217.7, 140.8, 132.3, 131.6, 129.3, 127.7, 121.0, 59.8, 37.9, 36.7, 18.9, 18.3; IR (ATR): 3025, 2961, 1735, 1586, 1488, 822 cm^{-1}. HRMS (ESI-TOF) *m / z* calcd for $C_{14}H_{15}BrONa$ $[M + Na]^+$: 301.0204, found: 301.0208. SFC analysis: 95% *ee*, 250 mm CHIRALCEL IC, 2% *i*PrOH, 2.5 mL/min, 220 nm, 44 °C, nozzle pressure = 200 bar CO_2, t_{R1} (major) = 7.84 min, t_{R2} (minor) = 7.48 min. $[\alpha]_D^{24}$ –15.0 (*c* 0.580, $CHCl_3$)

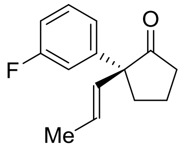

2e

(*S*)-(–)-2-(3-Fluorophenyl)-2-(prop-1-en-1-yl)cyclopentan-1-one (2e)

The product **2e** was obtained by purification using preparative TLC (eluting with 20:1 hexanes/ethyl acetate) and isolated as a colorless oil (18.1 mg, 83%). ^{1}H NMR (400 MHz, $CDCl_3$) δ 7.35–7.23 (m, 1H), 7.06 (dddd, J = 12.5, 10.8, 2.9, 1.4 Hz, 2H), 6.93 (tdd, J = 8.3, 2.6, 0.9 Hz, 1H), 5.58–5.45 (m, 2H), 2.48–2.25 (m, 4H), 2.03–1.86 (m, 2H), 1.74 (ddd, J = 5.9, 3.6, 1.1 Hz, 3H); ^{13}C NMR (101 MHz, $CDCl_3$) δ 217.6, 163.1 (d, J= 246.4 Hz), 144.5 (d, J= 7.1 Hz), 132.2, 130.0 (d, J= 9.1 Hz), 127.8, 123.1 (d, J= 2.8 Hz), 114.8 (d, J= 22.4 Hz), 113.8 (d, J= 21.2 Hz), 60.1, 38.1, 36.9, 19.0, 18.4; IR (ATR): 3027, 2963, 1737, 1612, 1586, 782, 695 cm^{-1}. HRMS (ESI-TOF) *m / z* calcd for $C_{14}H_{15}OFNa$ $[M + Na]^+$: 241.1005, found: 241.0996. SFC analysis: 99% *ee*, 250 mm CHIRALCEL AD-H, 10% *i*PrOH, 2.0 mL/min, 215 nm, 60 °C, nozzle pressure = 100 bar CO_2, t_{R1} (major) = 2.94 min, t_{R2} (minor) = 2.79 min, $[\alpha]_D^{24}$ –17.7 (*c* 0.265, $CHCl_3$).

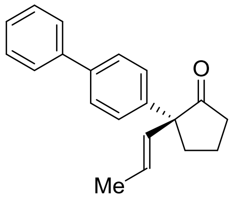

2f

(*S*)-(–)-2-([1,1'-Biphenyl]-4-yl)-2-(prop-1-en-1-yl)cyclopentan-1-one (2f)

The product **2f** was obtained by purification using preparative TLC (eluting with 20:1 hexanes/ethyl acetate) and isolated as a colorless oil (24.9 mg, 91%). ^{1}H NMR (400 MHz, $CDCl_3$) δ 7.64–7.52 (m, 4H), 7.48–7.29 (m, 5H), 5.70–5.47 (m, 2H), 2.42 (ttd, *J* = 16.3, 13.1, 7.2 Hz, 4H), 2.06–1.88 (m, 2H), 1.75 (dd, *J* = 6.0, 1.2 Hz, 3H); ^{13}C NMR (101 MHz, $CDCl_3$) δ 218.3, 140.9, 140.7, 139.8, 132.7, 128.9, 127.8, 127.4, 127.3, 127.2, 60.1, 38.1, 36.8, 19.0, 18.4; IR (ATR): 3028, 2960, 1735, 1600, 1486, 763, 730, 697 cm^{-1}; HRMS (ESI-TOF) *m* / *z* calcd for $C_{20}H_{20}ONa$ [M + Na]$^+$: 299.1412, found: 299.1403. SFC analysis: 99% *ee*, 100 mm CHIRALCEL AD-H, 3% *i*PrOH, 2.5 mL/min, 220 nm, 44 °C, nozzle pressure = 200 bar CO_2, t_{R1} (major) = 13.01 min, t_{R2} (minor) = 16.5 min. $[\alpha]_D^{24}$ –1.5 (*c* 0.980, $CHCl_3$)

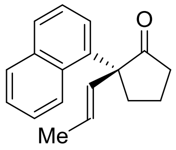

2g

(*S*)-(+)-2-(Naphthalen-1-yl)-2-(prop-1-en-1-yl)cyclopentan-1-one (2g)

The product **2g** was obtained by purification using preparative TLC (eluting with 20:1 hexanes/ethyl acetate) and isolated as a colorless oil (20.7 mg, 83%). ^{1}H NMR (400 MHz, $CDCl_3$) δ 7.99–7.81 (m, 2H), 7.77 (dd, *J* = 6.8, 2.4 Hz, 1H), 7.49–7.41 (m, 2H), 7.41–7.33 (m, 2H), 5.74 (ddd, *J* = 15.7, 2.8, 1.3 Hz, 1H), 5.61 (dq, *J* = 15.7, 6.3 Hz, 1H), 2.75 (dt, *J* = 14.2, 7.3 Hz, 1H), 2.69–2.42 (m, 3H), 2.05 (tdt, *J* = 14.4, 8.8, 7.2 Hz, 1H), 1.98–1.86 (m, 1H), 1.80 (dd, *J* = 6.3, 1.5 Hz, 3H); ^{13}C NMR (101 MHz, $CDCl_3$) δ 219.1, 138.5, 135.4, 132.7, 130.7, 129.6, 129.0, 128.5, 126.7, 126.5, 125.3, 125.3, 125.1, 61.4, 37.8, 37.6, 19.4, 18.6. IR (ATR): 3046, 2960, 1733, 1598, 775 cm^{-1}. HRMS (ESI-TOF) *m* / *z* calcd for $C_{18}H_{18}ONa$ [M + Na]$^+$: 273.1255, found: 273.1248. SFC analysis: 99% *ee*, 100 mm CHIRALCEL OD-H, 2% *i*PrOH, 2.5 mL/min, 220 nm, 44 °C, nozzle pressure = 200 bar CO_2, t_{R1} (major) = 6.47 min, t_{R2} (minor) = 7.80 min. $[\alpha]_D^{24}$ +92.2 (*c* 0.825, $CHCl_3$).

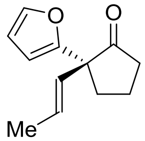

2h

(*S*)-(+)-2-(Furan-2-yl)-2-(prop-1-en-1-yl)cyclopentan-1-one (2h)

The product **2h** was obtained by purification using preparative TLC (eluting with 20:1 hexanes/ethyl acetate) and isolated as a colorless oil (16.6 mg, 85%). ^{1}H NMR (400 MHz, $CDCl_3$) δ 7.37 (dd, *J* = 1.9, 0.9 Hz, 1H), 6.31 (dd, *J* = 3.2, 1.9 Hz, 1H), 6.15 (dd, *J* = 3.2, 0.9 Hz, 1H), 5.62–5.49 (m, 2H), 2.55 (dt, *J* = 13.0, 7.3 Hz, 1H), 2.37 (t, *J* = 7.8 Hz, 2H), 2.28–2.16 (m, 1H), 1.95 (tt, *J* = 8.0, 3.9 Hz, 2H), 1.80–1.67 (m, 3H); ^{13}C NMR (101 MHz, $CDCl_3$) δ 215.6, 154.2, 142.4, 129.6, 128.2, 110.3, 107.4, 56.9, 37.4, 35.0, 19.2, 18.3; IR (ATR): 3119, 2966, 1732, 1652, 1140, 969 cm^{-1}. HRMS (ESI-TOF) *m* / *z* calcd for $C_{12}H_{14}O_2Na$ [M + Na]$^+$: 213.0892, found: 213.0887; SFC analysis: >95% *ee*, 250 mm Whelk-O (*R*,*R*), 1% *i*PrOH, 2 mL/min, 215 nm, 44 °C, nozzle pressure = 100 bar CO_2, t_{R1} (major) = 7.8 min, t_{R2} (minor) = 7.5 min. $[\alpha]_D^{24}$ +3.1 (*c* 0.425, $CHCl_3$)

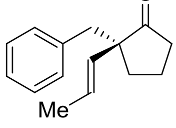

2i

(*S*)-(–)-2-Benzyl-2-(prop-1-en-1-yl)cyclopentan-1-one (2i)

The product **2i** was obtained by purification using preparative TLC (eluting with 20:1 hexanes/ethyl acetate) and isolated as a colorless oil (16.2 mg, 76%). ^{1}H NMR (400 MHz, $CDCl_3$) δ 7.27–7.17 (m, 3H), 7.12–7.06 (m, 2H), 5.43 (dq, *J* = 15.7, 6.1 Hz, 1H), 5.37–5.29 (m, 1H), 2.90 (d, *J* = 13.4 Hz, 1H), 2.75 (d, *J* = 13.4 Hz, 1H), 2.36–2.24 (m, 1H), 2.03 (dt, *J* = 19.3, 9.0 Hz, 1H), 1.87 (m, 2H), 1.79 (m, 2H), 1.69 (dd, *J* = 6.1, 1.3 Hz, 3H). ^{13}C NMR (101 MHz, $CDCl_3$) δ 220.1, 137.9, 132.3, 130.5, 128.1, 126.6, 126.4, 56.5, 42.6, 38.0, 32.9, 18.7, 18.3; IR (ATR): 3027, 2960,

1733, 1603, 701 cm^{-1}; HRMS (ESI-TOF) *m* / *z* calcd for $C_{15}H_{18}ONa$ $[M + Na]^+$: 237.1255, found: 237.1250. The product could not be separated by chiral SFC analysis. In order to obtain a racemic assay, the olefin was hydrogenated to produce material that can be separated by chiral SFC. The enantioselectivity was deduced by analyzing the *ee* of the hydrogenated product. SFC analysis: 91% *ee*, 250 mm CHIRALCEL IC, 2% *i*PrOH, 2.5 mL/min, 220 nm, 44 °C, nozzle pressure = 200 bar CO_2, t_{R1} (major) = 7.07 min, t_{R2} (minor) = 5.84 min. $[\alpha]_D^{24}$ –41.8 (*c* 0.280, $CHCl_3$)

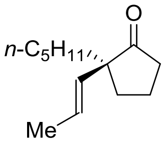

2j

(*S*)-(–)-2-Pentyl-2-(prop-1-en-1-yl)cyclopentan-1-one (2j)

The product **2j** was obtained by purification using preparative TLC (eluting with 20:1 hexanes/ethyl acetate) and isolated as a colorless oil (14.2 mg, 73%). ^{1}H NMR (400 MHz, $CDCl_3$) δ 5.47 (dq, *J* = 15.7, 6.3 Hz, 1H), 5.31 (dq, *J* = 15.7, 1.5 Hz, 1H), 2.36–2.24 (m, 1H), 2.22–2.10 (m, 1H), 2.07–1.98 (m, 1H), 1.92–1.78 (m, 3H), 1.73–1.65 (m, 3H), 1.53 (dd, *J* = 12.5, 3.3 Hz, 1H), 1.40−1.20 (m, 6H), 1.18–1.06 (m, 1H), 0.87 (dd, *J* = 9.1, 5.0 Hz, 3H); ^{13}C NMR (101 MHz, $CDCl_3$) δ 221.0, 132.3, 125.9, 55.4, 37.8, 36.7, 33.6, 32.5, 24.2, 22.7, 19.0, 18.4, 14.2; IR (ATR): 3024, 2957, 2930, 2858, 1735, 1466, 1452, 1152, 974 cm^{-1}; HRMS (ESI-TOF) *m* / *z* calcd for $C_{13}H_{22}ONa$ $[M + Na]^+$: 217.1568, found: 217.1559. SFC analysis: >95% *ee*, 250 mm Whelk-O (*R*,*R*), 8% *i*PrOH, 2 mL/min, 215 nm, 44 °C, nozzle pressure = 100 bar CO_2, t_{R1} (major) = 2.57 min, t_{R2} (minor) = 2.68 min. $[\alpha]_D^{24}$ –50.9 (*c* 0.230, $CHCl_3$).

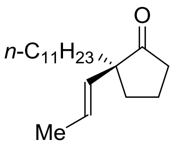

2k

(*S*)-(–)-2-Undecyl-2-(prop-1-en-1-yl)cyclopentan-1-one (2k)

The product **2k** was obtained by purification using preparative TLC (eluting with 20:1 hexanes/ethyl acetate) and isolated as a colorless oil (22.7 mg, 82%). ^{1}H NMR (400 MHz, $CDCl_3$) δ 5.47 (dq, *J* = 15.7, 6.3 Hz, 1H), 5.38–5.25 (m, 1H), 2.36–2.24 (m, 1H), 2.22–2.11 (m, 1H), 2.06–1.99 (m, 1H), 1.93–1.77 (m, 3H), 1.69 (dd, *J* = 6.3, 1.5 Hz, 3H), 1.53 (dd, *J* = 12.8, 3.1 Hz, 1H), 1.46–1.18 (m, 18H), 1.12 (dd, *J* = 12.8, 4.2 Hz, 1H), 0.89 (t, *J* = 6.9 Hz, 3H). ^{13}C NMR (101 MHz, $CDCl_3$) δ 220.9, 132.2, 125.8, 55.3, 37.7, 36.7, 33.6, 32.1, 30.3, 29.8, 29.8, 29.7, 29.5, 24.5, 22.8, 18.9, 18.4, 14.3; IR (ATR) 3024, 2922, 2853, 1737, 1466, 1456, 1152, 974 cm^{-1}; HRMS (ESI-TOF) *m* / *z* calcd for $C_{19}H_{34}ONa$ $[M + Na]^+$: 301.2507, found: 301.2516. SFC analysis: 99% *ee*, 250 mm Whelk-O (*R*,*R*), 8% *i*PrOH, 2.0 mL/min, 215 nm, 44 °C, nozzle pressure = 100 bar CO_2, t_{R1} (major) = 3.74 min, t_{R2} (minor) = 3.93 min. $[\alpha]_D^{24}$ –41.0 (*c* 0.705, $CHCl_3$).

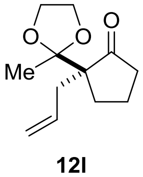

12l

(–)-2-Allyl-2-(2-methyl-1,3-dioxolan-2-yl)cyclopentan-1-one (12l)

The product **12l** was obtained by purification using preparative TLC (eluting with 20:1 hexanes/ethyl acetate) and isolated as a colorless oil (18.7 mg, 89%). ^{1}H NMR (400 MHz, $CDCl_3$) δ 5.70 (dddd, *J* = 16.9, 10.0, 8.0, 6.8 Hz, 1H), 5.17–4.94 (m, 2H), 4.07–3.81 (m, 4H), 2.51 (ddt, *J* = 13.6, 6.8, 1.3 Hz, 1H), 2.40–2.09 (m, 4H), 2.01–1.85 (m, 2H), 1.77–1.64 (m, 1H), 1.35 (s, 3H); ^{13}C NMR (101 MHz, $CDCl_3$) δ 220.1, 134.4, 118.3, 65.1, 64.8, 58.5, 40.3, 38.2, 29.9, 20.3, 19.1; IR (ATR): 3076, 2964, 2885, 1733, 1640, 1157, 1038 cm^{-1}; HRMS (ESI-TOF) *m* / *z* calcd for $C_{12}H_{18}O_3Na$ $[M + Na]^+$: 233.1154, found: 233.1160. $[\alpha]_D^{24}$ –27.7 (*c* 0.285, $CHCl_3$).

D-labeling experiment (Scheme 2a)

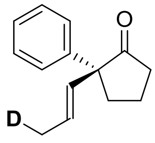

d-**2a**

The product *d*-**2a** was obtained by purification using preparative TLC (eluting with 20:1 hexanes/ethyl acetate) and isolated as a colorless oil (18.3 mg, 91%). ^{1}H NMR (400 MHz, $CDCl_3$) δ 7.36–7.28 (m, 4H), 7.26–7.21 (m, 1H), 5.61 (dt, *J* = 15.7, 1.3 Hz, 1H), 5.56–5.43 (m, 1H), 2.51–2.27 (m, 4H), 2.05–1.83 (m, 2H), 1.77–1.67 (m, 2H); ^{2}H NMR (61.4 MHz, $CDCl_3$) δ 1.75 (q, *J* = 1.3 Hz, 1D); ^{13}C NMR (101 MHz, $CDCl_3$) δ 218.4, 141.6, 132.8, 128.5, 127.4, 127.0, 126.9, 60.3, 38.1, 36.8, 19.0, 18.0 (t, *J* = 19.4 Hz); IR (ATR) 3026, 2960, 1735, 1598, 756, 697 cm^{-1}; HRMS (ESI-TOF) *m* / *z* calcd for $C_{14}H_{15}ODNa$ $[M + Na]^+$: 224.1162, found: 224.1162, $[\alpha]_D^{24}$ –11.6 (*c* 0.760, $CHCl_3$).

***rac*-2-Allyl-2-phenylcyclopentanone (12a)**

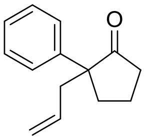

12a

The product **13a** was observed by Rh (10 mol%) / dppf (10 mol%) catalysis (DCE, 40 °C, 18 h, 50 %). The pure **13a** was obtained by purification using preparative TLC (eluting with 20:1 hexanes/ethyl acetate) and isolated as a colorless oil (16.0 mg, 40%). The ^{1}H and ^{13}C NMR spectra matched the literature reported values.[2] ^{1}H NMR (400 MHz, $CDCl_3$) δ 7.45–7.38 (m, 2H), 7.38–7.30 (m, 2H), 7.27–7.22 (m, 1H), 5.58–5.44 (m, 1H), 5.04–4.94 (m, 2H), 2.65–2.51 (m, 2H), 2.44 (dd, *J* = 13.9, 7.5 Hz, 1H), 2.39–2.19 (m, 2H), 2.10 (ddd, *J* = 13.4, 9.9, 6.7 Hz, 1H), 1.94 (dddd, *J* = 11.2, 9.2, 6.9, 3.3 Hz, 1H), 1.88–1.78 (m, 1H); ^{13}C NMR (101 MHz, $CDCl_3$) δ 220.1, 137.9, 132.3, 130.5, 128.1, 126.6, 126.4, 56.5, 42.6, 38.0, 32.9, 18.7, 18.3; IR (ATR) 3026, 2960, 1735, 1598, 756, 697 cm^{-1}.

3 Preparation of substrates

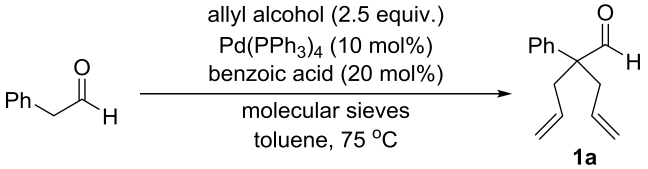

One-Step Synthesis of 2-Allyl-2-phenylpent-4-enal (**1a**): Aldehyde **1a** was prepared in one-step according to a procedure in literature by List.[3] A 250 mL round bottom flask was charged with toluene (60 mL), 4A molecular sieve (6 g), benzoic acid (20 mol%), $Pd(PPh_3)_4$ (835 mg, 0.722 mmol, 10 mol%), allyl alcohol (4.2 ml, 61.8 mmol, 2.5 equiv), then phenylacetaldehyde (2.7 mL, 24.2 mmol) was added. The reaction mixture was stirred at 75 °C for 23 hours. The reaction mixture was cooled to rt and filtered through filter paper to remove molecular sieves. The solution was concentrated *in vacuo*. Purification by flash column chromatography gave pure **1a** (2.71 g, 56% isolated yield). The ^{1}H NMR spectrum matched the literature reported values.[3] ^{1}H NMR (400 MHz, $CDCl_3$) δ 9.55 (s, 1H), 7.44–7.36 (m, 2H), 7.34–7.28 (m, 1H), 7.25–7.20 (m, 2H), 5.57 (ddt, *J* = 17.4, 10.2, 7.3 Hz, 2H), 5.16–5.03 (m, 4H), 2.73 (dd, *J* = 7.3, 1.1 Hz, 4H).

---

[2] F. Nahra, Y. Mace, A. Boreux, F. Billard, O. Riant, *Chem. Eur. J.* 2014, **20**, 10970.
[3] G. Jiang, B. List, *Adv. Synth. Catal.* 2011, **353**, 1667.

General Procedure for Synthesis of α,α-Bisallylaldehyde 1

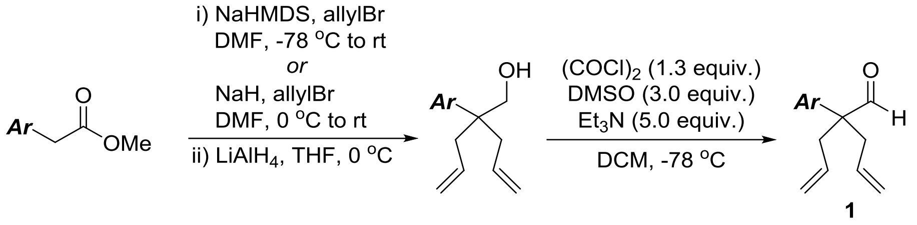

- Representative examples for synthesis of **1b-OH** – **1h-OH**

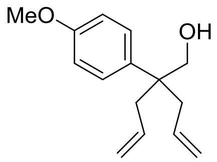

1b-OH

2-Allyl-2-(4-methoxyphenyl)pent-4-en-1-ol (**1b-OH**)

Sodium bis(trimethylsilyl)amide (NaHMDS) solution (6.5 mL, 2.0 M solution in THF, 12.5 mmol, 2.5 equiv) was added to THF solution of methyl 4-methoxyphenylacetate (900 mg, 5 mmol, 1 equiv) in an acetone/dry ice bath at –78 °C. The solution was stirred for 30 minutes. Then, allyl bromide (1.1 mL, 12.5 mmol, 2.5 equiv) was added dropwise to the reaction mixture. The solution was warmed to room temperature and stirred for 4 hours. The reaction mixture was quenched with aqueous NH_4Cl solution and aqueous 2 M HCl solution, and the aqueous layer extracted with ethyl acetate 3 times. The organic layers were combined and dried over $MgSO_4$, filtered, and concentrated. The resulting α,α-bisallyl ester was used without further purification. $LiAlH_4$ (473 mg, 12.5 mmol, 2.5 equiv) was added slowly to a stirring solution of ester (1.3 g, 5 mmol, 1 equiv) in 25 mL THF at 0 °C. (This reaction mixture was cooled using an ice bath). After addition of $LiAlH_4$, the ice bath was removed and the reaction mixture was allowed to stir at room temperature for 4 hours. The reaction mixture was quenched using the Fieser method and the resulting solution was dried with $MgSO_4$, filtered, and concentrated. The pure alcohol **1b-OH** was obtained after column chromatography (960 mg, 83% over 2 steps) as a colorless oil. The ^{1}H and ^{13}C NMR spectra matched the literature reported values.[1] ^{1}H NMR (400 MHz, $CDCl_3$) δ 7.28 (d, J = 8.9 Hz, 2H), 6.91 (d, J = 8.9 Hz, 2H), 5.66 (ddt, J = 17.3, 10.1, 7.2 Hz, 2H), 5.08 (dddd, J = 17.1, 10.1, 2.3, 1.2 Hz, 4H), 3.82 (s, 3H), 3.77 (s, 2H), 2.49 (qd, J = 14.0, 7.2 Hz, 4H); ^{13}C NMR (101 MHz, $CDCl_3$) δ 158.0, 135.4, 134.6, 128.0, 117.9, 113.9, 68.1, 55.3, 45.4, 39.7.

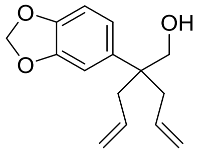

1c-OH

2-Allyl-2-(benzo[d][1,3]dioxol-5-yl)pent-4-en-1-ol (**1c-OH**)

For bisallylation, NaH was used as a base instead of NaHMDS. The methyl ester (1.0 g, 4.9 mmol, 1 equiv) was added to a DMF (20 mL, 0.25 M) solution of NaH (60%, 500 mg, 12.5 mmol, 2.5 equiv), and the mixture was stirred at 0 °C using an ice bath. Allyl bromide (1.1 mL, 12.5 mmol, 2.5 equiv) was added to the reaction mixture and stirred for 4 hours at room temperature. The reaction mixture was quenched with saturated aqueous NH_4Cl solution, diluted with ethyl acetate, and washed with H_2O three times. The organic layer was dried over $MgSO_4$, filtered, and concentrated under reduced procedure. For reduction, the resulting α,α-bisallyl ester (1.15 g, 4.2 mmol) was treated with $LiAlH_4$ (578 mg, 15 mmol, 3.6 equiv) in Et_2O (20 mL, 0.20 M). The pure alcohol was obtained as a colorless liquid. The pure alcohol **1c-OH** was obtained as a colorless liquid (620 mg, 61%) after column chromatography. ^{1}H NMR (400 MHz, $CDCl_3$) δ 6.88 (t, J = 1.2 Hz, 1H), 6.85–6.75 (m, 2H), 5.95 (d, J = 3.7 Hz, 2H), 5.65 (ddt, J = 17.3, 10.1, 7.2 Hz, 2H), 5.08 (ddtd, J = 14.3, 10.1, 2.1, 1.2 Hz, 4H), 3.74 (s, 2H), 2.56–2.34 (m, 4H); ^{13}C NMR (101 MHz, $CDCl_3$) δ 148.1,

146.0, 137.5, 120.1, 118.1, 108.2, 107.6, 101.1, 68.1, 45.9, 39.9; IR (ATR) 3412 (br), 3074, 2889, 1638, 1504, 1489, 1434, 1230, 1037, 913, 898, 808 cm^{-1}.

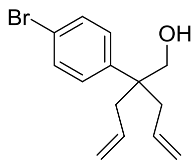

1d-OH

2-Allyl-2-(4-bromophenyl)pent-4-en-1-ol (1d-OH)

For bisallylation, methyl 4-bromophenylacetate (1.1 g, 4.8 mmol), NaH (60%, 460 mg, 12 mmol), allyl bromide (1.1 mL, 12 mmol) and DMF (20 mL) were used. For reduction, ester (930 mg, 3 mmol), $LiAlH_4$ (227 mg, 6 mmol, 2.0 equiv) and THF (15 mL, 0.20 M) were used. The pure alcohol **1d-OH** was obtained after column chromatography as a colorless oil (640 mg, 50% over 2 steps). ^{1}H NMR (400 MHz, $CDCl_3$) δ 7.51–7.45 (m, 1H), 7.26–7.20 (m, 1H), 5.73–5.50 (m, 1H), 5.17–4.97 (m, 2H), 3.79 (s, 1H), 2.59–2.40 (m, 2H); ^{13}C NMR (101 MHz, $CDCl_3$) δ 142.8, 134.1, 131.6, 128.9, 120.4, 118.4; IR (ATR) 3405 (br), 3076, 2979, 2932, 1639, 1048, 1012, 996, 913, 734 cm^{-1}; LRMS (EI) *m* / *z* calcd for $[C_{14}H_{13}Br - H_2O]^+$: 262.1, found: 262.1.

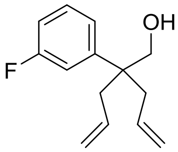

1e-OH

2-Allyl-2-(3-fluorophenyl)pent-4-en-1-ol (1e-OH)

For bisallylation, methyl 3-fluorophenylacetate (1.29 g, 7.67 mmol, 1 equiv), NaHMDS (2.0 M solution, 9.6 mg, 19.18 mmol), allyl bromide (1.1 mL, 12.5 mmol) and DMF (20 mL) were used. For reduction, $LiAlH_4$ (726 mg, 19.18 mmol, 2.5 equiv) and THF (30 mL, 0.20 M) were used. The pure alcohol **1e-OH** was obtained after column chromatography as a colorless oil (1.28 mg, 76%). ^{1}H NMR (400 MHz, $CDCl_3$) δ 7.37–7.29 (m, 1H), 7.14 (ddd, *J* = 7.9, 1.8, 0.9 Hz, 1H), 7.10–7.04 (m, 1H), 6.94 (tdd, *J* = 8.3, 2.5, 0.9 Hz, 1H), 5.63 (ddt, *J* = 17.3, 10.1, 7.3 Hz, 2H), 5.08 (ddtd, *J* = 14.3, 10.1, 2.1, 1.2 Hz, 4H), 3.80 (s, 2H), 2.61–2.35 (m, 4H).; ^{13}C NMR (101 MHz, $CDCl_3$) δ 163.3 (d, *J*= 242.4 Hz), 146.8 (d, *J*= 6.1 Hz), 134.1, 130.0 (d, *J*= 8.1 Hz), 122.7 (d, *J*= 3.0 Hz), 118.5, 114.4 (d, *J*= 22.2 Hz), 113.4 (d, *J*= 21.2 Hz), 67.8, 46.2, 39.9; IR (ATR) 3408 (br), 3076, 2979, 2929, 1639, 1614, 1586, 1437, 1226, 1047, 997, 914, 892, 783, 706 cm^{-1}; LRMS (EI) *m* / *z* calcd for $[C_{14}H_{17}OF]^+$: 220.1, found: 219.9.

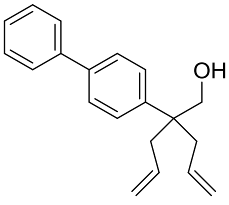

1f-OH

2-([1,1'-Biphenyl]-4-yl)-2-allylpent-4-en-1-ol (1f-OH)

For bisallylation, methyl 2-([1,1'-biphenyl]-4-yl)acetate (1.0 g, 4.4 mmol, 1 equiv), NaHMDS (2.0 M solution, 5.5 mL, 11 mmol), allyl bromide (0.95 mL, 11 mmol) and DMF (20 mL) were used. For reduction, ester (1.2 g, 3.9 mmol, 1 equiv), $LiAlH_4$ (369 mg, 9.75 mmol, 2.5 equiv) and THF (20 mL, 0.19 M) were used. The pure alcohol **1f-OH** was obtained after column chromatography as a colorless oil (952 mg, 87% over 2 steps). ^{1}H NMR (500 MHz, $CDCl_3$) δ 7.62-7.58 (m, 4H), 7.46-7.42 (m, 4H), 7.36-7.33 (m, 1H), 5.69 (ddt, *J* = 17.2, 10.0, 7.2 Hz, 2H), 5.15-5.11 (m, 2H), 5.08-5.05 (m, 2H), 3.84 (s, 2H), 2.61-2.49 (m, 4H), 1.43-1.43 (m, 2H); ^{13}C NMR (126 MHz, $CDCl_3$) δ 142.7, 140.8, 139.2, 134.5, 128.9, 127.43, 127.35, 127.23, 127.11, 118.1, 68.1, 45.9, 39.8; IR (ATR) 3409 (br), 3074, 2977, 2925, 1638, 1045, 1006, 997, 912, 834, 766, 735, 696 cm^{-1}; LRMS (EI) *m* / *z* calcd for $[C_{20}H_{22}O]^+$: 278.2, found: 278.1.

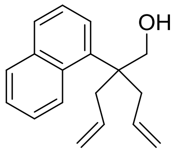

1g-OH

2-Allyl-2-(naphthalen-1-yl)pent-4-en-1-ol (1g-OH)

For bisallylation, methyl 2-(naphthalen-1-yl)acetate (1.56 g, 7.8 mmol), NaH (60%, 780 mg, 19.5 mmol), allyl bromide (1.7 mL, 19.5 mmol) and DMF (20 mL) were used. For reduction, $LiAlH_4$ (738 mg, 19.5 mmol, 2.5 equiv) and THF (30 mL, 0.20 M) were used. The pure alcohol **1g-OH** was obtained after column chromatography as a colorless oil (510 mg, 26% over steps). ^{1}H NMR (400 MHz, $CDCl_3$) δ 8.46 (d, *J* = 8.0 Hz, 1H), 7.90 (dd, *J* = 7.8, 1.8 Hz, 1H), 7.78 (dd, *J* = 5.4, 3.8 Hz, 1H), 7.60 – 7.37 (m, 4H), 5.55 (ddt, *J* = 17.4, 10.0, 7.2 Hz, 2H), 5.01 (ddd, *J* = 13.6, 11.2, 1.2 Hz, 4H), 4.18 (s, 2H), 2.95 (ddd, *J* = 22.1, 14.3, 7.2 Hz, 4H); ^{13}C NMR (101 MHz, $CDCl_3$) δ 138.6, 135.3, 135.1, 132.0, 130.2, 128.5, 126.7, 125.7, 125.4, 125.2, 125.1, 117.7, 67.3, 47.6, 40.1.

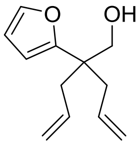

1h-OH

2-Allyl-2-(furan-2-yl)pent-4-en-1-ol (1h-OH)

For bisallylation, methyl (2-furyl)acetate (440 mg, 3 mmol, 1 equiv), NaH (400 mg, 10.4 mmol), allyl bromide (1.1 mL, 10.4 mmol) and DMF (15 mL) were used. For reduction, the resulting ester (620 mg, 2.8 mmol), $LiAlH_4$ (287 mg, 7.58 mmol, 2.7 equiv) and Et_2O (15 mL, 0.20 M) were used. The pure alcohol **1h-OH** was obtained after column chromatography as a pale-yellow oil (471.5 mg, 87%). ^{1}H NMR (500 MHz, $CDCl_3$) δ 7.37 (t, *J* = 0.9 Hz, 1H), 6.31 (dd, *J* = 3.2, 1.9 Hz, 1H), 6.11 (dd, *J* = 3.2, 0.7 Hz, 1H), 5.72-5.63 (m, 2H), 5.13-5.03 (m, 4H), 3.70-3.69 (m, 2H), 2.46-2.40 (m, 4H), 1.59-1.52 (m, 1H); ^{13}C NMR (126 MHz, $CDCl_3$) δ 157.8, 141.5, 133.9, 118.2, 110.1, 106.6, 66.6, 44.8, 37.9; IR (ATR) 3405, 3076, 2979, 2932, 1639, 1048, 1012, 996, 913, 734 cm^{-1}.

- Representative procedure for synthesis of **1i-OH–1k-OH**

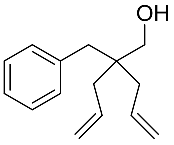

1i-OH

2-Allyl-2-benzylpent-4-en-1-ol (1i-OH)

For bisallylation, NaHMDS (2.0 M solution in THF, 7.3 mL, 14.64 mmol) was added dropwise to DMF (30 mL) solution in methyl hydrocinnamate (2.0 mg, 12.2 mmol) at –78 ^{o}C by using a flask cooled in an acetone/dry ice bath. The reaction mixture was stirred for 30 minutes. Allyl bromide (1.1 mL, 10.4 mmol) was added to the mixture and the reaction was stirred for 4 hours. The reaction mixture was quenched with aqueous NH_4Cl solution and ethyl acetate was added to the resulting mixture. The organic layer was separated and washed with H_2O three times, dried over $MgSO_4$, and concentrated *in vacuo*. The resulting monoallylated ester was used without further purification. The material isolated in this manner was directed subjected to NaHMDS (2.0 M solution in THF, 7.3 ml, 14.64 mmol), allyl bromide (1.1 mL, 10.4 mmol) and DMF (30 mL) to afford α,α-bisallyl ester (1.60 g, 53%). For reduction, the resulting α,α-bisallyl ester (1.4 g, 5.7 mmol), $LiAlH_4$ (539 mg, 14.2 mmol, 2.5 equiv) and Et_2O (25 mL, 0.21 M) were used. The pure alcohol **1i-OH** was obtained after column chromatography as a colorless oil (1.1 mg, 88%). The ^{1}H and ^{13}C NMR spectra matched the literature reported values.[1] ^{1}H NMR (400 MHz, $CDCl_3$) δ 7.37–7.13 (m, 4H), 6.03–5.87 (m, 1H), 5.19–5.09 (m, 2H), 3.39 (s, 1H), 2.67 (s, 1H), 2.08 (dt, J = 7.4, 1.1 Hz, 2H); ^{13}C NMR (101 MHz, $CDCl_3$) δ 138.4, 134.9, 130.8, 128.3, 126.4, 118.2, 67.1, 42.3, 40.7, 38.9.

2,2-Diallylheptan-1-ol (1j-OH)

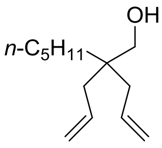

1j-OH

Following the representative procedure for **1i-OH**. For bisallylation, methyl heptanoate (2.0 g, 12.5 mmol), NaHMDS (2.0 M solution in THF, 8.3 mL, 16.6 mmol), allyl bromide (1.4 mL, 16.6 mmol) and DMF (30 mL) were used. THF (30 mL) was used as the solvent in the second allylation. The α,α-bisallyl ester (1.55 g, 50 %) was used for the next reaction without further purification. For reduction, the α,α-bisallyl ester (1.5 g, 6.7 mmol), $LiAlH_4$ (630 mg, 16.74 mmol, 2.5 equiv) and THF (30 mL, 0.23 M) were used. The pure alcohol **1h-OH** was obtained as a colorless liquid (880 mg, 67%) after column chromatography. ^{1}H NMR (400 MHz, $CDCl_3$) δ 6.00–5.71 (m, 2H), 5.22–4.96 (m, 4H), 3.40 (s, 2H), 2.13–1.97 (m, 4H), 1.43–1.15 (m, 8H), 0.90 (t, *J* = 7.0 Hz, 3H); ^{13}C NMR (101 MHz, $CDCl_3$) δ 135.1, 117.6, 67.7, 41.0, 39.1, 33.8, 32.8, 22.8, 22.7, 14.2; IR (ATR): 3359 (br), 3075, 2955, 2929, 2860, 1638, 1443, 1045, 995, 911 cm^{-1}; LRMS (EI) *m* / *z* calcd for $[C_{13}H_{24}O]^+$: 196.2, found: 196.0.

2,2-Diallyltridecan-1-ol (1k-OH)

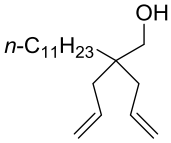

1k-OH

For bisallylation, methyl tridecanoate (5.0 g, 21.9 mmol), LDA (*n*-BuLi (1.5 M solution in hexanes, 32.1 mL, 48.2 mmol) + diisopropylamine (6.75 mL, 48.2 mmol)), allyl bromide (4.6 mL, 52.5 mmol) and THF (100 mL) were used. For reduction, $LiAlH_4$ (3.32 g, 87.6 mmol, 4.0 equiv) and THF (100 mL, 0.22 M) were used. The pure alcohol **1k-OH** was obtained after column chromatography as a colorless liquid (2.96 g, 49%). ^{1}H NMR (400 MHz, $CDCl_3$) δ 5.97–5.75 (m, 2H), 5.17–5.00 (m, 4H), 3.40 (s, 2H), 2.05 (ddt, *J* = 7.5, 2.1, 1.2 Hz, 4H), 1.29 (d, *J* = 16.6 Hz, 20H), 0.89 (t, *J* = 6.9 Hz, 3H); ^{13}C NMR (101 MHz, $CDCl_3$) δ 135.1, 117.6, 67.7, 41.0, 39.2, 33.9, 32.1, 30.6, 29.8, 29.8, 29.5, 23.0, 22.8, 14.3; IR (ATR): 3372 (br), 3075, 2922, 2853, 1638, 1466, 911 cm^{-1}; LRMS (EI) *m* / *z* calcd for $[C_{19}H_{36}O]^+$: 280.3, found: 280.3.

- Representative procedure for synthesis of aldehyde **1** from oxidation of **1-OH**

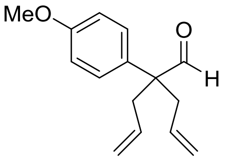

1b

DMSO (873 μL, 12.3 mmol, 3 equiv) was added dropwise to a DCM solution of oxalyl chloride (457 μL, 5.3 mmol, 1.3 equiv) at –78 °C in an acetone/dry ice bath, and then stirred for 30 minutes. A solution of alcohol **1b-OH** (960 mg, 4.1 mmol, 1 equiv) in DCM was added dropwise at –78 °C and stirred for 30 minutes. Triethylamine (2.9 mL, 20.5 mmol, 5 equiv) was added dropwise at –78 °C. The reaction mixture was then warmed to room temperature and stirred for an additional 30 minutes. The reaction was quenched with water and extracted with DCM. The organic layer was washed with water, dried with anhydrous $MgSO_4$, filtered, and concentrated *in vacuo*. The crude residue was purified by silica gel column chromatography to afford 2-allyl-2-(4-methoxyphenyl)pent-4-enal (756.3 mg, 80%) as a colorless liquid. The ^{1}H and ^{13}C NMR spectra matched the literature reported values.[1] ^{1}H NMR (400 MHz, $CDCl_3$) δ 9.49 (s, 1H), 7.14 (d, *J* = 9.0 Hz, 2H), 6.98–6.85 (m, 2H), 5.56 (ddt, *J* = 17.4, 10.2, 7.3 Hz, 2H), 5.18–4.97 (m, 4H), 3.82 (s, 3H), 2.69 (dd, *J* = 7.2, 0.9 Hz, 4H); ^{13}C NMR (126 MHz, $CDCl_3$) δ 201.9, 159.0, 132.9, 129.8, 128.9, 119.0, 114.4, 56.3, 55.4, 36.8.

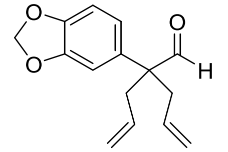

1c

2-Allyl-2-(benzo[d][1,3]dioxol-5-yl)pent-4-enal (**1c**)

Alcohol **1c-OH** (600 mg, 2.44 mmol), oxalyl chloride (270 µL), DMSO (520 µL), triethylamine (1.7 ml) and DCM (15 mL) were used. The pure aldehyde **1c** was obtained after column chromatography as a colorless liquid (569.5 mg, 96%). ^{1}H NMR (400 MHz, $CDCl_3$) δ 9.47 (s, 1H), 6.83 (d, *J* = 8.1 Hz, 1H), 6.73 (d, *J* = 1.9 Hz, 1H), 6.67 (dd, *J* = 8.1, 1.9 Hz, 1H), 5.98 (s, 2H), 5.56 (ddt, *J* = 17.6, 10.3, 7.2 Hz, 2H), 5.13–5.04 (m, 4H), 2.66 (dd, *J* = 6.9, 1.0 Hz, 4H). ^{13}C NMR (101 MHz, $CDCl_3$) δ 201.5, 148.5, 147.0, 132.8, 131.8, 121.2, 119.1, 108.6, 108.1, 101.4, 56.6, 36.9. IR (ATR): 3077, 2979, 2902, 2802, 2709, 1721, 1640, 1610, 1504, 1488, 1243, 1038, 917, 808, 653 cm^{-1}; HRMS (ESI-TOF) *m* / *z* calcd for $C_{15}H_{16}O_3Na$ $[M + Na]^+$: 267.0997, found: 267.0995.

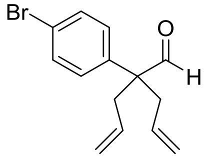

1d

2-Allyl-2-(4-bromophenyl)pent-4-enal (**1d**)

Alcohol **1d-OH** (562 mg, 2.0 mmol), oxalyl chloride (224 µL), DMSO (430 µL), triethylamine (1.4 mL) and DCM (15 mL) were used. The pure aldehyde **1d** was obtained after column chromatography as a colorless liquid (380.2 mg, 68%). ^{1}H NMR (400 MHz, $CDCl_3$) δ 9.52 (s, 1H), 7.62–7.47 (m, 2H), 7.16–6.95 (m, 2H), 5.54 (ddt, *J* = 20.4, 9.7, 7.3 Hz, 2H), 5.18–5.01 (m, 4H), 2.77–2.61 (m, 4H); ^{13}C NMR (100 MHz, $CDCl_3$) δ 201.4, 137.3, 132.3, 132.1, 129.5, 121.9, 119.5, 56.7, 37.0; IR (ATR): 3077, 29789, 2917, 2805, 2712, 1723, 1640, 1492, 1008, 996, 917, 817 cm^{-1}; HRMS (CI-TOF) *m* / *z* calcd for $C_{14}H_{15}OBrNH_4$ $[M + NH_4]^+$: 296.0650, found: 296.0642.

2-Allyl-2-(3-fluorophenyl)pent-4-enal (**1e**)

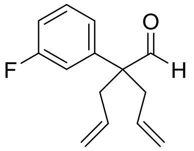

1e

Alcohol **1e-OH** (1.1 g, 5.0 mmol), oxalyl chloride (558 µL), DMSO (1.1 mL), triethylamine (3.5 mL) and DCM (25 mL) were used. The pure aldehyde **1d** was obtained after column chromatography as a pale-yellow liquid (812.0 mg, 74%). ^{1}H NMR (400 MHz, CDCl3) δ 9.54 (s, 1H), 7.40–7.32 (m, 1H), 7.06–6.92 (m, 3H), 5.64–5.46 (m, 2H), 5.15–5.01 (m, 4H), 2.77–2.61 (m, 4H). ^{13}C NMR (101 MHz, $CDCl_3$) δ 201.3, 163.3 (d, *J*= 247.5 Hz), 141.0 (d, *J*= 6.1 Hz), 132.3, 130.4 (d, *J*= 8.1 Hz), 123.5 (d, *J*= 8.1 Hz), 119.4, 114.9 (d, *J*= 23.2 Hz), 114.6 (d, *J*= 21.2 Hz), 56.8 (d, *J*= 2.0 Hz), 37.0; IR (ATR): 3079, 2980, 2918, 2807, 2715, 1724, 1640, 1612, 1587, 918, 784, 697 cm^{-1}; HRMS (CI-TOF) *m* / *z* calcd for $C_{14}H_{15}OFNH_4$ $[M + NH_4]^+$: 236.1451, found: 236.1447.

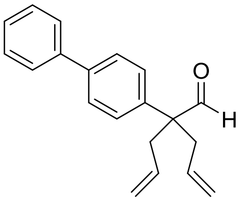

1f

2-([1,1'-Biphenyl]-4-yl)-2-allylpent-4-enal (**1f**)

Alcohol **1f-OH** (900 mg, 5.0 mmol), oxalyl chloride (360 µL), DMSO (700 µL), triethylamine (2.3 mL) and DCM (20 mL) were used. The pure aldehyde **1f** was obtained after column chromatography as a colorless liquid (772.3 mg, 86%). ^{1}H NMR (400 MHz, $CDCl_3$) δ 9.58 (s, 1H), 7.68–7.55 (m, 4H), 7.50–7.42 (m, 2H), 7.38 (dt, *J* = 9.4, 4.3 Hz, 1H), 7.33–7.28 (m, 2H), 5.61 (ddt, *J* = 17.3, 10.1, 7.3 Hz, 2H), 5.18–5.05 (m, 4H), 2.80–2.74 (m, 4H); ^{13}C NMR (100 MHz, $CDCl_3$) δ 201.9, 140.5, 137.1, 132.8, 129.0, 128.2, 127.7, 127.6, 127.2, 119.2, 56.8, 36.9; IR (ATR): 3077, 3029, 2979, 2917, 2803, 2711, 1722, 1640, 1600, 1486, 916, 764, 732, 696 cm^{-1}; HRMS (ESI-TOF) *m* / *z* calcd for $C_{20}H_{20}ONa$ $[M + Na]^+$: 299.1412, found: 299.1403.

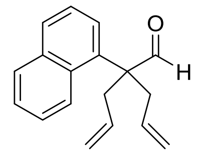

1g

2-Allyl-2-(naphthalen-1-yl)pent-4-enal (1g)

Alcohol **1g-OH** (510 mg, 2.0 mmol), oxalyl chloride (223 μL), DMSO (426 μL), triethylamine (1.4 mL) and DCM (10 mL) were used. The pure aldehyde **1g** was obtained after column chromatography as a colorless liquid (432 mg, 86%). ^{1}H NMR (400 MHz, $CDCl_3$) δ 9.75 (s, 1H), 7.88 (dddd, J = 8.3, 7.3, 4.7, 3.0 Hz, 3H), 7.65–7.39 (m, 4H), 5.70–5.41 (m, 2H), 5.13–5.00 (m, 4H), 3.06–2.86 (m, 4H). ^{13}C NMR (100 MHz, $CDCl_3$) δ 205.2, 134.9, 134.6, 132.7, 131.6, 129.8, 129.3, 126.7, 126.5, 125.7, 125.3, 124.3, 119.2, 57.3, 37.1; IR (ATR): 3076, 2978, 2806, 2709, 1718, 1639, 1599, 916, 775 cm^{-1}; HRMS (ESI-TOF) m / z calcd for $C_{18}H_{18}ONa$ $[M + Na]^+$: 273.1255, found: 273.1252.

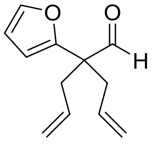

1h

2-Allyl-2-(furan-2-yl)pent-4-enal (1h)

Alcohol **1h-OH** (410 mg, 2.13 mmol), oxalyl chloride (237 μL), DMSO (426 μL), triethylamine (1.4 mL) and DCM (10 mL) were used. The pure aldehyde **1h** was obtained after column chromatography (231.2 mg, 57%) as a colorless liquid. ^{1}H NMR (400 MHz, $CDCl_3$) δ 9.54 (s, 1H), 7.43 (dd, J = 1.9, 0.8 Hz, 1H), 6.38 (dd, J = 3.3, 1.9 Hz, 1H), 6.28 (dd, J = 3.3, 0.8 Hz, 1H), 5.60 (ddt, J = 17.4, 10.2, 7.3 Hz, 2H), 5.22–5.02 (m, 4H), 2.80–2.55 (m, 4H); ^{13}C NMR (101 MHz, $CDCl_3$) δ 199.8, 152.6, 142.9, 132.3, 119.2, 110.6, 108.5, 54.9, 35.7. IR (ATR): 3079, 2981, 2918, 2807, 2716, 1728, 1641, 918, 736 cm^{-1}; HRMS (CI-TOF) m / z calcd for $C_{12}H_{14}O_2NH_4$ $[M + NH_4]^+$: 208.1338, found: 208.1348.

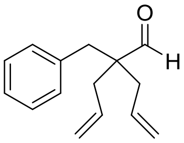

1i

2-Allyl-2-benzylpent-4-enal (1i)

Alcohol **1i-OH** (1.1 g, 5.09 mmol), oxalyl chloride (568 μL), DMSO (1.08 mL), triethylamine (2.1 mL) and DCM (30 mL) were used. The pure aldehyde **1h** was obtained after column chromatography (932 mg, 86%) as a colorless liquid. The ^{1}H NMR spectrum matched the literature reported values.[1] ^{1}H NMR (400 MHz, $CDCl_3$) δ 9.61 (s, 1H), 7.35–7.15 (m, 3H), 7.16–7.02 (m, 2H), 5.78 (ddt, J = 17.6, 10.3, 7.3 Hz, 2H), 5.21–5.01 (m, 4H), 2.87 (s, 2H), 2.41–2.18 (m, 4H); ^{13}C NMR (101 MHz, $CDCl_3$) δ 206.1, 136.6, 132.9, 130.4, 128.4, 126.8, 119.3, 53.1, 39.7, 36.7.

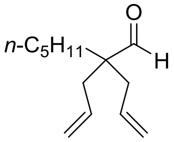

1j

2,2-Diallylheptanal (1j)

Alcohol **1j-OH** (600 mg, 3.05 mmol), oxalyl chloride (340 μL), DMSO (650 μL), triethylamine (2.1 mL) and DCM (20 mL) were used. The pure aldehyde **1j** was obtained after column chromatography (515 mg, 87%) as a pale-yellow liquid. ^{1}H NMR (400 MHz, $CDCl_3$) δ 9.50 (s, 1H), 5.69 (m, 2H), 5.09 (m, 4H), 2.28 (dt, J= 7.4, 1.1 Hz, 4H), 1.52–1.48 (m, 2H), 1.30–1.22 (m, 6H), 0.88 (t, J = 7.2 Hz, 3H); ^{13}C NMR (100 MHz, $CDCl_3$) δ 206.4, 133.0, 118.7, 52.2, 36.6, 32.7, 32.5, 23.3, 22.6, 14.1; IR (ATR): 3078, 2930, 2860, 2710, 1725, 1640, 1446, 914 cm^{-1}; HRMS (CI-TOF) m / z calcd for $C_{13}H_{22}ONH_4$ $[M + NH_4]^+$: 212.2014, found: 212.2009.

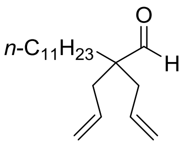

1k

2,2-Diallyltridecanal (1k)

Alcohol **1k-OH** (1.08 g, 3.86 mmol), oxalyl chloride (430 μL), DMSO (820 μL), triethylamine (3.24 mL) and DCM (20 mL) were used. The pure aldehyde **1k** was obtained after column chromatography (803 mg, 80%) as a colourless liquid. ^{1}H NMR (400 MHz, $CDCl_3$) δ 9.49 (s, 1H), 5.81–5.56 (m, 2H), 5.19–4.97 (m,

4H), 2.37–2.19 (m, 4H), 1.55–1.44 (m, 2H), 1.37–1.11 (m, 19H), 0.89 (t, J = 6.9 Hz, 3H). ^{13}C NMR (100 MHz, $CDCl_3$) δ 206.4, 133.0, 118.7, 52.2, 36.6, 32.8, 32.1, 30.3, 29.8, 29.7, 29.6, 29.5, 23.6, 22.8, 14.3; IR (ATR): 3078, 2923, 2853, 2710, 1727, 1640, 1466, 915 cm^{-1}; HRMS (CI-TOF) m / z calcd for $C_{19}H_{34}ONH_4$ [M + NH_4]$^+$: 296.2953, found: 296.2946.

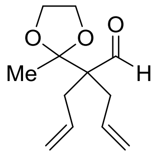

1l

2-Allyl-2-(2-methyl-1,3-dioxolan-2-yl)pent-4-enal (**1l**)

Alcohol **1l-OH** (1.0 g, 4.71 mmol),[1] oxalyl chloride (526 μL), DMSO (836 μL), triethylamine (3.3 mL) and DCM (50 mL) were used. The pure aldehyde **1l** was obtained after column chromatography (764 mg, 77%) as a colorless liquid. The ^{1}H NMR spectrum matched the literature reported values.[1] ^{1}H NMR (400 MHz, $CDCl_3$) δ 9.69 (s, 1H), 5.78 (ddt, J = 17.1, 10.0, 7.2 Hz, 2H), 5.11–5.04 (m, 4H), 4.01–3.93 (m, 4H), 2.49 (ddt, J = 7.2, 4.3, 1.3 Hz, 4H), 1.27 (s, 3H).

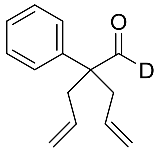

d-**1a**

Synthesis of 2-allyl-2-phenylpent-4-enal-*d* (*d*-**1a**)

Alcohol (434.7 mg, 2.13 mmol), oxalyl chloride (223 μL), DMSO (426 μL), triethylamine (1.4 mL) and DCM (10 mL) were used. The pure aldehyde **1h** was obtained after column chromatography (345 mg, 80%) as a colourless oil. ^{1}H NMR (400 MHz, $CDCl_3$) δ 7.44–7.36 (m, 2H), 7.34–7.28 (m, 1H), 7.25–7.19 (m, 2H), 5.57 (ddt, J = 17.4, 10.2, 7.3 Hz, 2H), 5.18–4.98 (m, 4H), 2.77–2.66 (m, 4H). ^{13}C NMR (101 MHz, $CDCl_3$) δ 138.2, 132.9, 129.1, 127.8, 127.69, 119.2, 56.9, 37.0; LRMS (ESI-TOF) m / z calcd for $C_{15}H_{16}O_3Na$ [M + Na]$^+$: 267.0997, found: 267.0995; LRMS (EI) m / z calcd for [$C_{14}H_{15}DO$]$^+$: 201.1, found: 201.1.

4 X-Ray Crystallographic Data for (±)-4a and (*S*)-9

- **(±)-4a**

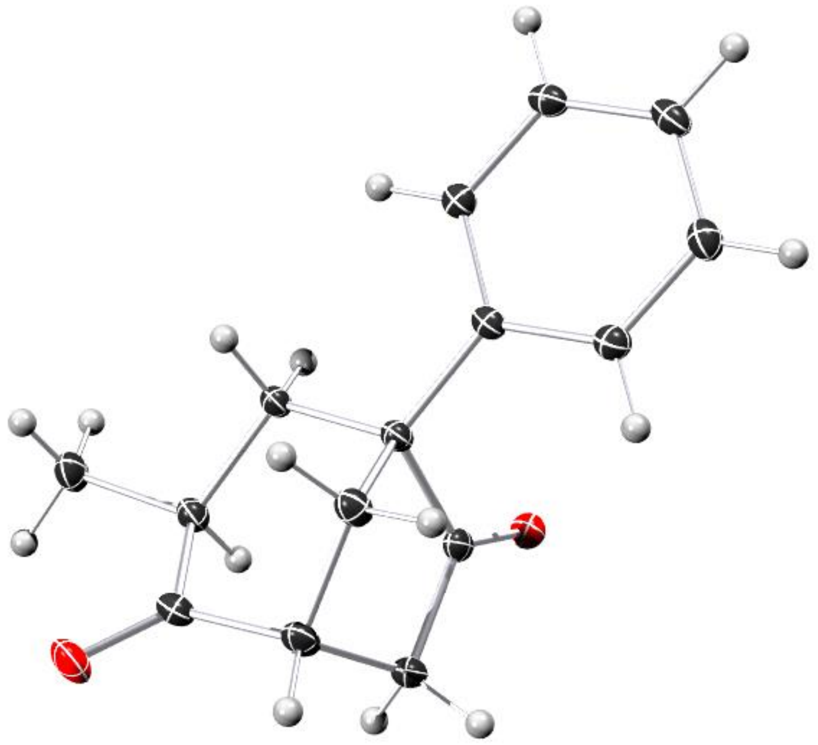

Figure S1. Single Crystal X-Ray Structure of (±)-**4a**.

(±)-**4a**. The structure was unambiguously determined by single-crystal X-ray crystallography. Approximately 10 mg of pure material was dissolved in CH_2Cl_2 (approximately 2 mL) in a 20 mL scintillation vial and topped with a layer of hexanes (approximate 0.5 mL). The vial was loosely capped to allow for slow evaporation of solvents.

X-Ray Data Collection*, *Structure Solution and Refinement

A colorless crystal of approximate dimensions 0.338 x 0.244 x 0.206 mm was mounted on a glass fiber and transferred to a Bruker SMART APEX II diffractometer. The APEX2[1] program package was used to determine the unit-cell parameters and for data collection (10 sec/frame scan time for a sphere of diffraction data). The raw frame data was processed using SAINT[2] and SADABS[3] to yield the reflection data file. Subsequent calculations were carried out using the SHELXTL[4] program. There were no systematic absences nor any diffraction symmetry other than the Friedel condition. The centrosymmetric triclinic space group $P\bar{1}$ was assigned and later determined to be correct.

The structure was solved by direct methods and refined on F^2 by full-matrix least-squares techniques. The analytical scattering factors[5] for neutral atoms were used throughout the analysis. Hydrogen atoms were located from a difference-Fourier map and refined (x,y,z and U_{iso}).

At convergence, wR2 = 0.0994 and Goof = 1.049 for 218 variables refined against 2863 data (0.73 A), R1 = 0.0360 for those 2498 data with $I > 2.0\sigma(I)$.

CCDC 1056687 contains the X-ray crystallographic data for this compound. This data can be obtained free of charge from The Cambridge Crystallographic Data Centre via http://www.ccdc.cam.ac.uk/data_request/cif.

- **(*S*)-9**

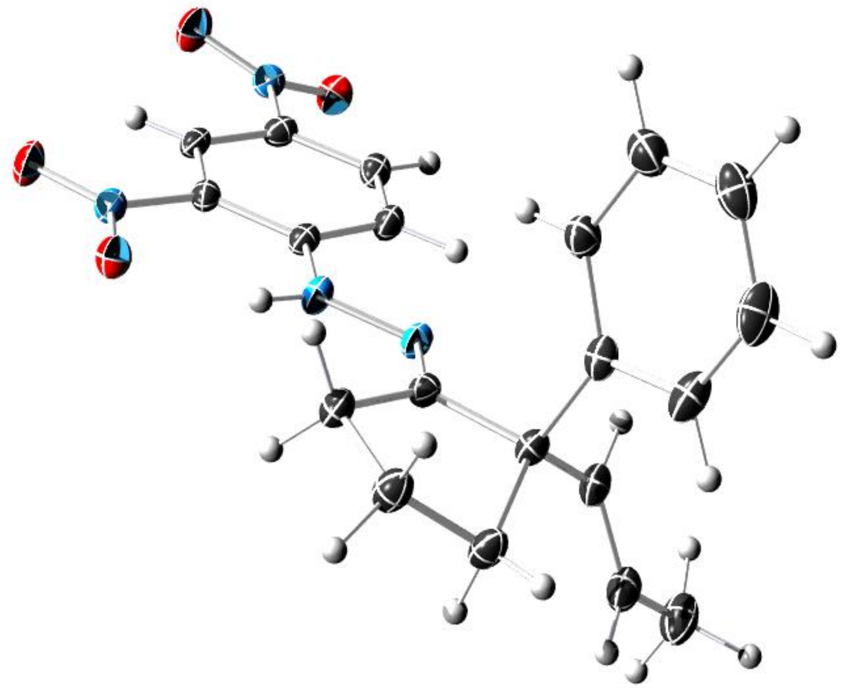

Figure S2. Single Crystal X-Ray Structure of (*S*)-**9**.

(*S*)-**9**. The structure was unambiguously determined by single-crystal X-ray crystallography. There were four molecules of the formula-unit present. Only one has been shown for clarity. Approximately 10 mg of pure material was dissolved in CH_2Cl_2 (approximately 0.5 mL) in a 1-dram scintillation vial and topped with a layer of hexanes (approximate 0.5 mL). The vial was loosely capped to allow for slow evaporation of solvents.

X-ray Data Collection, Structure Solution and Refinement

An orange crystal of approximate dimensions 0.144 x 0.300 x 0.574 mm was mounted on a glass fiber and transferred to a Bruker SMART APEX II diffractometer. The APEX2 program package was used to determine the unit-cell parameters and for data collection (15 sec/frame scan time for a sphere of diffraction data). The raw frame data was processed using SAINT and SADABS to yield the reflection data file. Subsequent calculations were carried out using the SHELXTL program. There were no systematic absences. The noncentrosymmetric triclinic space group *P*1 was assigned and later determined to be correct.

The structure was solved by direct methods and refined on F^2 by full-matrix least-squares techniques. The analytical scattering factors for neutral atoms were used throughout the analysis. Hydrogen atoms were included using a riding model. There were four molecules of the formula-unit present (Z = 4).

At convergence, wR2 = 0.0955 and Goof = 1.025 for 1013 variables refined against 17563 data (0.73Å), R1 = 0.0377 for those 16382 data with $I > 2.0\sigma(I)$. The Flack parameter was low with a high standard deviation (-0.4(3)). The absolute structure assignment was based on the synthetic method employed.

CCDC 1062118 contains the X-ray crystallographic data for this compound. This data can be obtained free of charge from The Cambridge Crystallographic Data Centre via http://www.ccdc.cam.ac.uk/data_request/cif.

Table 1. Crystal data and structure refinement for vmd20.

Identification code	vmd20 (Jung Woo Park)	
Empirical formula	$C_{20} H_{20} N_4 O_4$	
Formula weight	380.40	
Temperature	88(2) K	
Wavelength	0.71073 Å	
Crystal system	Triclinic	
Space group	*P*1	
Unit cell dimensions	a = 7.9466(5) Å	= 78.9198(8)°.
	b = 14.9541(10) Å	= 79.5663(9)°.
	c = 16.2556(11) Å	= 82.7612(9)°.
Volume	1856.0(2) Å^3	
Z	4	
Density (calculated)	1.361 Mg/m^3	
Absorption coefficient	0.097 mm^{-1}	
F(000)	800	
Crystal color	orange	
Crystal size	0.574 x 0.300 x 0.144 mm^3	
Theta range for data collection	1.729 to 29.198°	
Index ranges	$-10 \leq h \leq 10$, $-19 \leq k \leq 20$, $-21 \leq l \leq 21$	
Reflections collected	23347	
Independent reflections	17563 [R(int) = 0.0144]	
Completeness to theta = 25.500°	99.8 %	
Absorption correction	Semi-empirical from equivalents	
Max. and min. transmission	0.8622 and 0.8072	
Refinement method	Full-matrix least-squares on F^2	
Data / restraints / parameters	17563 / 3 / 1013	
Goodness-of-fit on F^2	1.025	
Final R indices [I>2sigma(I) = 16382 data]	R1 = 0.0377, wR2 = 0.0925	
R indices (all data, 0.73Å)	R1 = 0.0413, wR2 = 0.0955	
Absolute structure parameter	-0.4(3)	
Largest diff. peak and hole	0.308 and -0.207 e.Å^{-3}	

5 NMR Spectra

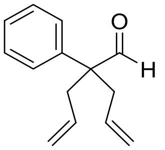

(**1a**)

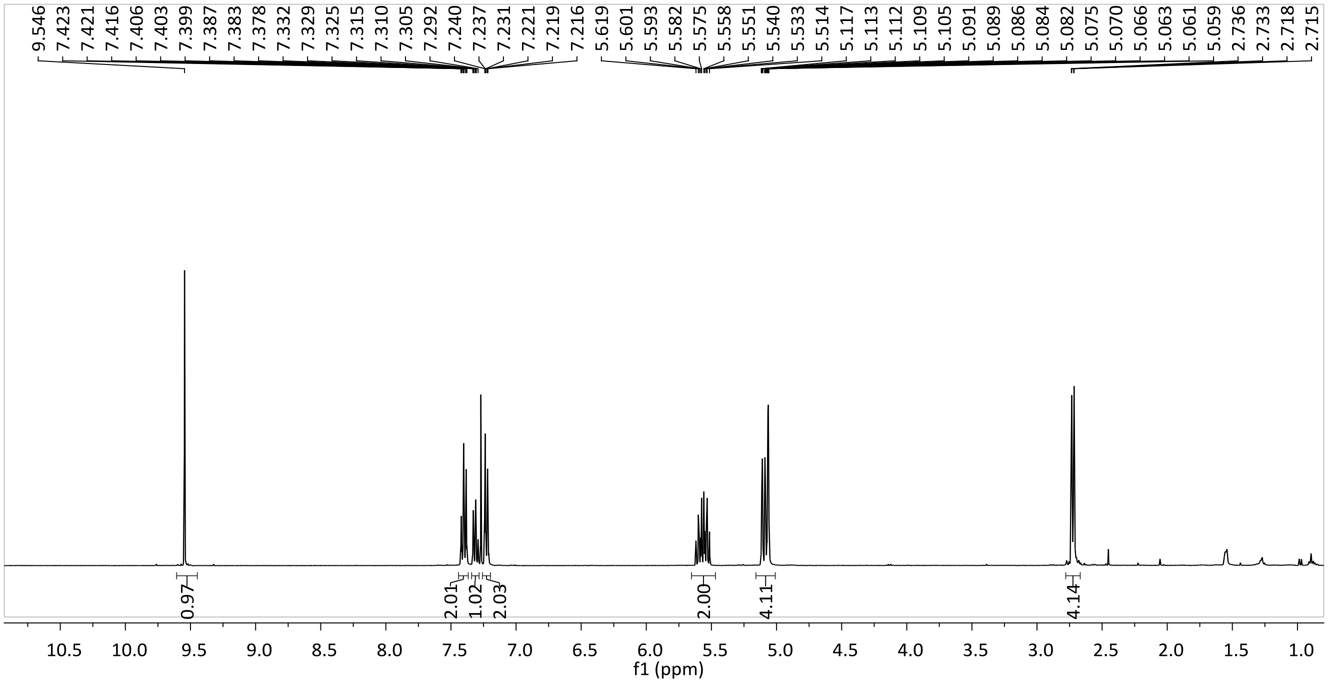

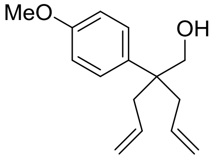
MeO
OH

(1b-OH)

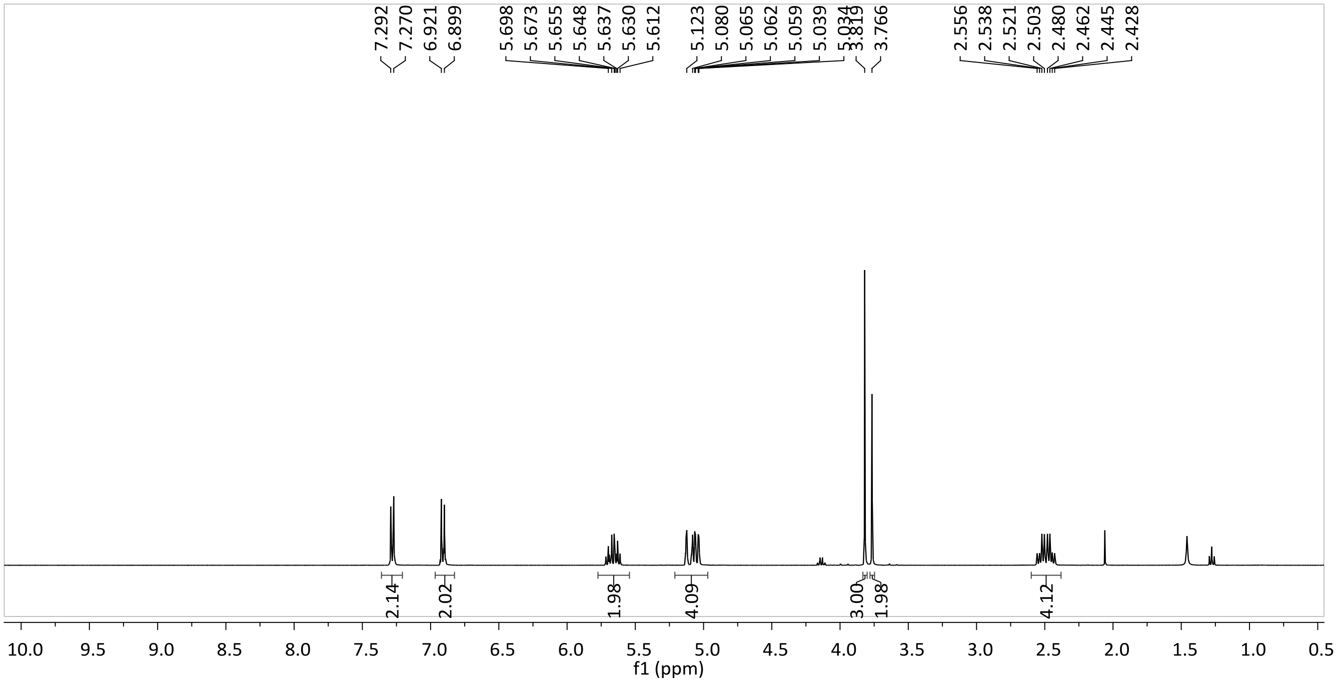
7.292
7.270
6.921
6.899
5.698
5.673
5.655
5.648
5.637
5.630
5.612
5.123
5.080
5.065
5.062
5.059
5.039
5.034
3.819
3.766
2.556
2.538
2.521
2.503
2.480
2.462
2.445
2.428
2.14
2.02
1.98
4.09
3.00
1.98
4.12
10.0 9.5 9.0 8.5 8.0 7.5 7.0 6.5 6.0 5.5 5.0 4.5 4.0 3.5 3.0 2.5 2.0 1.5 1.0 0.5
f1 (ppm)

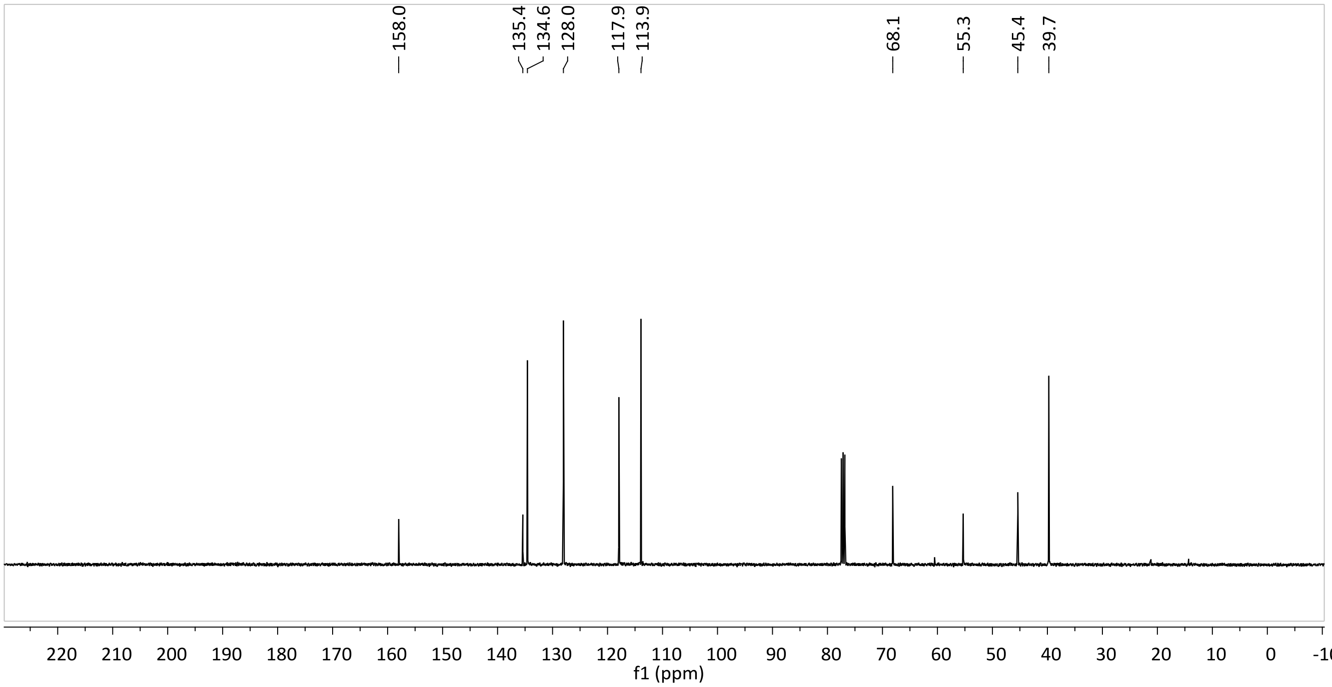
158.0
135.4
134.6
128.0
117.9
113.9
68.1
55.3
45.4
39.7
220 210 200 190 180 170 160 150 140 130 120 110 100 90 80 70 60 50 40 30 20 10 0 -10
f1 (ppm)

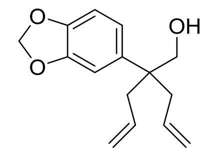
OH
O
O

(1c-OH)

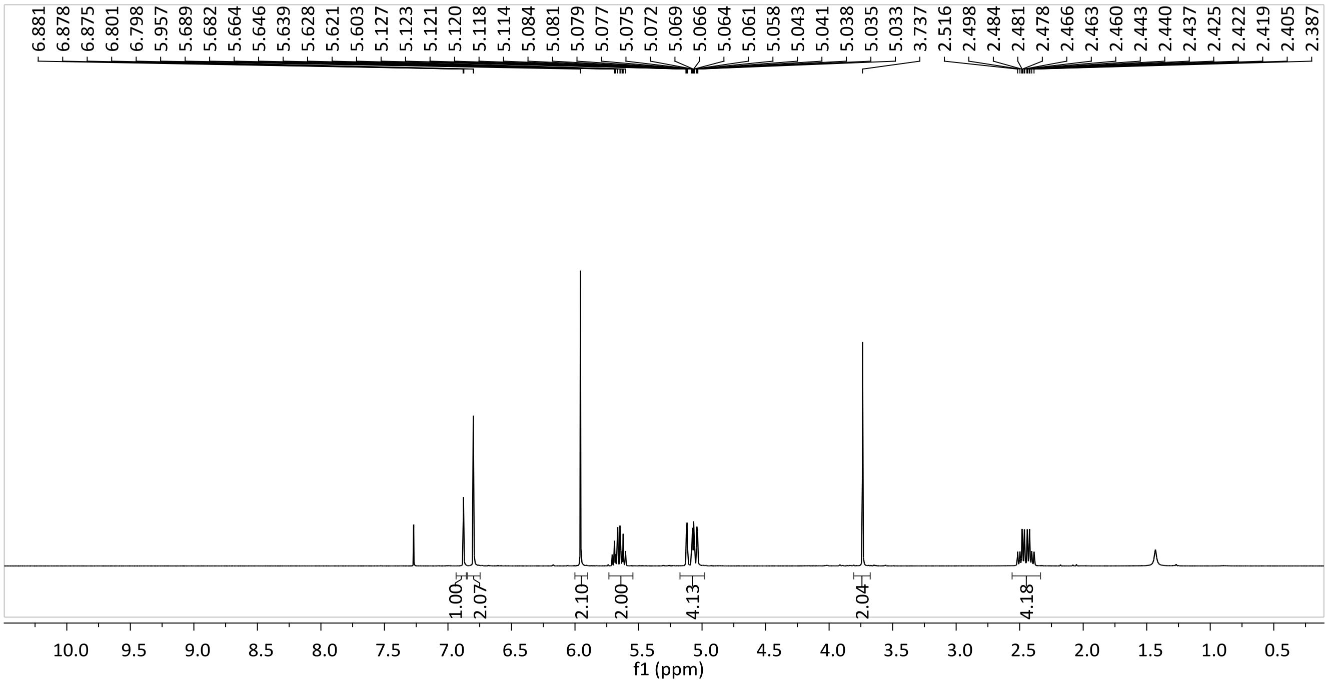
6.881 6.878 6.875 6.801 6.798 5.957 5.689 5.682 5.664 5.646 5.639 5.628 5.621 5.603 5.127 5.123 5.121 5.120 5.118 5.114 5.084 5.081 5.079 5.077 5.075 5.072 5.069 5.066 5.064 5.061 5.058 5.043 5.041 5.038 5.035 5.033 3.737 2.516 2.498 2.484 2.481 2.478 2.466 2.463 2.460 2.443 2.440 2.437 2.425 2.422 2.419 2.405 2.387
1.00
2.07
2.10
2.00
4.13
2.04
4.18
10.0 9.5 9.0 8.5 8.0 7.5 7.0 6.5 6.0 5.5 5.0 4.5 4.0 3.5 3.0 2.5 2.0 1.5 1.0 0.5
f1 (ppm)

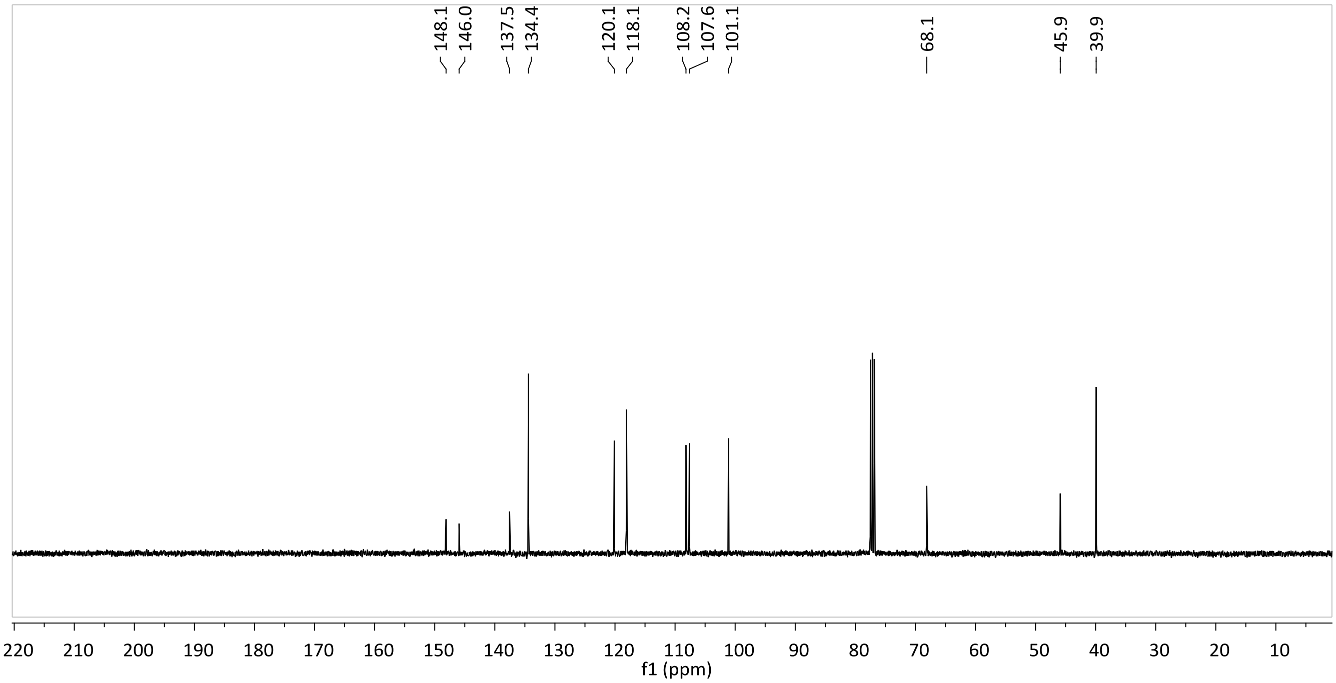
148.1 146.0 137.5 134.4 120.1 118.1 108.2 107.6 101.1 68.1 45.9 39.9
220 210 200 190 180 170 160 150 140 130 120 110 100 90 80 70 60 50 40 30 20 10
f1 (ppm)

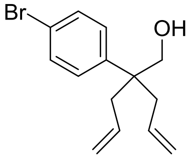

(**1d-OH**)

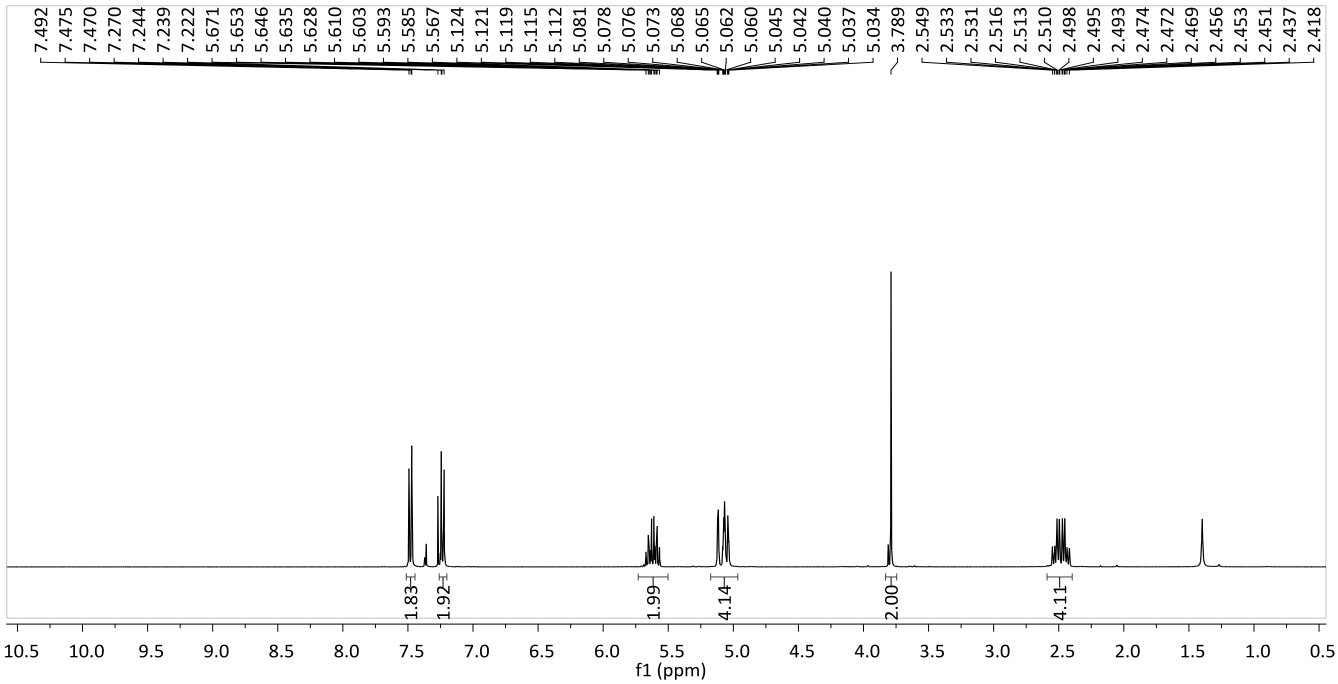
7.492
7.475
7.470
7.270
7.244
7.239
7.222
5.671
5.653
5.646
5.635
5.628
5.610
5.603
5.593
5.585
5.567
5.124
5.121
5.119
5.115
5.112
5.081
5.078
5.076
5.073
5.068
5.065
5.062
5.060
5.045
5.042
5.040
5.037
5.034
3.789
2.549
2.533
2.531
2.516
2.513
2.510
2.498
2.495
2.493
2.474
2.472
2.469
2.456
2.453
2.451
2.437
2.418
1.83
1.92
1.99
4.14
2.00
4.11
10.5 10.0 9.5 9.0 8.5 8.0 7.5 7.0 6.5 6.0 5.5 5.0 4.5 4.0 3.5 3.0 2.5 2.0 1.5 1.0 0.5
f1 (ppm)

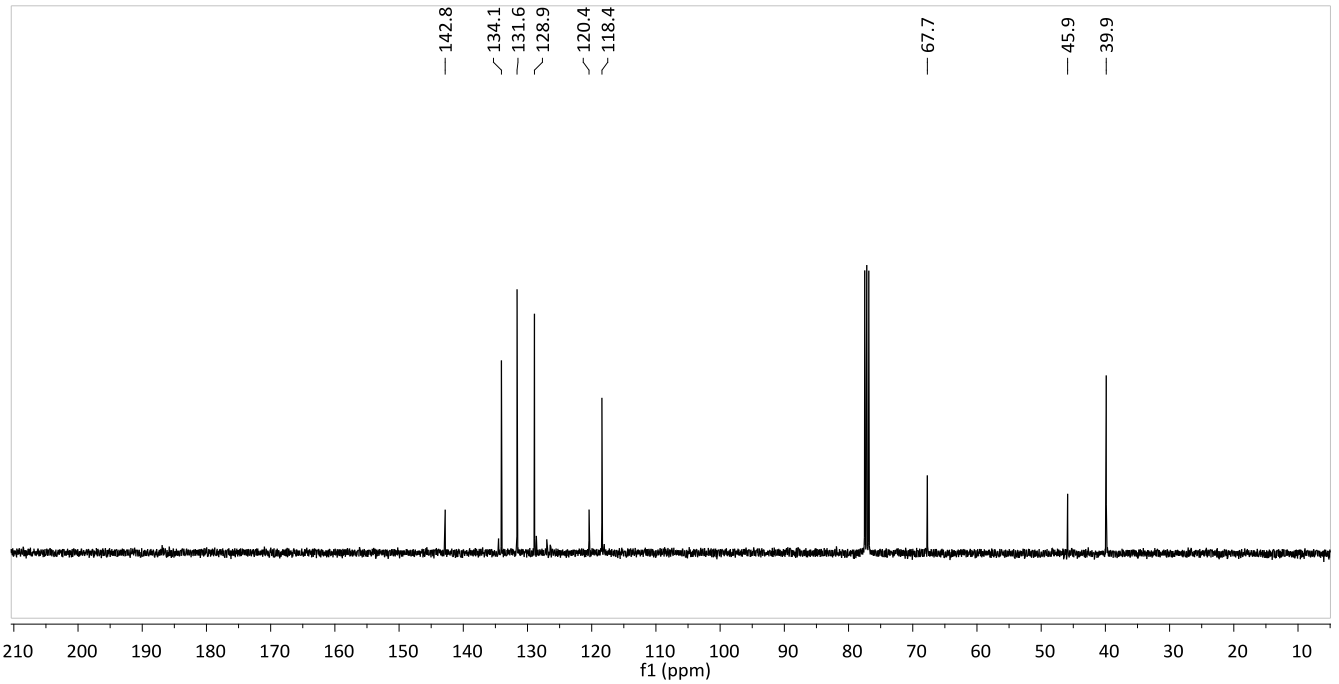
142.8
134.1
131.6
128.9
120.4
118.4
67.7
45.9
39.9
210 200 190 180 170 160 150 140 130 120 110 100 90 80 70 60 50 40 30 20 10
f1 (ppm)

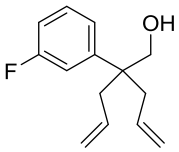
OH
F

(**1e-OH**)

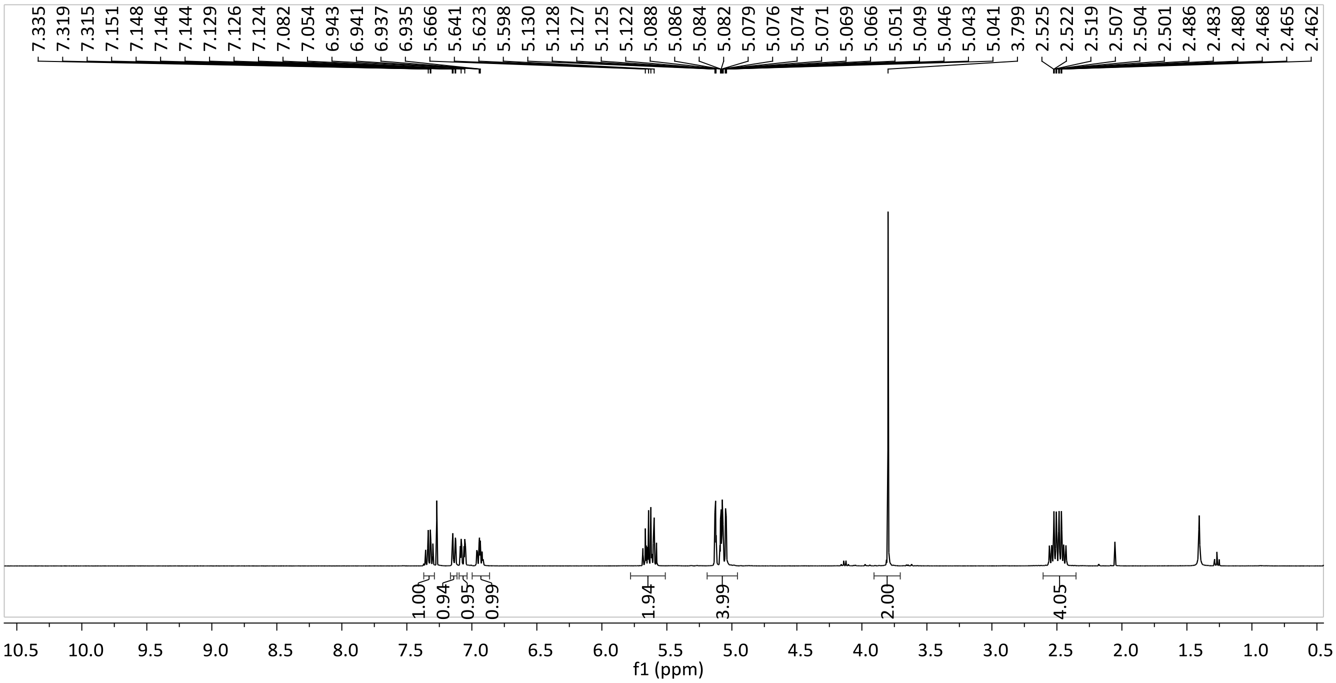
7.335
7.319
7.315
7.151
7.148
7.146
7.144
7.129
7.126
7.124
7.082
7.054
6.943
6.941
6.937
6.935
5.666
5.641
5.623
5.598
5.130
5.128
5.127
5.125
5.122
5.088
5.086
5.084
5.082
5.079
5.076
5.074
5.071
5.069
5.066
5.051
5.049
5.046
5.043
5.041
3.799
2.525
2.522
2.519
2.507
2.504
2.501
2.486
2.483
2.480
2.468
2.465
2.462
1.00
0.94
0.95
0.99
1.94
3.99
2.00
4.05
10.5
10.0
9.5
9.0
8.5
8.0
7.5
7.0
6.5
6.0
5.5
5.0
4.5
4.0
3.5
3.0
2.5
2.0
1.5
1.0
0.5
f1 (ppm)

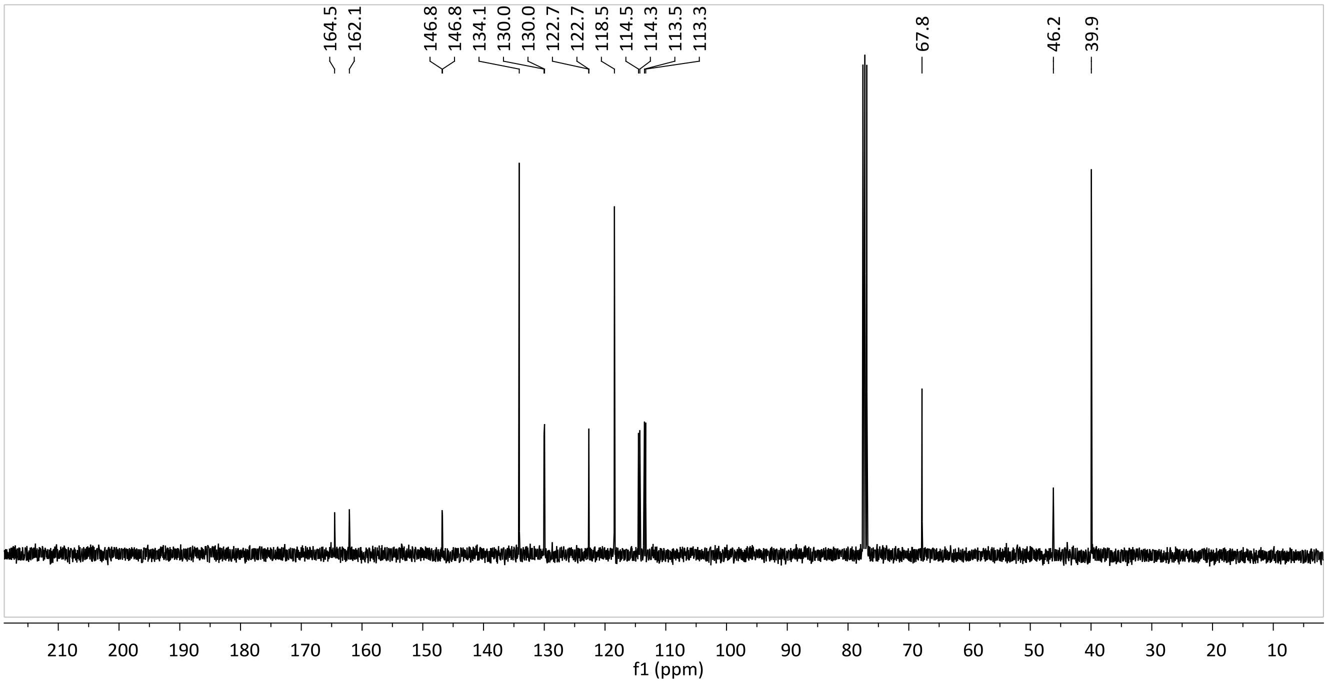
164.5
162.1
146.8
146.8
134.1
130.0
130.0
122.7
122.7
118.5
114.5
114.3
113.5
113.3
67.8
46.2
39.9
210
200
190
180
170
160
150
140
130
120
110
100
90
80
70
60
50
40
30
20
10
f1 (ppm)

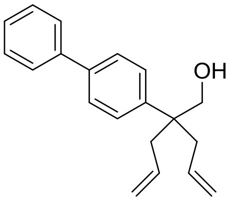

(1f-OH)

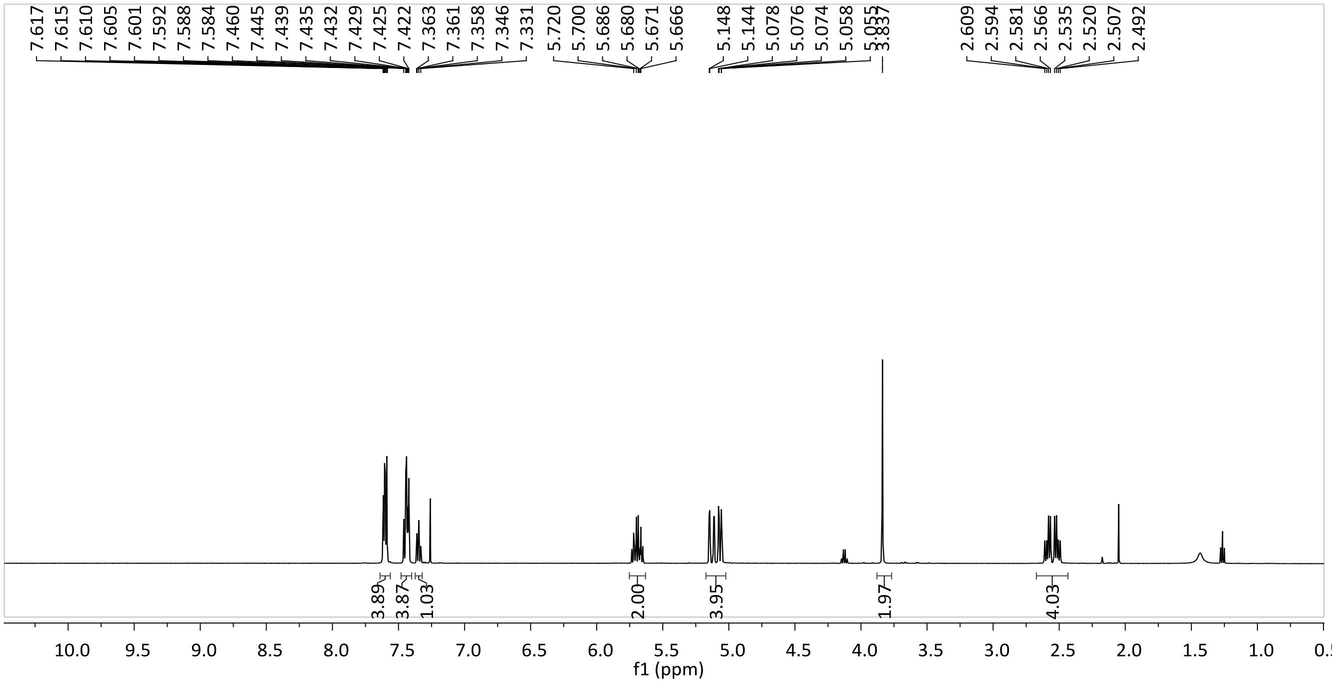
7.617
7.615
7.610
7.605
7.601
7.592
7.588
7.584
7.460
7.445
7.439
7.435
7.432
7.429
7.425
7.422
7.363
7.361
7.358
7.346
7.331
5.720
5.700
5.686
5.680
5.671
5.666
5.148
5.144
5.078
5.076
5.074
5.058
5.055
3.837
2.609
2.594
2.581
2.566
2.535
2.520
2.507
2.492
3.89
3.87
1.03
2.00
3.95
1.97
4.03
f1 (ppm)

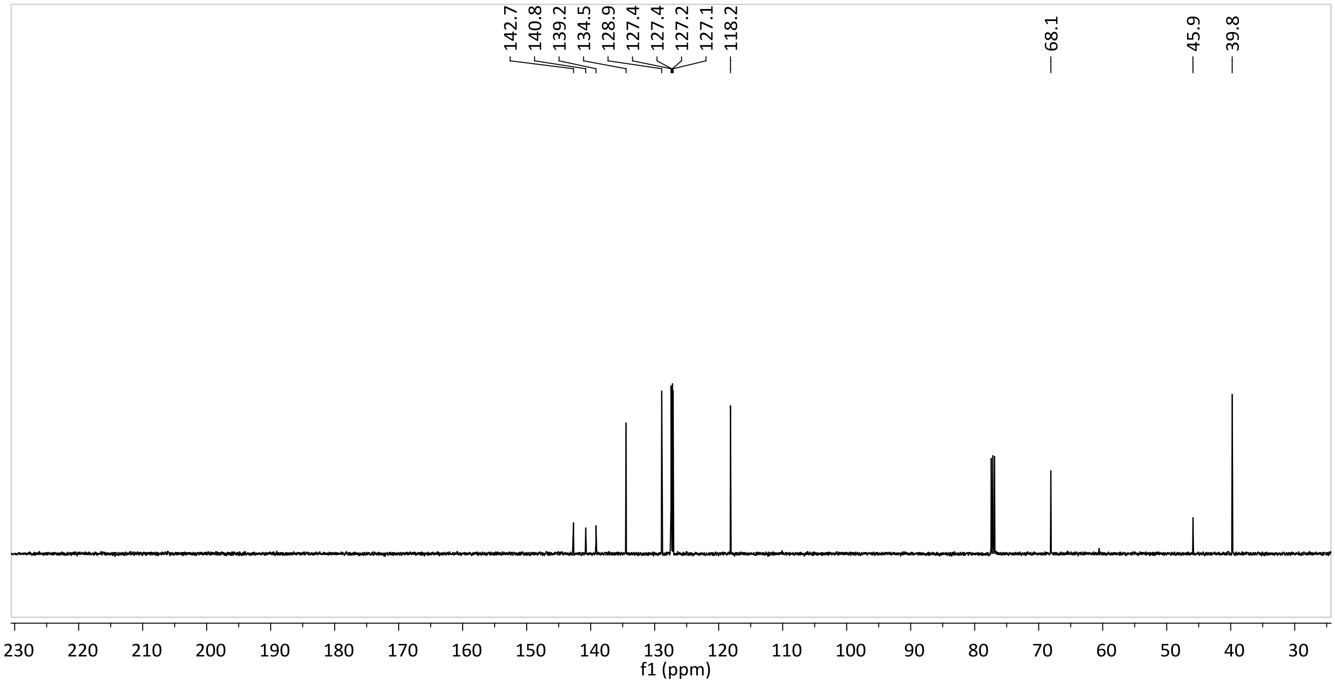
142.7
140.8
139.2
134.5
128.9
127.4
127.2
127.1
118.2
68.1
45.9
39.8
f1 (ppm)

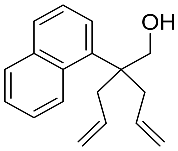
OH

(**1g-OH**)

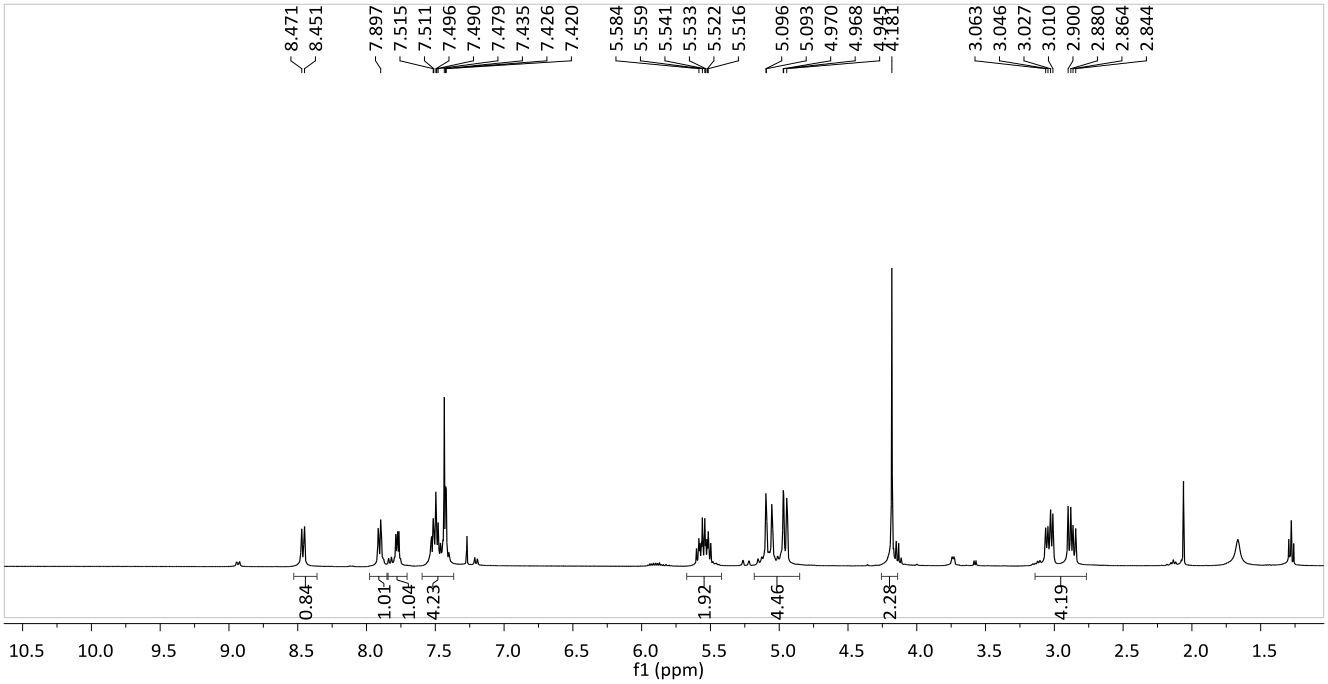
8.471
8.451
7.897
7.515
7.511
7.496
7.490
7.479
7.435
7.426
7.420
5.584
5.559
5.541
5.533
5.522
5.516
5.096
5.093
4.970
4.968
4.945
4.181
3.063
3.046
3.027
3.010
2.900
2.880
2.864
2.844
0.84
1.01
1.04
4.23
1.92
4.46
2.28
4.19
10.5 10.0 9.5 9.0 8.5 8.0 7.5 7.0 6.5 6.0 5.5 5.0 4.5 4.0 3.5 3.0 2.5 2.0 1.5
f1 (ppm)

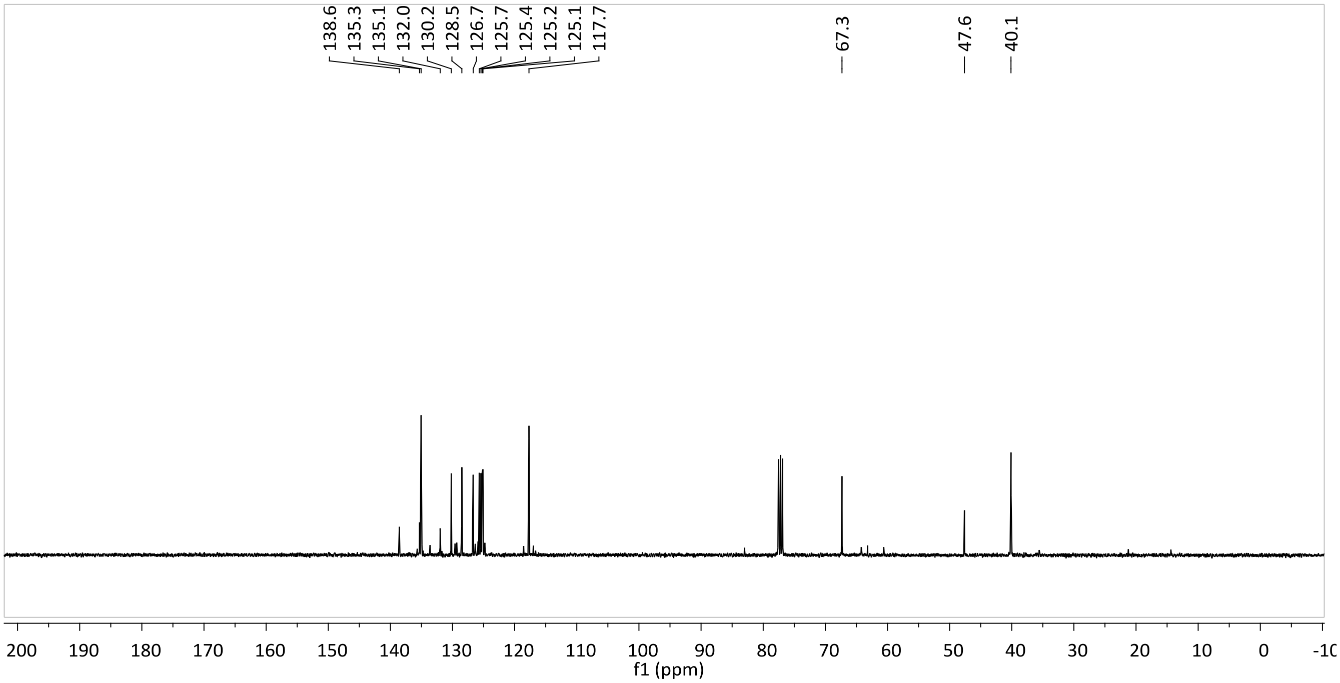
138.6
135.3
135.1
132.0
130.2
128.5
126.7
125.7
125.4
125.2
125.1
117.7
67.3
47.6
40.1
200 190 180 170 160 150 140 130 120 110 100 90 80 70 60 50 40 30 20 10 0 -10
f1 (ppm)

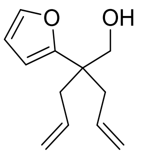
O
OH

(1h-OH)

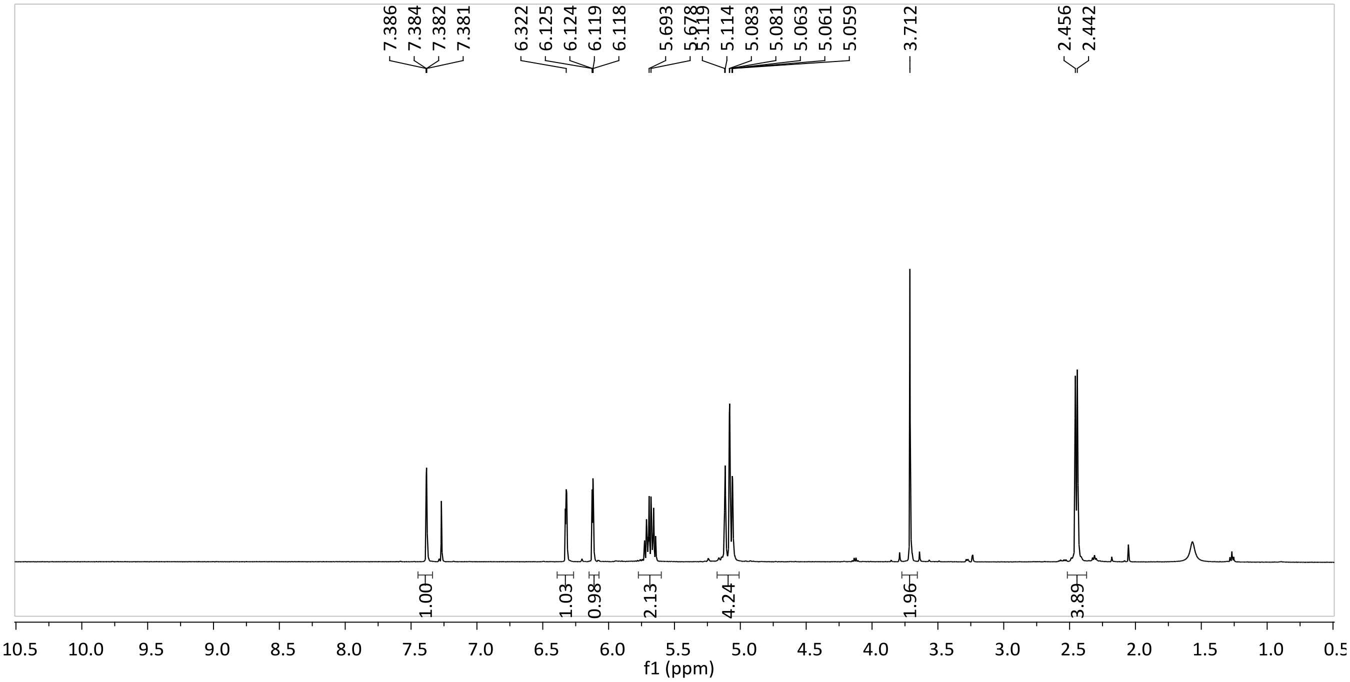
7.386
7.384
7.382
7.381
6.322
6.125
6.124
6.119
6.118
5.693
5.678
5.119
5.114
5.083
5.081
5.063
5.061
5.059
3.712
2.456
2.442
1.00
1.03
0.98
2.13
4.24
1.96
3.89
10.5 10.0 9.5 9.0 8.5 8.0 7.5 7.0 6.5 6.0 5.5 5.0 4.5 4.0 3.5 3.0 2.5 2.0 1.5 1.0 0.5
f1 (ppm)

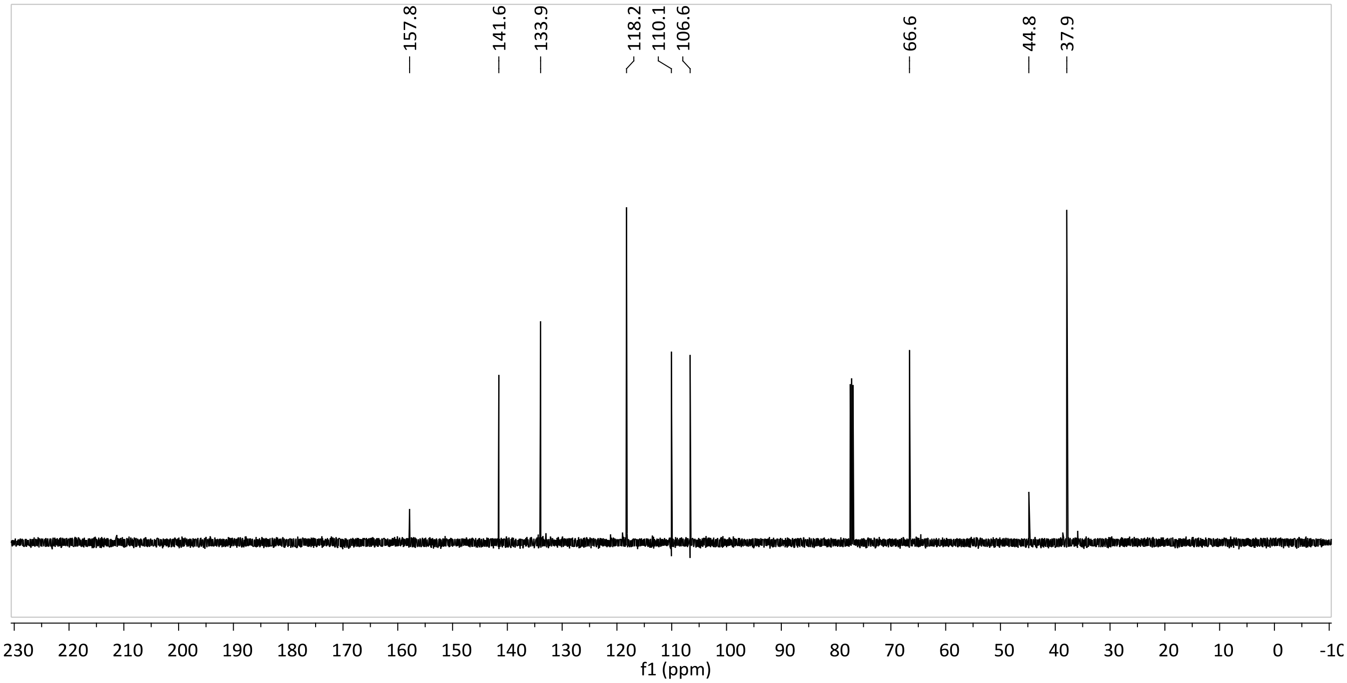
157.8
141.6
133.9
118.2
110.1
106.6
66.6
44.8
37.9
230 220 210 200 190 180 170 160 150 140 130 120 110 100 90 80 70 60 50 40 30 20 10 0 -10
f1 (ppm)

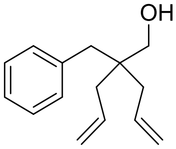

(**1i-OH**)

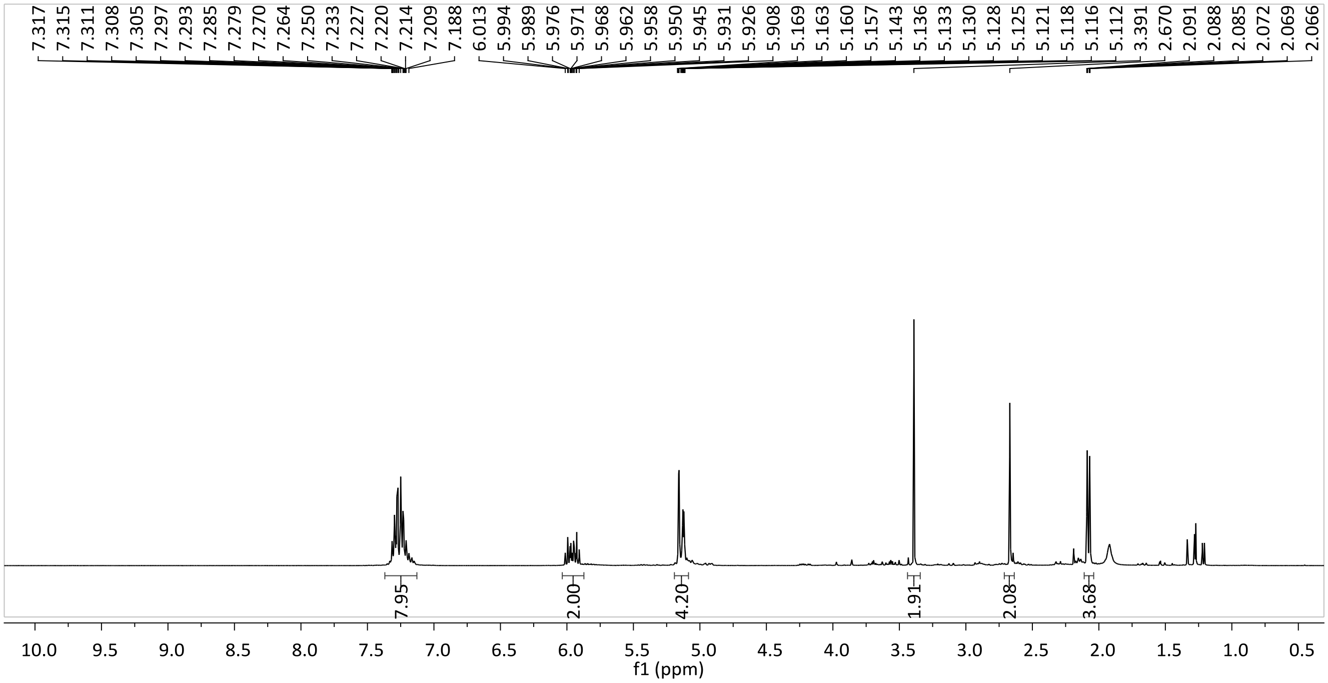

7.317
7.315
7.311
7.308
7.305
7.297
7.293
7.285
7.279
7.270
7.264
7.250
7.233
7.227
7.220
7.214
7.209
7.188
6.013
5.994
5.989
5.976
5.971
5.968
5.962
5.958
5.950
5.945
5.931
5.926
5.908
5.169
5.163
5.160
5.157
5.143
5.136
5.133
5.130
5.128
5.125
5.121
5.118
5.116
5.112
3.391
2.670
2.091
2.088
2.085
2.072
2.069
2.066
7.95
2.00
4.20
1.91
2.08
3.68
10.0 9.5 9.0 8.5 8.0 7.5 7.0 6.5 6.0 5.5 5.0 4.5 4.0 3.5 3.0 2.5 2.0 1.5 1.0 0.5
f1 (ppm)

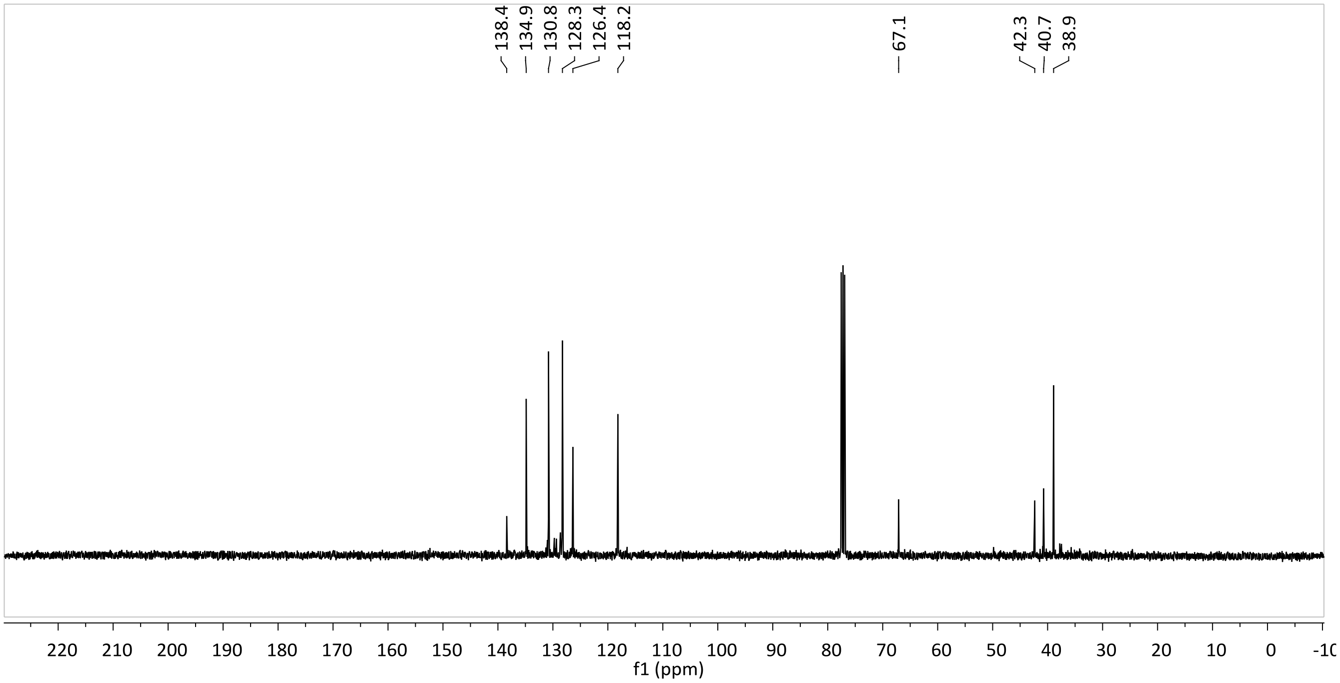

138.4
134.9
130.8
128.3
126.4
118.2
67.1
42.3
40.7
38.9
220 210 200 190 180 170 160 150 140 130 120 110 100 90 80 70 60 50 40 30 20 10 0 -10
f1 (ppm)

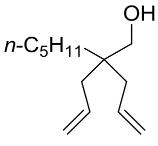
OH
n-C5H11

(1j-OH)

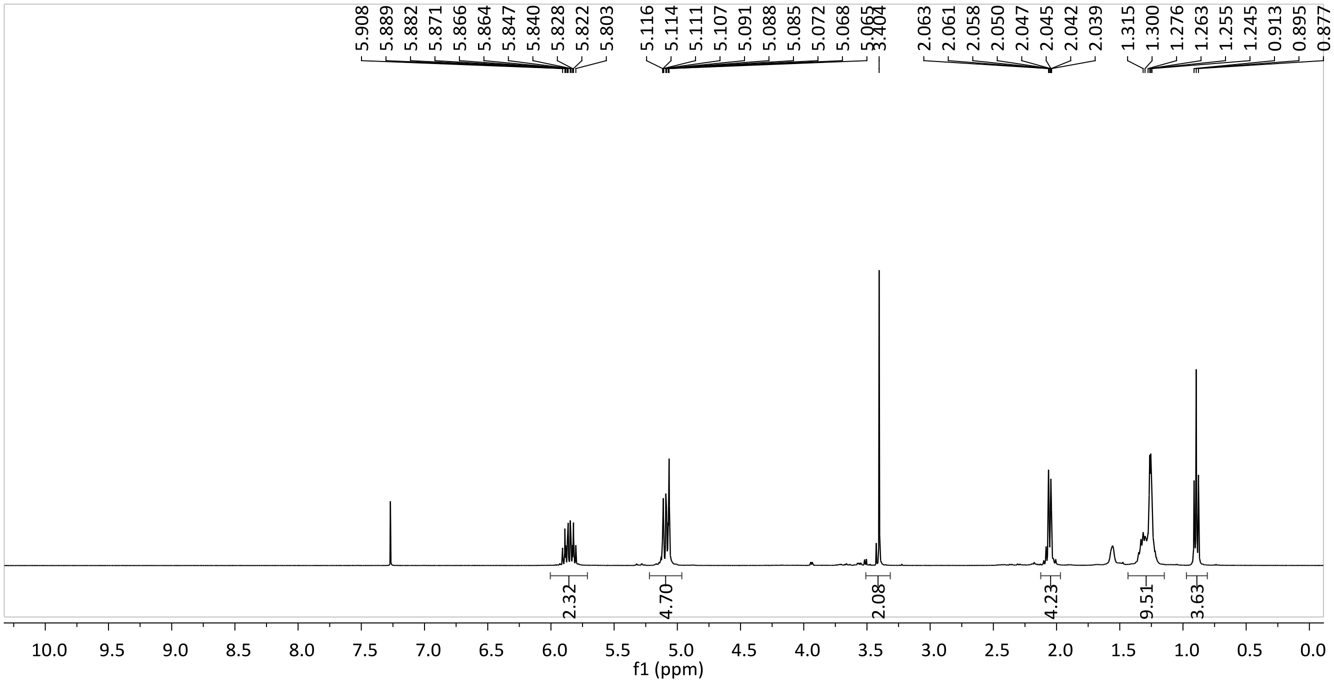
5.908
5.889
5.882
5.871
5.866
5.864
5.847
5.840
5.828
5.822
5.803
5.116
5.114
5.111
5.107
5.091
5.088
5.085
5.072
5.068
5.065
3.404
2.063
2.061
2.058
2.050
2.047
2.045
2.042
2.039
1.315
1.300
1.276
1.263
1.255
1.245
0.913
0.895
0.877
2.32
4.70
2.08
4.23
9.51
3.63
10.0 9.5 9.0 8.5 8.0 7.5 7.0 6.5 6.0 5.5 5.0 4.5 4.0 3.5 3.0 2.5 2.0 1.5 1.0 0.5 0.0
f1 (ppm)

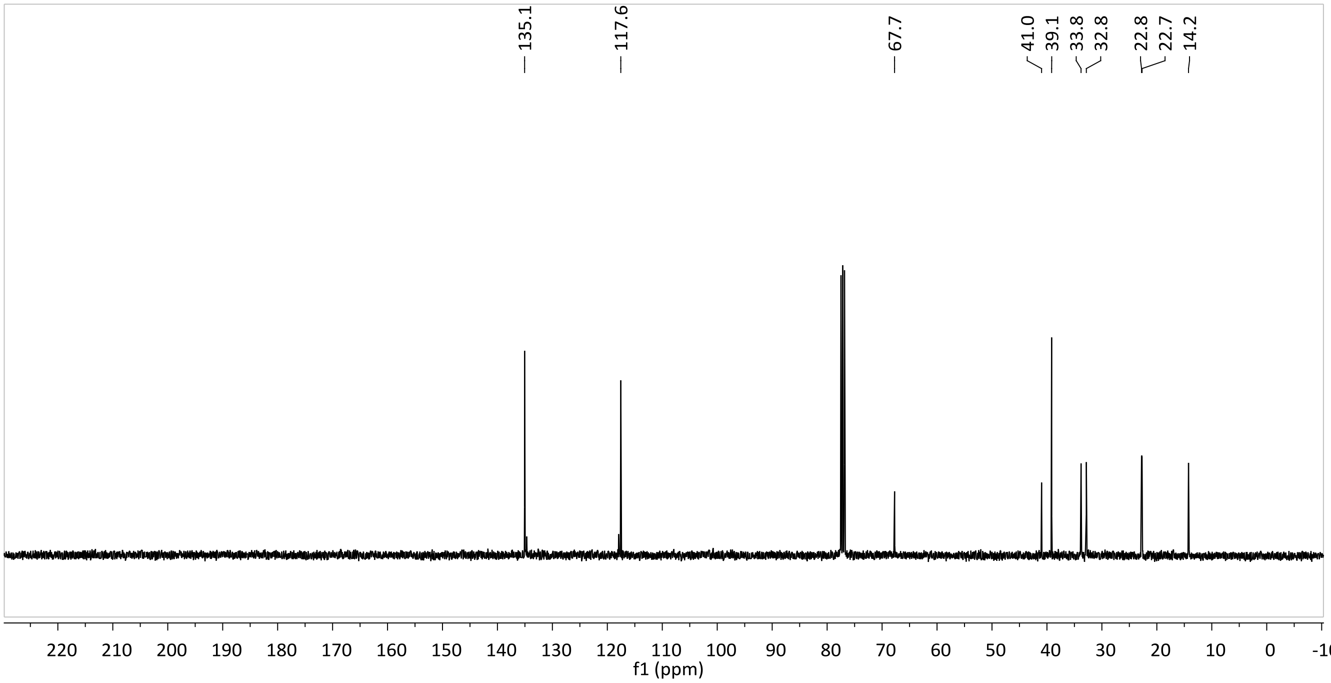
135.1
117.6
67.7
41.0
39.1
33.8
32.8
22.8
22.7
14.2
220 210 200 190 180 170 160 150 140 130 120 110 100 90 80 70 60 50 40 30 20 10 0 -10
f1 (ppm)

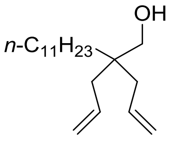
OH
n-C11H23

(1k-OH)

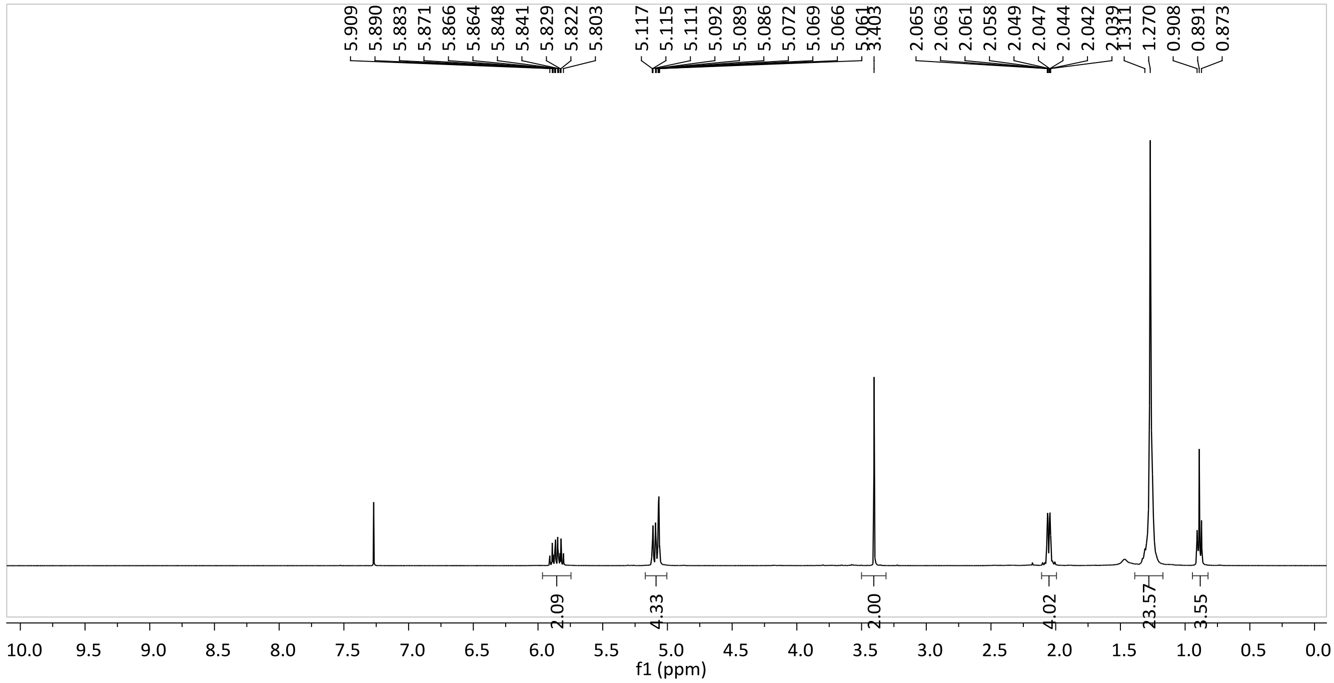
5.909
5.890
5.883
5.871
5.866
5.864
5.848
5.841
5.829
5.822
5.803
5.117
5.115
5.111
5.092
5.089
5.086
5.072
5.069
5.066
5.061
3.403
2.065
2.063
2.061
2.058
2.049
2.047
2.044
2.042
2.039
1.311
1.270
0.908
0.891
0.873
2.09
4.33
2.00
4.02
23.57
3.55
10.0 9.5 9.0 8.5 8.0 7.5 7.0 6.5 6.0 5.5 5.0 4.5 4.0 3.5 3.0 2.5 2.0 1.5 1.0 0.5 0.0
f1 (ppm)

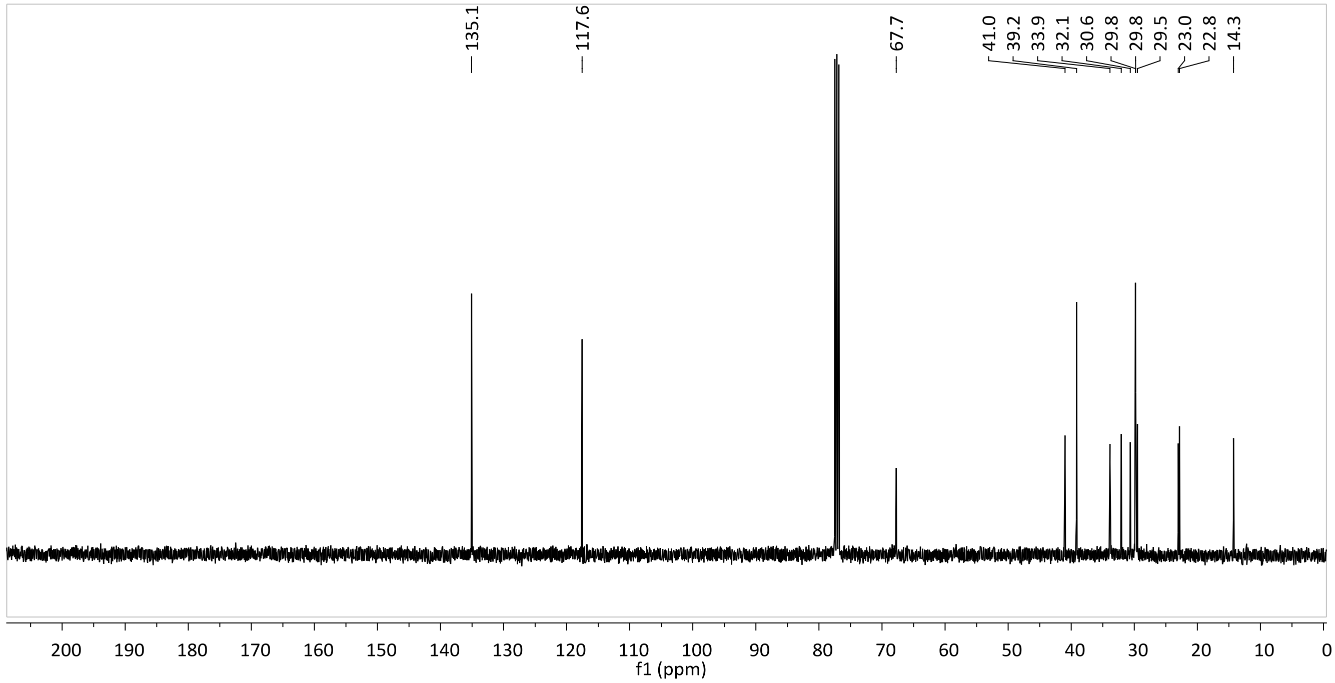
135.1
117.6
67.7
41.0
39.2
33.9
32.1
30.6
29.8
29.8
29.5
23.0
22.8
14.3
200 190 180 170 160 150 140 130 120 110 100 90 80 70 60 50 40 30 20 10 0
f1 (ppm)

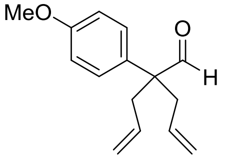
MeO
O
H

(**1b**)

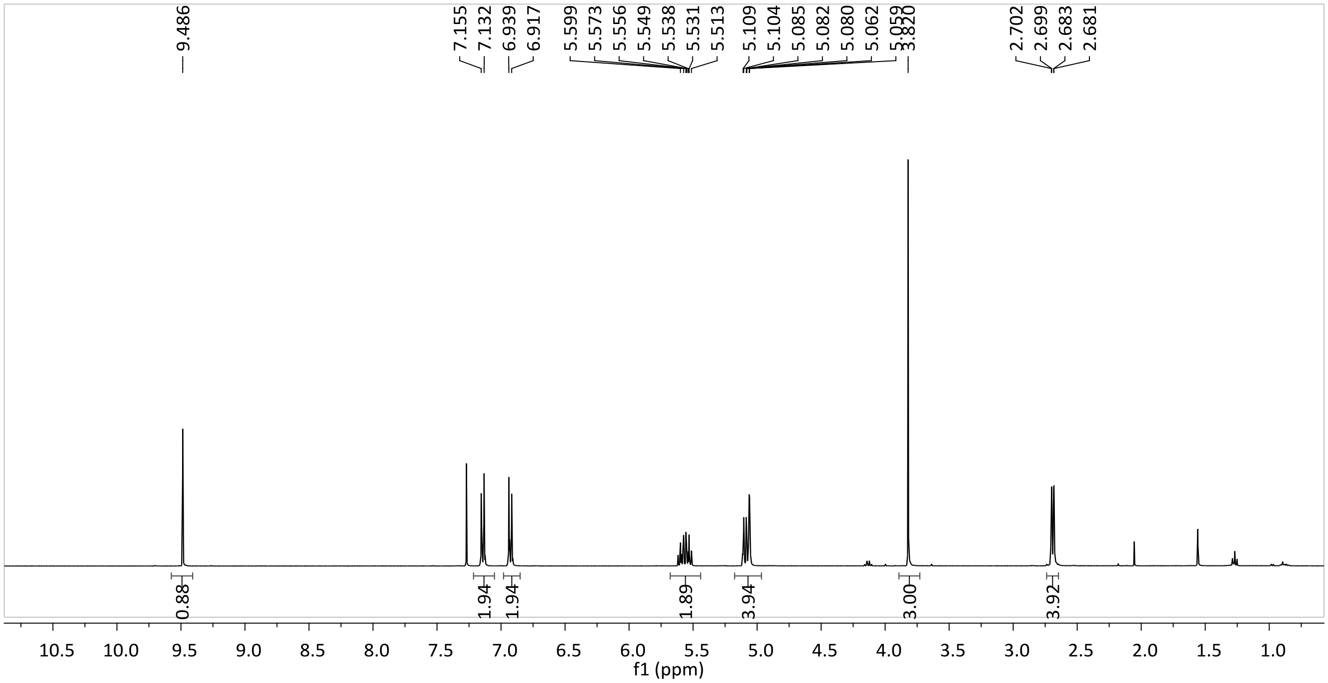
9.486
7.155
7.132
6.939
6.917
5.599
5.573
5.556
5.549
5.538
5.531
5.513
5.109
5.104
5.085
5.082
5.080
5.062
5.059
3.820
2.702
2.699
2.683
2.681
0.88
1.94
1.94
1.89
3.94
3.00
3.92
10.5 10.0 9.5 9.0 8.5 8.0 7.5 7.0 6.5 6.0 5.5 5.0 4.5 4.0 3.5 3.0 2.5 2.0 1.5 1.0
f1 (ppm)

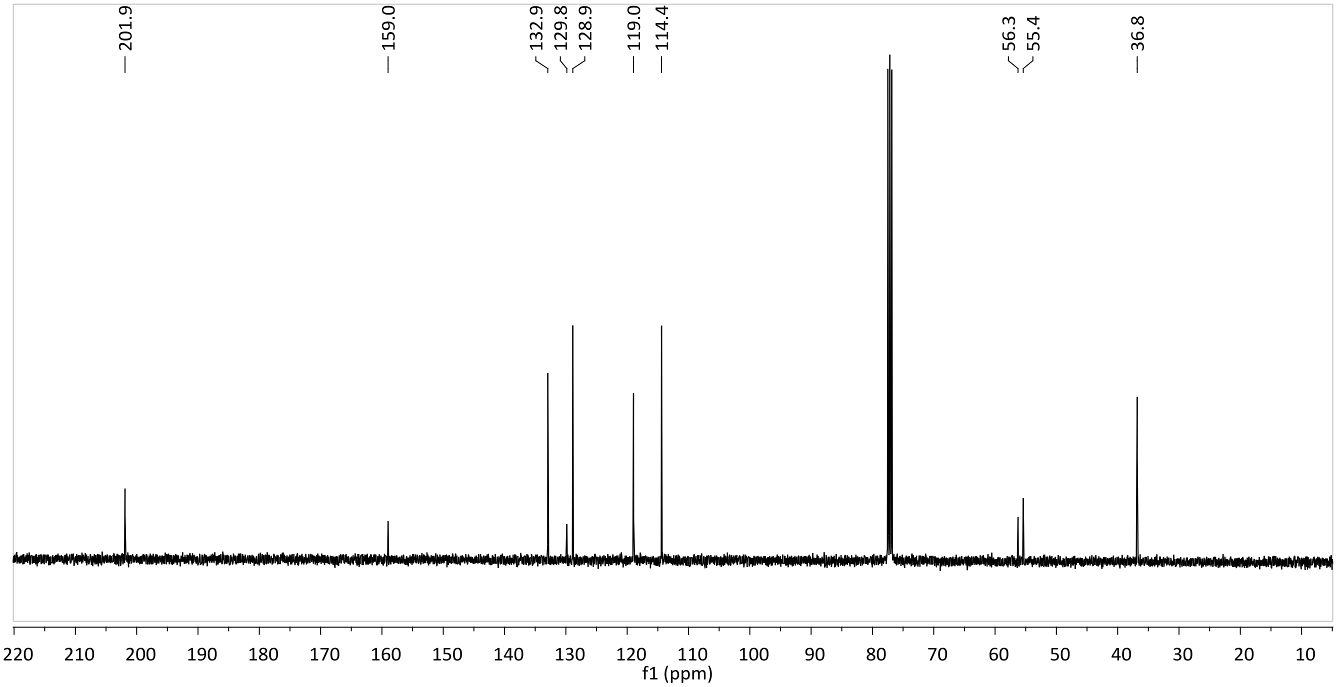
201.9
159.0
132.9
129.8
128.9
119.0
114.4
56.3
55.4
36.8
220 210 200 190 180 170 160 150 140 130 120 110 100 90 80 70 60 50 40 30 20 10
f1 (ppm)

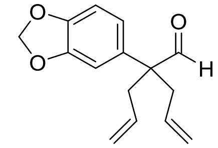
O
O
O
H

(**1c**)

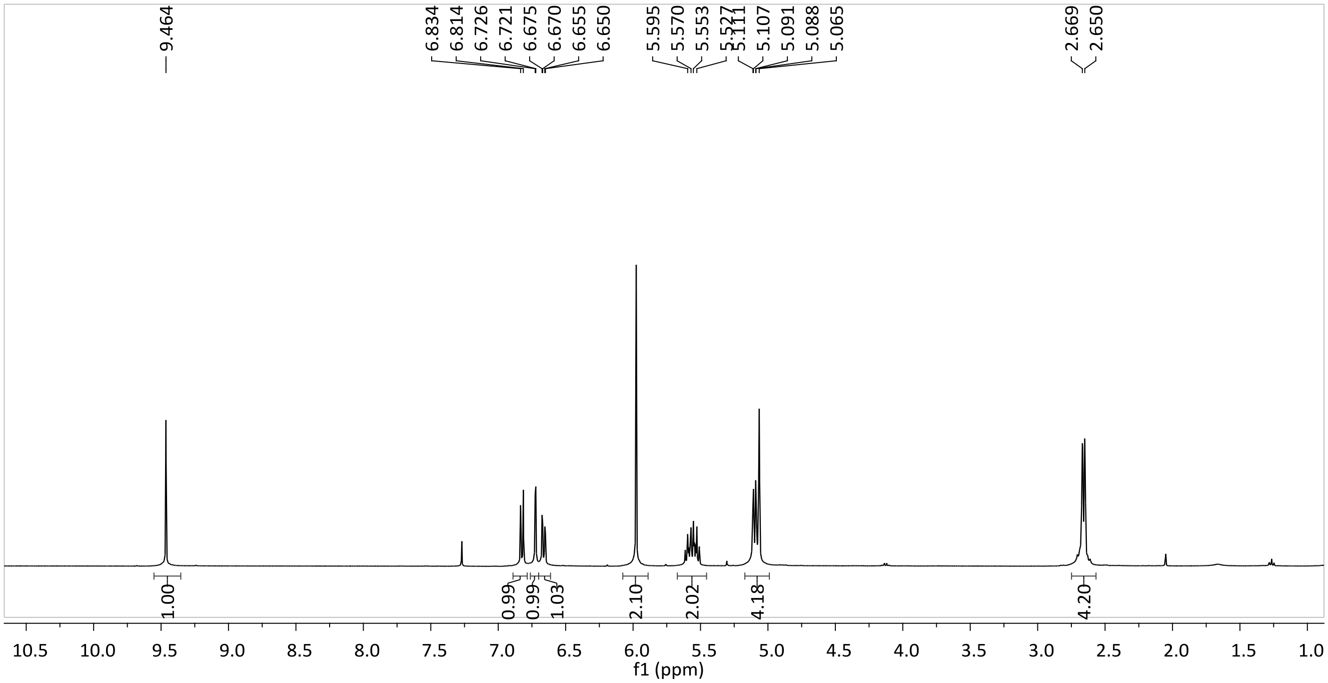
9.464
6.834
6.814
6.726
6.721
6.675
6.670
6.655
6.650
5.595
5.570
5.553
5.527
5.111
5.107
5.091
5.088
5.065
2.669
2.650
1.00
0.99
0.99
1.03
2.10
2.02
4.18
4.20
10.5 10.0 9.5 9.0 8.5 8.0 7.5 7.0 6.5 6.0 5.5 5.0 4.5 4.0 3.5 3.0 2.5 2.0 1.5 1.0
f1 (ppm)

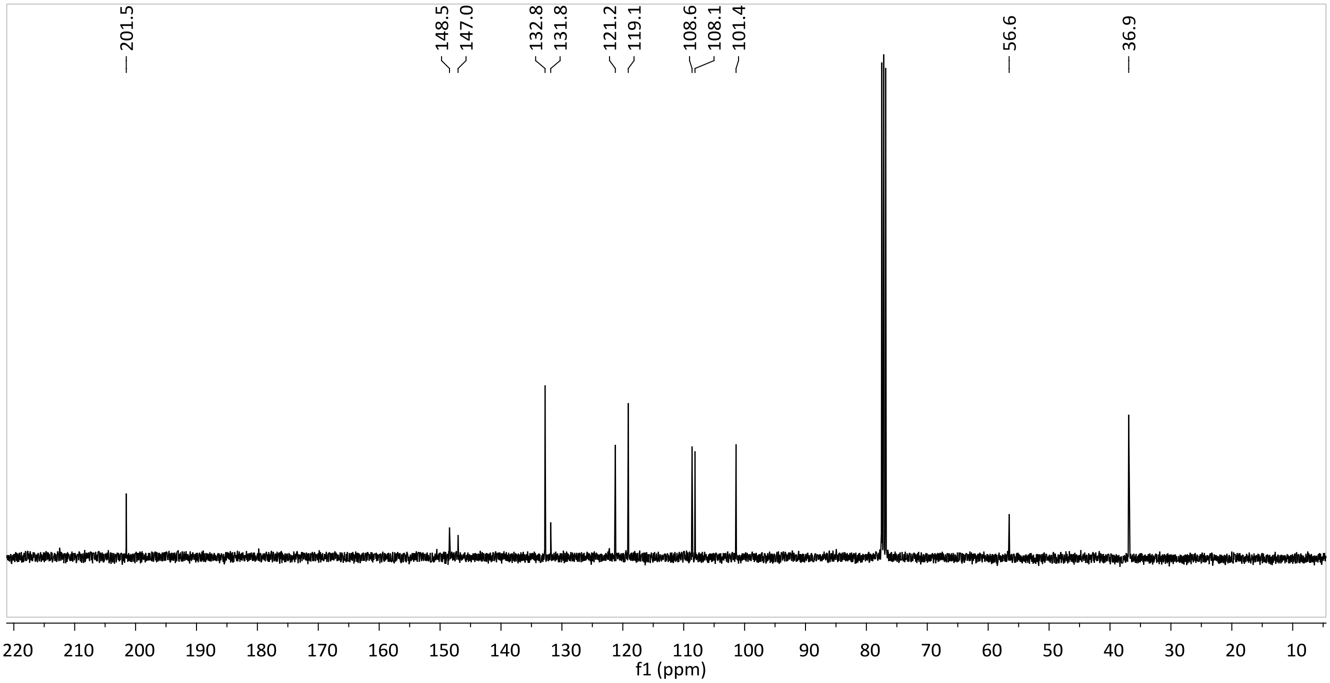
201.5
148.5
147.0
132.8
131.8
121.2
119.1
108.6
108.1
101.4
56.6
36.9
220 210 200 190 180 170 160 150 140 130 120 110 100 90 80 70 60 50 40 30 20 10
f1 (ppm)

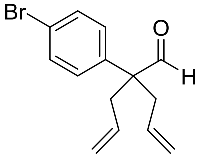
Br
O
H

(**1d**)

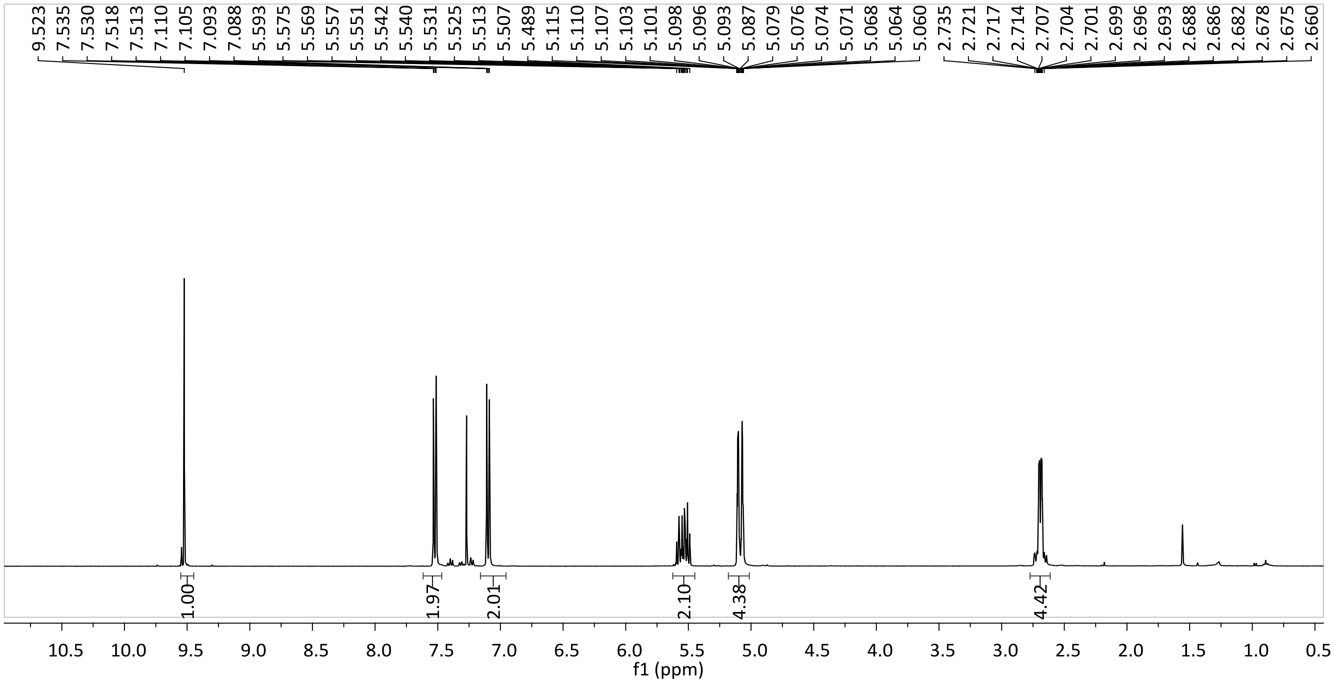
9.523
7.535
7.530
7.518
7.513
7.110
7.105
7.093
7.088
5.593
5.575
5.569
5.557
5.551
5.542
5.540
5.531
5.525
5.513
5.507
5.489
5.115
5.110
5.107
5.103
5.101
5.098
5.096
5.093
5.087
5.079
5.076
5.074
5.071
5.068
5.064
5.060
2.735
2.721
2.717
2.714
2.707
2.704
2.701
2.699
2.696
2.693
2.688
2.686
2.682
2.678
2.675
2.660
1.00
1.97
2.01
2.10
4.38
4.42
10.5 10.0 9.5 9.0 8.5 8.0 7.5 7.0 6.5 6.0 5.5 5.0 4.5 4.0 3.5 3.0 2.5 2.0 1.5 1.0 0.5
f1 (ppm)

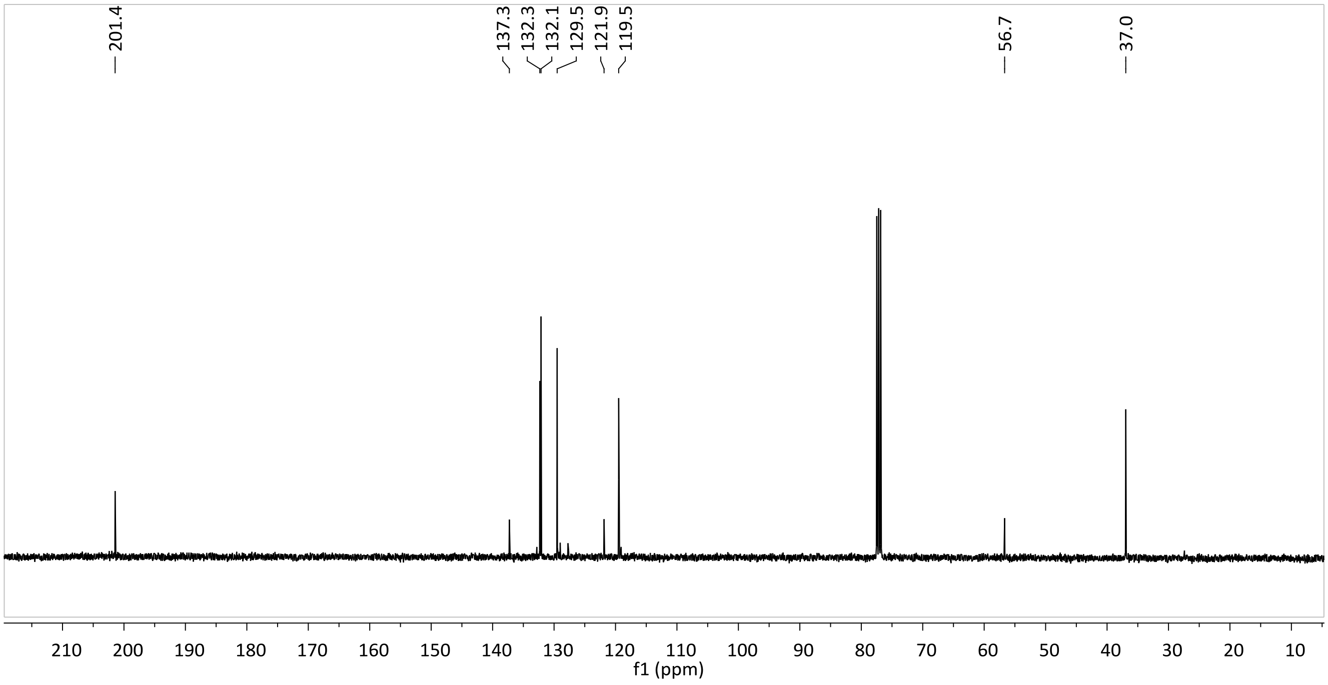
201.4
137.3
132.3
132.1
129.5
121.9
119.5
56.7
37.0
210 200 190 180 170 160 150 140 130 120 110 100 90 80 70 60 50 40 30 20 10
f1 (ppm)

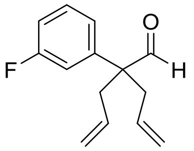
F
O
H

(**1e**)

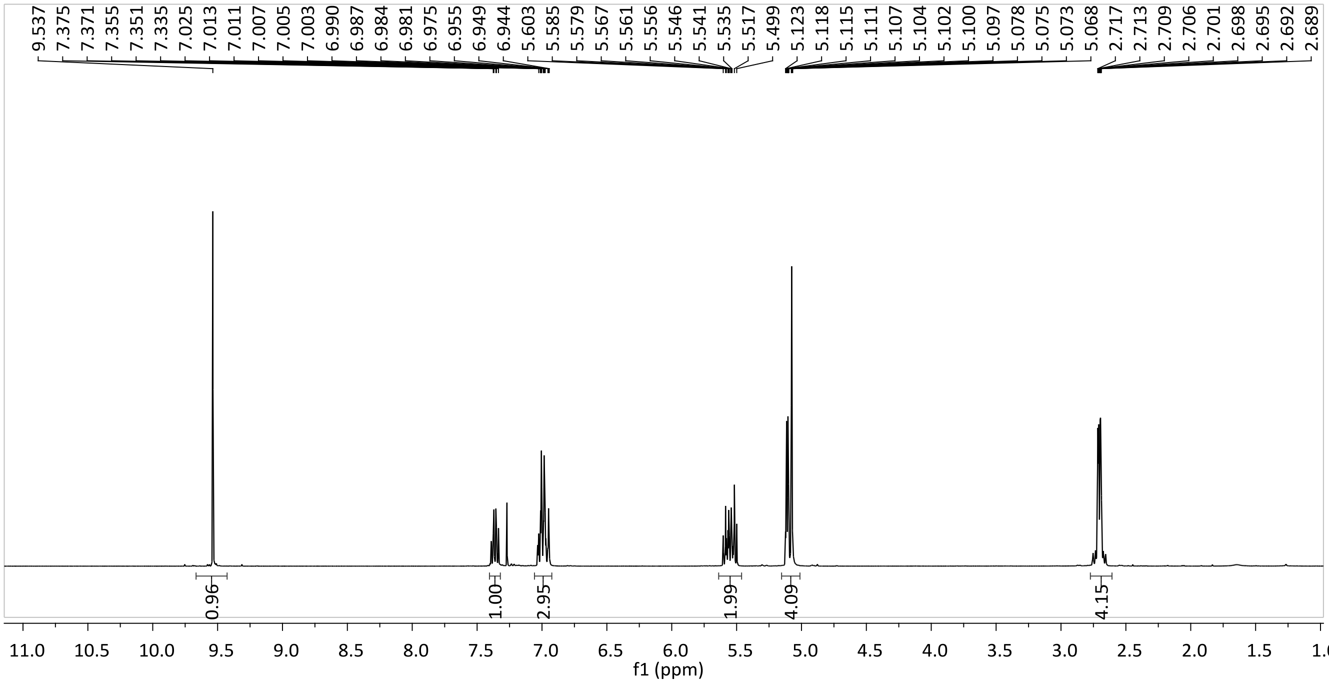
9.537
7.375
7.371
7.355
7.351
7.335
7.025
7.013
7.011
7.007
7.005
7.003
6.990
6.987
6.984
6.981
6.975
6.955
6.949
6.944
5.603
5.585
5.579
5.567
5.561
5.556
5.546
5.541
5.535
5.517
5.499
5.123
5.118
5.115
5.111
5.107
5.104
5.102
5.100
5.097
5.078
5.075
5.073
5.068
2.717
2.713
2.709
2.706
2.701
2.698
2.695
2.692
2.689
0.96
1.00
2.95
1.99
4.09
4.15
11.0 10.5 10.0 9.5 9.0 8.5 8.0 7.5 7.0 6.5 6.0 5.5 5.0 4.5 4.0 3.5 3.0 2.5 2.0 1.5 1.0
f1 (ppm)

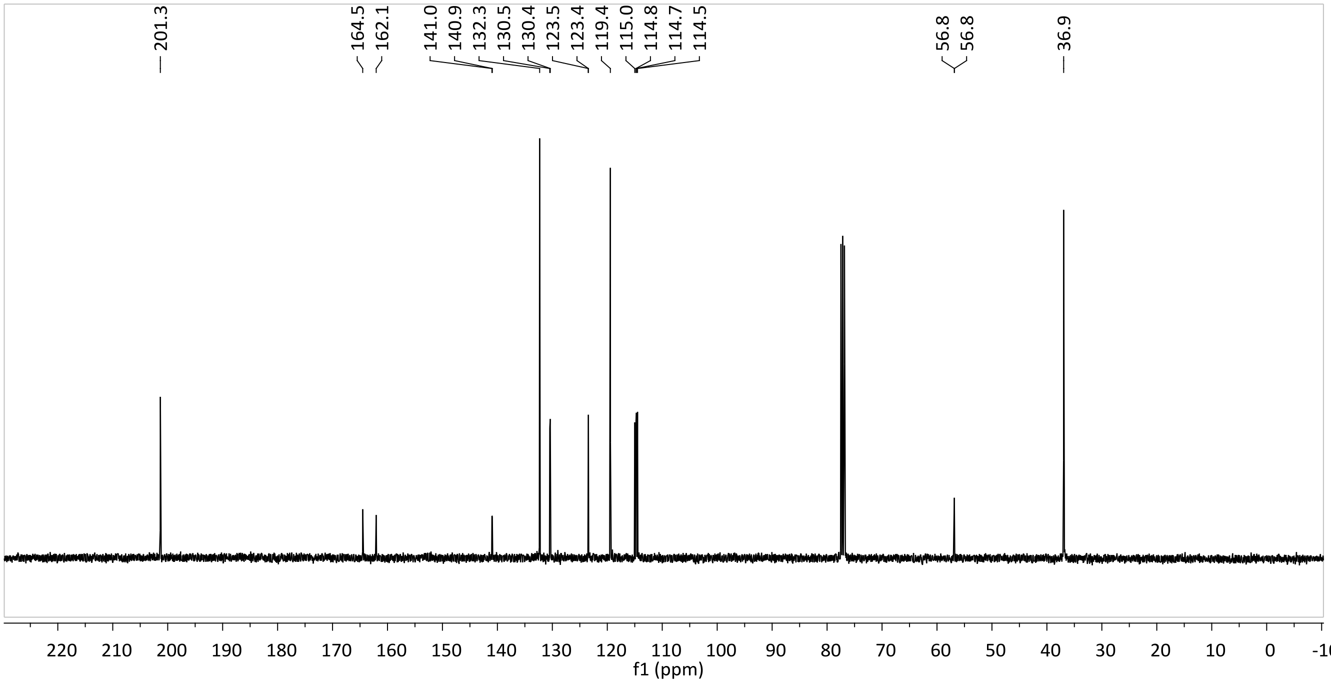
201.3
164.5
162.1
141.0
140.9
132.3
130.5
130.4
123.5
123.4
119.4
115.0
114.8
114.7
114.5
56.8
56.8
36.9
220 210 200 190 180 170 160 150 140 130 120 110 100 90 80 70 60 50 40 30 20 10 0 -10
f1 (ppm)

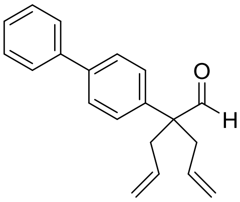
O
H

(**1f**)

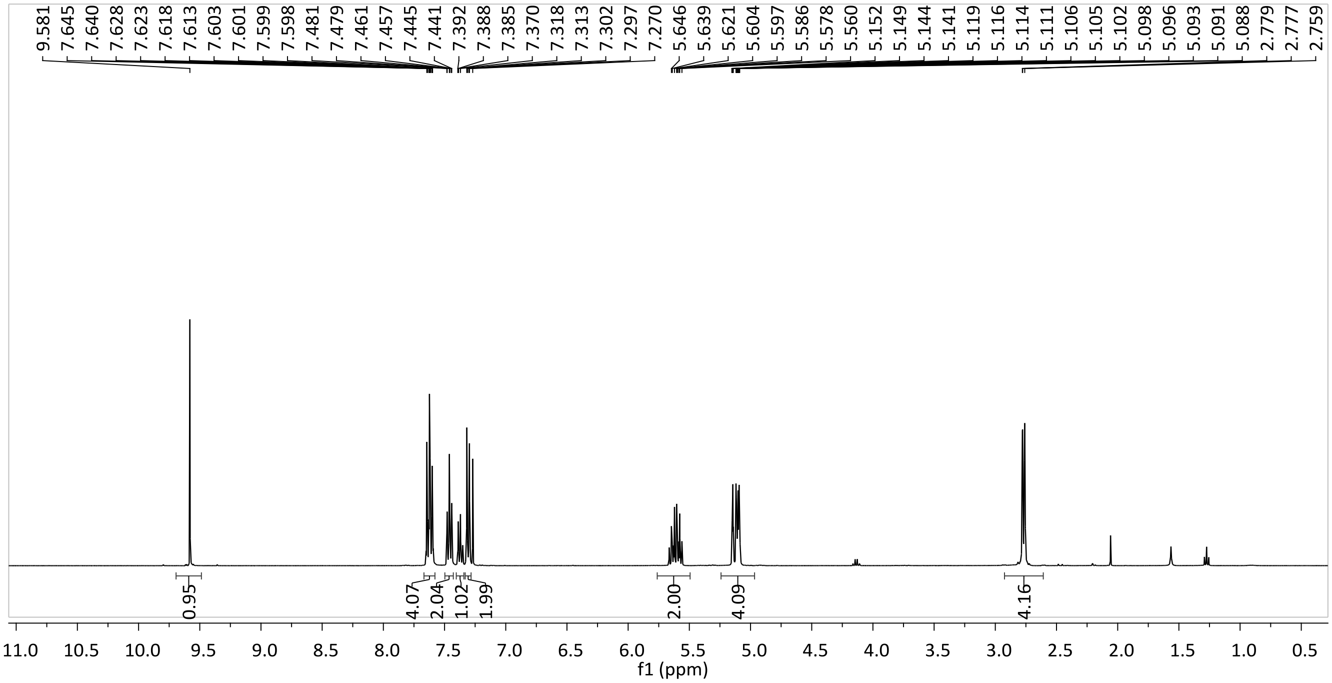
9.581
7.645
7.640
7.628
7.623
7.618
7.613
7.603
7.601
7.599
7.598
7.481
7.479
7.461
7.457
7.445
7.441
7.392
7.388
7.385
7.370
7.318
7.313
7.302
7.297
7.270
5.646
5.639
5.621
5.604
5.597
5.586
5.578
5.560
5.152
5.149
5.144
5.141
5.119
5.116
5.114
5.111
5.106
5.105
5.102
5.098
5.096
5.093
5.091
5.088
2.779
2.777
2.759
0.95
4.07
2.04
1.02
1.99
2.00
4.09
4.16
11.0 10.5 10.0 9.5 9.0 8.5 8.0 7.5 7.0 6.5 6.0 5.5 5.0 4.5 4.0 3.5 3.0 2.5 2.0 1.5 1.0 0.5
f1 (ppm)

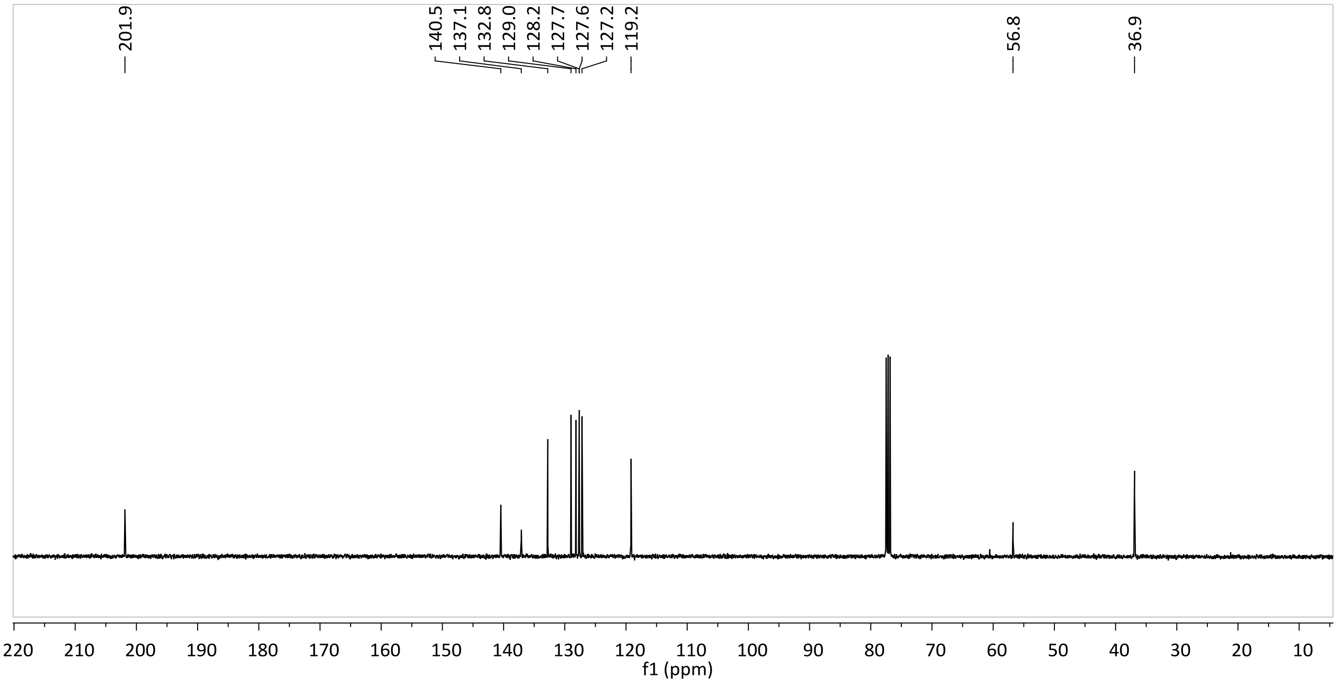
201.9
140.5
137.1
132.8
129.0
128.2
127.7
127.6
127.2
119.2
56.8
36.9
220 210 200 190 180 170 160 150 140 130 120 110 100 90 80 70 60 50 40 30 20 10
f1 (ppm)

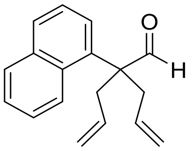
O
H

(1g)

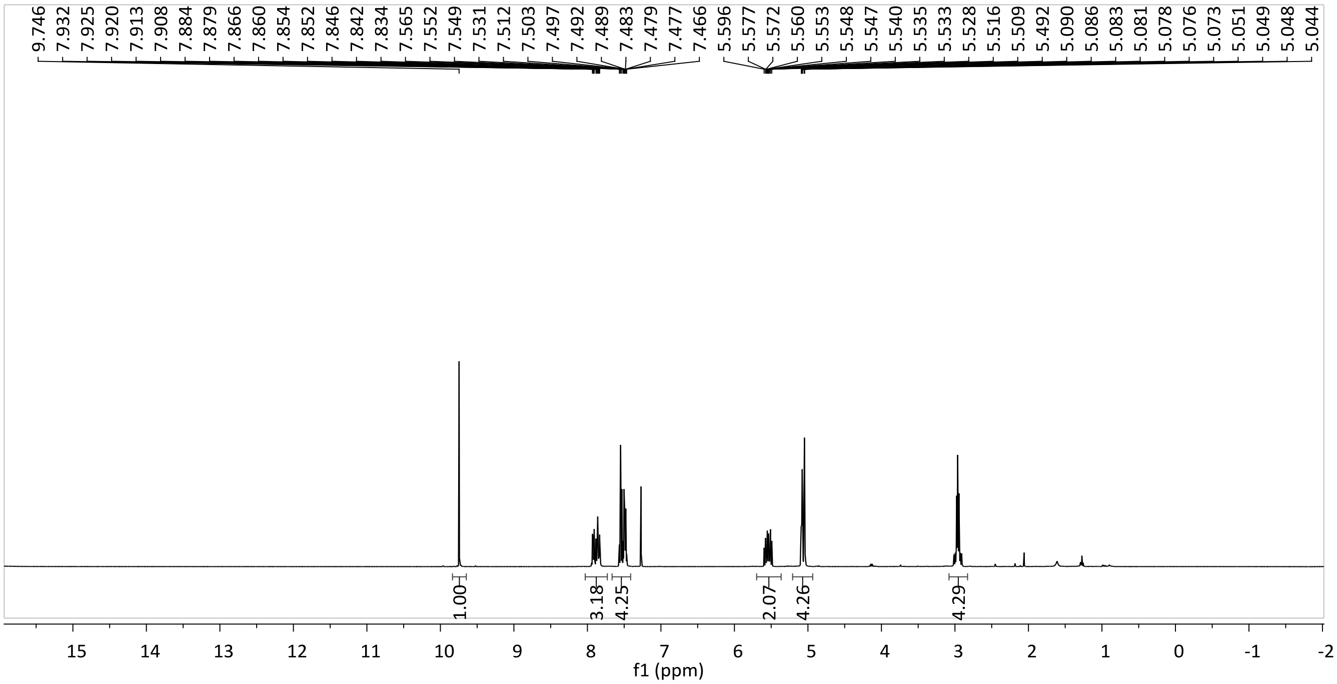
9.746
7.932
7.925
7.920
7.913
7.908
7.884
7.879
7.866
7.860
7.854
7.852
7.846
7.842
7.834
7.565
7.552
7.549
7.531
7.512
7.503
7.497
7.492
7.489
7.483
7.479
7.477
7.466
5.596
5.577
5.572
5.560
5.553
5.548
5.547
5.540
5.535
5.533
5.528
5.516
5.509
5.492
5.090
5.086
5.083
5.081
5.078
5.076
5.073
5.051
5.049
5.048
5.044
1.00
3.18
4.25
2.07
4.26
4.29
15 14 13 12 11 10 9 8 7 6 5 4 3 2 1 0 -1 -2
f1 (ppm)

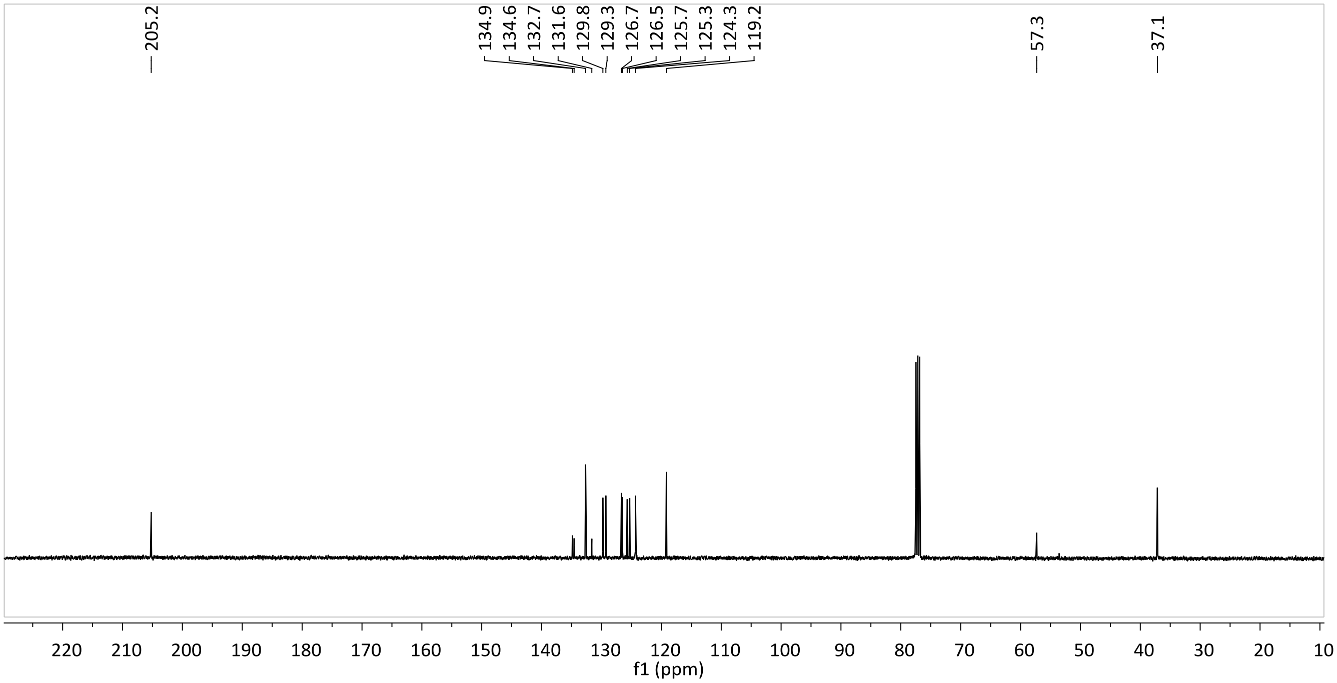
205.2
134.9
134.6
132.7
131.6
129.8
129.3
126.7
126.5
125.7
125.3
124.3
119.2
57.3
37.1
220 210 200 190 180 170 160 150 140 130 120 110 100 90 80 70 60 50 40 30 20 10
f1 (ppm)

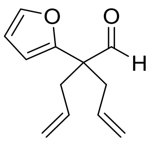

(**1h**)

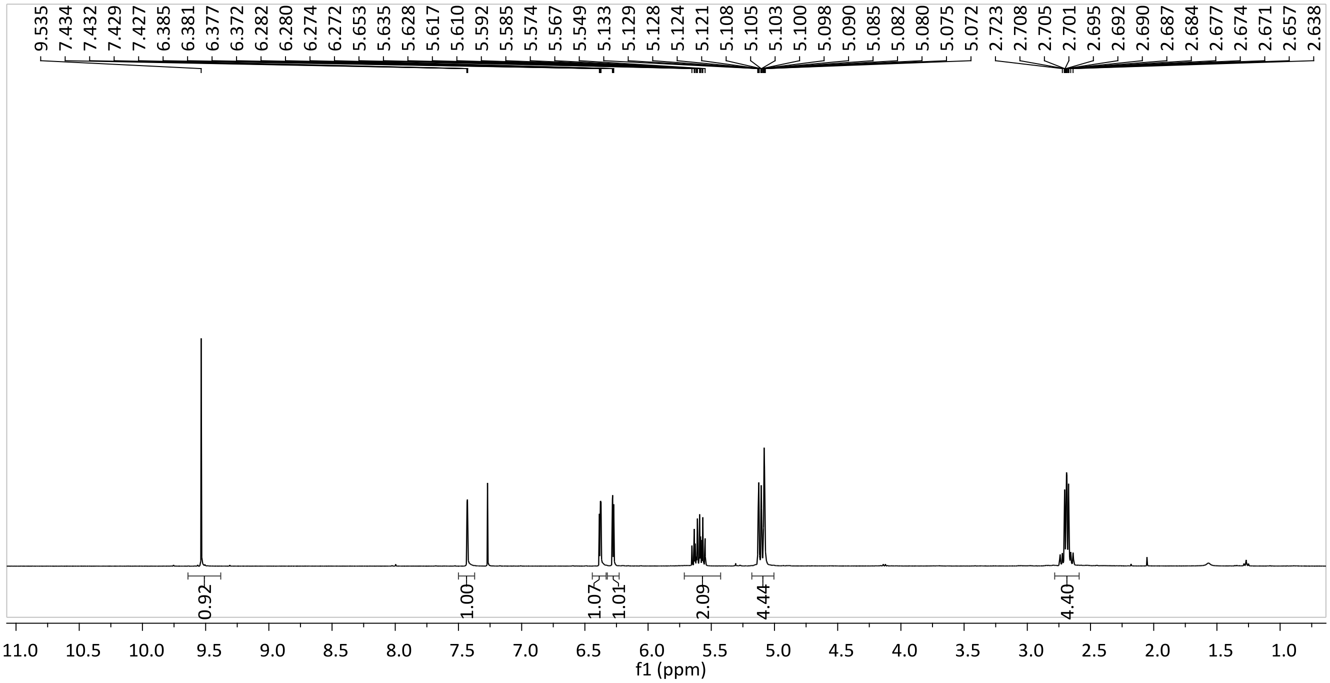
9.535
7.434
7.432
7.429
7.427
6.385
6.381
6.377
6.372
6.282
6.280
6.274
6.272
5.653
5.635
5.628
5.617
5.610
5.592
5.585
5.574
5.567
5.549
5.133
5.129
5.128
5.124
5.121
5.108
5.105
5.103
5.100
5.098
5.090
5.085
5.082
5.080
5.075
5.072
2.723
2.708
2.705
2.701
2.695
2.692
2.690
2.687
2.684
2.677
2.674
2.671
2.657
2.638
0.92
1.00
1.07
1.01
2.09
4.44
4.40
11.0 10.5 10.0 9.5 9.0 8.5 8.0 7.5 7.0 6.5 6.0 5.5 5.0 4.5 4.0 3.5 3.0 2.5 2.0 1.5 1.0
f1 (ppm)

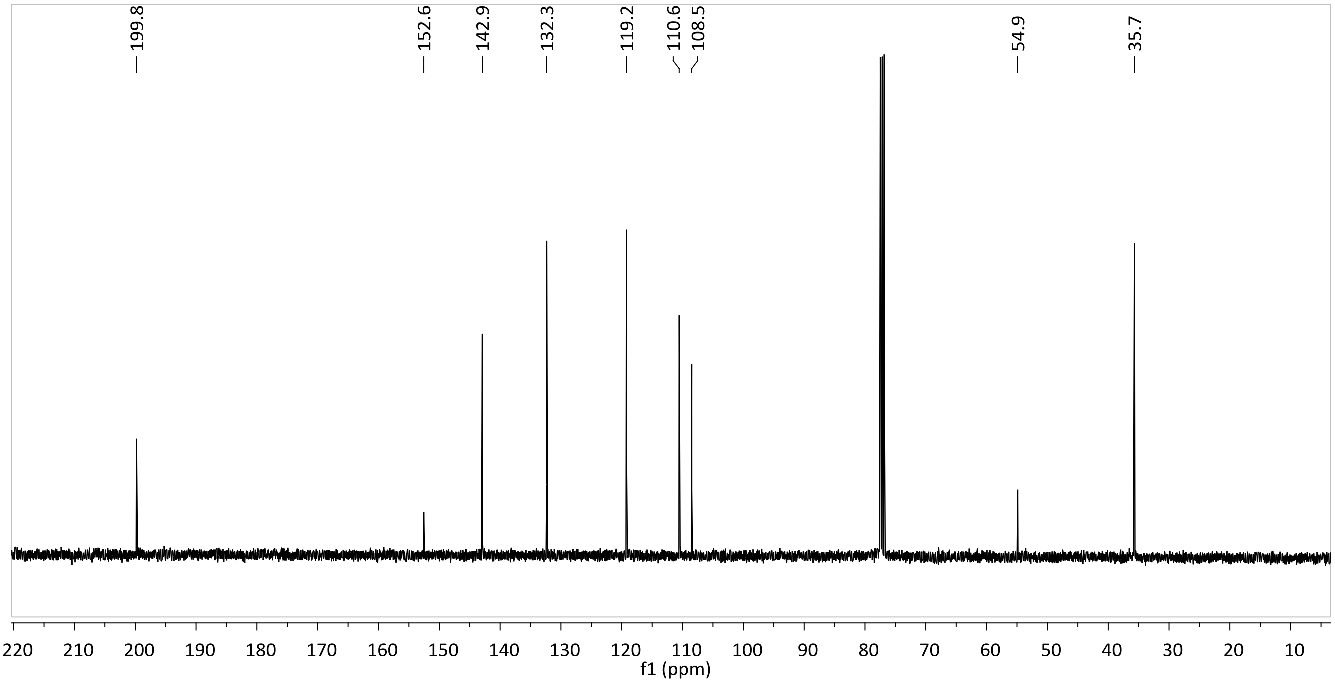
199.8
152.6
142.9
132.3
119.2
110.6
108.5
54.9
35.7
220 210 200 190 180 170 160 150 140 130 120 110 100 90 80 70 60 50 40 30 20 10
f1 (ppm)

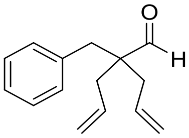

(**1i**)

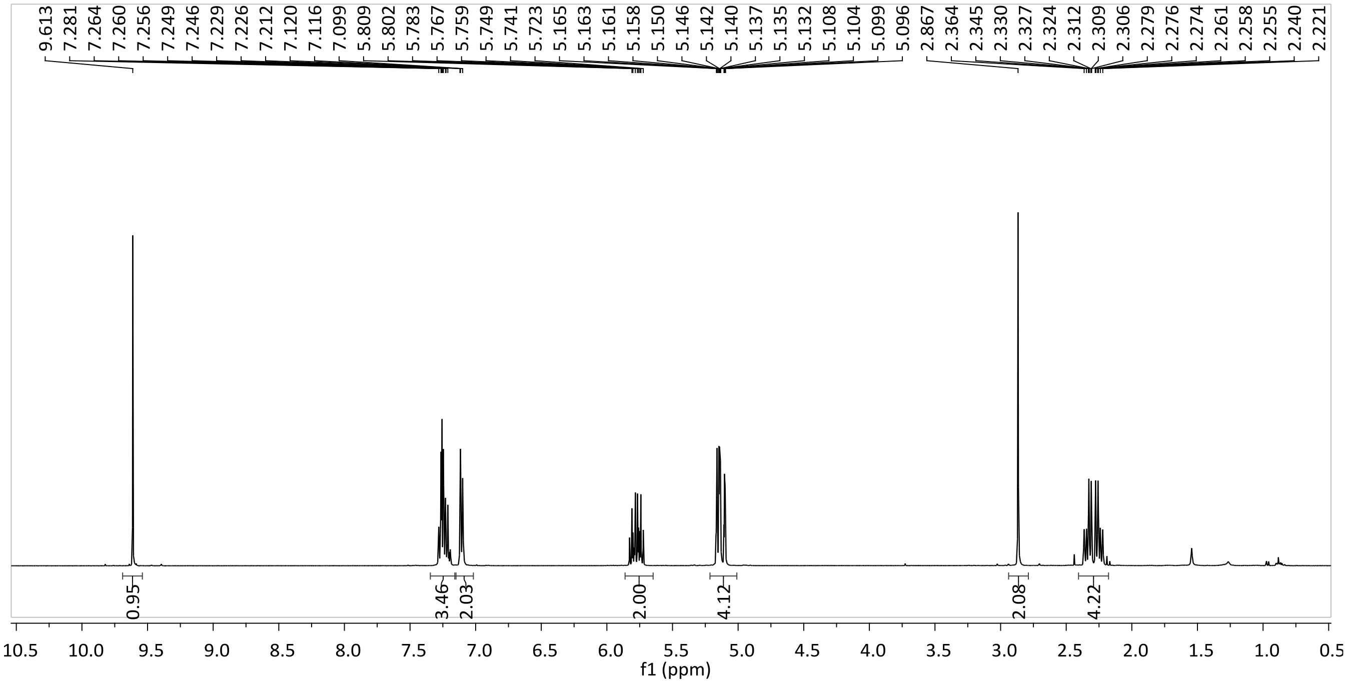

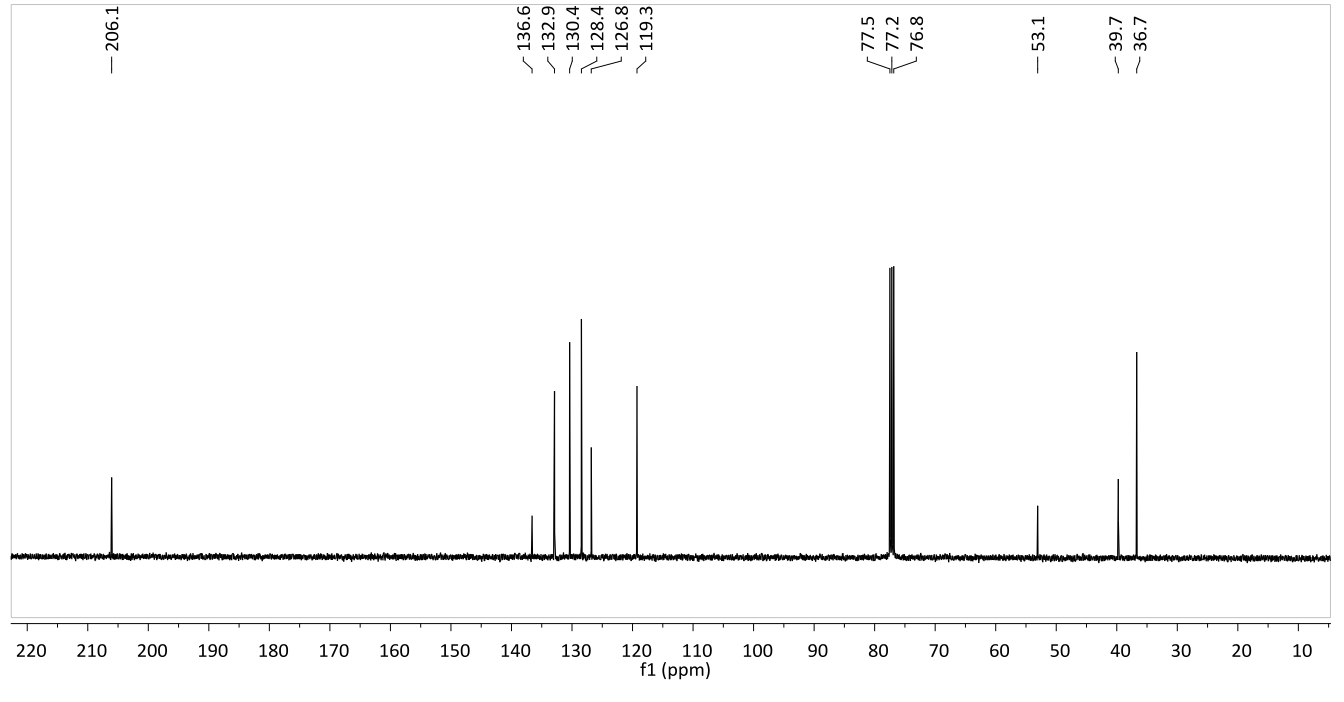

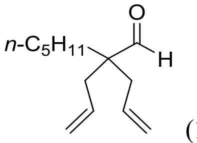

(**1j**)

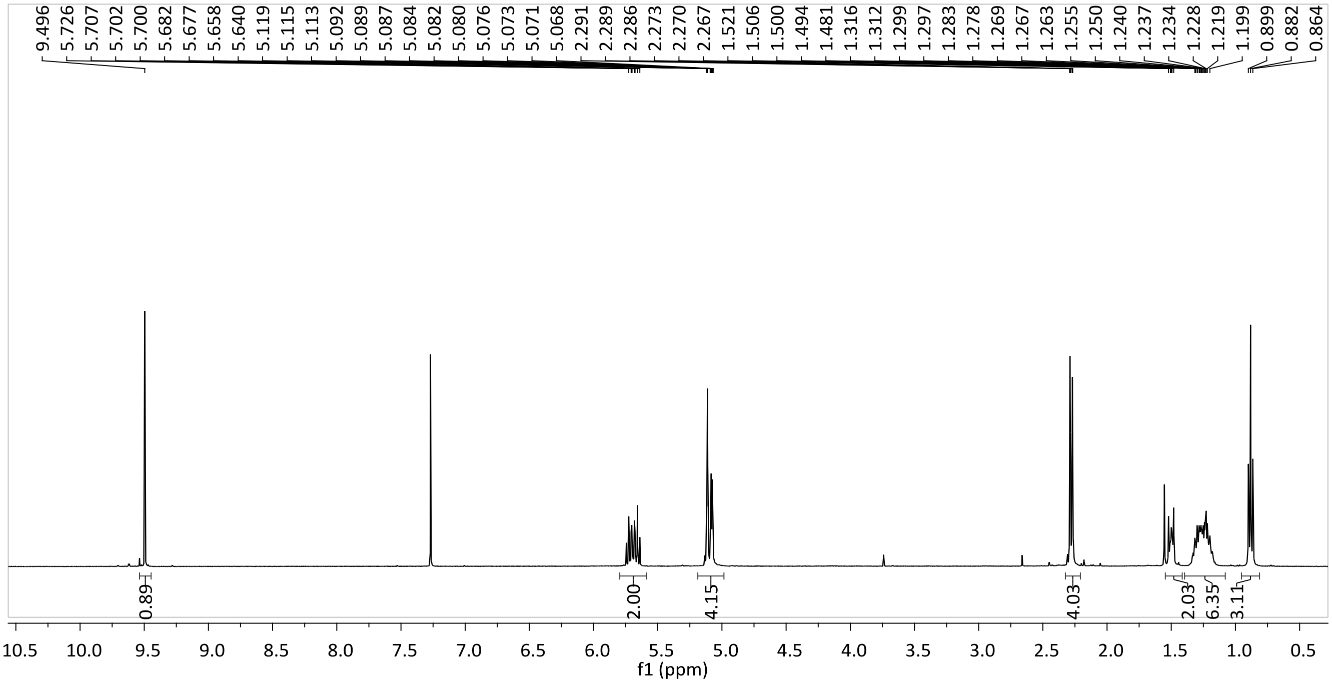

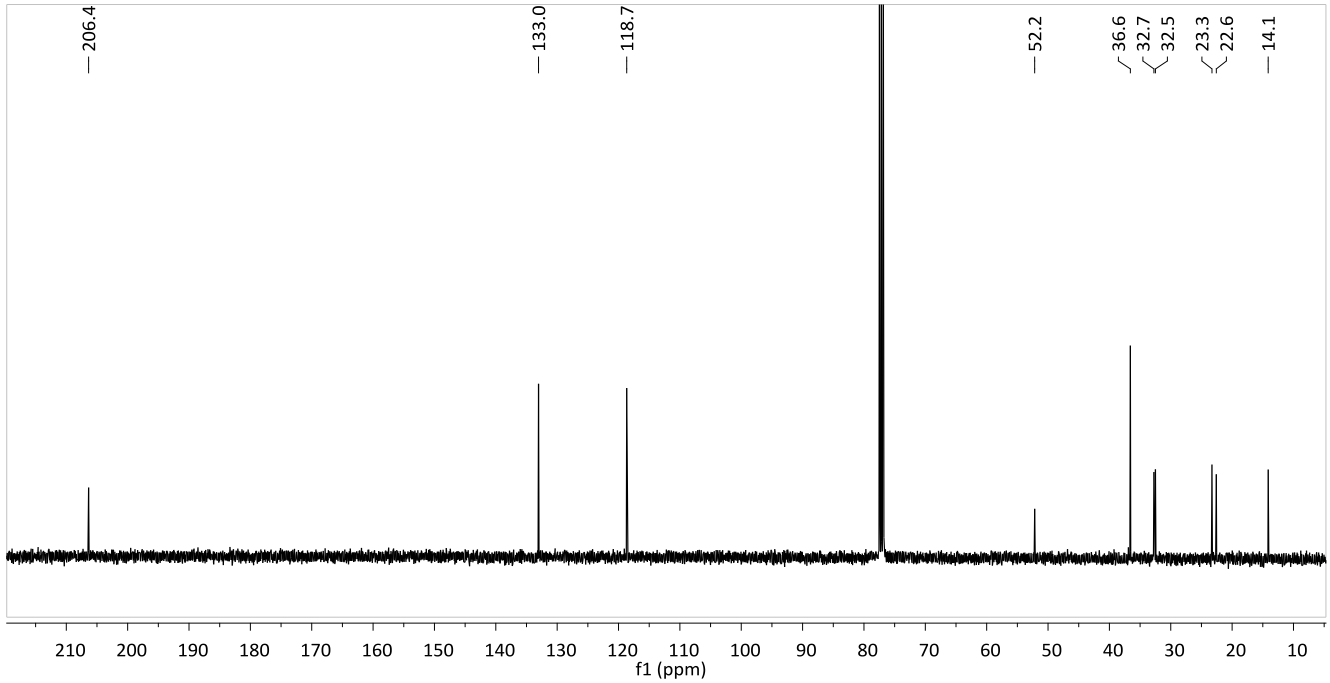

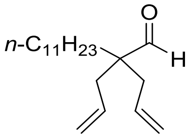

(**1k**)

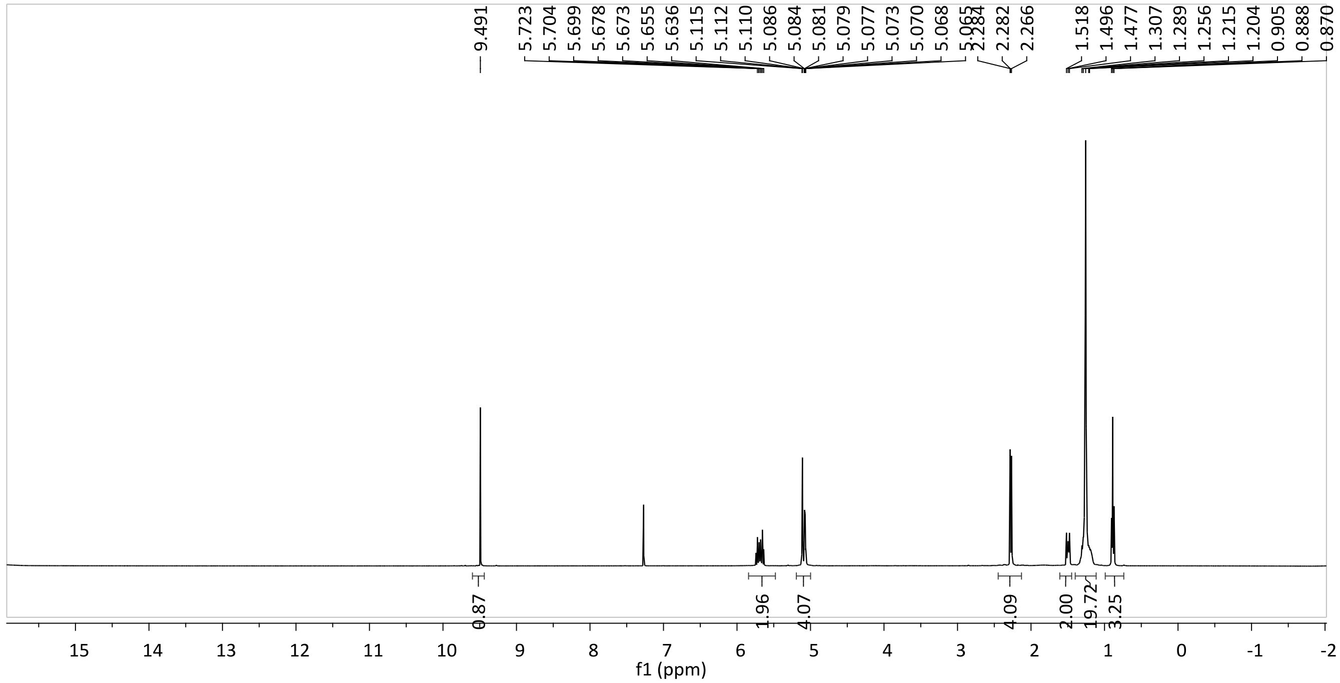

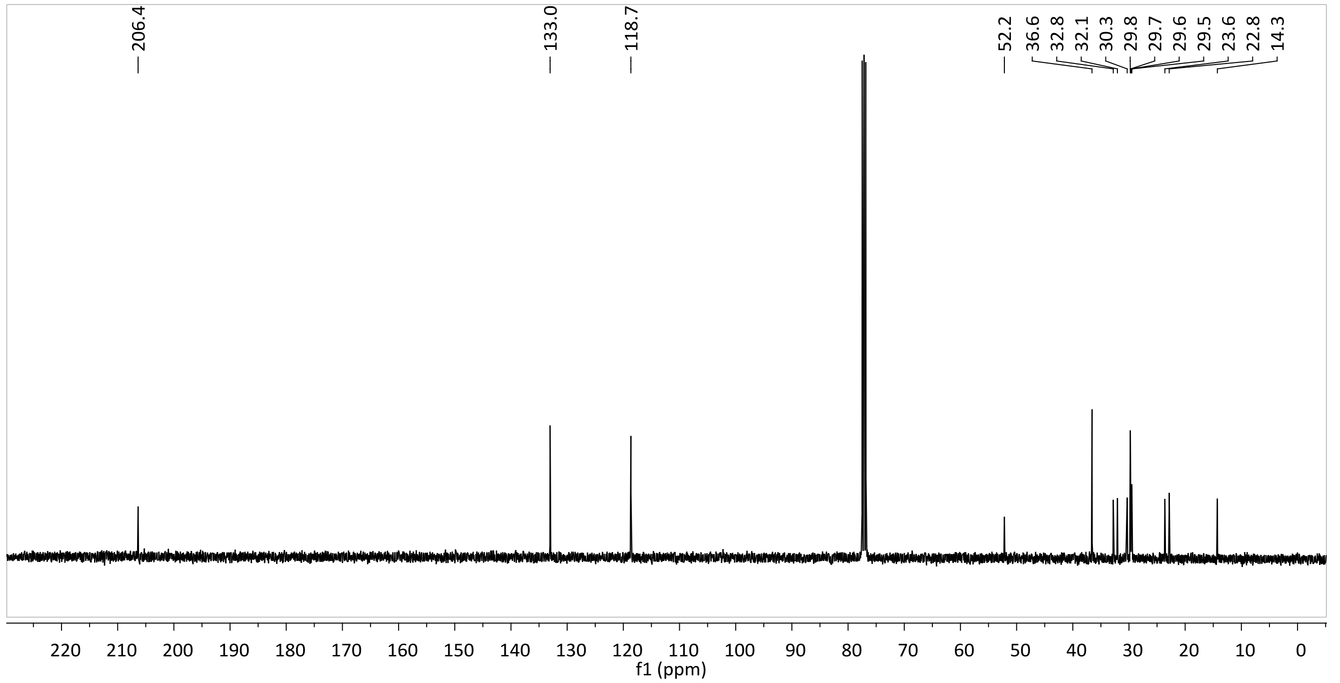

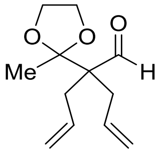
O
O
O
Me
H

(**1l**)

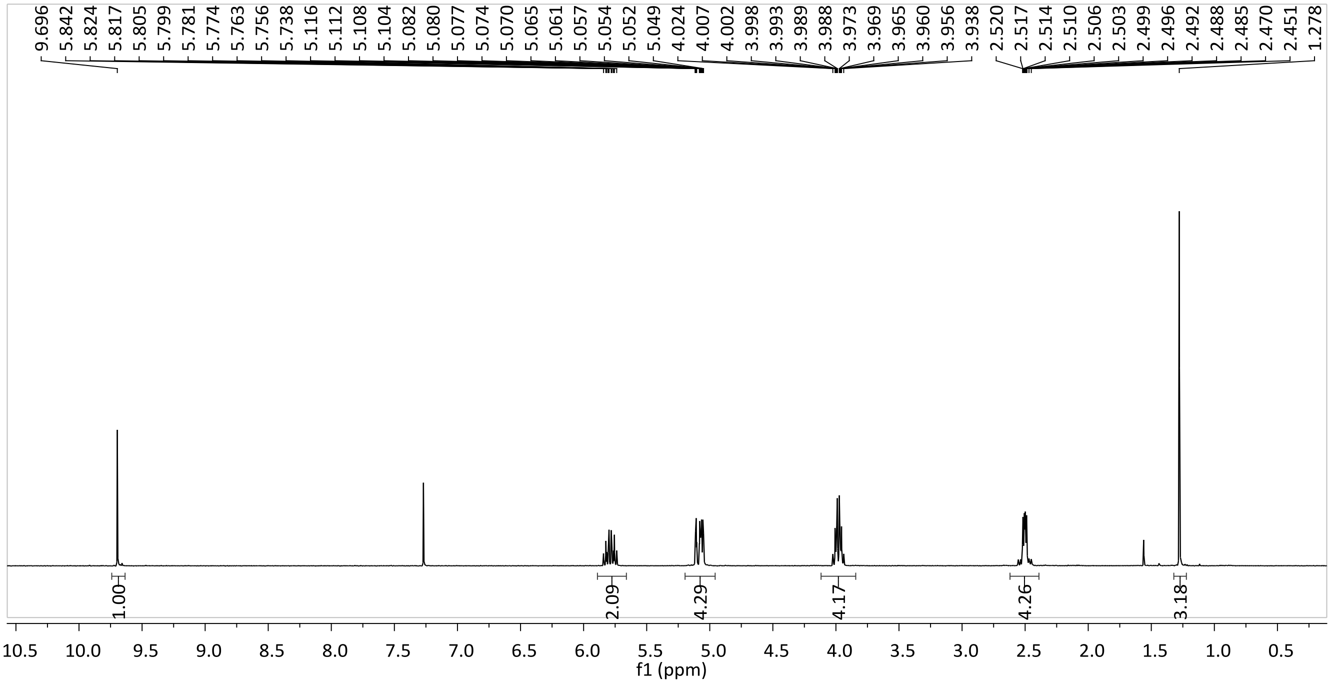
9.696 5.842 5.824 5.817 5.805 5.799 5.781 5.774 5.763 5.756 5.738 5.116 5.112 5.108 5.104 5.082 5.080 5.077 5.074 5.070 5.065 5.061 5.057 5.054 5.052 5.049 4.024 4.007 4.002 3.998 3.993 3.989 3.988 3.973 3.969 3.965 3.960 3.956 3.938 2.520 2.517 2.514 2.510 2.506 2.503 2.499 2.496 2.492 2.488 2.485 2.470 2.451 1.278
1.00
2.09
4.29
4.17
4.26
3.18
10.5 10.0 9.5 9.0 8.5 8.0 7.5 7.0 6.5 6.0 5.5 5.0 4.5 4.0 3.5 3.0 2.5 2.0 1.5 1.0 0.5
f1 (ppm)

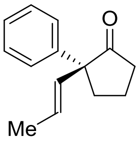
O
Me

(**2a**)

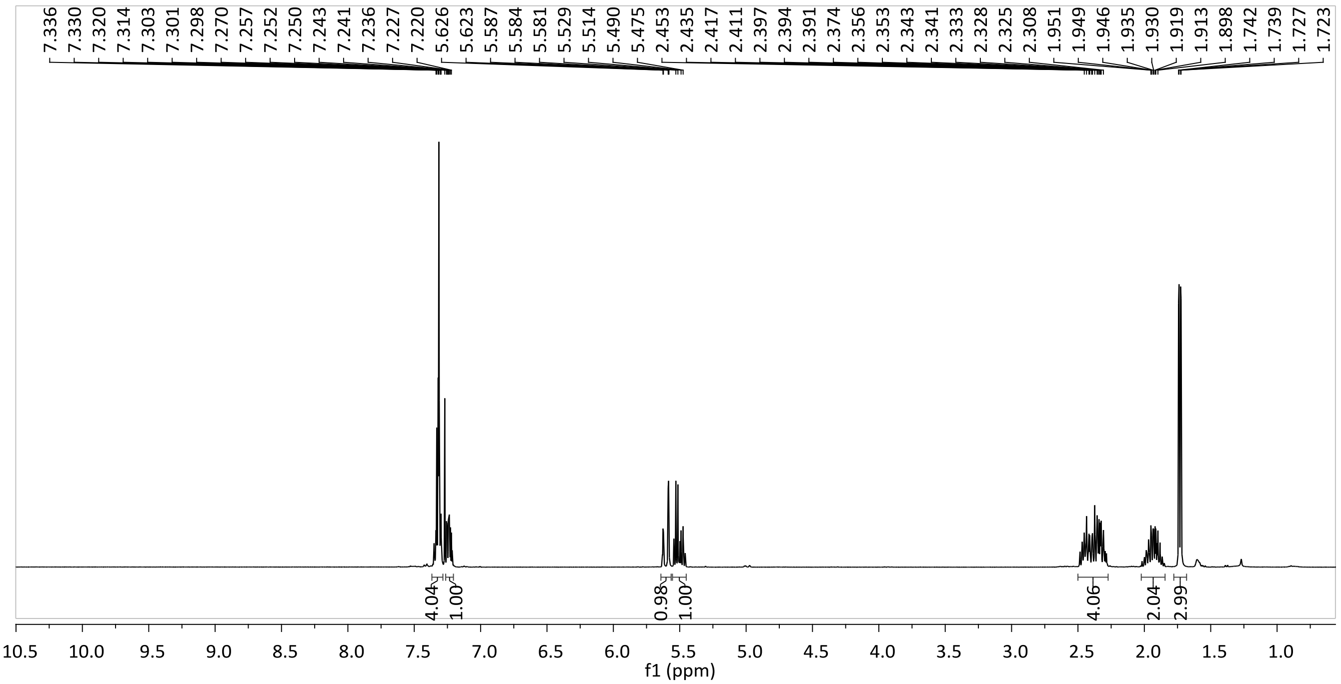
7.336
7.330
7.320
7.314
7.303
7.301
7.298
7.270
7.257
7.252
7.250
7.243
7.241
7.236
7.227
7.220
5.626
5.623
5.587
5.584
5.581
5.529
5.514
5.490
5.475
2.453
2.435
2.417
2.411
2.397
2.394
2.391
2.374
2.356
2.353
2.343
2.341
2.333
2.328
2.325
2.308
1.951
1.949
1.946
1.935
1.930
1.919
1.913
1.898
1.742
1.739
1.727
1.723
4.04
1.00
0.98
1.00
4.06
2.04
2.99
10.5
10.0
9.5
9.0
8.5
8.0
7.5
7.0
6.5
6.0
5.5
5.0
4.5
4.0
3.5
3.0
2.5
2.0
1.5
1.0
f1 (ppm)

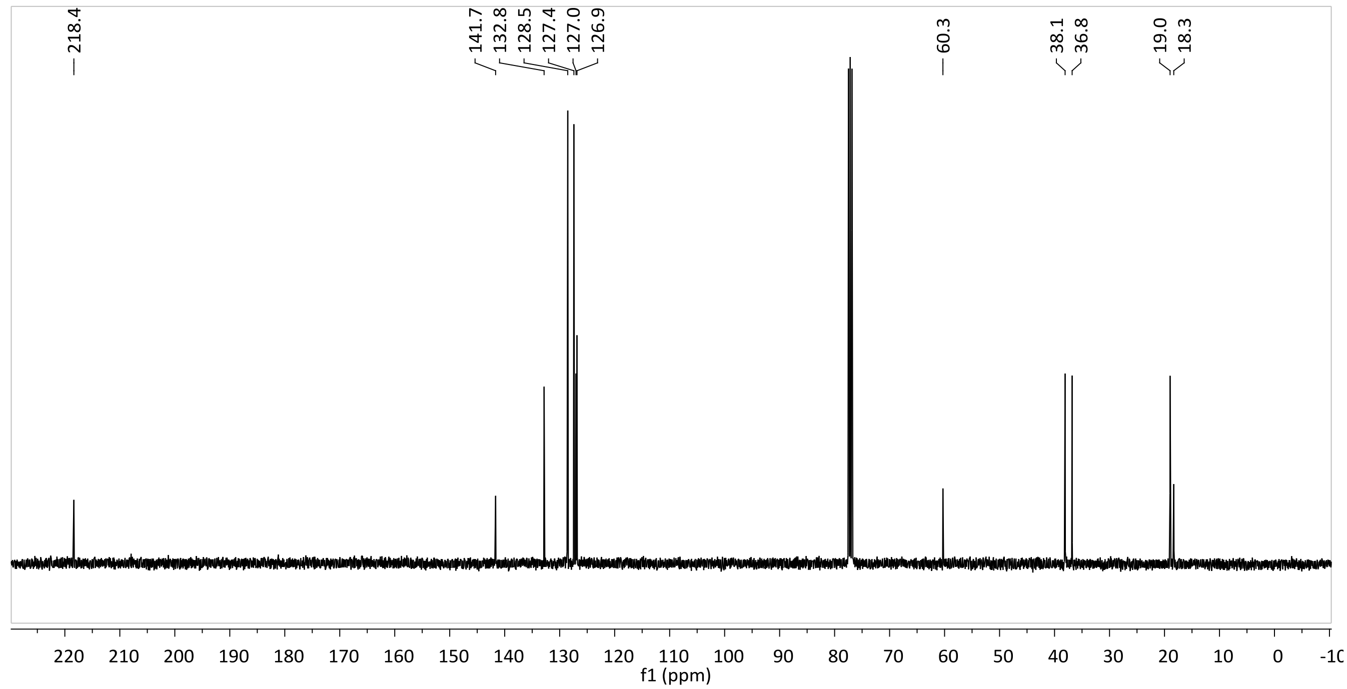
218.4
141.7
132.8
128.5
127.4
127.0
126.9
60.3
38.1
36.8
19.0
18.3
220
210
200
190
180
170
160
150
140
130
120
110
100
90
80
70
60
50
40
30
20
10
0
-10
f1 (ppm)

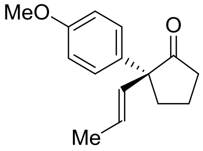
MeO
O
Me

(**2b**)

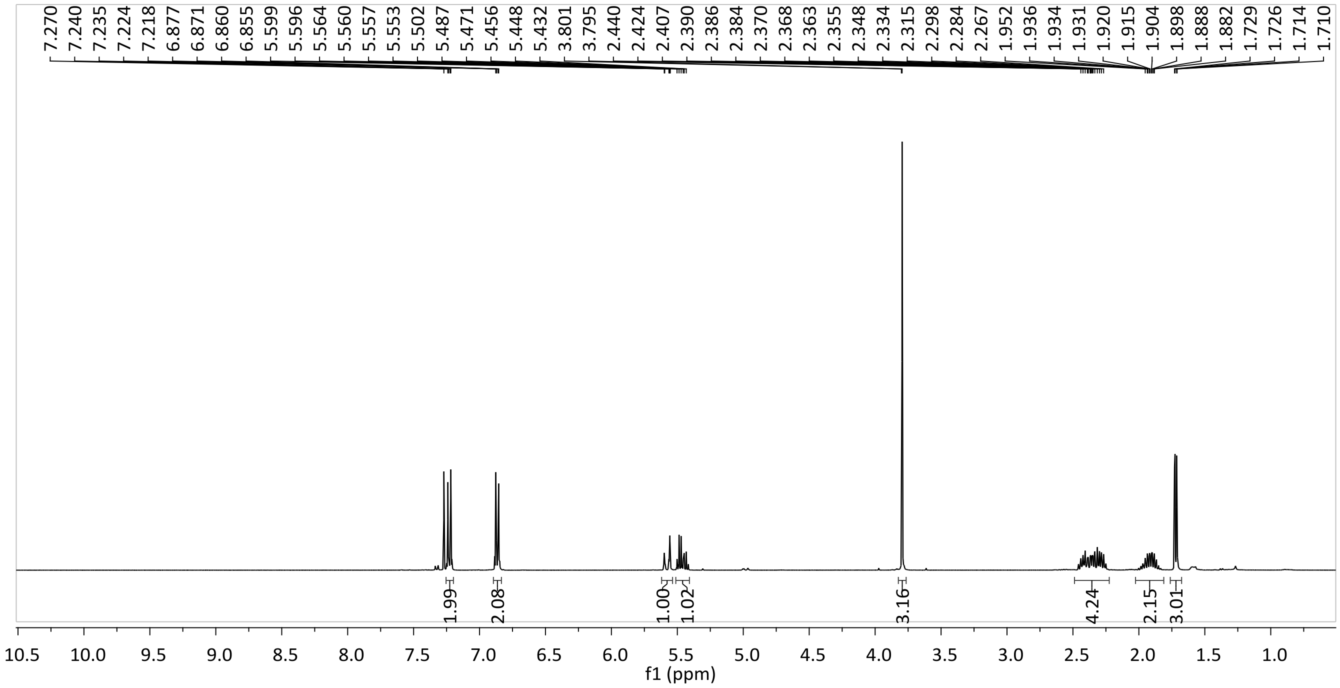
7.270 7.240 7.235 7.224 7.218 6.877 6.871 6.860 6.855 5.599 5.596 5.564 5.560 5.557 5.553 5.502 5.487 5.471 5.456 5.448 5.432 3.801 3.795 2.440 2.424 2.407 2.390 2.386 2.384 2.370 2.368 2.363 2.355 2.348 2.334 2.315 2.298 2.284 2.267 1.952 1.936 1.934 1.931 1.920 1.915 1.904 1.898 1.888 1.882 1.729 1.726 1.714 1.710
1.99
2.08
1.00
1.02
3.16
4.24
2.15
3.01
10.5 10.0 9.5 9.0 8.5 8.0 7.5 7.0 6.5 6.0 5.5 5.0 4.5 4.0 3.5 3.0 2.5 2.0 1.5 1.0
f1 (ppm)

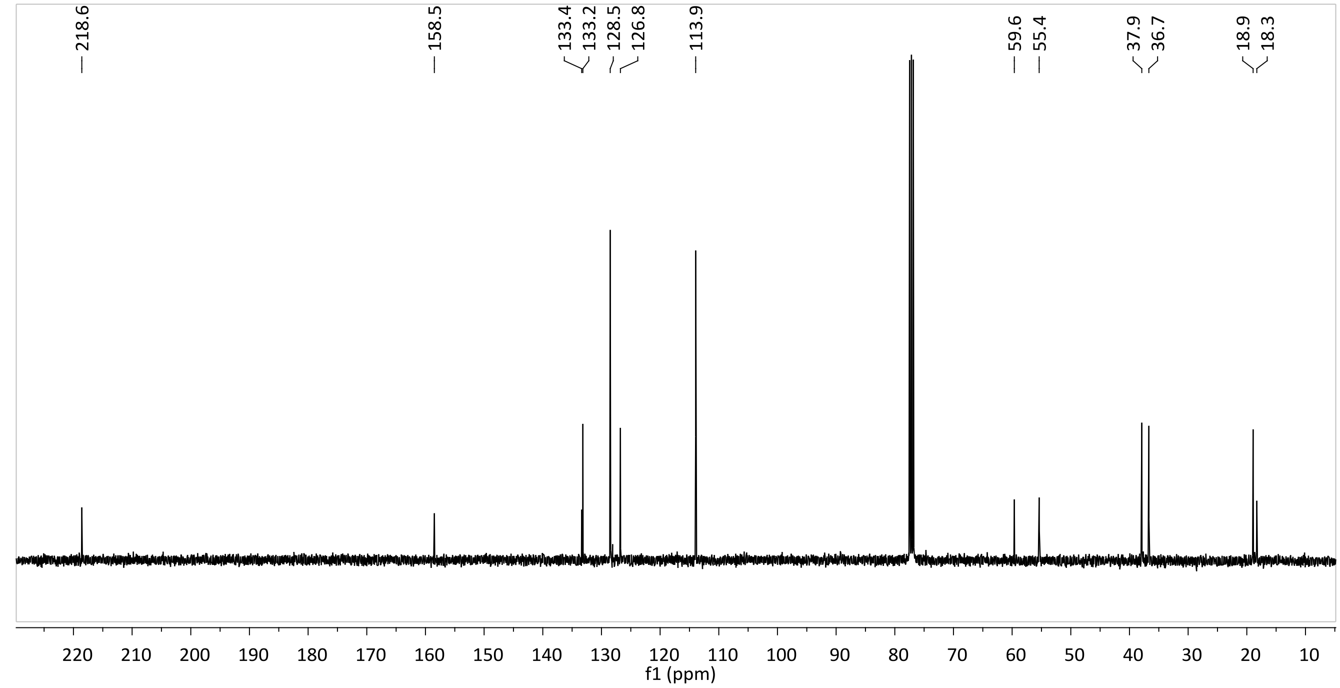
218.6
158.5
133.4 133.2 128.5 126.8
113.9
59.6 55.4
37.9 36.7
18.9 18.3
220 210 200 190 180 170 160 150 140 130 120 110 100 90 80 70 60 50 40 30 20 10
f1 (ppm)

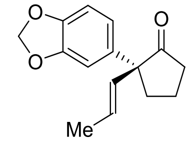
O
O
O
Me

(**2c**)

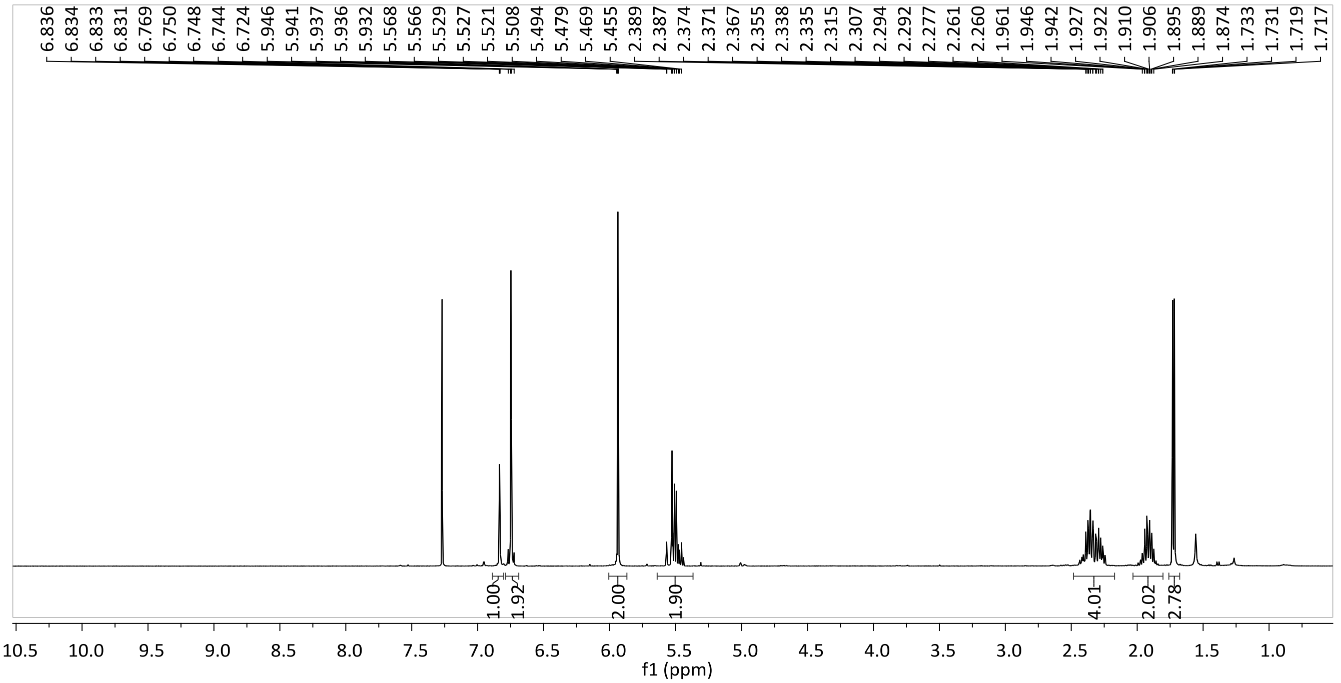
6.836
6.834
6.833
6.831
6.769
6.750
6.748
6.744
6.724
5.946
5.941
5.937
5.936
5.932
5.568
5.566
5.529
5.527
5.521
5.508
5.494
5.479
5.469
5.455
2.389
2.387
2.374
2.371
2.367
2.355
2.338
2.335
2.315
2.307
2.294
2.292
2.277
2.261
2.260
1.961
1.946
1.942
1.927
1.922
1.910
1.906
1.895
1.889
1.874
1.733
1.731
1.719
1.717
1.00
1.92
2.00
1.90
4.01
2.02
2.78
10.5
10.0
9.5
9.0
8.5
8.0
7.5
7.0
6.5
6.0
5.5
5.0
4.5
4.0
3.5
3.0
2.5
2.0
1.5
1.0
f1 (ppm)

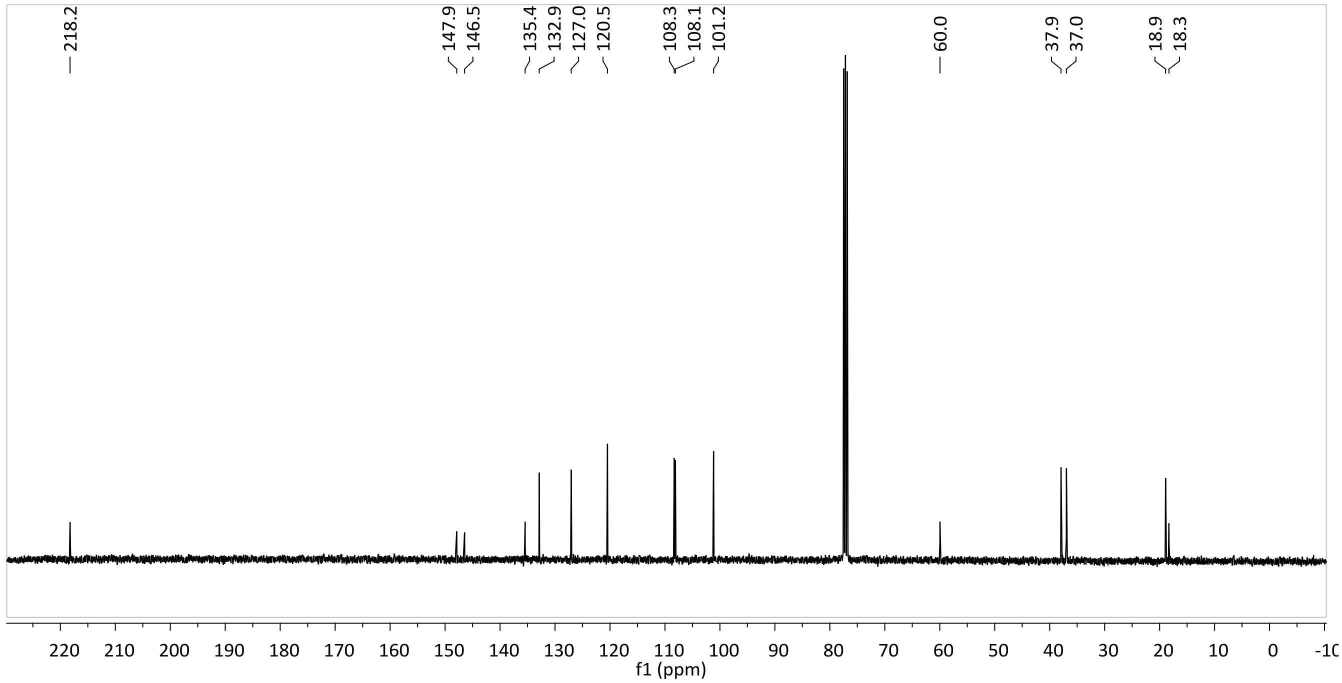
218.2
147.9
146.5
135.4
132.9
127.0
120.5
108.3
108.1
101.2
60.0
37.9
37.0
18.9
18.3
220
210
200
190
180
170
160
150
140
130
120
110
100
90
80
70
60
50
40
30
20
10
0
-10
f1 (ppm)

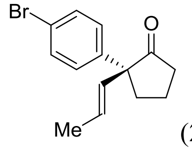

(**2d**)

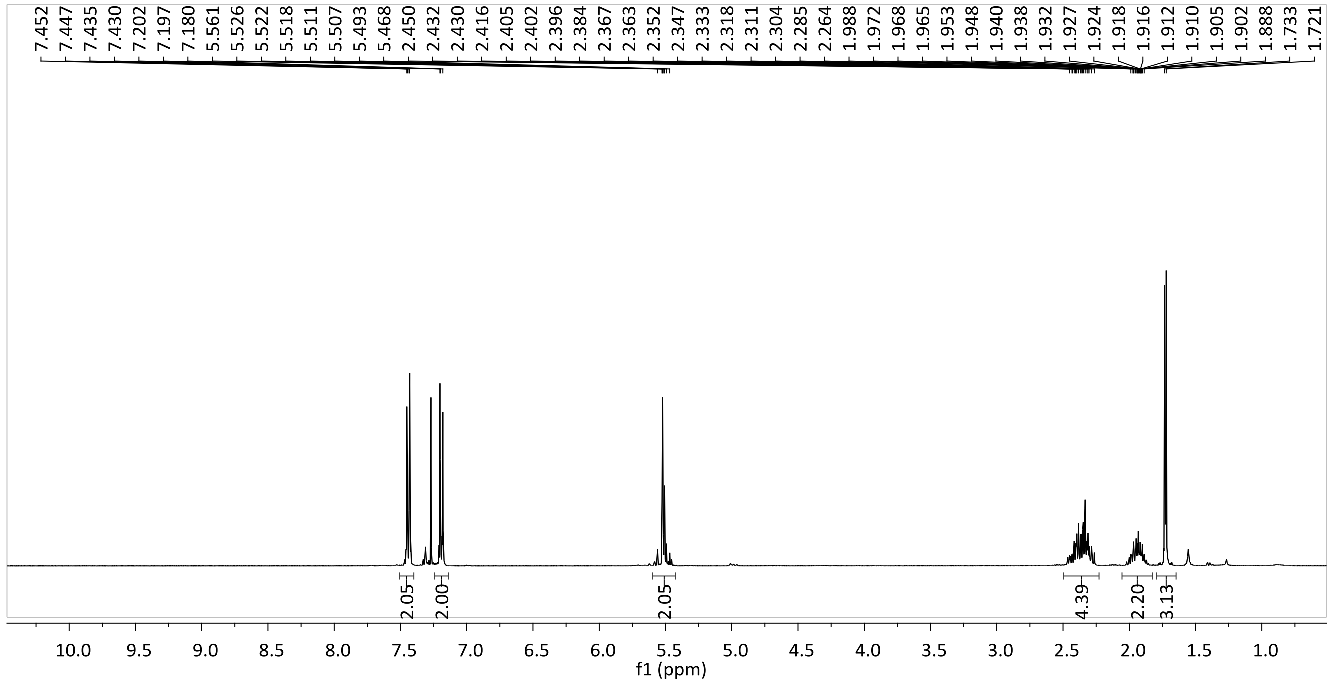

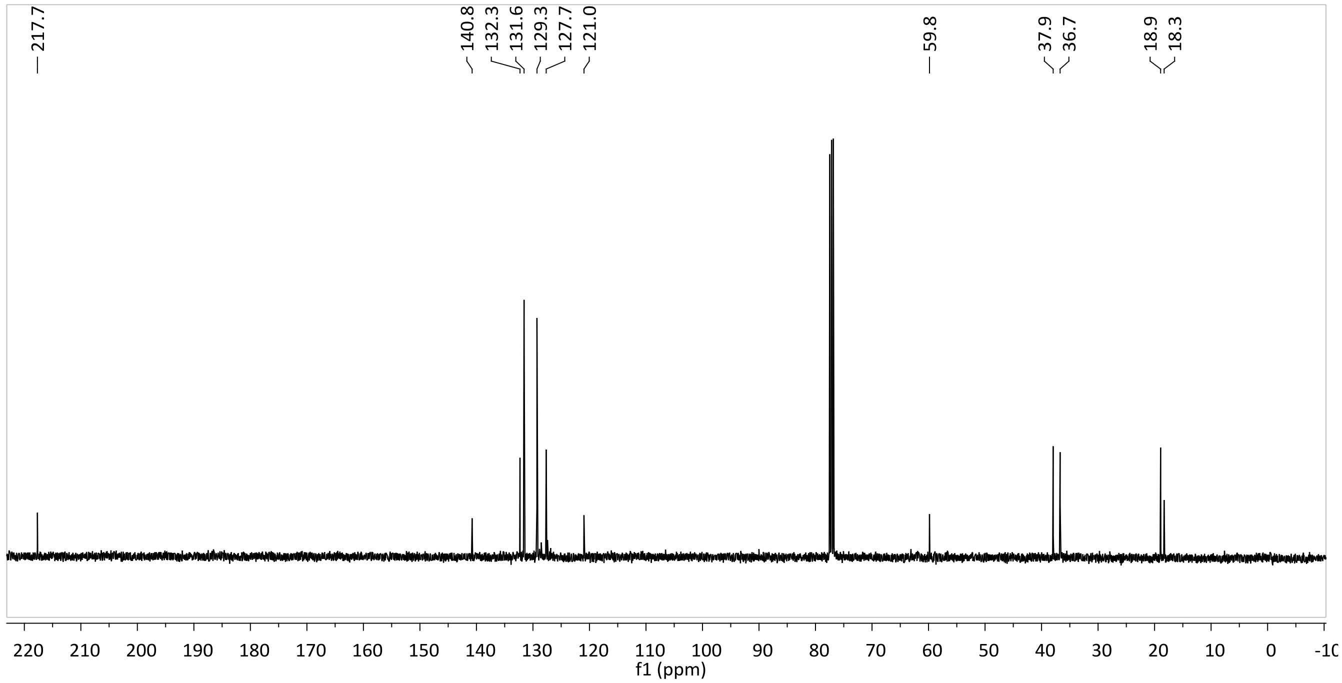

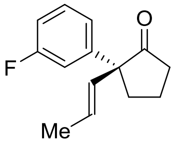
O
F
Me

(**2e**)

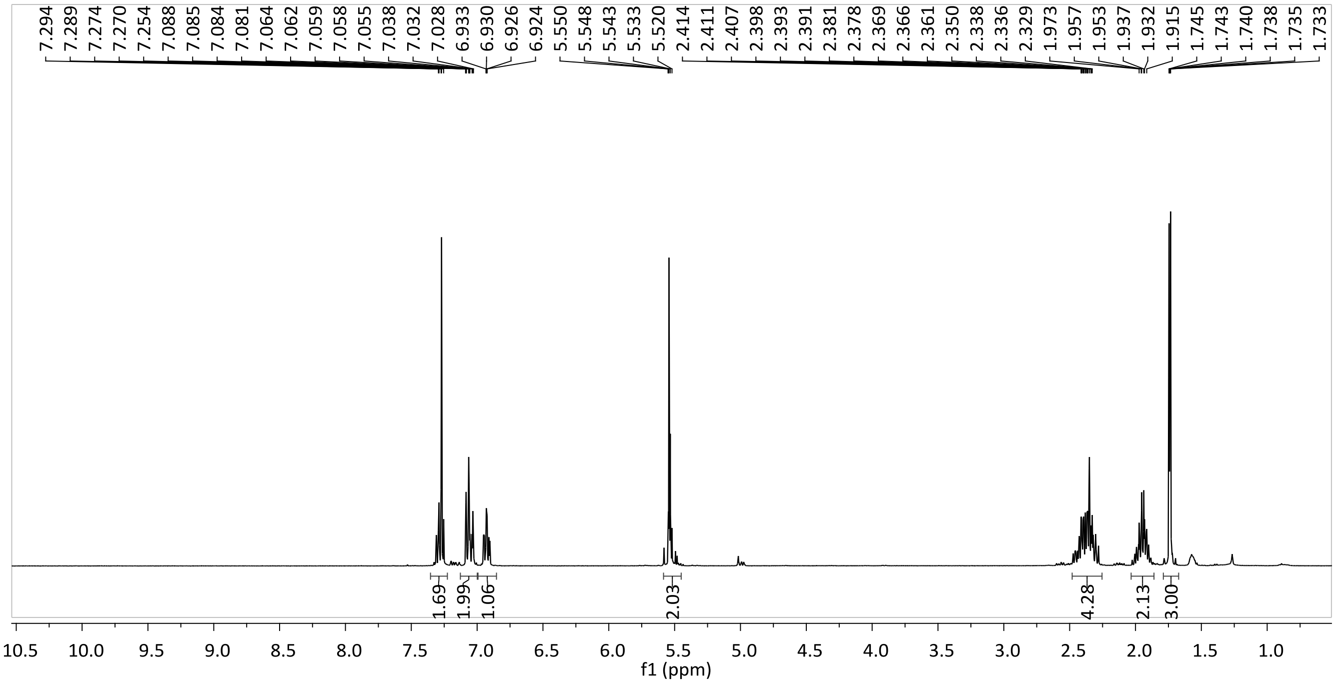
7.294
7.289
7.274
7.270
7.254
7.088
7.085
7.084
7.081
7.064
7.062
7.059
7.058
7.055
7.038
7.032
7.028
6.933
6.930
6.926
6.924
5.550
5.548
5.543
5.533
5.520
2.414
2.411
2.407
2.398
2.393
2.391
2.381
2.378
2.369
2.366
2.361
2.350
2.338
2.336
2.329
1.973
1.957
1.953
1.937
1.932
1.915
1.745
1.743
1.740
1.738
1.735
1.733
1.69
1.99
1.06
2.03
4.28
2.13
3.00
10.5
10.0
9.5
9.0
8.5
8.0
7.5
7.0
6.5
6.0
5.5
5.0
4.5
4.0
3.5
3.0
2.5
2.0
1.5
1.0
f1 (ppm)

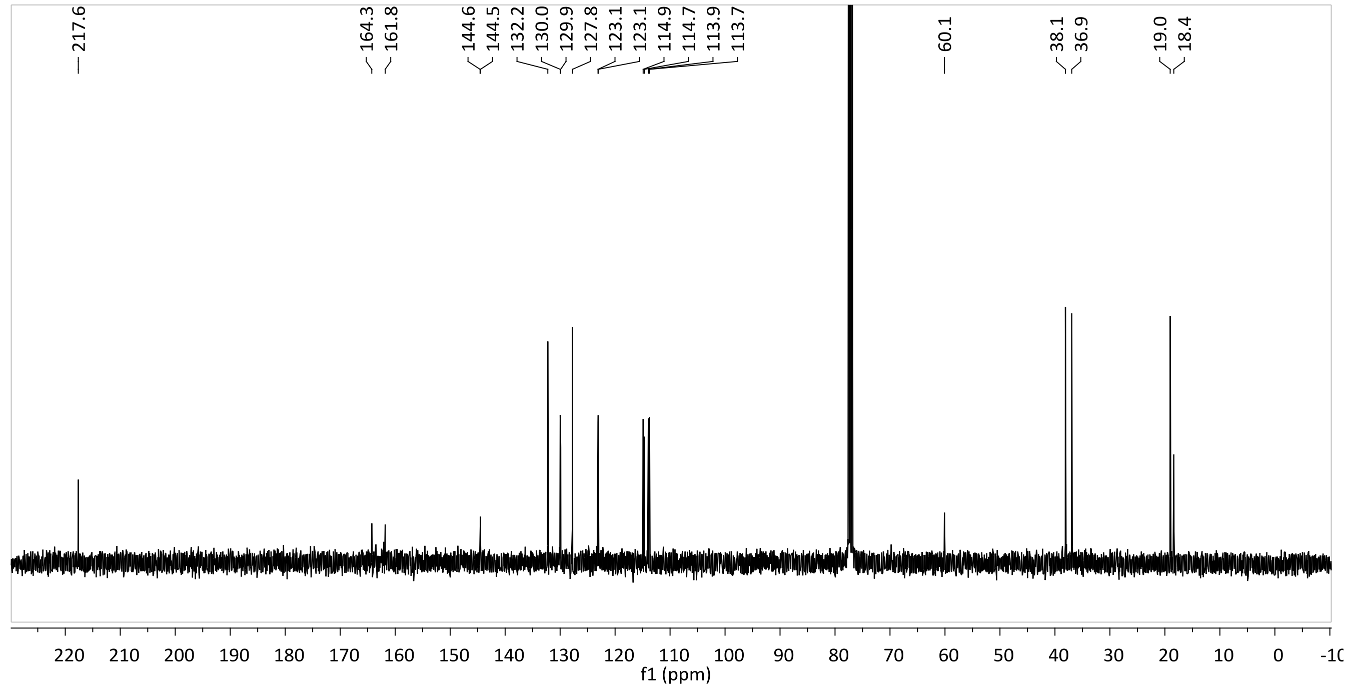
217.6
164.3
161.8
144.6
144.5
132.2
130.0
129.9
127.8
123.1
123.1
114.9
114.7
113.9
113.7
60.1
38.1
36.9
19.0
18.4
220
210
200
190
180
170
160
150
140
130
120
110
100
90
80
70
60
50
40
30
20
10
0
f1 (ppm)

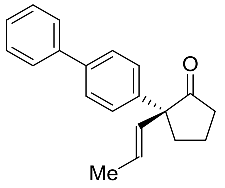
O
Me

(2f)

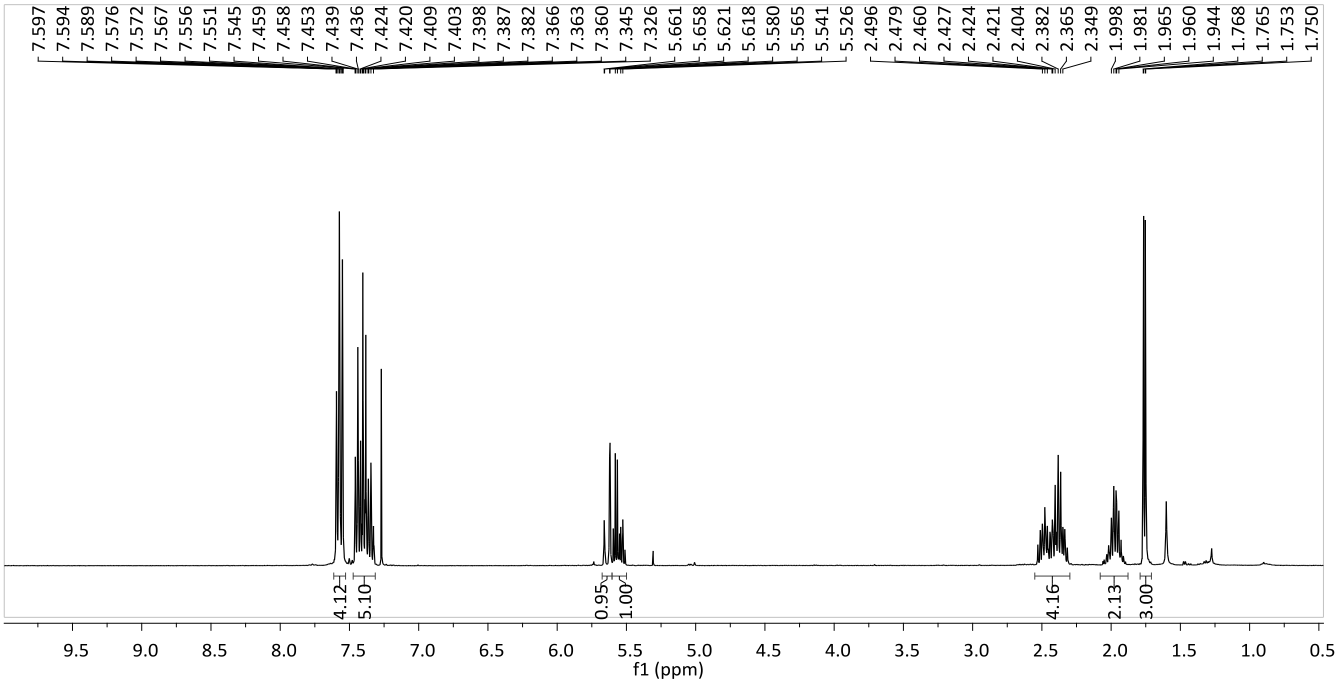
7.597
7.594
7.589
7.576
7.572
7.567
7.556
7.551
7.545
7.459
7.458
7.453
7.439
7.436
7.424
7.420
7.409
7.403
7.398
7.387
7.382
7.366
7.363
7.360
7.345
7.326
5.661
5.658
5.621
5.618
5.580
5.565
5.541
5.526
2.496
2.479
2.460
2.427
2.424
2.421
2.404
2.382
2.365
2.349
1.998
1.981
1.965
1.960
1.944
1.768
1.765
1.753
1.750
4.12
5.10
0.95
1.00
4.16
2.13
3.00
9.5 9.0 8.5 8.0 7.5 7.0 6.5 6.0 5.5 5.0 4.5 4.0 3.5 3.0 2.5 2.0 1.5 1.0 0.5
f1 (ppm)

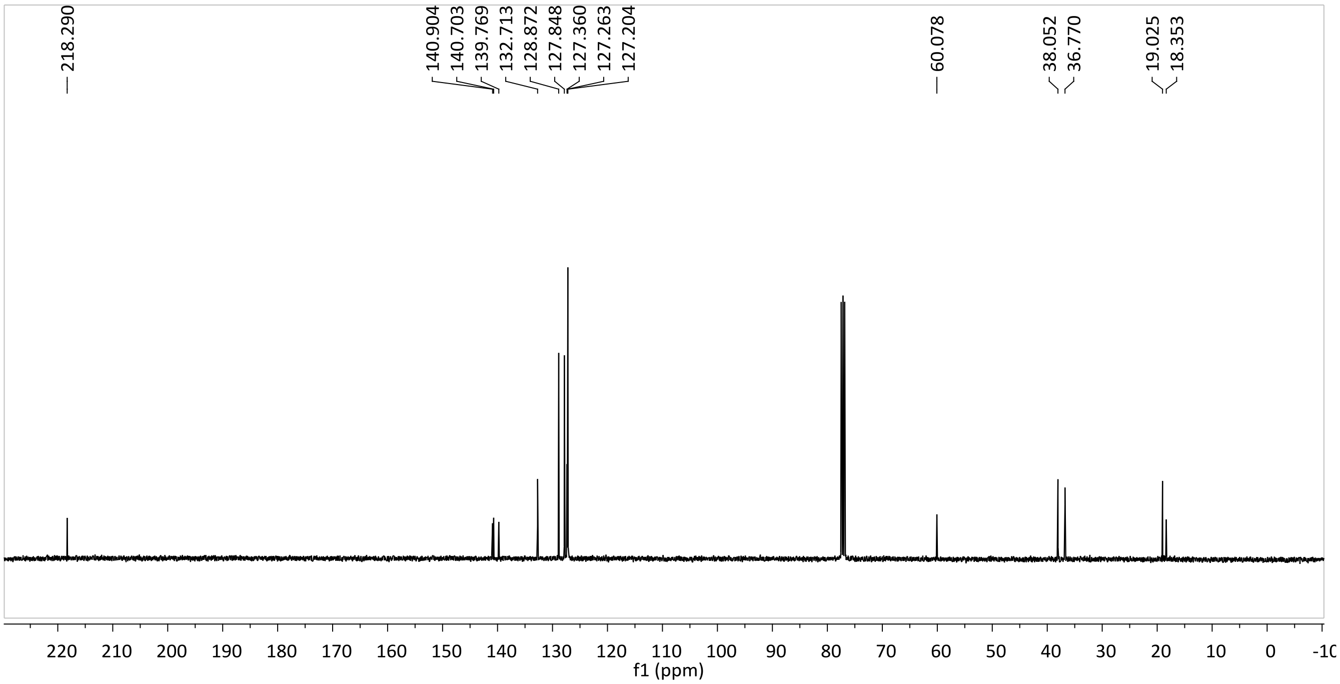
218.290
140.904
140.703
139.769
132.713
128.872
127.848
127.360
127.263
127.204
60.078
38.052
36.770
19.025
18.353
220 210 200 190 180 170 160 150 140 130 120 110 100 90 80 70 60 50 40 30 20 10 0 -10
f1 (ppm)

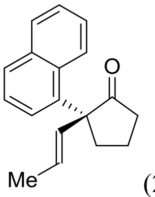
O
Me

(**2g**)

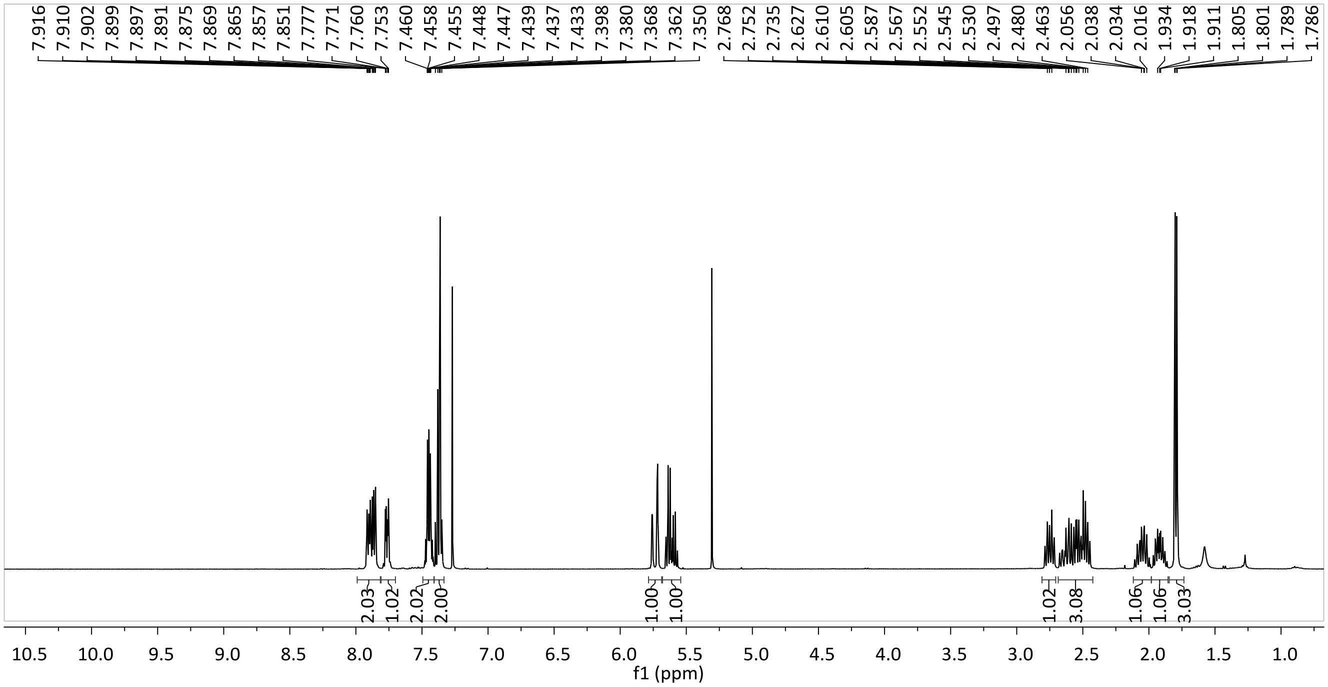
7.916
7.910
7.902
7.899
7.897
7.891
7.875
7.869
7.865
7.857
7.851
7.777
7.771
7.760
7.753
7.460
7.458
7.455
7.448
7.447
7.439
7.437
7.433
7.398
7.380
7.368
7.362
7.350
2.768
2.752
2.735
2.627
2.610
2.605
2.587
2.567
2.552
2.545
2.530
2.497
2.480
2.463
2.056
2.038
2.034
2.016
1.934
1.918
1.911
1.805
1.801
1.789
1.786
2.03
1.02
2.02
2.00
1.00
1.00
1.02
3.08
1.06
1.06
3.03
10.5 10.0 9.5 9.0 8.5 8.0 7.5 7.0 6.5 6.0 5.5 5.0 4.5 4.0 3.5 3.0 2.5 2.0 1.5 1.0
f1 (ppm)

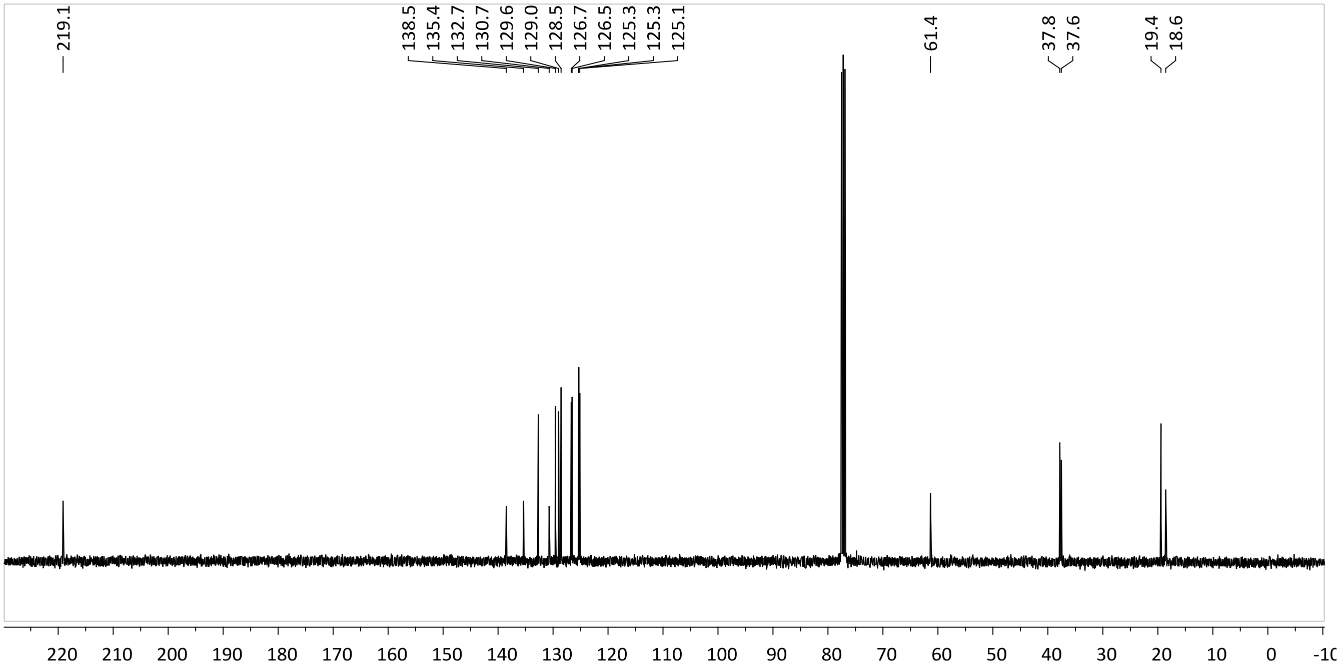
219.1
138.5
135.4
132.7
130.7
129.6
129.0
128.5
126.7
126.5
125.3
125.3
125.1
61.4
37.8
37.6
19.4
18.6
220 210 200 190 180 170 160 150 140 130 120 110 100 90 80 70 60 50 40 30 20 10 0 -10
f1 (ppm)

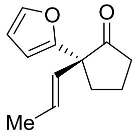
O
O
Me

(2h)

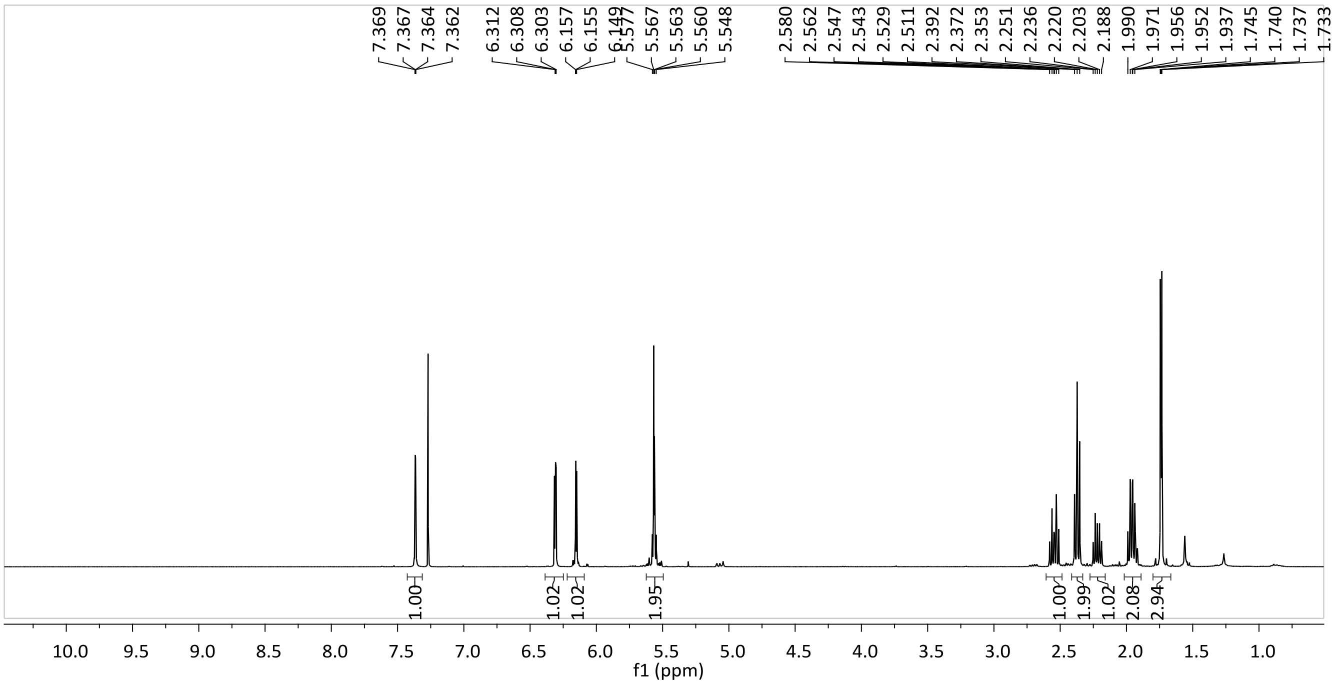
7.369
7.367
7.364
7.362
6.312
6.308
6.303
6.157
6.155
6.149
5.577
5.567
5.563
5.560
5.548
2.580
2.562
2.547
2.543
2.529
2.511
2.392
2.372
2.353
2.251
2.236
2.220
2.203
2.188
1.990
1.971
1.956
1.952
1.937
1.745
1.740
1.737
1.733
1.00
1.02
1.02
1.95
1.00
1.99
1.02
2.08
2.94
10.0 9.5 9.0 8.5 8.0 7.5 7.0 6.5 6.0 5.5 5.0 4.5 4.0 3.5 3.0 2.5 2.0 1.5 1.0
f1 (ppm)

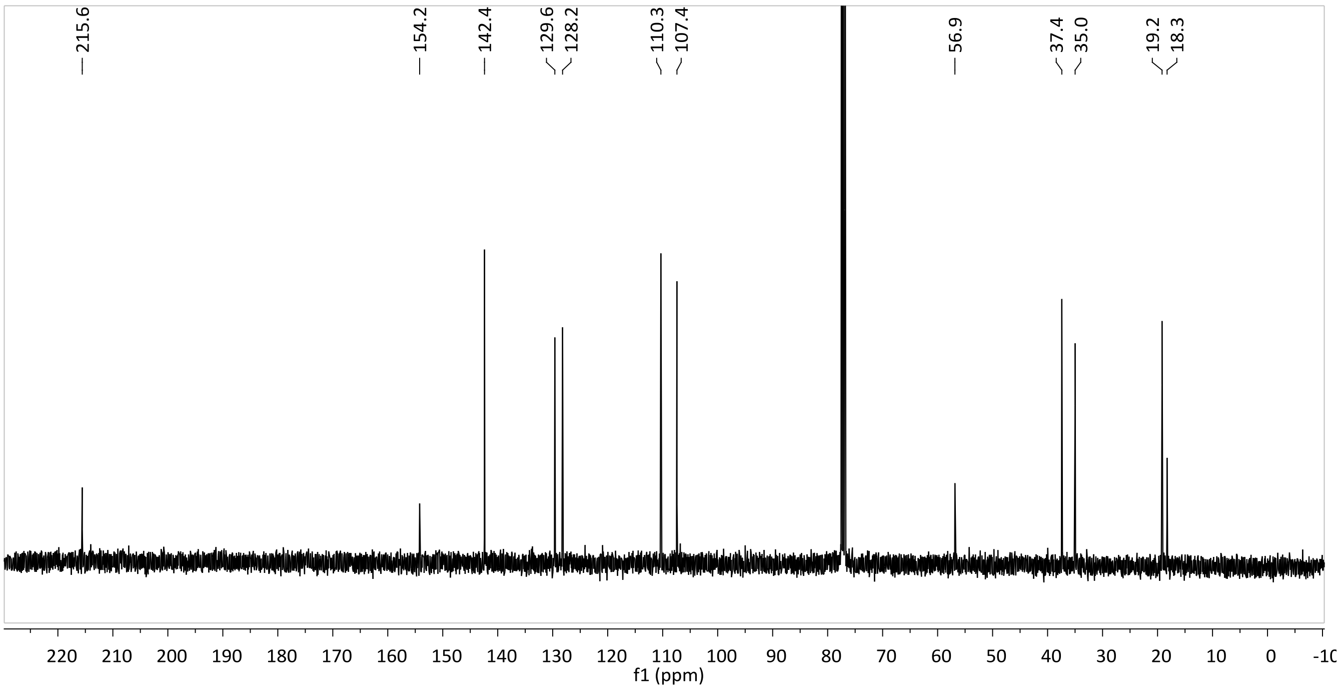
215.6
154.2
142.4
129.6
128.2
110.3
107.4
56.9
37.4
35.0
19.2
18.3
220 210 200 190 180 170 160 150 140 130 120 110 100 90 80 70 60 50 40 30 20 10 0 -10
f1 (ppm)

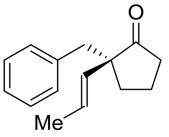
O
Me

(**2i**)

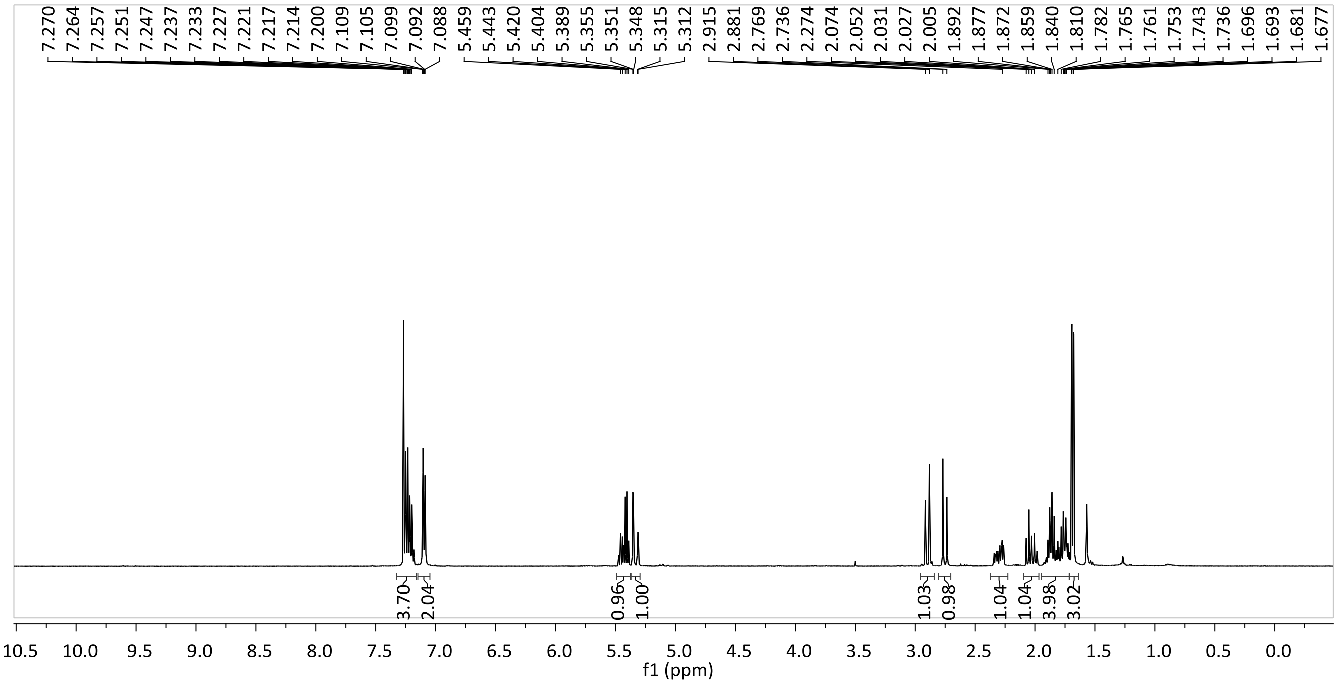
7.270
7.264
7.257
7.251
7.247
7.237
7.233
7.227
7.221
7.217
7.214
7.200
7.109
7.105
7.099
7.092
7.088
5.459
5.443
5.420
5.404
5.389
5.355
5.351
5.348
5.315
5.312
2.915
2.881
2.769
2.736
2.274
2.074
2.052
2.031
2.027
2.005
1.892
1.877
1.872
1.859
1.840
1.810
1.782
1.765
1.761
1.753
1.743
1.736
1.696
1.693
1.681
1.677
3.70
2.04
0.96
1.00
1.03
0.98
1.04
1.04
3.98
3.02
f1 (ppm)

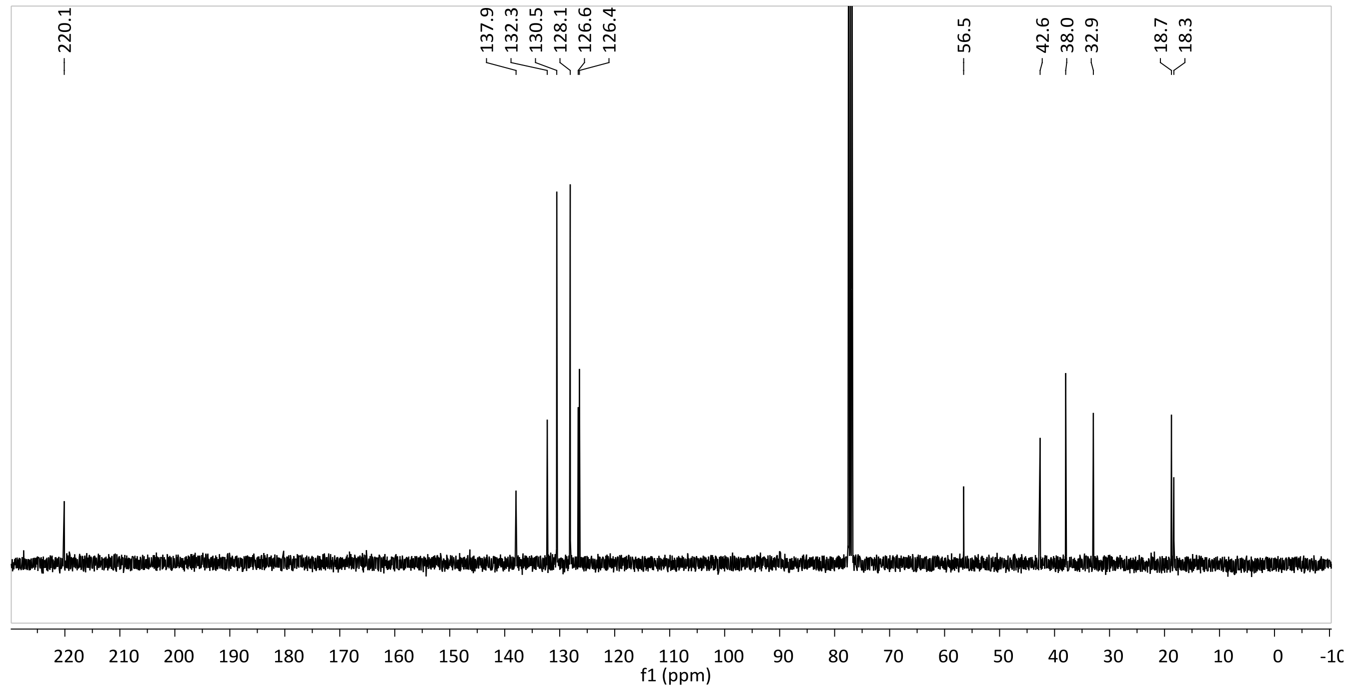
220.1
137.9
132.3
130.5
128.1
126.6
126.4
56.5
42.6
38.0
32.9
18.7
18.3
f1 (ppm)

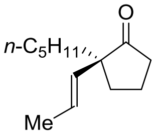
O
n-C5H11
Me

(**2j**)

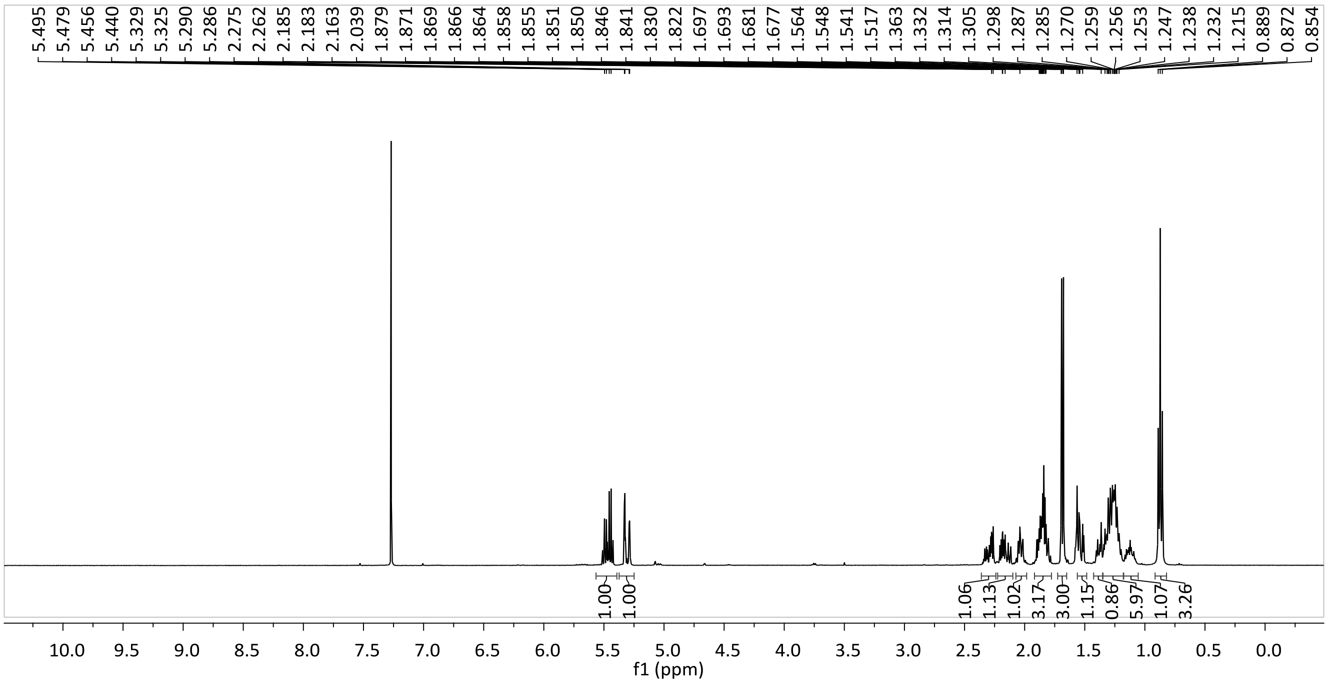
5.495 5.479 5.456 5.440 5.329 5.325 5.290 5.286 2.275 2.262 2.185 2.183 2.163 2.039 1.879 1.871 1.869 1.866 1.864 1.858 1.855 1.851 1.850 1.846 1.841 1.830 1.822 1.697 1.693 1.681 1.677 1.564 1.548 1.541 1.517 1.363 1.332 1.314 1.305 1.298 1.287 1.285 1.270 1.259 1.256 1.253 1.247 1.238 1.232 1.215 0.889 0.872 0.854
1.00 1.00 1.06 1.13 1.02 3.17 3.00 1.15 0.86 5.97 1.07 3.26
10.0 9.5 9.0 8.5 8.0 7.5 7.0 6.5 6.0 5.5 5.0 4.5 4.0 3.5 3.0 2.5 2.0 1.5 1.0 0.5 0.0
f1 (ppm)

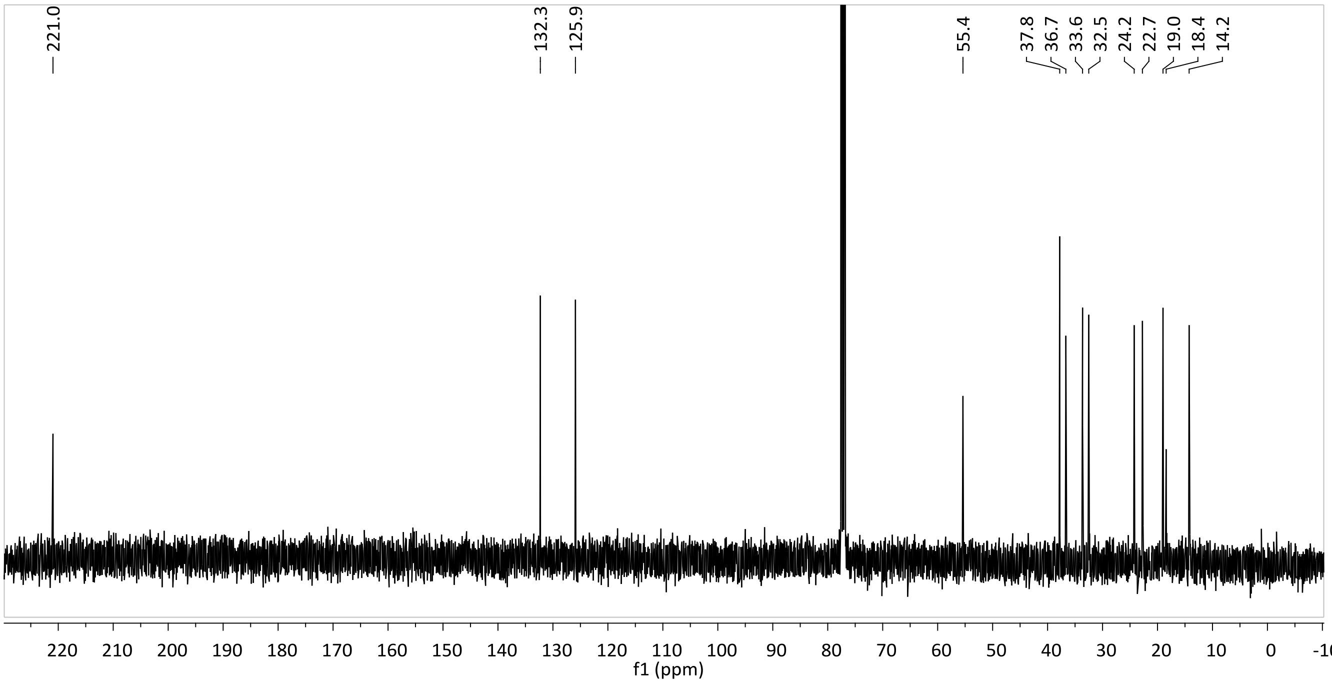
221.0
132.3
125.9
55.4
37.8 36.7 33.6 32.5 24.2 22.7 19.0 18.4 14.2
220 210 200 190 180 170 160 150 140 130 120 110 100 90 80 70 60 50 40 30 20 10 0 -10
f1 (ppm)

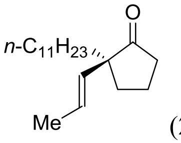
O
n-C11H23
Me

(**2k**)

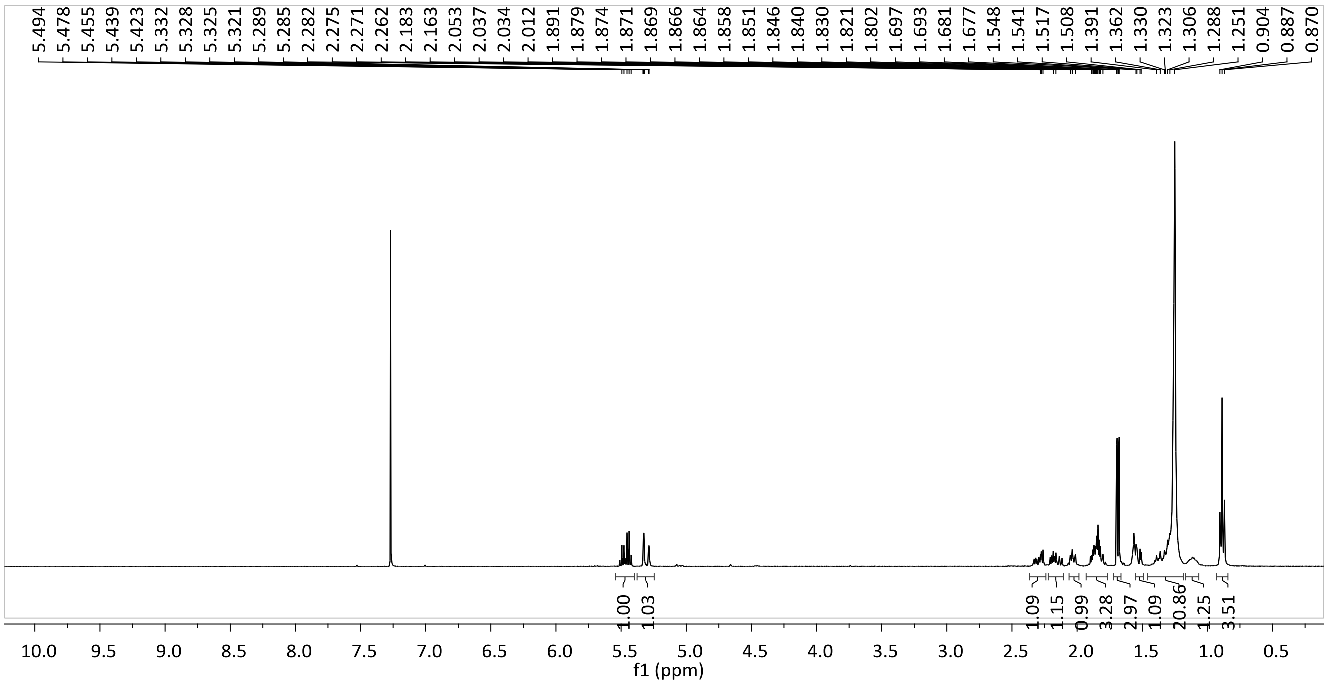
5.494
5.478
5.455
5.439
5.423
5.332
5.328
5.325
5.321
5.289
5.285
2.282
2.275
2.271
2.262
2.183
2.163
2.053
2.037
2.034
2.012
1.891
1.879
1.874
1.871
1.869
1.866
1.864
1.858
1.851
1.846
1.840
1.830
1.821
1.802
1.697
1.693
1.681
1.677
1.548
1.541
1.517
1.508
1.391
1.362
1.330
1.323
1.306
1.288
1.251
0.904
0.887
0.870
1.00
1.03
1.09
1.15
0.99
3.28
2.97
1.09
20.86
1.25
3.51
10.0
9.5
9.0
8.5
8.0
7.5
7.0
6.5
6.0
5.5
5.0
4.5
4.0
3.5
3.0
2.5
2.0
1.5
1.0
0.5
f1 (ppm)

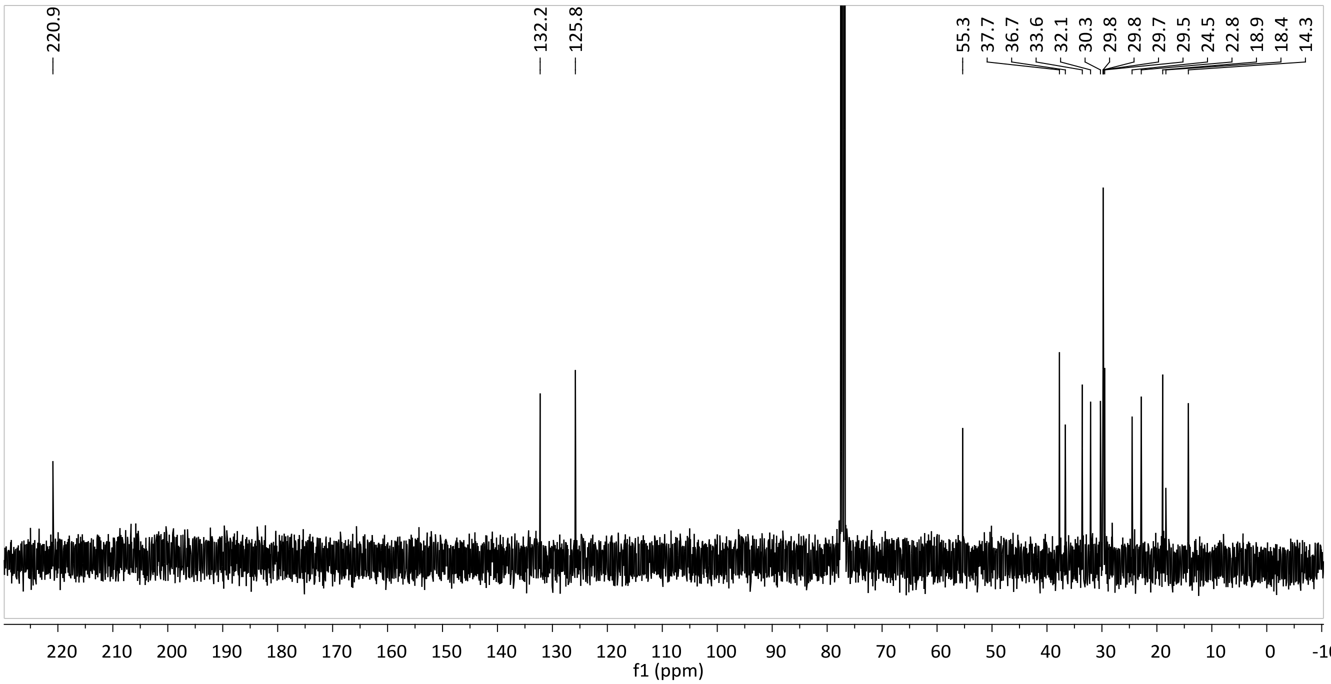
220.9
132.2
125.8
55.3
37.7
36.7
33.6
32.1
30.3
29.8
29.8
29.7
29.5
24.5
22.8
18.9
18.4
14.3
220
210
200
190
180
170
160
150
140
130
120
110
100
90
80
70
60
50
40
30
20
10
0
-1C
f1 (ppm)

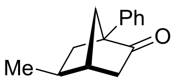
Ph
O
Me

(**3a**)

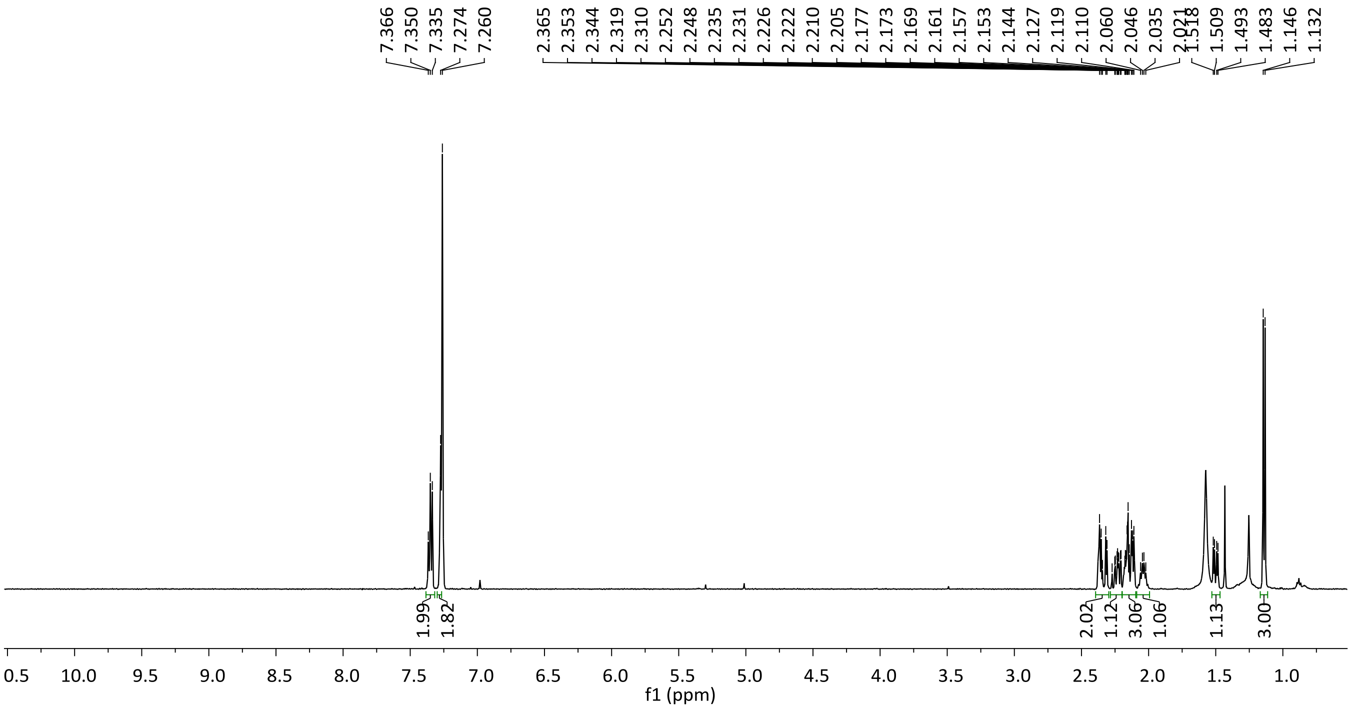
7.366
7.350
7.335
7.274
7.260
2.365
2.353
2.344
2.319
2.310
2.252
2.248
2.235
2.231
2.226
2.222
2.210
2.205
2.177
2.173
2.169
2.161
2.157
2.153
2.144
2.127
2.119
2.110
2.060
2.046
2.035
2.021
1.518
1.509
1.493
1.483
1.146
1.132
1.99
1.82
2.02
1.12
3.06
1.06
1.13
3.00
0.5 10.0 9.5 9.0 8.5 8.0 7.5 7.0 6.5 6.0 5.5 5.0 4.5 4.0 3.5 3.0 2.5 2.0 1.5 1.0
f1 (ppm)

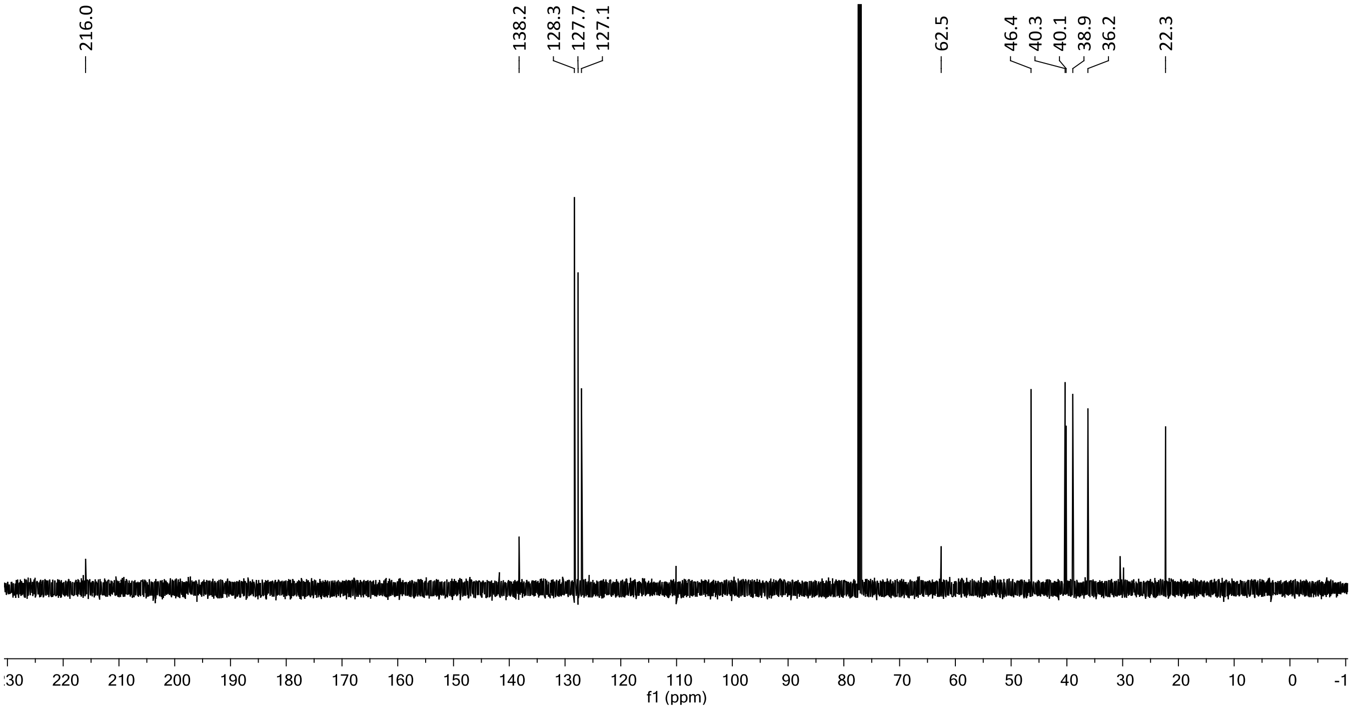
216.0
138.2
128.3
127.7
127.1
62.5
46.4
40.3
40.1
38.9
36.2
22.3
30 220 210 200 190 180 170 160 150 140 130 120 110 100 90 80 70 60 50 40 30 20 10 0 -1
f1 (ppm)

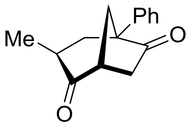
Me
Ph
O
O

(**4a**)

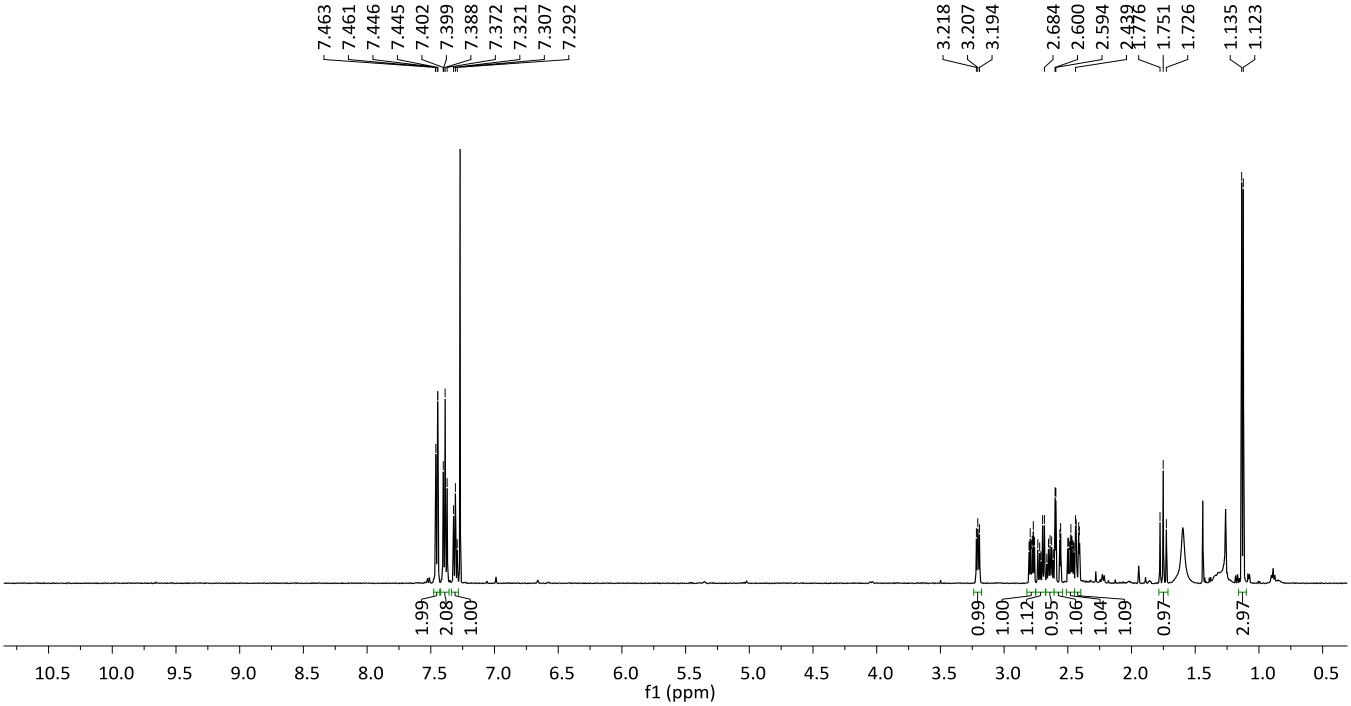
7.463
7.461
7.446
7.445
7.402
7.399
7.388
7.372
7.321
7.307
7.292
3.218
3.207
3.194
2.684
2.600
2.594
2.439
1.776
1.751
1.726
1.135
1.123
1.99
2.08
1.00
0.99
1.00
1.12
0.95
1.06
1.04
1.09
0.97
2.97
10.5 10.0 9.5 9.0 8.5 8.0 7.5 7.0 6.5 6.0 5.5 5.0 4.5 4.0 3.5 3.0 2.5 2.0 1.5 1.0 0.5
f1 (ppm)

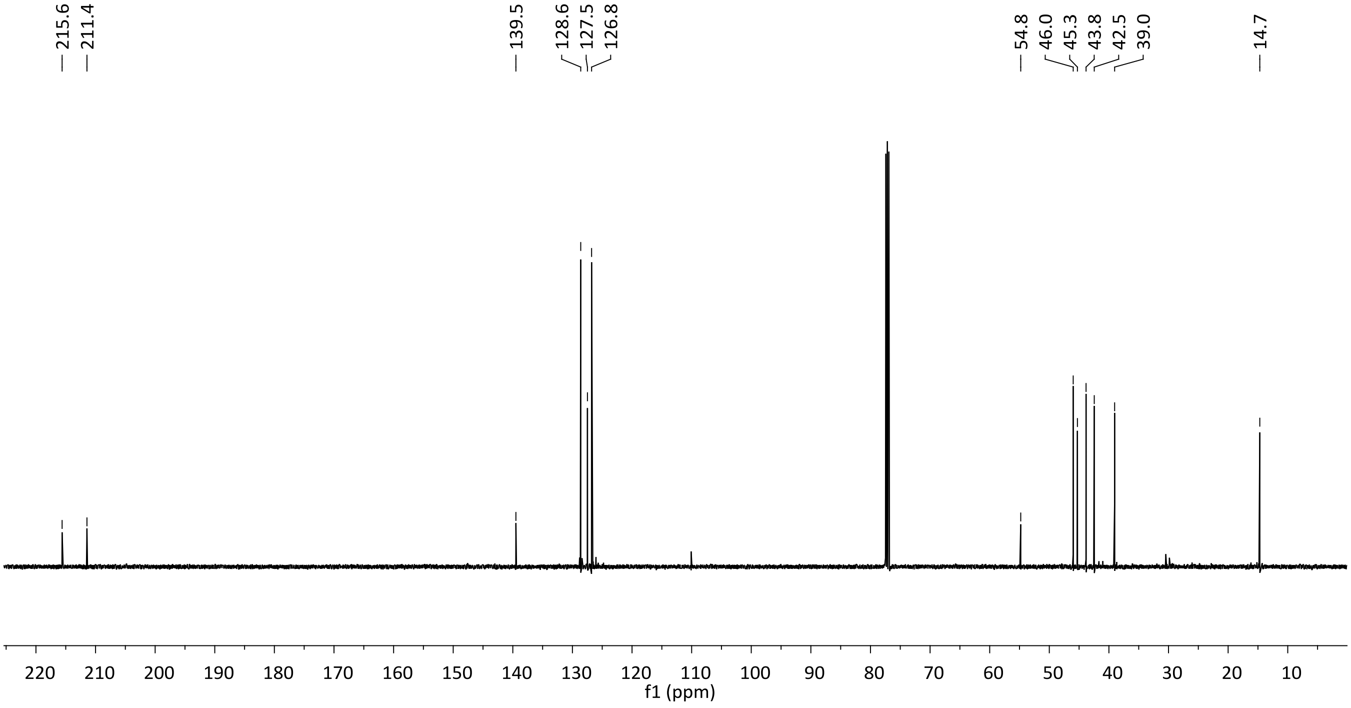
215.6
211.4
139.5
128.6
127.5
126.8
54.8
46.0
45.3
43.8
42.5
39.0
14.7
220 210 200 190 180 170 160 150 140 130 120 110 100 90 80 70 60 50 40 30 20 10
f1 (ppm)

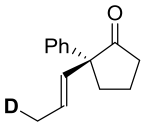
O
Ph
D

(*d*-**2a**)

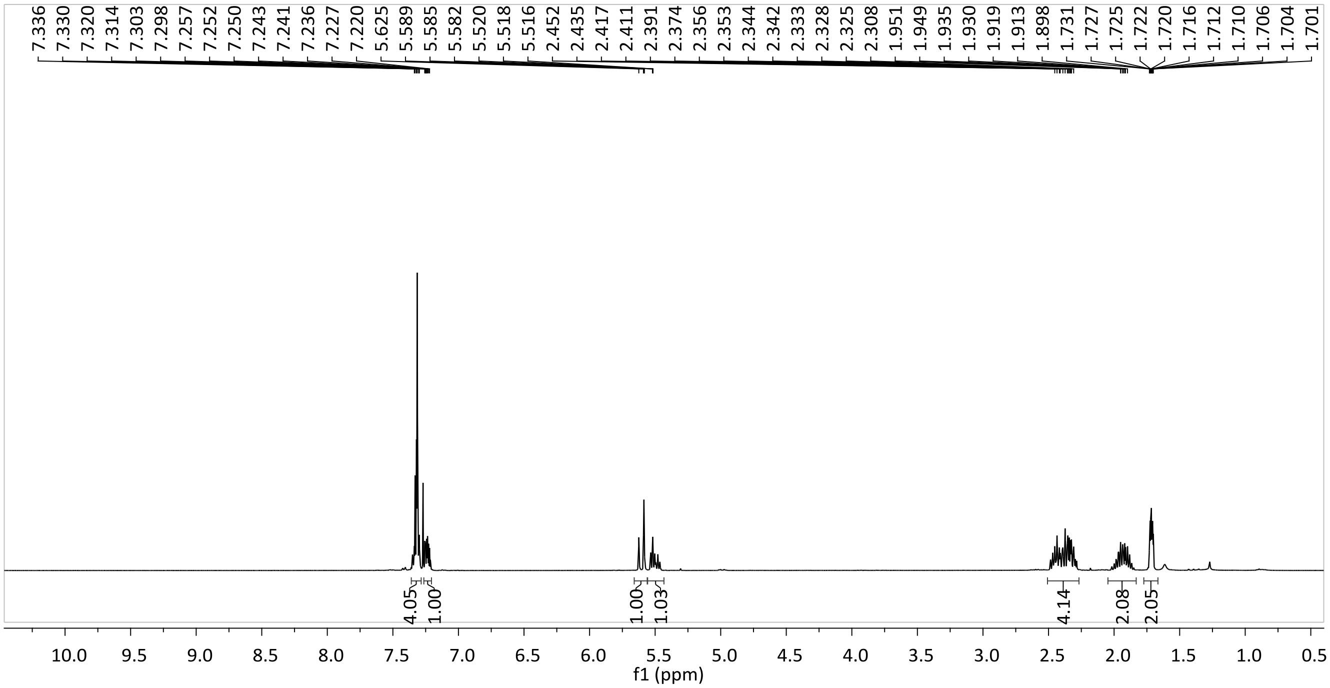
7.336
7.330
7.320
7.314
7.303
7.298
7.257
7.252
7.250
7.243
7.241
7.236
7.227
7.220
5.625
5.589
5.585
5.582
5.520
5.518
5.516
2.452
2.435
2.417
2.411
2.391
2.374
2.356
2.353
2.344
2.342
2.333
2.328
2.325
2.308
1.951
1.949
1.935
1.930
1.919
1.913
1.898
1.731
1.727
1.725
1.722
1.720
1.716
1.712
1.710
1.706
1.704
1.701
4.05
1.00
1.00
1.03
4.14
2.08
2.05
10.0 9.5 9.0 8.5 8.0 7.5 7.0 6.5 6.0 5.5 5.0 4.5 4.0 3.5 3.0 2.5 2.0 1.5 1.0 0.5
f1 (ppm)

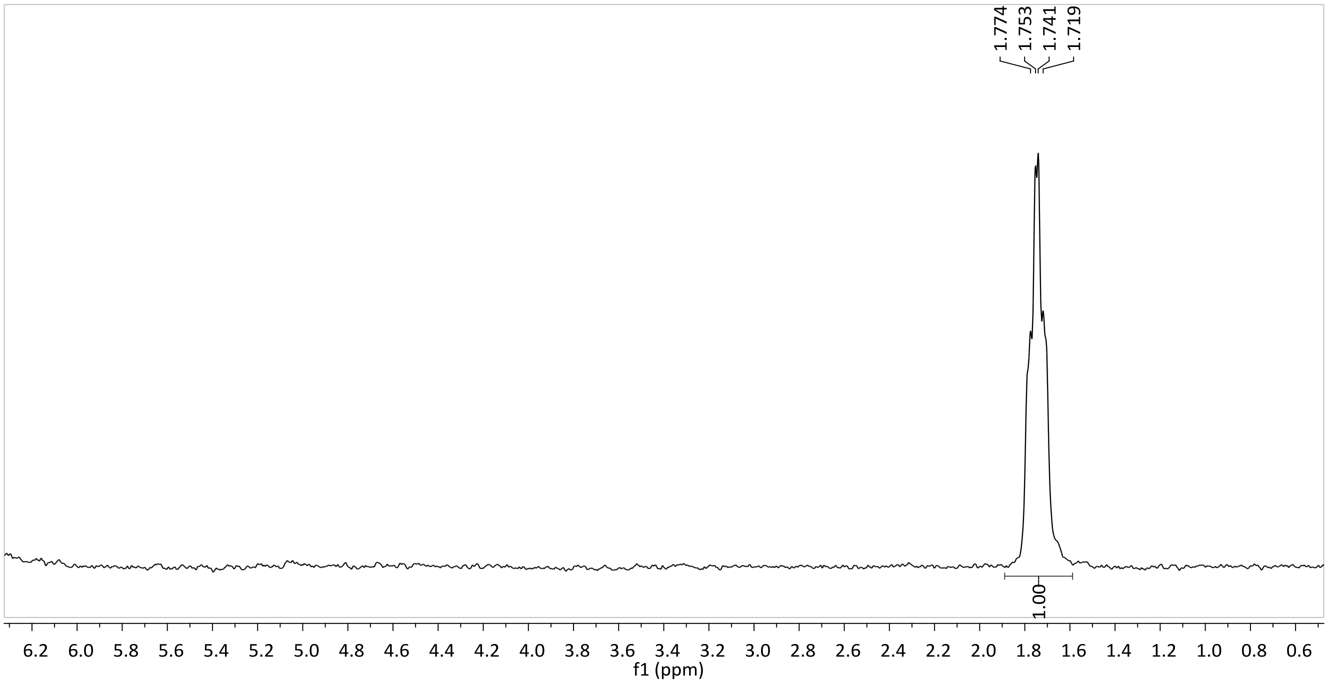
1.774
1.753
1.741
1.719
1.00
6.2 6.0 5.8 5.6 5.4 5.2 5.0 4.8 4.6 4.4 4.2 4.0 3.8 3.6 3.4 3.2 3.0 2.8 2.6 2.4 2.2 2.0 1.8 1.6 1.4 1.2 1.0 0.8 0.6
f1 (ppm)

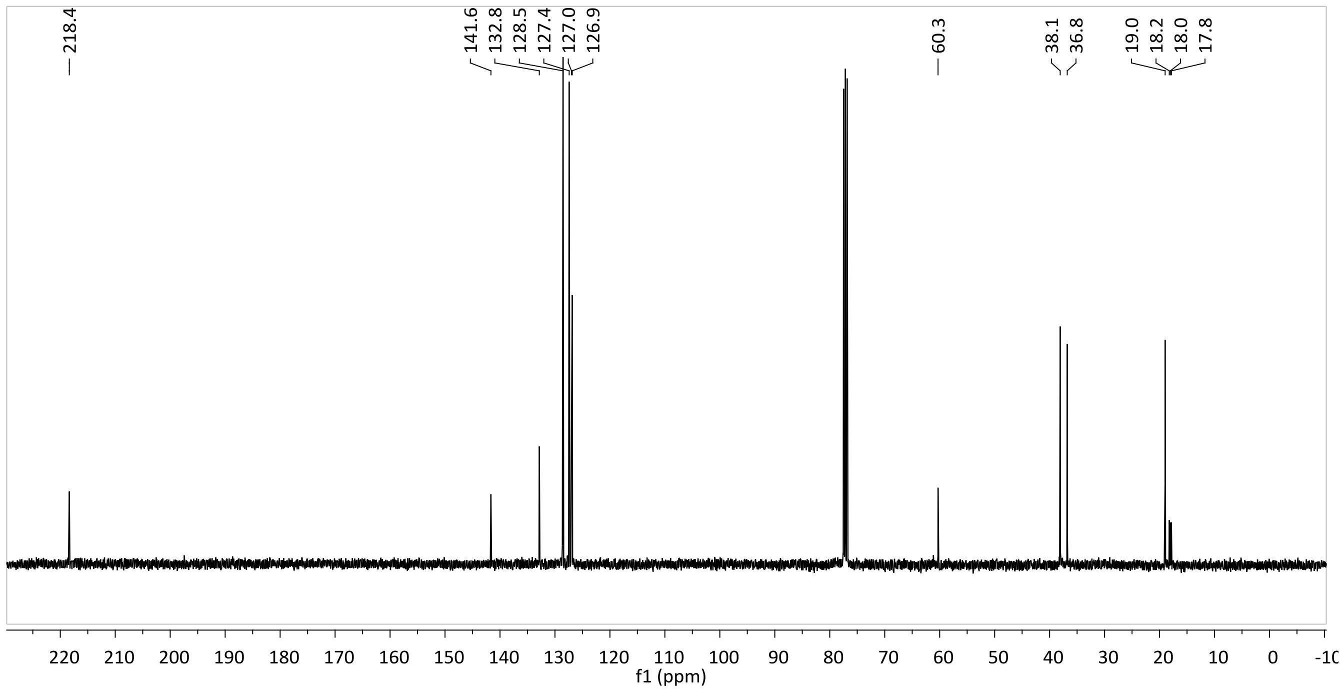
218.4
141.6
132.8
128.5
127.4
127.0
126.9
60.3
38.1
36.8
19.0
18.2
18.0
17.8
220 210 200 190 180 170 160 150 140 130 120 110 100 90 80 70 60 50 40 30 20 10 0 -10
f1 (ppm)

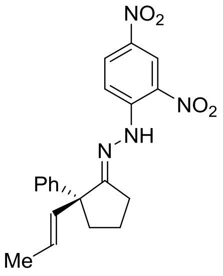

NO2
NO2
NH
N
Ph
Me

(9)

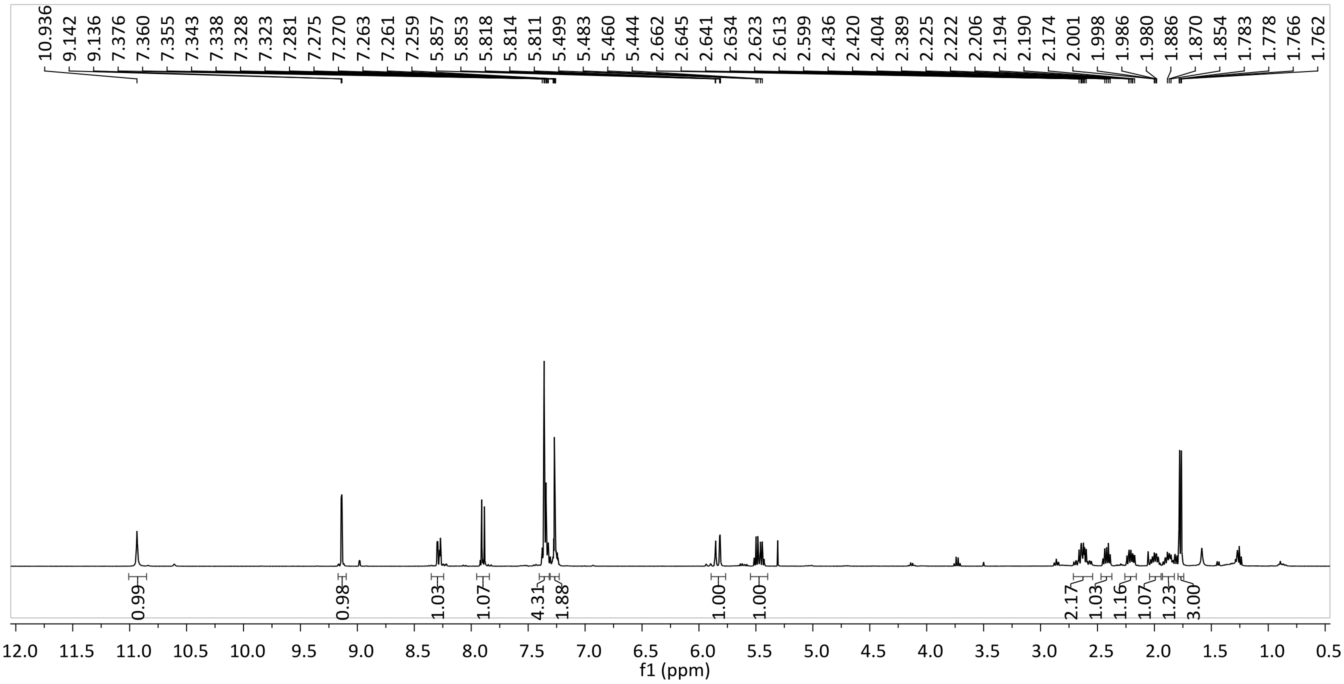

10.936
9.142
9.136
7.376
7.360
7.355
7.343
7.338
7.328
7.323
7.281
7.275
7.270
7.263
7.261
7.259
5.857
5.853
5.818
5.814
5.811
5.499
5.483
5.460
5.444
2.662
2.645
2.641
2.634
2.623
2.613
2.599
2.436
2.420
2.404
2.389
2.225
2.222
2.206
2.194
2.190
2.174
2.001
1.998
1.986
1.980
1.886
1.870
1.854
1.783
1.778
1.766
1.762
0.99
0.98
1.03
1.07
4.31
1.88
1.00
1.00
2.17
1.03
1.16
1.07
1.23
3.00
f1 (ppm)

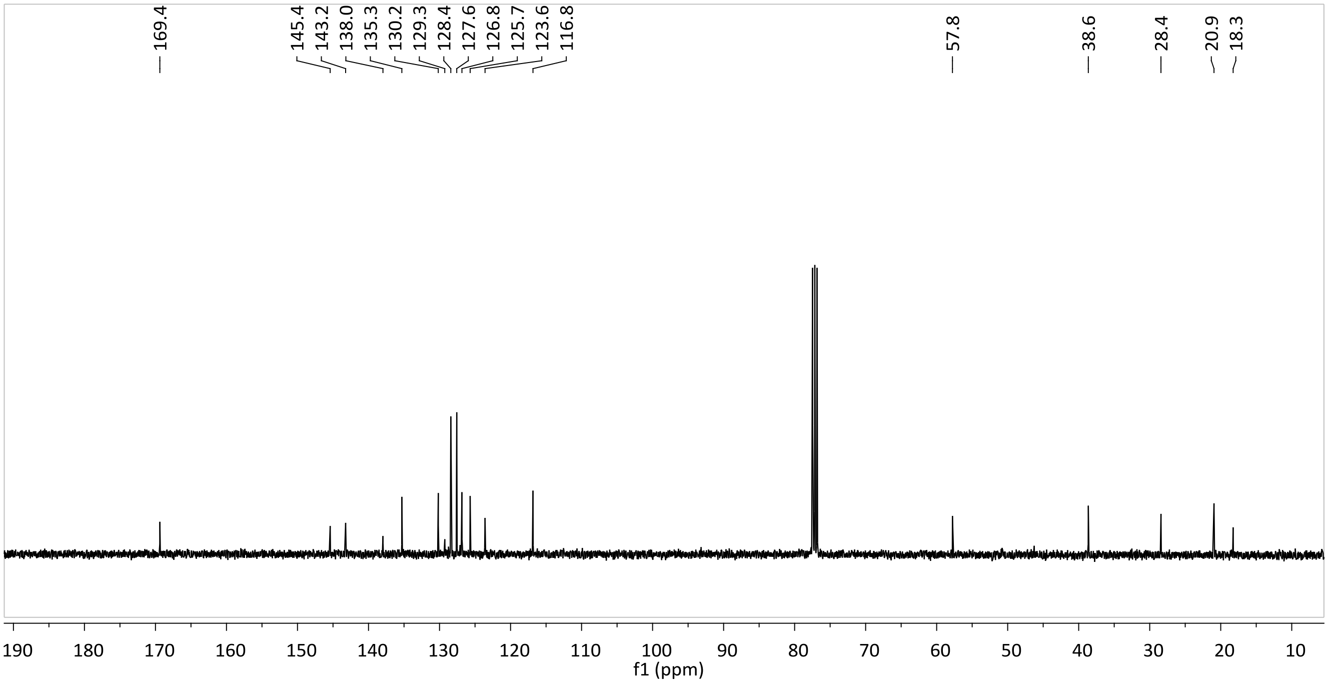

169.4
145.4
143.2
138.0
135.3
130.2
129.3
128.4
127.6
126.8
125.7
123.6
116.8
57.8
38.6
28.4
20.9
18.3
f1 (ppm)

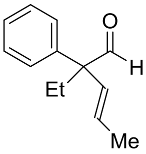
O
H
Et
Me

(11)

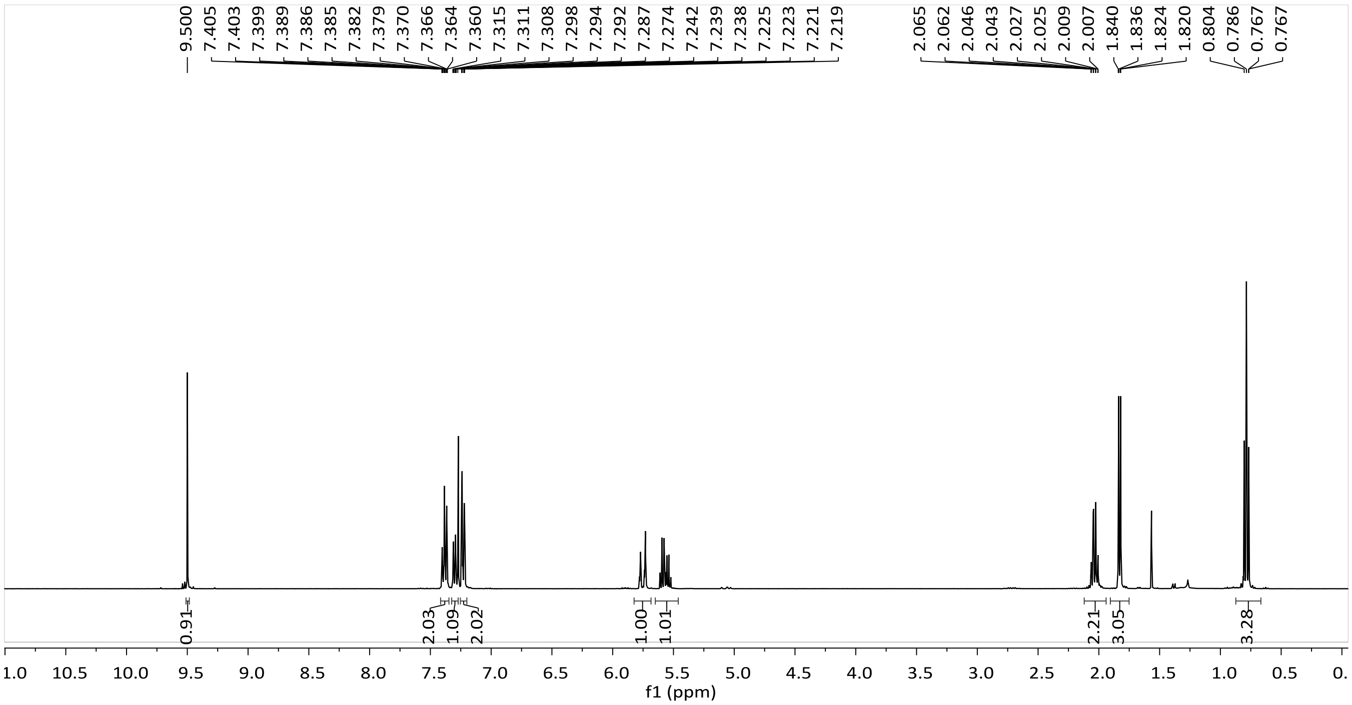
9.500
7.405
7.403
7.399
7.389
7.386
7.385
7.382
7.379
7.370
7.366
7.364
7.360
7.315
7.311
7.308
7.298
7.294
7.292
7.287
7.274
7.242
7.239
7.238
7.225
7.223
7.221
7.219
2.065
2.062
2.046
2.043
2.027
2.025
2.009
2.007
1.840
1.836
1.824
1.820
0.804
0.786
0.767
0.767
0.91
2.03
1.09
2.02
1.00
1.01
2.21
3.05
3.28
f1 (ppm)

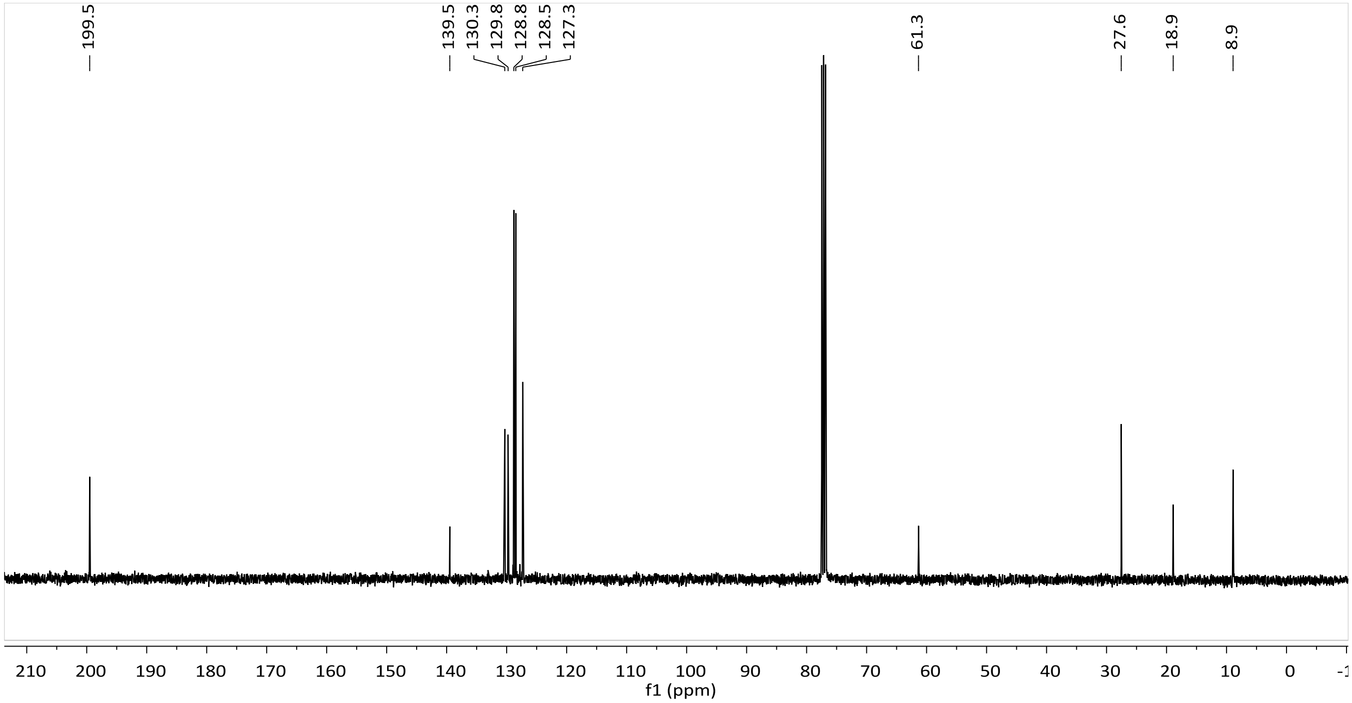
199.5
139.5
130.3
129.8
128.8
128.5
127.3
61.3
27.6
18.9
8.9
f1 (ppm)

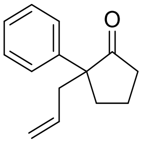
O

(12a)

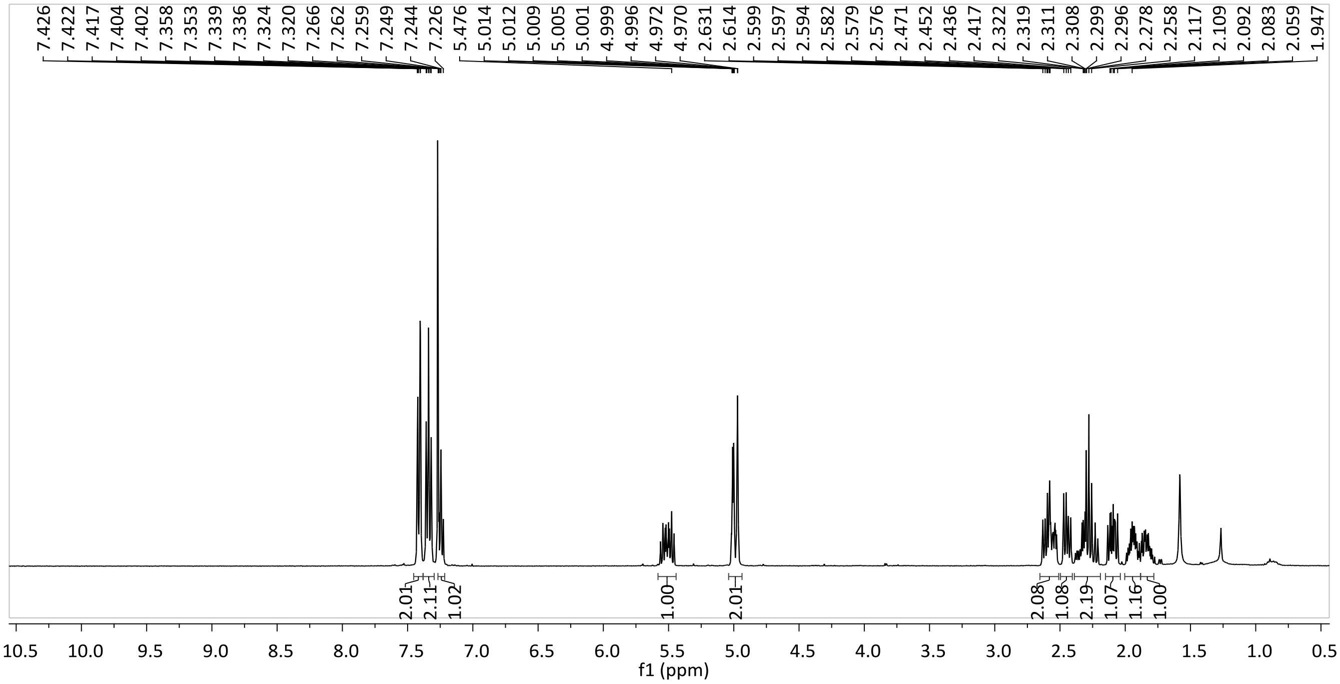
7.426
7.422
7.417
7.404
7.402
7.358
7.353
7.339
7.336
7.324
7.320
7.266
7.262
7.259
7.249
7.244
7.226
5.476
5.014
5.012
5.009
5.005
5.001
4.999
4.996
4.972
4.970
2.631
2.614
2.599
2.597
2.594
2.582
2.579
2.576
2.471
2.452
2.436
2.417
2.322
2.319
2.311
2.308
2.299
2.296
2.278
2.258
2.117
2.109
2.092
2.083
2.059
1.947
2.01
2.11
1.02
1.00
2.01
2.08
1.08
2.19
1.07
1.16
1.00
10.5
10.0
9.5
9.0
8.5
8.0
7.5
7.0
6.5
6.0
5.5
5.0
4.5
4.0
3.5
3.0
2.5
2.0
1.5
1.0
0.5
f1 (ppm)

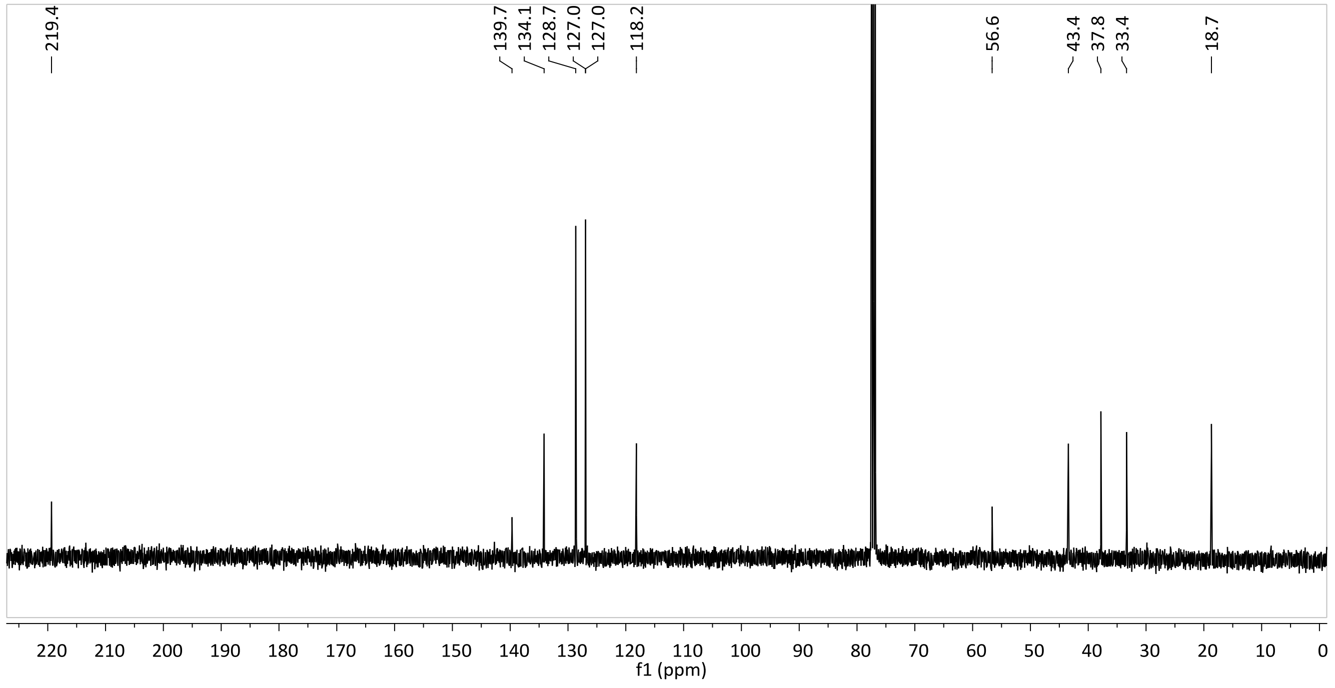
219.4
139.7
134.1
128.7
127.0
127.0
118.2
56.6
43.4
37.8
33.4
18.7
220
210
200
190
180
170
160
150
140
130
120
110
100
90
80
70
60
50
40
30
20
10
0
f1 (ppm)

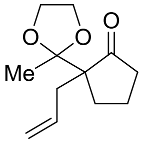
O
O
O
Me

(12l)

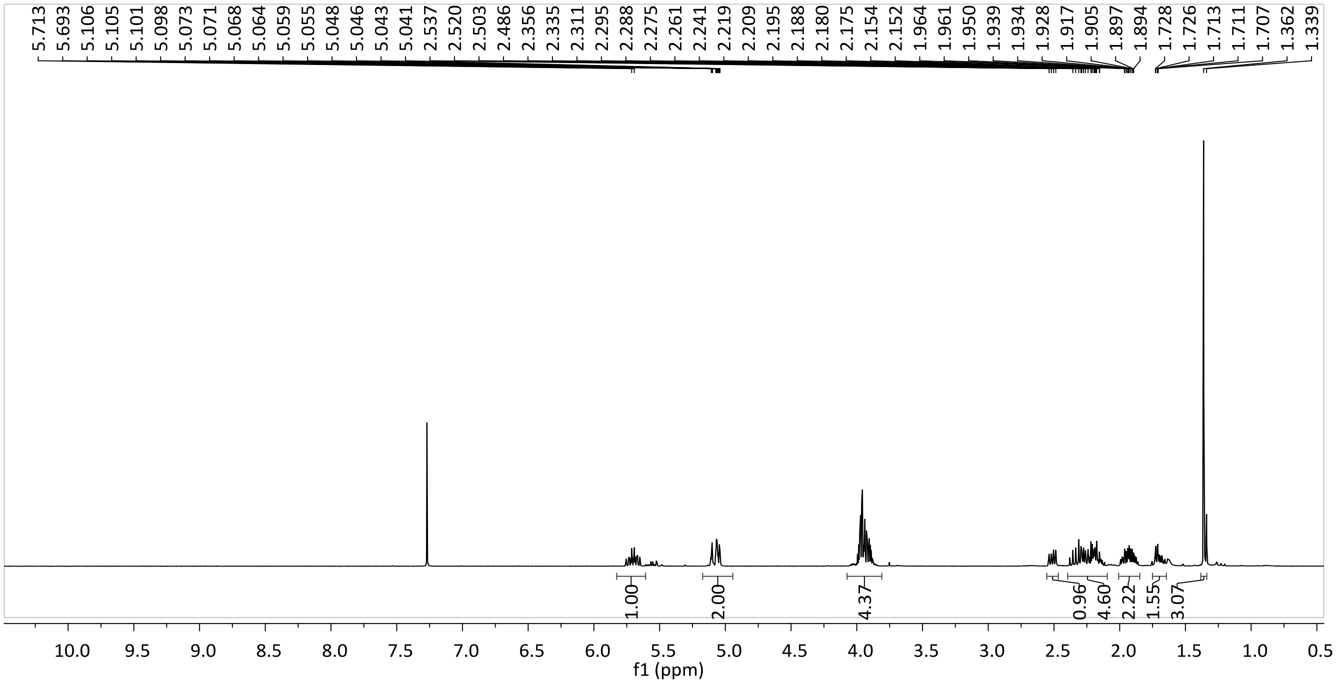
5.713
5.693
5.106
5.105
5.101
5.098
5.073
5.071
5.068
5.064
5.059
5.055
5.048
5.046
5.043
5.041
2.537
2.520
2.503
2.486
2.356
2.335
2.311
2.295
2.288
2.275
2.261
2.241
2.219
2.209
2.195
2.188
2.180
2.175
2.154
2.152
1.964
1.961
1.950
1.939
1.934
1.928
1.917
1.905
1.897
1.894
1.728
1.726
1.713
1.711
1.707
1.362
1.339
1.00
2.00
4.37
0.96
4.60
2.22
1.55
3.07
10.0 9.5 9.0 8.5 8.0 7.5 7.0 6.5 6.0 5.5 5.0 4.5 4.0 3.5 3.0 2.5 2.0 1.5 1.0 0.5
f1 (ppm)

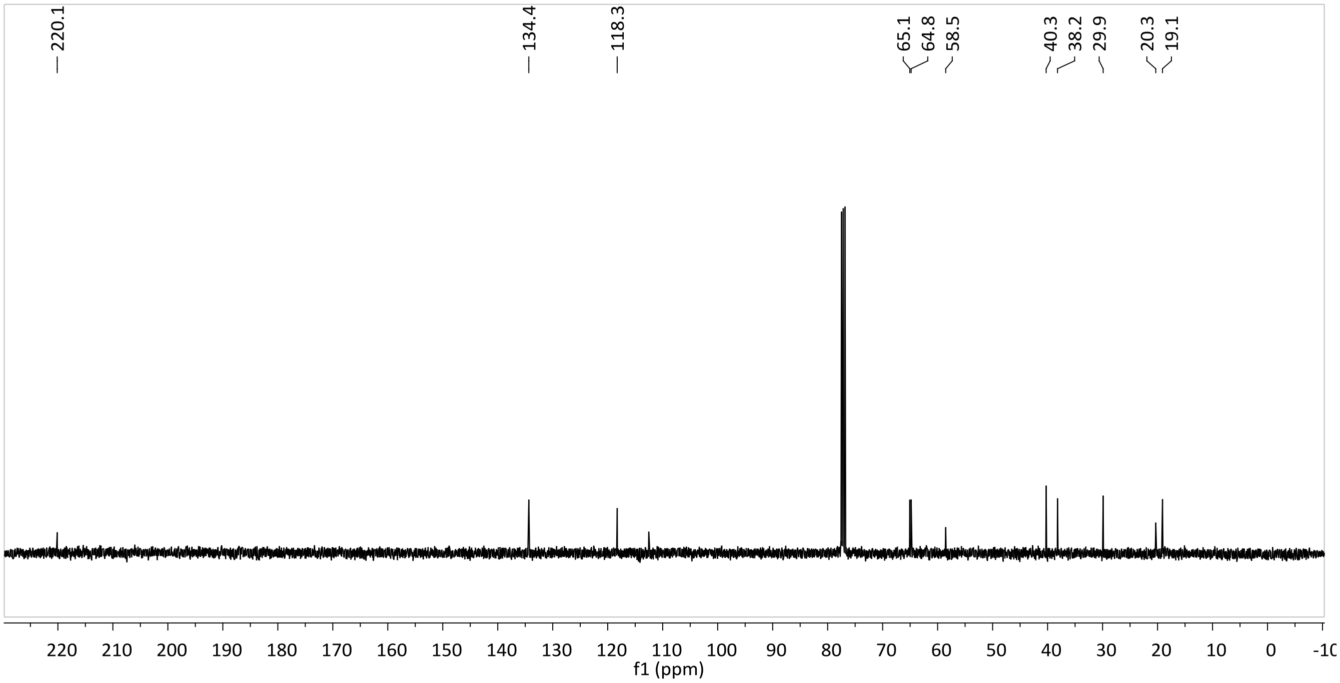
220.1
134.4
118.3
65.1
64.8
58.5
40.3
38.2
29.9
20.3
19.1
220 210 200 190 180 170 160 150 140 130 120 110 100 90 80 70 60 50 40 30 20 10 0 -10
f1 (ppm)

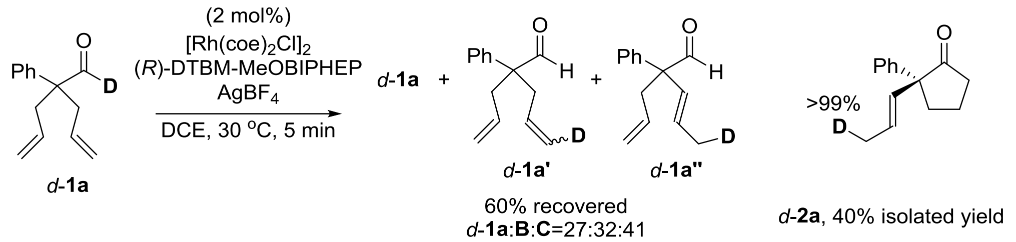

^{1}H and [2]D NMR for recovered aldehydes (equation 2)

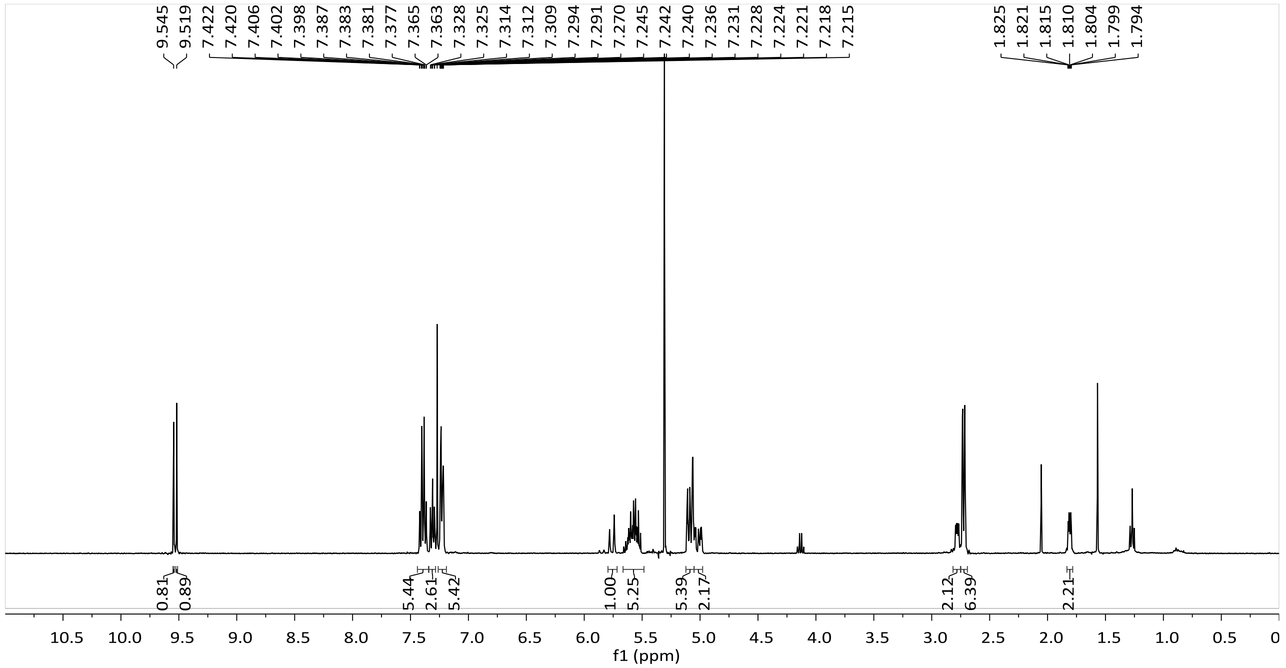

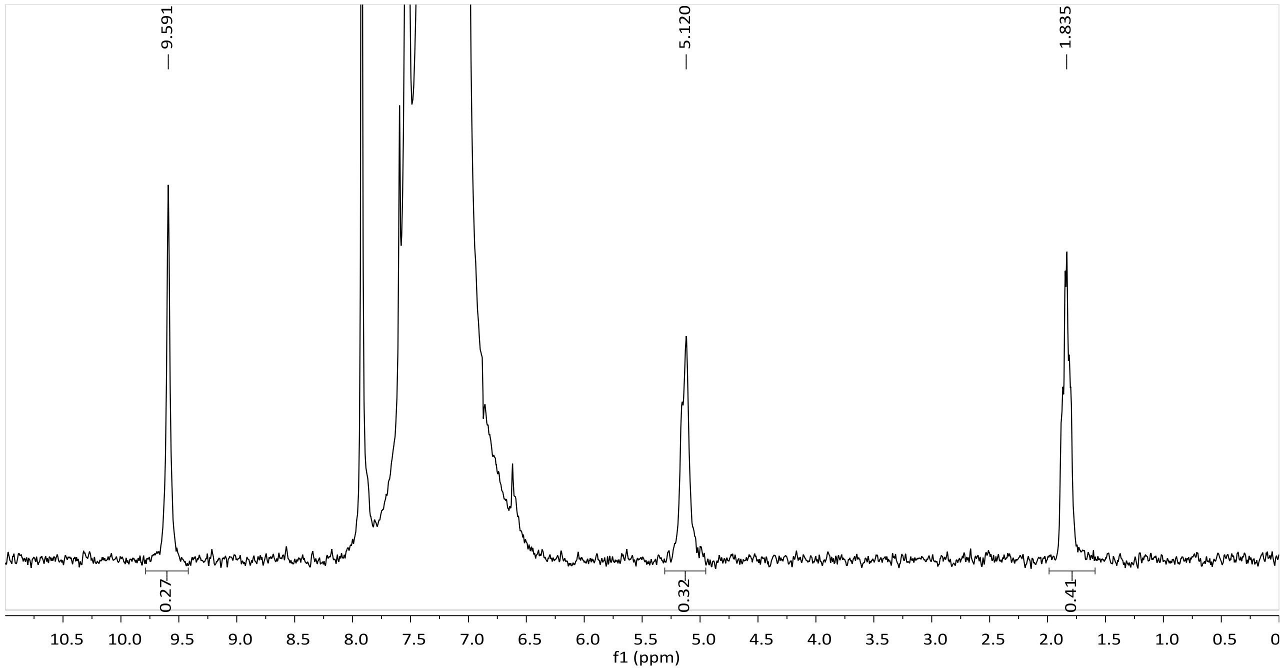

6 Chiral SFC Analysis

See Section 3 (S3) for details on chromatography conditions for each case.

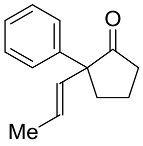

(±)-**2a**

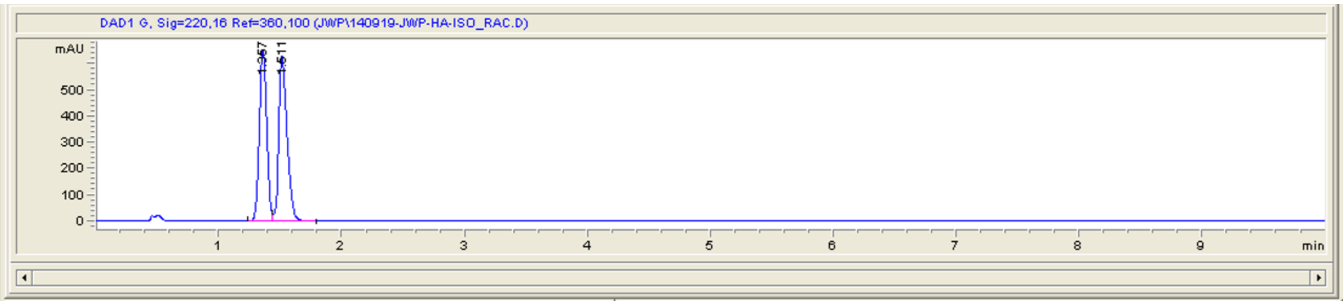

File Information

LC-File	140919-JWP-HA-ISO_RAC.D
File Path	C:\CHEM32\1\DATA\JWP\
[illegible]	[illegible]

#	Time	Area	Height	Width	Area%	Symmetry
1	1.357	2748.9	655	0.066	48.385	0.937
2	1.511	2932.3	630.1	0.0683	51.615	0.565

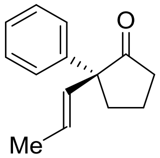

Enantioenriched (–)-**2a**

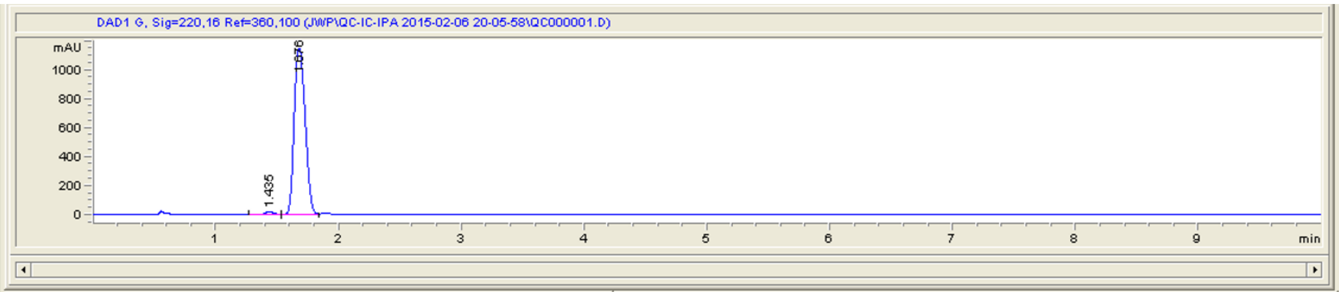

File Information

LC-File	QC000001.D
File Path	C:\CHEM32\1\DATA\JWP\QC-IC-IPA 2015-02-06 20-05-58\
[illegible]	[illegible]

#	Time	Area	Height	Width	Area%	Symmetry
1	1.435	110.4	20.1	0.0871	1.503	0.91
2	1.676	7236.4	1151.3	0.1023	98.497	0.745

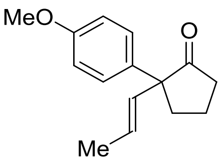

(±)-**2b**

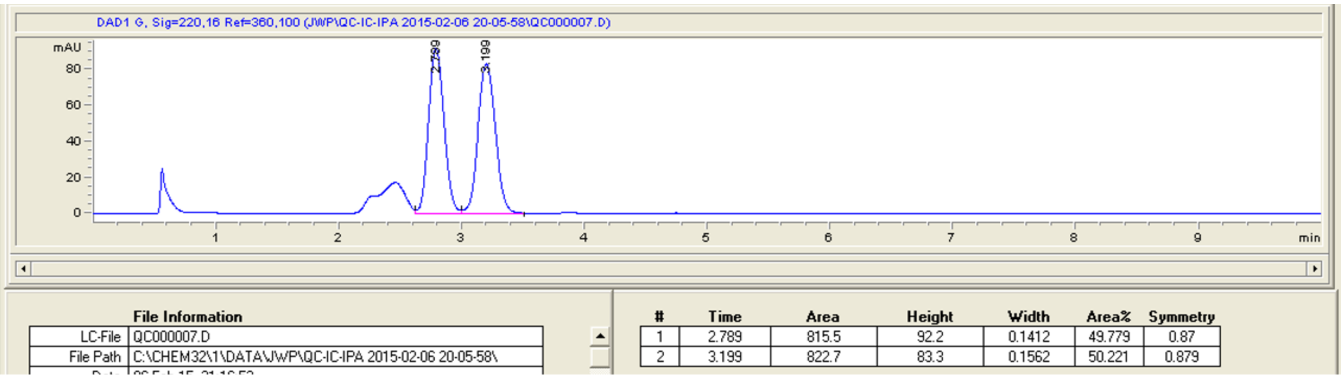

File Information

LC-File	QC000007.D
File Path	C:\CHEM32\1\DATA\JWP\QC-IC-IPA 2015-02-06 20-05-58\

#	Time	Area	Height	Width	Area%	Symmetry
1	2.789	815.5	92.2	0.1412	49.779	0.87
2	3.199	822.7	83.3	0.1562	50.221	0.879

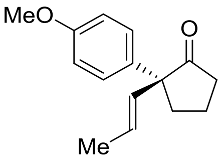

Enantioenriched (–)-**2b**

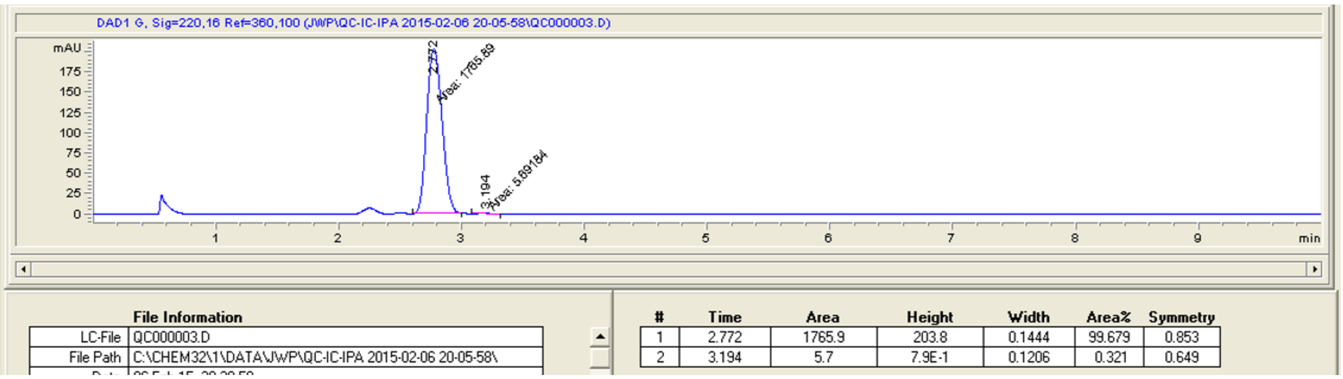

File Information

LC-File	QC000003.D
File Path	C:\CHEM32\1\DATA\JWP\QC-IC-IPA 2015-02-06 20-05-58\

#	Time	Area	Height	Width	Area%	Symmetry
1	2.772	1765.9	203.8	0.1444	99.679	0.853
2	3.194	5.7	7.9E-1	0.1206	0.321	0.649

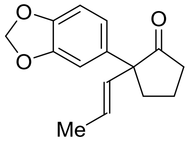

(±)-**2c**

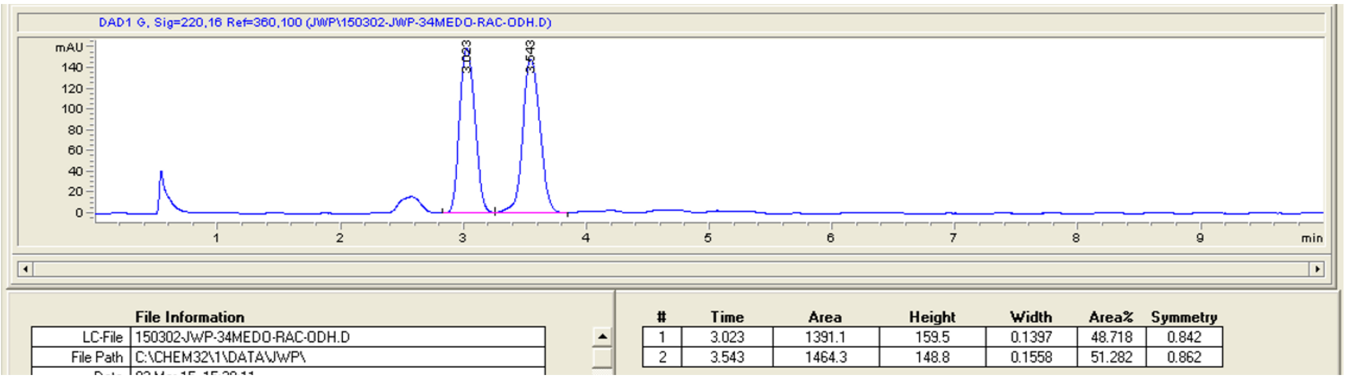

LC-File	150302-JWP-34MEDO-RAC-ODH.D
File Path	C:\CHEM32\1\DATA\JWP\
[illegible]	[illegible]

#	Time	Area	Height	Width	Area%	Symmetry
1	3.023	1391.1	159.5	0.1397	48.718	0.842
2	3.543	1464.3	148.8	0.1558	51.282	0.862

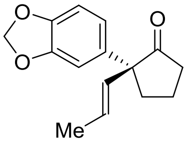

Enantioenriched (–)-**2c**

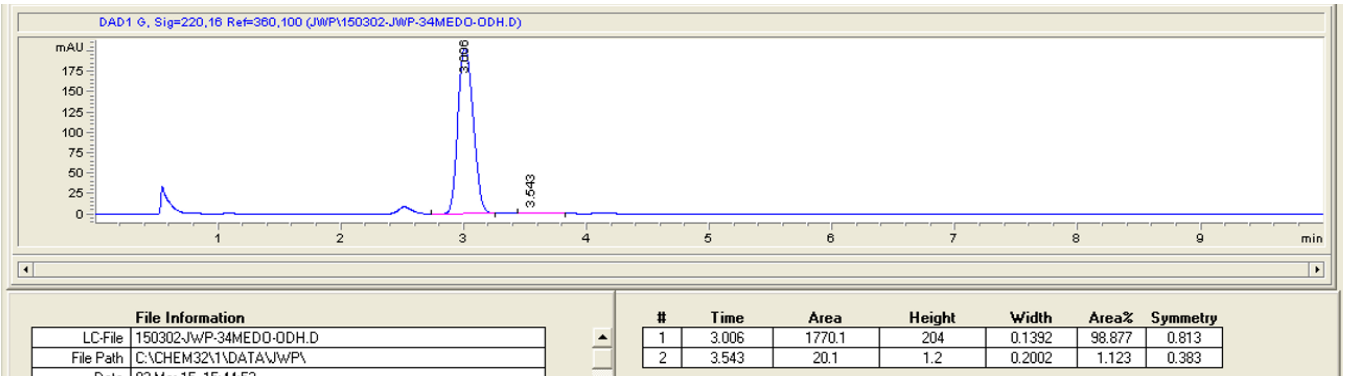

LC-File	150302-JWP-34MEDO-ODH.D
File Path	C:\CHEM32\1\DATA\JWP\
[illegible]	[illegible]

#	Time	Area	Height	Width	Area%	Symmetry
1	3.006	1770.1	204	0.1392	98.877	0.813
2	3.543	20.1	1.2	0.2002	1.123	0.383

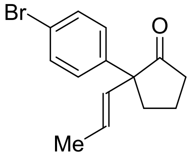

(±)-**2d**

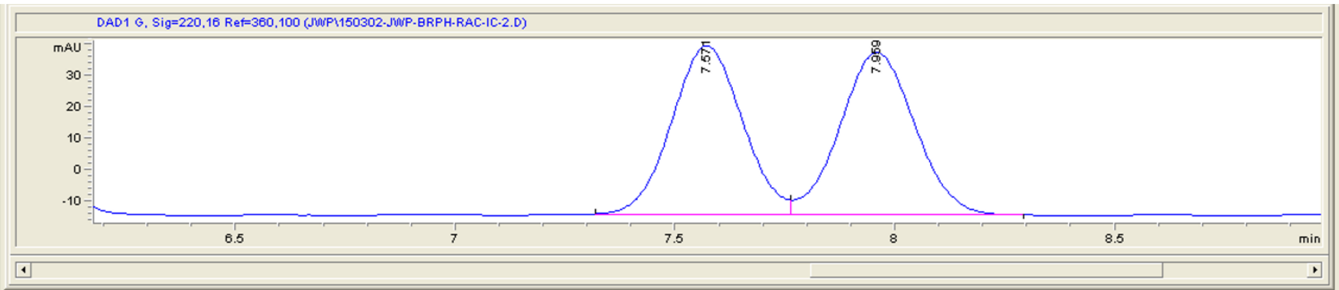

	File Information
LC-File	150302-JWP-BRPH-RAC-IC-2.D
File Path	C:\CHEM32\1\DATA\JWP\

#	Time	Area	Height	Width	Area%	Symmetry
1	7.571	604.9	53.9	0.174	49.532	0.958
2	7.959	616.3	51.9	0.1846	50.468	0.943

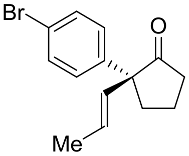

Enantioenriched (–)-**2d**

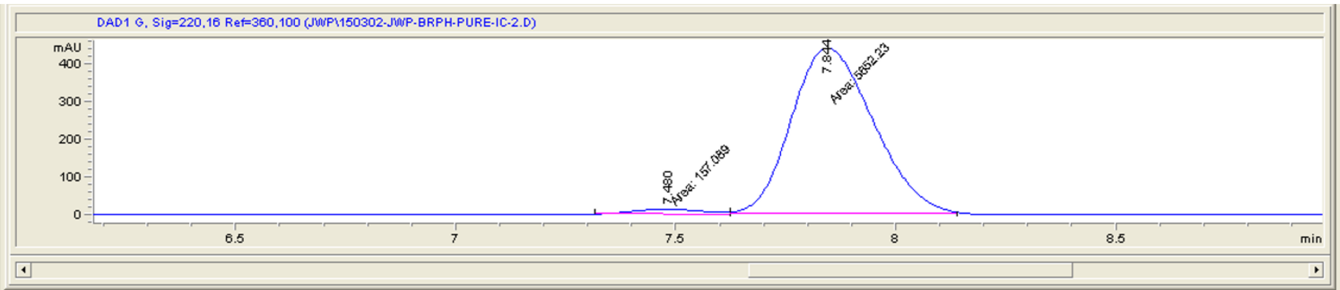

	File Information
LC-File	150302-JWP-BRPH-PURE-IC-2.D
File Path	C:\CHEM32\1\DATA\JWP\

#	Time	Area	Height	Width	Area%	Symmetry
1	7.48	157.1	13.9	0.1884	2.704	0.814
2	7.844	5652.2	438.1	0.2151	97.296	0.774

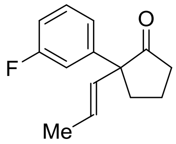

(±)-**2e**

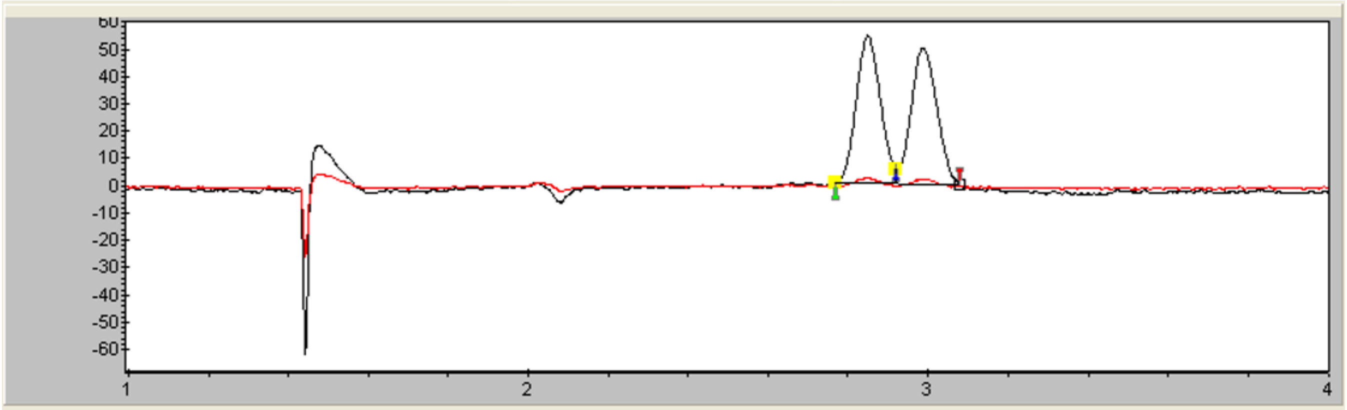

#	Name	Start [Min]	Time [Min]	End [Min]	RT Offset [Min]	Quantity [% Area]	Height [µV]	Area [µV.Min]	Area [%]
1	UNKNOWN	2.77	2.85	2.92	0.00	50.42	54.1	3.8	50.417
2	UNKNOWN	2.92	2.99	3.08	0.00	49.58	50.1	3.7	49.583
Total						100.00	104.2	7.5	100.000

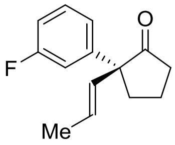

Enantioenriched (–)-**2e**

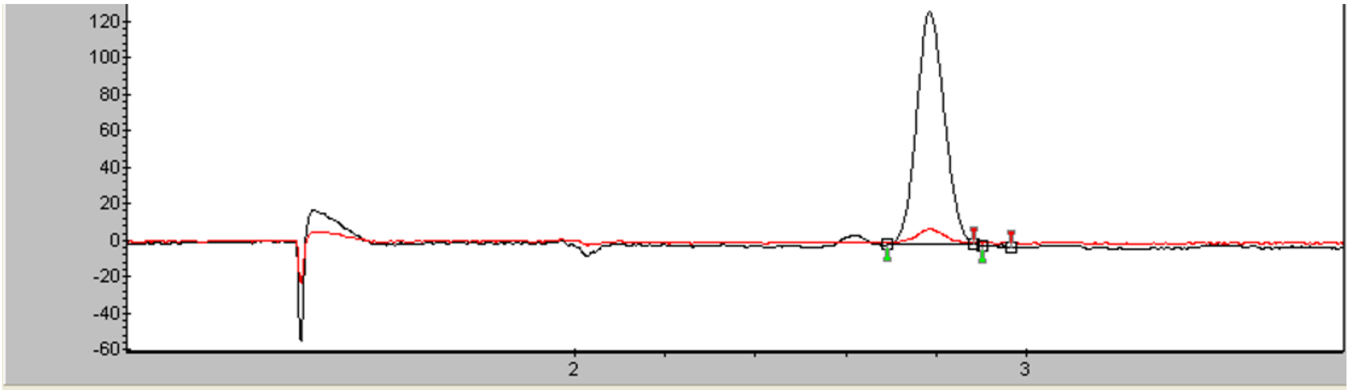

#	Name	Start [Min]	Time [Min]	End [Min]	RT Offset [Min]	Quantity [% Area]	Height [µV]	Area [µV.Min]	Area [%]
1	UNKNOWN	2.69	2.79	2.88	0.00	99.74	127.8	9.1	99.743
2	UNKNOWN	2.90	2.94	2.97	0.00	0.26	1.1	0.0	0.257
Total						100.00	128.9	9.1	100.000

* Peak corresponding to 2.6 is small impurity of **12e**.

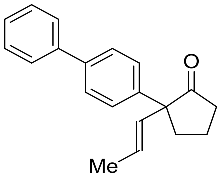

(±)-**2f**

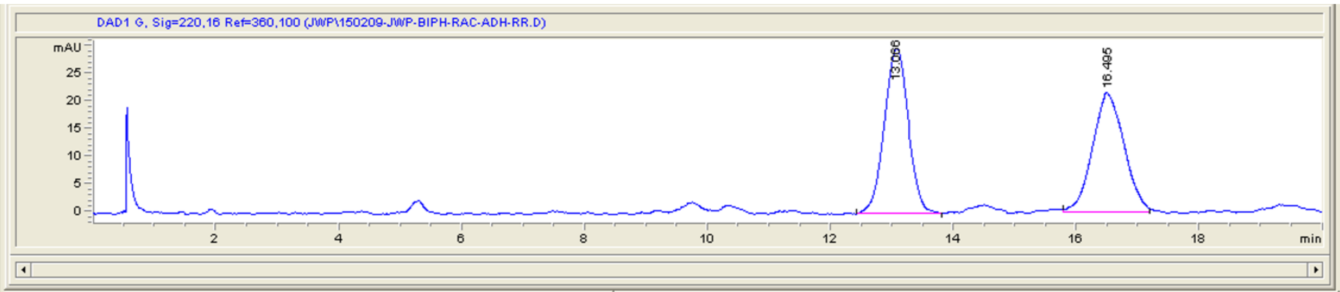

File Information

LC-File	150209-JWP-BIPH-RAC-ADH-RR.D
File Path	C:\CHEM32\1\DATA\JWP\

#	Time	Area	Height	Width	Area%	Symmetry
1	13.066	818.5	29.9	0.3859	51.095	0.991
2	16.495	783.4	21.6	0.4411	48.905	0.877

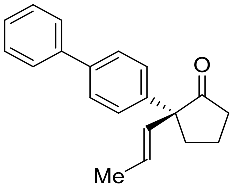

Enantioenriched (–)-**2f**

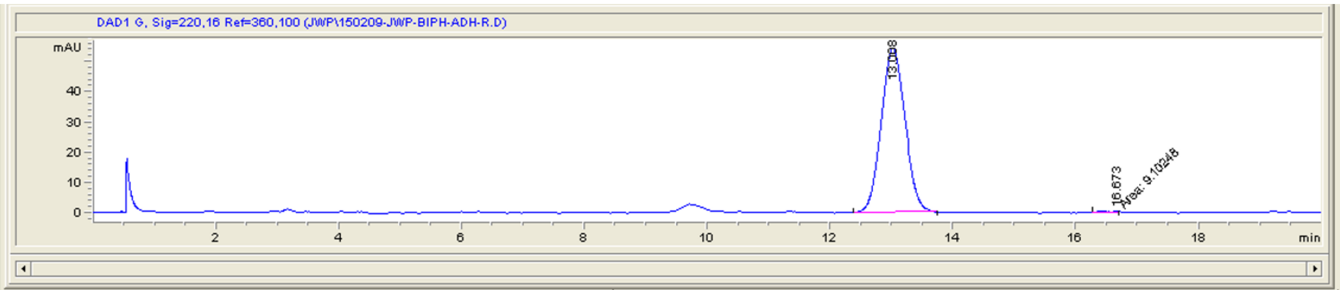

File Information

LC-File	150209-JWP-BIPH-ADH-R.D
File Path	C:\CHEM32\1\DATA\JWP\

#	Time	Area	Height	Width	Area%	Symmetry
1	13.008	1466.9	54.4	0.3815	99.383	0.874
2	16.673	9.1	4.2E-1	0.3627	0.617	15.473

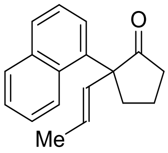

(±)-**2g**

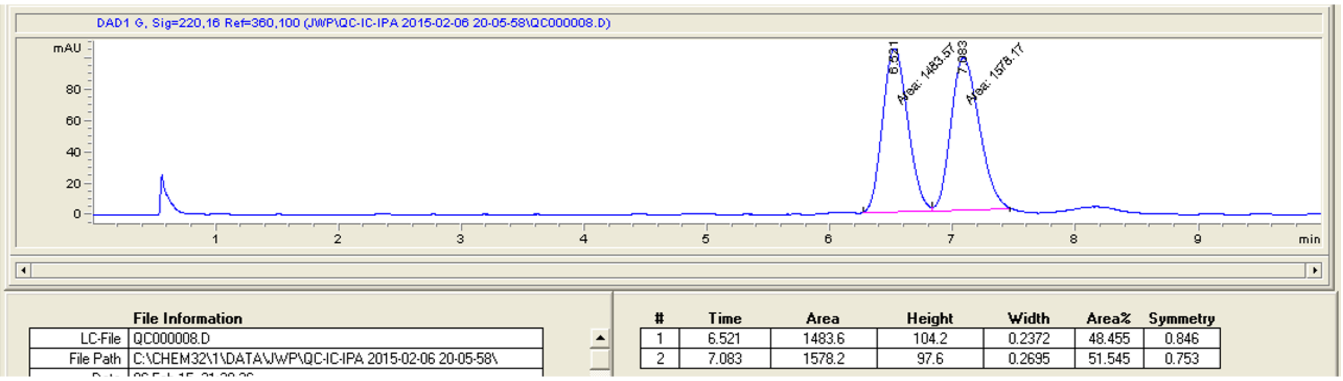

File Information

LC-File	QC000008.D
File Path	C:\CHEM32\1\DATA\JWP\QC-IC-IPA 2015-02-06 20-05-58\

#	Time	Area	Height	Width	Area%	Symmetry
1	6.521	1483.6	104.2	0.2372	48.455	0.846
2	7.083	1578.2	97.6	0.2695	51.545	0.753

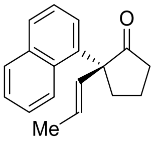

Enantioenriched (+)-**2g**

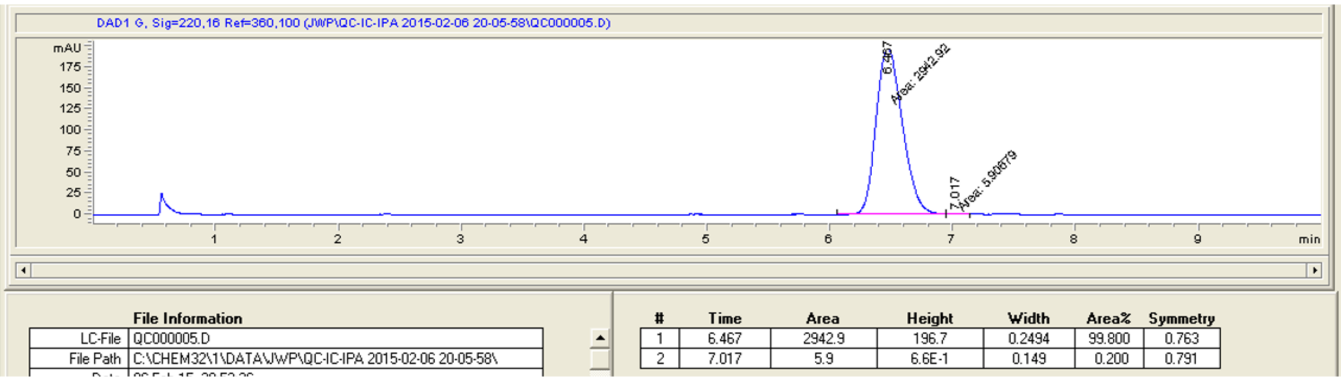

File Information

LC-File	QC000005.D
File Path	C:\CHEM32\1\DATA\JWP\QC-IC-IPA 2015-02-06 20-05-58\

#	Time	Area	Height	Width	Area%	Symmetry
1	6.467	2942.9	196.7	0.2494	99.800	0.763
2	7.017	5.9	6.6E-1	0.149	0.200	0.791

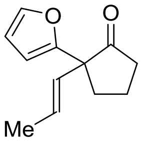

(±)-**2h**

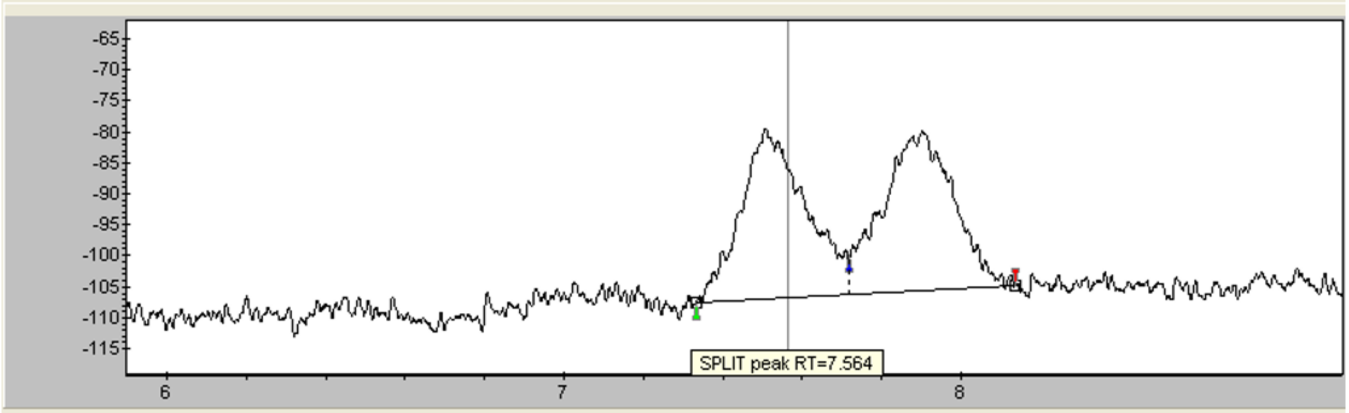

#	Name	Start [Min]	Time [Min]	End [Min]	RT Offset [Min]	Quantity [% Area]	Height [µV]	Area [µV.Min]	Area [%]
1	UNKNOWN	7.33	7.51	7.72	0.00	49.48	27.6	5.2	49.482
2	UNKNOWN	7.72	7.90	8.14	0.00	50.52	25.7	5.3	50.518
Total						100.00	53.2	10.5	100.000

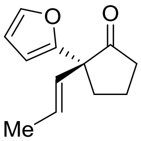

Enantioenriched **2i-reduction**

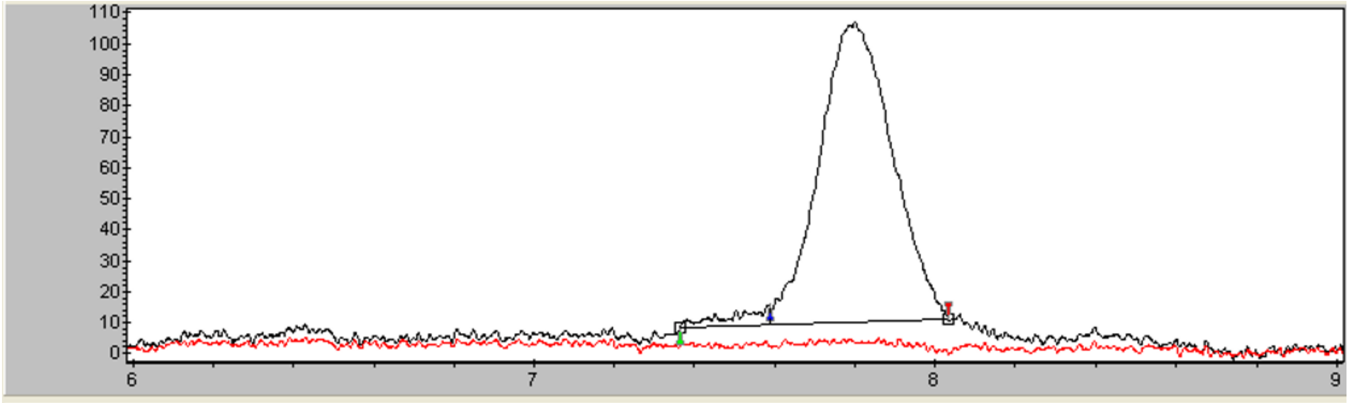

#	Name	Start [Min]	Time [Min]	End [Min]	RT Offset [Min]	Quantity [% Area]	Height [µV]	Area [µV.Min]	Area [%]
1	UNKNOWN	7.36	7.58	7.59	0.00	3.27	6.3	0.7	3.267
2	UNKNOWN	7.59	7.80	8.03	0.00	96.73	96.6	20.4	96.733
Total						100.00	102.9	21.1	100.000

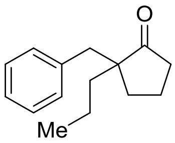

(±)-**2i-reduction**

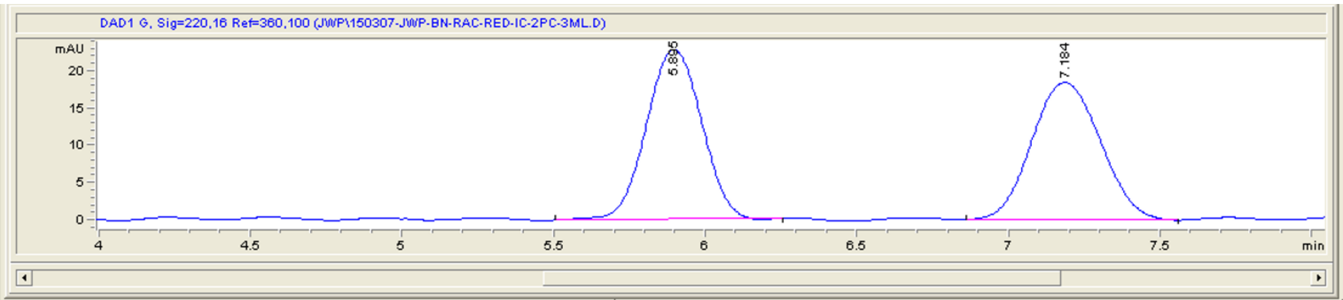

File Information	
LC-File	150307-JWP-BN-RAC-RED-IC-2PC-3ML.D
File Path	C:\CHEM32\1\DATA\JWP\
[illegible]	[illegible]

#	Time	Area	Height	Width	Area%	Symmetry
1	5.895	289.9	22.8	0.2002	49.683	0.965
2	7.184	293.6	18.5	0.2534	50.317	0.908

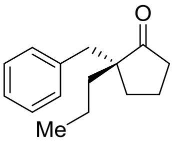

Enantioenriched **2i-reduction**

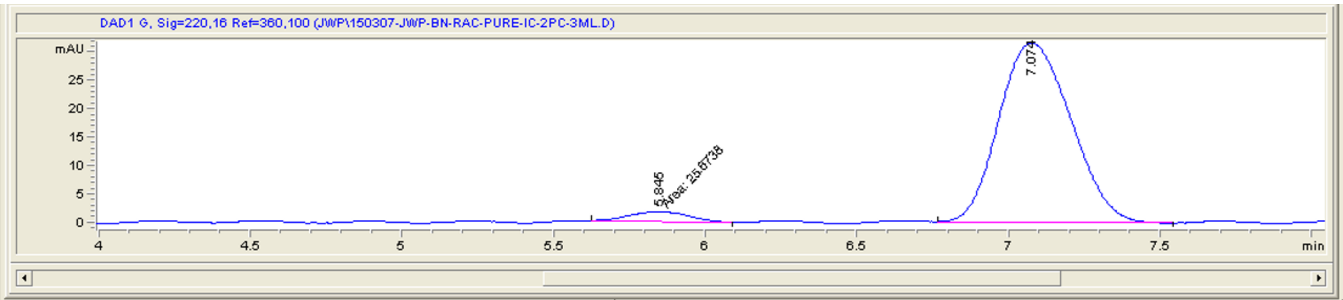

File Information	
LC-File	150307-JWP-BN-RAC-PURE-IC-2PC-3ML.D
File Path	C:\CHEM32\1\DATA\JWP\
[illegible]	[illegible]

#	Time	Area	Height	Width	Area%	Symmetry
1	5.845	25.7	1.8	0.2363	4.639	0.932
2	7.074	527.7	31.7	0.263	95.361	0.804

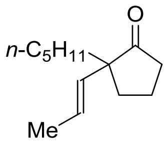

(±)-**2j**

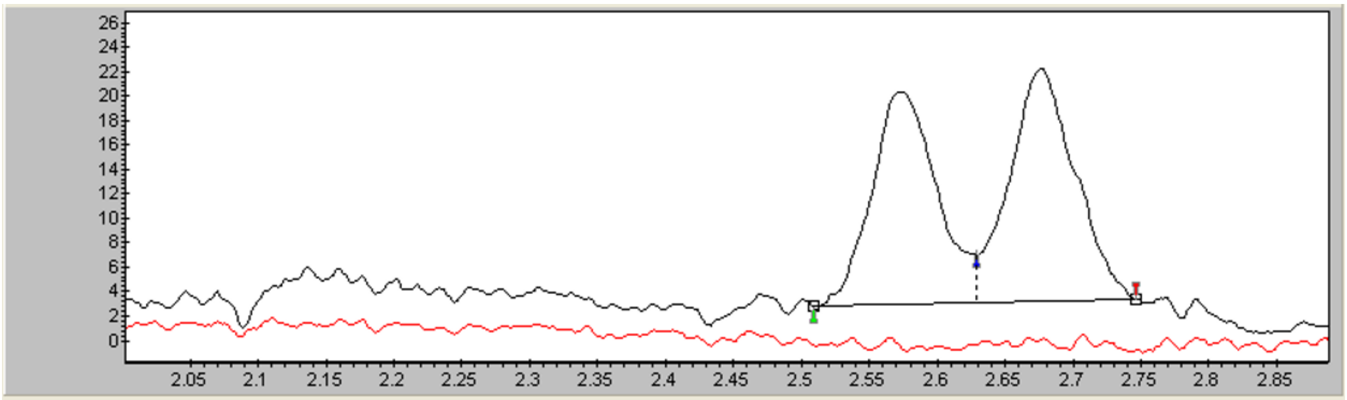

#	Name	Start [Min]	Time [Min]	End [Min]	RT Offset [Min]	Quantity [% Are	Height [µV]	Area [µV.Min]	Area [%]
1	UNKNOWN	2.51	2.57	2.63	0.00	47.39	17.3	1.0	47.386
2	UNKNOWN	2.63	2.68	2.75	0.00	52.61	19.0	1.1	52.614
Total						100.00	36.4	2.0	100.000

*Note: This compound has low UV activity and was detected at 215 nm wavelength.

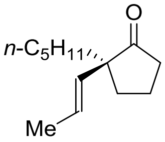

Enantioenriched (–)-**2j**

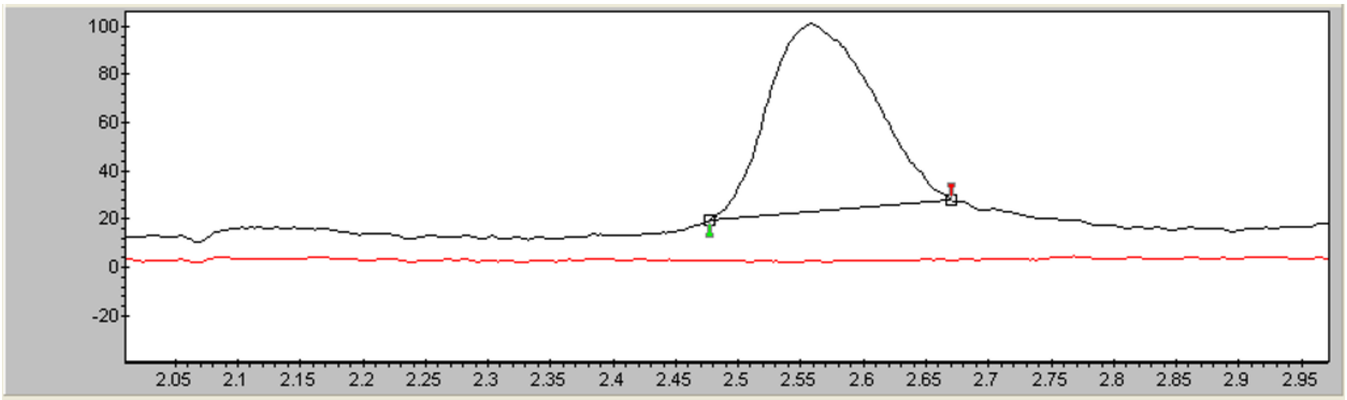

#	Name	Start [Min]	Time [Min]	End [Min]	RT Offset [Min]	Quantity [% Are	Height [µV]	Area [µV.Min]	Area [%]
1	UNKNOWN	2.48	2.56	2.67	0.00	100.00	77.7	7.5	100.000
Total						100.00	77.7	7.5	100.000

*Note: This compound has low UV activity and was detected at 215 nm wavelength.

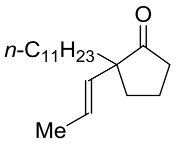

(±)-**2k**

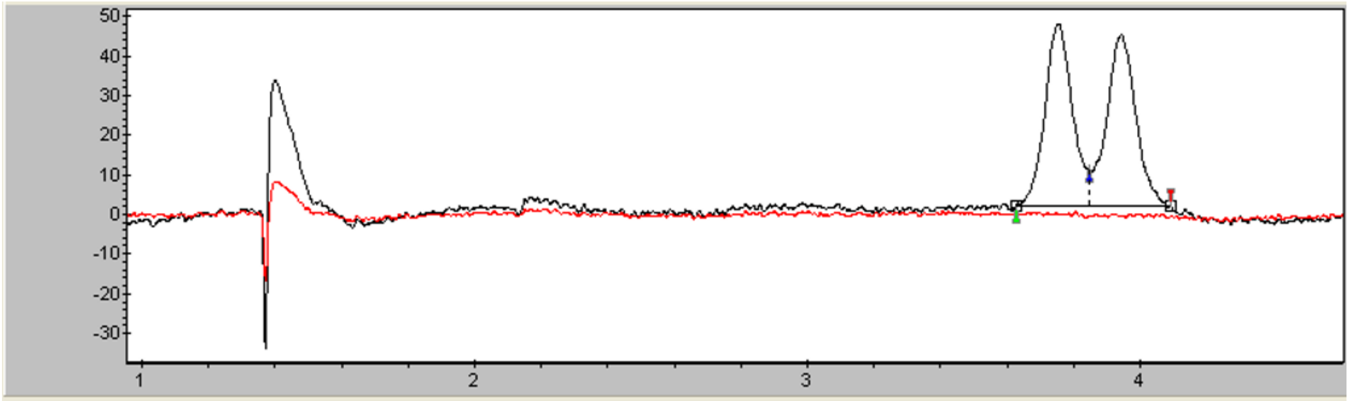

#	Name	Start [Min]	Time [Min]	End [Min]	RT Offset [Min]	Quantity [% Area]	Height [µV]	Area [µV.Min]	Area [%]
1	UNKNOWN	3.63	3.76	3.85	0.00	50.15	45.9	4.4	50.151
2	UNKNOWN	3.85	3.95	4.09	0.00	49.85	43.4	4.4	49.849
Total						100.00	89.3	8.9	100.000

*Note: This compound has low UV activity and was detected at 220 nm wavelength.

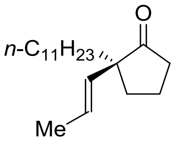

Enantioenriched (–)-**2k**

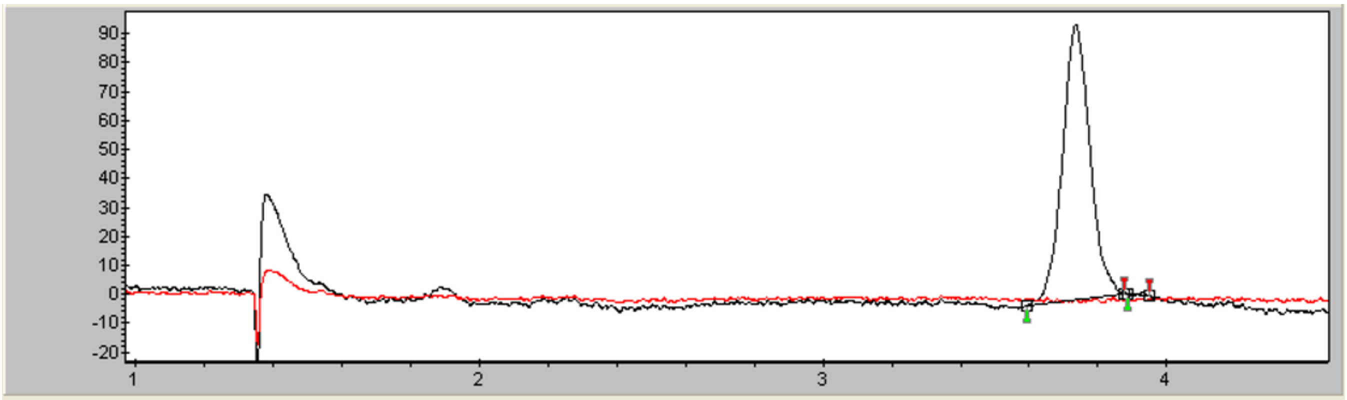

#	Name	Start [Min]	Time [Min]	End [Min]	RT Offset [Min]	Quantity [% Area]	Height [µV]	Area [µV.Min]	Area [%]
1	UNKNOWN	3.59	3.74	3.88	0.00	100.00	95.3	8.8	99.998
2	UNKNOWN	3.89	3.93	3.95	0.00	0.00	1.0	0.0	0.002
Total						100.00	96.3	8.8	100.000

*Note: This compound has low UV activity and was detected at 215 nm wavelength.
